# Dynamic transcriptomic profiles of zebrafish gills in response to zinc supplementation

**DOI:** 10.1186/1471-2164-11-553

**Published:** 2010-10-11

**Authors:** Dongling Zheng, Peter Kille, Graham P Feeney, Phil Cunningham, Richard D Handy, Christer Hogstrand

**Affiliations:** 1 Mineral Metabolism Group, Nutritional Sciences Division, King's College London, London SE1 9NH, UK; 2 School of Biosciences, University of Cardiff, Cardiff, CF10 3TL, UK; 3 School of Biological Sciences, University of Plymouth, Plymouth, PL4 8AA, UK; 4 Current Address: Division of Infection and Immunity, Windeyer Institute of Medical Sciences, University College London, London W1T 4JF, UK

## Abstract

**Background:**

Dietary zinc supplementation may help to promote growth, boost the immune system, protect against diabetes, and aid recovery from diarrhoea. We exploited the zebrafish (*Danio rerio*) gill as a unique vertebrate ion transporting epithelium model to study the time-dependent regulatory networks of gene-expression leading to homeostatic control during zinc supplementation. This organ forms a conduit for zinc uptake whilst exhibiting conservation of zinc trafficking components.

**Results:**

Fish were maintained with either zinc supplemented water (4.0 μM) and diet (2023 mg zinc kg^-1^) or water and diet containing Zn^2+ ^at 0.25 μM and 233 mg zinc kg^-1^, respectively. Gill tissues were harvested at five time points (8 hours to 14 days) and transcriptome changes analysed in quintuplicate using a 16 K microarray with results anchored to gill Zn^2+ ^influx and whole body nutrient composition (protein, carbohydrate, lipid, elements). The number of regulated genes increased up to day 7 but declined as the fish acclimated. In total 525 genes were regulated (having a fold-change more than 1.8 fold change and an adjusted P-value less than 0.1 which is controlling a 10% False discovery rate, FDR) by zinc supplementation, but little overlap was observed between genes regulated at successive time-points. Many genes displayed cyclic expression, typical for homeostatic control mechanisms. Annotation enrichment analysis revealed strong overrepresentation of "transcription factors", with specific association evident with "steroid hormone receptors". A suite of genes linked to "development" were also statistically overrepresented. More specifically, early regulation of genes was linked to a few key transcription factors (e.g. Mtf1, Jun, Stat1, Ppara, Gata3) and was followed by hedgehog and bone morphogenic protein signalling.

**Conclusions:**

The results suggest that zinc supplementation reactivated developmental pathways in the gill and stimulated stem cell differentiation, a response likely reflecting gill remodelling in response to its altered environment. This provides insight to the role of zinc during cell differentiation and illustrates the critical nature of maintaining zinc status. The study also highlights the importance of temporal transcriptomics analysis in order resolve the discrete elements of biological processes, such as zinc acclimation.

## Background

One of the signatures of life is the ability of organisms to maintain internal concentrations of ions independent of their environment [1]. The homeostatic control of zinc is a notable example of this capability; most vertebrates contain approximately 0.5 mmol zinc kg^-1^, which is about 60,000 times higher than that of typical uncontaminated waters [2]. Zinc is an essential micronutrient for all organisms. There are estimated 3,000 zinc binding proteins in the human genome of which over 300 are enzymes of all classes [3]. However, the majority of zinc-proteins use zinc for structural purposes, generating folds characteristic of zinc- and RING-finger domains (C3HisC4 type Zinc fingers). In addition, there is an increasing appreciation for the involvement of cytosolic Zn^2+ ^transients in cell signalling processes through its interactions with specific enzymes [4,5]. Therefore, concentration of free Zn^2+ ^in the cytoplasm has to be maintained in the picomolar range to avoid triggering signalling events [4-6]. Acquiring appropriate zinc to maintain this balance is a particular challenge for aquatic organisms, such as freshwater fish, which may encounter water zinc concentrations ranging five orders of magnitude, 0.005 - 1,000 μg L^-1 ^[2,7]. It is evident from physiological studies that zinc regulation in fish is indeed highly dynamic and efficient [8-10].

In vertebrate cells zinc is tightly controlled by uptake, efflux, and compartmentalisation to maintain adequate levels and prevent toxic overaccumulation. Several zinc uptake transporters have been characterised and they are mainly from the ZIP family (Zrt, Irt-like proteins; Slc39) of metal transporters [5,11]. Although exceptions exist, expression of ZIP transporters or recruitment of these to the plasma membrane is typically increased in zinc-limited conditions, while zinc excess generally results in down-regulation of ZIP expression and abundance at the plasma membrane. The efflux transporters, called the ZnT family (zinc transporter; Slc30), are members of the cation diffusion facilitator (CDF) super-family of metal ion transporters. At least nine ZnT proteins have been found in mammalian cells [5,11]. ZnT1 was the first zinc transporter to be identified in mammals [12] and fish [13] and was shown to be responsible for extrusion of zinc from the cell. With the exception of a splice variant of ZnT5, which is localised to the plasma membrane and can mediate bi-directional movement of zinc [14,15], all other characterised ZnT proteins regulate zinc transport into different intracellular membrane compartments [5]. The zebrafish genome carries a set of zinc transporters that map very closely to the human repertoire and only in a single instance (zip8) does the zebrafish have a zinc transporter paralog not present in mammals [11]. As in mammals expression of several zebrafish zinc transporters is regulated by zinc status [10].

Another important system that controls the concentration of labile Zn^2+ ^in cells is the metallothioneins (MTs) [16]. MTs are a family of low molecular weight and high metal content proteins ubiquitously present in animals, plants, fungi, and cyanobacteria. They control the concentration of labile Zn^2+ ^by binding or releasing zinc when necessary [16]. They are induced by toxic metals such as cadmium and silver, or essential trace elements such as zinc and copper [17]. There are four principal isoforms of MT genes in mammals, among which MT-I and MT-II function in non-neural tissues to detoxify heavy metals, regulate zinc and copper homeostasis, and limit oxidative damage. The MTs found in different fish species are homologous to the mammalian MT and are functionally equivalent to the MT-I and MT-II isoforms [18]. Zebrafish has at least two in-species paralogs of MT, designated Mt1 and Mt2 [19,20].

Zinc-driven transcription of MT genes is controlled by metal-regulatory transcription factor 1 (MTF1) [21]. Upon association of zinc to its zinc-finger domain in the cytosol, MTF1 moves to the nucleus where it binds to metal-response elements (MREs) in target genes. In addition to MT, ZnT1 is also induced by MTF1 and we recently showed that zebrafish Mtf1 is a repressor of *zip10 *gene expression [10,22,23]. There are several other genes regulated by Mtf1, but it is not obvious how or if these are involved in zinc homeostasis [22]. Interestingly, there is a remarkable overrepresentation of genes involved in organism development among predicted zebrafish Mtf1 targets. This fits well with observations in mouse that MTF1 is essential for embryonic organ development, whereas targeted deletion post-partum is not lethal [22,23]. Although MTF1 is an important and specific zinc signalling factor there is increasing evidence for direct roles of zinc signalling at all levels of signal transduction, including modulatory effects on protein tyrosine phosphatases and Ca^2+^-ATPases [5,24-26]. Thus, the cellular regulation of zinc is complex and zinc itself is intimately involved in regulation of numerous cellular processes.

Changes in cellular zinc levels evoke complex responses within an organism, making it difficult to explain effects observed at the organ and organism levels based on one or a few regulatory entities. Because of this an integrated functional genomics approach to study zinc homeostasis is appropriate. There are several informative publications on global changes in gene expression of cultured cells following zinc supplementation [22,27-31]. Less is known about effects of zinc supplementation on gene expression profiles *in vivo*, with animal studies showing transcriptome responses at single time-points in oesophagus, liver and brain [31-36]. To understand regulation of zinc uptake it would be useful to have information on time-dependent transcriptional responses at the site of uptake. The fish gill is arguably a perfect organ for such studies as it is in direct contact with surrounding water, has well-defined zinc transporting cells, is responsible for a substantial part of the total zinc uptake, and is also the primary target organ for zinc toxicity [6].

We have studied the 14-day time-course of changes in gene expression of zinc transporters in gills of zebrafish treated with zinc depletion or supplementation in water and diet [10,11]. Of the 15 zinc transporter genes analysed, four (*znt1*, *znt5*, *zip3 *and *zip10*) showed consistent changes in mRNA levels in the gills in response to changes in zinc supply. Therefore the aims of the present study were to determine the time-course of global gene expression changes in the gill linked to changes in zinc uptake associated with zinc supplementation, and to use this data to reverse-engineer the biological processes and regulatory networks involved in the acclimation process. A 16 K oligonucleotide microarray was employed to quantify global transcript changes within zebrafish gill during a 14-day time course of acclimation to zinc supplemented diet and water. The physiological response of the organism was assessed by measuring gill Zn^2+ ^influx and whole body nutrient composition (protein, carbohydrate, lipid, elements). The analyses revealed maximal alteration in gene expression at day 7, followed by decline as the fish acclimated with a total of 525 genes whose expression was impacted by zinc supplementation independent of time. Many genes displayed cyclic expression, typical for homeostatic control mechanisms. Annotation enrichment analysis revealed overrepresentation of a number of functional categories including transcription factors with specific association evident with steroid hormone receptors. Additional pathways overrepresented link to developmental processes, which were dominated by genes involved in development of anatomical structure, nervous system, and cellular differentiation. A significant bias in genes associated with the insulin signalling pathway was also observed, together with differential expression of molecules involved in apoptosis, cell division, and DNA damage. Analysis of transcription factor binding sites in the regulatory region of genes exhibiting altered expression resolved initial regulation of genes responsive to a few key transcription factors (e.g. Mtf1, Jun, Stat1, Ppara, Gata3) followed by hedgehog and bone morphogenic protein signalling, possibly leading to stem cell differentiation. Corresponding analysis of the functional pathways and regulatory network associated with zebrafish subjected to zinc depletion is presented in a parallel paper [37].

## Results

To identify genes regulated under zinc supplementation we utilised a convenient model system, zebrafish gills, combined with microarray gene expression analysis. Zinc was introduced to a group of fish in continuous flow of reconstituted water with a final zinc concentration of 4.0 μM in the water and 30.9 mmol kg^-1 ^in the diet, compared to a control group with 0.25 μM and 3.56 mmol kg^-1 ^of zinc in the water and diet, respectively. The experiment was carried out for 14 days with sampling points at 8 hours, 1, 4, 7, and 14 days.

### Physiological characterisation of zinc treatment

**Figure 1:**
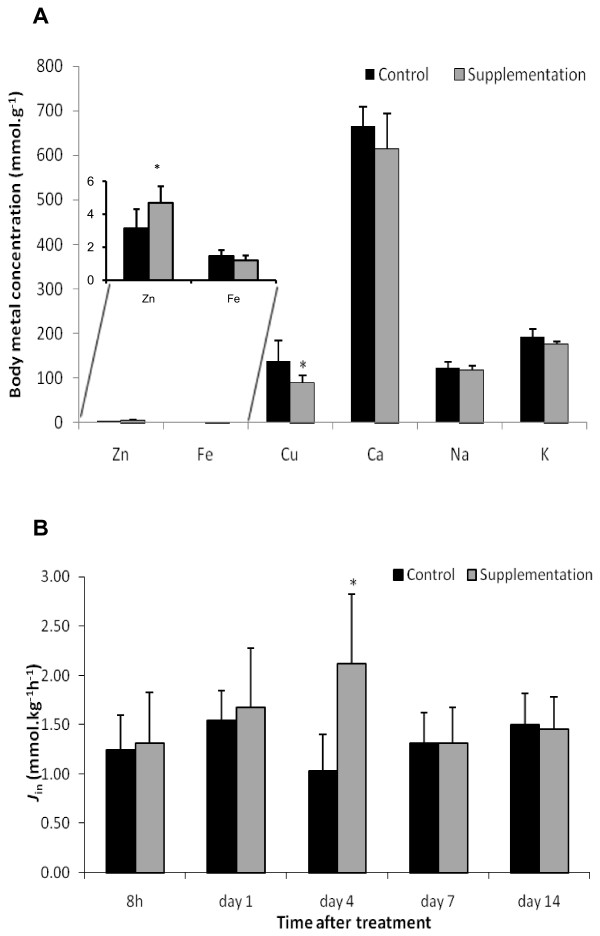
**Physiological changes of zebrafish after zinc supplementation**. Panel A: Elemental composition of whole body (minus gill) determined by ICP-MS. Panel B: Unidirectional Zn^2+ ^influx across the gills. Zebrafish treated with zinc supplementation in feed and water for 14 days compared with fish kept at the standard control condition. The bars represent the mean of nine fish for elemental composition or eight fish for Zn^2+ ^influx analysis; error bars denote SD. A statistical difference between zinc supplementation group and the control is shown by an asterisk (*P *< 0.05).

The zinc supplementation regime caused no effects on survival, body weight, lipid or protein content at the end of the two-week experiment (data not shown). At the termination of the experiment a spectrum of whole body elements was analysed, including Zn, Cu, Ca, Fe, Na, and K, in both zinc supplemented and control fish. Two metal ions exhibited significant (*P *< 0.05) alterations in body load: zinc, which increased by 50% over the control, and copper, which decreased by 34% (Figure 1A). There was also an 18% decrease in whole body iron, but this was not statistically significant (*P *= 0.054; Figure 1A).

It was of interest to examine any alteration in the flux of zinc across the fish gill to evaluate whether the organism is moderating zinc uptake rates in response to the elevated zinc concentrations within the diet and water. Since an important pathway to control zinc homeostasis is flux across the gill surface we evaluated zinc uptake across the gill in eight fish at each time-point using a unidirectional zinc influx assay (Figure 1B). Compared to the control group the zinc influx of the zinc supplemented group gradually increased, reached a peak at day 4, and decreased thereafter to equal that observed within the control group after 7 days of exposure. This increase in zinc uptake was contrary to the expected flux compensation, which was predicted to reduce zinc influx in response to supplementation. The interpretation of this observation is therefore unclear. It is possible that the up-regulation of zinc chelator (Mt) and basolateral exporter (Znt1) resulted in an increased rate of transcellular zinc movement, while keeping intracellular labile Zn^2+ ^at a manageable concentration.

### Real-time PCR validation

Since the present study was part of a larger study involving the effect of zinc depletion, a comprehensive joint qPCR validation of the microarray method was carried out, and the result is presented in the parallel article [37]. The validation involved qPCR analysis of mRNA abundance for 10 genes using nine biological replicates (gills from 9 fish) per group and per time point, including the five gill samples used for microarrays. The directionality of responses to zinc supplementation was in full agreement between microarrays and qPCR.

### General effect of zinc status on differential gene expression in gills

Five fish were randomly withdrawn at each of five time points (8 hours, 1, 4, 7, and 14 days) from the zinc supplemented and control groups (across four tanks of each group), and their gills removed for gene expression analysis. One control sample from day 4 was removed because of failure to pass quality control. Thus, a total number of 49 microarrays were hybridised and analysed with *N *= 4 or 5. Of approximately 16,137 zebrafish reporters 10,521 provided a significant (*p *> 0.05) signal in at least three independent arrays. This indicates that at least 65.2 ± 5.7% of all analysed genes were expressed in gill tissue independent of zinc treatment.

**Figure 2:**
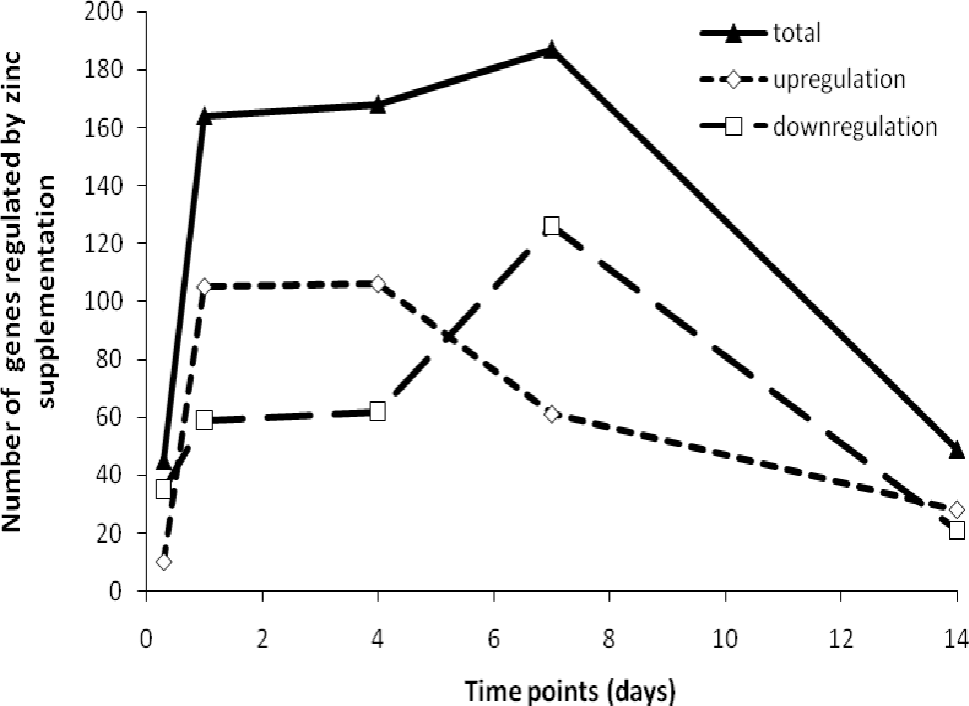
**Time-course of numbers of genes called regulated by zinc supplementationin gills of zebrafish**. Genes were called significantly regulated with greater than 1.8-fold change, compared to control, and FDR of 0.1 (*N *= 4 or 5 from each group at each time point). The triangles and solid line show the total number of regulated genes at each time-point, diamonds and short-dashed line give the numbers of up-regulated genes, and the numbers of down-regulated genes are indicated by rectangles and long-dash line.

Gene expression in gills of fish maintained in zinc supplemented feed and water was compared to branchial gene expression in fish under normal zinc supply at each time point. Genes having a fold-change greater than 1.8 and an adjusted P-value less than 0.1, controlling for a 10% false discovery rate (FDR) with the Benjamini-Hochberg method, were regarded as differentially regulated by zinc depletion compared to control group. A total of 525 genes showed differential expression at one or more time points (Additional file 1, Table S1). There was a remarkable variation in gene expression over time and of the 16 K reporters interrogated, only those for *mt2 *were consistently regulated in one direction (up-regulated) throughout the experiment. Expression of many transcripts appeared to oscillate, showing an up-regulation at one time point and down-regulation at another. The number of genes with up- or down-regulation across five time points is shown in Figure 2. It can be clearly seen that zinc supplementation induced maximal differential gene regulation at day 7, whilst the number of transcripts displaying significantly altered expression at 8 hours or 14 days were four-fold less. This latter observation indicates that cellular compensation mechanisms, such as reduced apical influx, increased basolateral efflux (i.e. expression of *znt1*), and cytosolic chelating capacity. i.e. elevation of Mt, might have been well established by 14 days of exposure. In the zinc supplementation condition many more genes were up-regulated than down-regulated at days 1 and 4; however, the trend was reversed at day 7.

### Functional classification of 'known' genes

In order to investigate the global impact zinc supplementation had on biological processes and molecular functions we performed annotation enrichment analysis to determine whether genes associated with specific gene ontology terms were significantly overrepresented within our lists of differentially regulated genes. Initial analysis interrogating the database with the Unigene identifiers, assigned to the zebrafish genes targeted by our reporters, yielded less than 20% (100 of the 525 genes) recognised by the DAVID (Database for Annotation, Visualisation and Integrated Discovery) functional annotation tool [38] (Table 1). The consequence of this low direct association with functional annotation was that ontological analysis of the gene lists from individual time points, where substantially smaller gene lists were involved, had low power. However, combined consideration of all genes regulated across all time points revealed significant enrichment of ontology terms associated with transcriptional responses such as 'regulation of gene expression', and indicated a strong association of zinc and hormonal control through overrepresentation of 'steroid hormone receptor activity' and 'ligand-dependent nuclear receptor activity' (Table 1), the latter category containing the retinoic acid receptor beta.

**Table 1:** Annotation enrichment analysis of genes regulated in gills by zinc supplementation on DAVID, using zebrafish Unigene gene identifiers.

Annotation Type*	Category Name	Genes	P-value**
GOTERM_MF_ALL	sequence-specific DNA binding	10	7.40E-04
GOTERM_MF_ALL	ligand-dependent nuclear receptor activity	4	3.50E-03
GOTERM_MF_ALL	steroid hormone receptor activity	4	3.50E-03
GOTERM_BP_ALL	regulation of biological process	16	3.70E-03
GOTERM_BP_ALL	regulation of cellular process	15	6.40E-03
GOTERM_BP_ALL	biological regulation	17	6.80E-03
GOTERM_CC_ALL	nucleus	15	1.00E-02
GOTERM_MF_ALL	transcription factor activity	9	1.20E-02
GOTERM_MF_ALL	transcription regulator activity	10	1.50E-02
GOTERM_BP_ALL	regulation of cellular metabolic process	11	2.30E-02
GOTERM_CC_ALL	intracellular part	22	2.40E-02
GOTERM_BP_ALL	regulation of gene expression	11	2.50E-02
GOTERM_BP_ALL	regulation of metabolic process	11	2.50E-02
GOTERM_BP_ALL	regulation of transcription, DNA-dependent	10	2.70E-02
GOTERM_BP_ALL	transcription, DNA-dependent	10	3.00E-02
GOTERM_BP_ALL	RNA biosynthetic process	10	3.10E-02
GOTERM_MF_ALL	DNA binding	11	3.20E-02
GOTERM_CC_ALL	membrane-bound organelle	16	4.40E-02
GOTERM_CC_ALL	intracellular membrane-bound organelle	16	4.40E-02
GOTERM_BP_ALL	regulation of transcription	10	4.90E-02

*MF is the abbreviation for molecular function, BP for biological process and CC for cellular component.

** Categories enriched in the data-set at *P *< 0.05 and containing three or more genes are listed.

**Figure 3:**
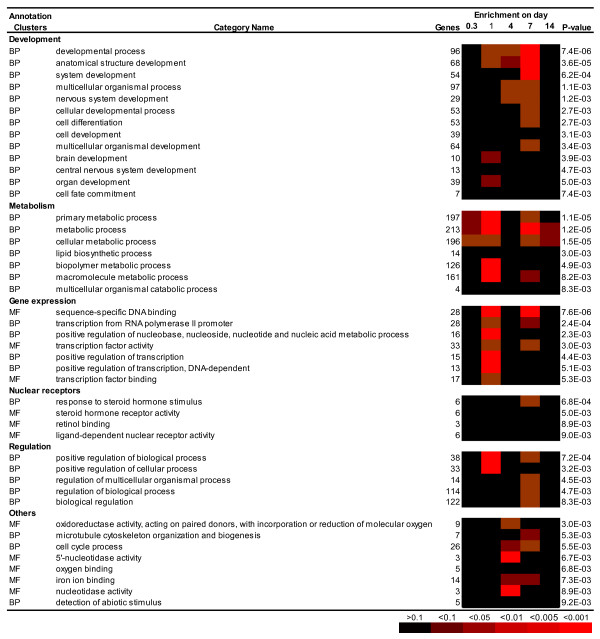
**Gene Ontology enrichment analysis of genes regulated by zinc supplementation in zebrafish gill**. The closest human homologue to each regulated zebrafish gene at each time point was submitted to the online DAVID functional annotation classification tool and significantly overrepresented GO terms were extracted and presented as clusters based on interrelationships between terms calculated by the tool. P-value for enrichment of each category is given in numerals for the entire data set (all genes from all time points). Levels of probability for enrichment at discrete time points are indicated by the heat-map.

**Figure 4:**
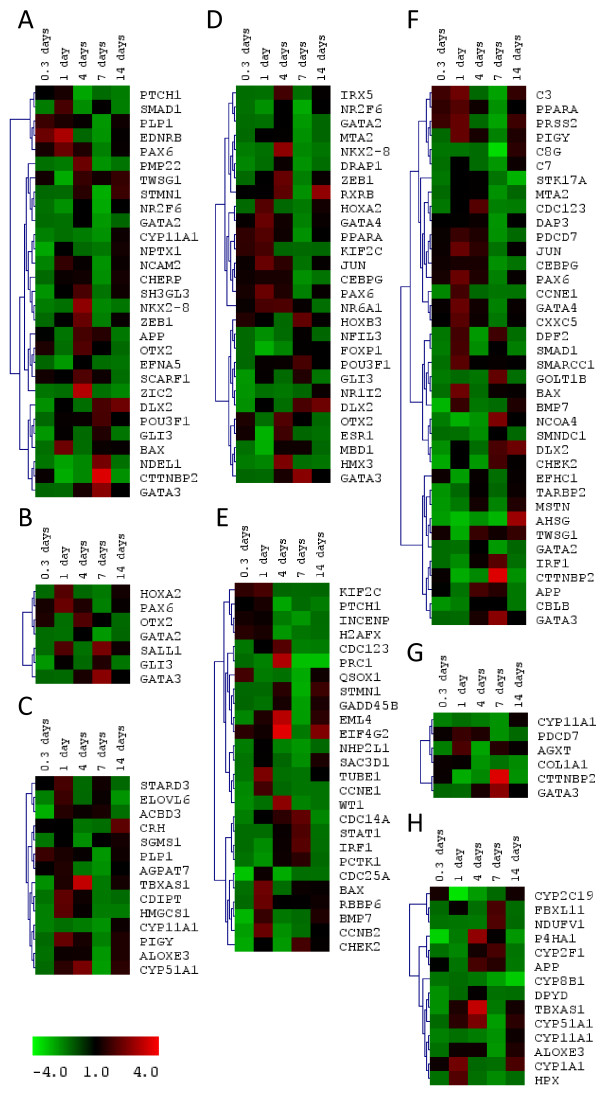
**Heatmaps of expression profiles for regulated genes, sharing enriched annotation categories, during treatment with zinc supplementation for 0.3, 1, 4, 7, and 14 days**. Gene expression is given as fold-change ranging from -4 (green) to +4 (red, see colour bar) in zinc supplementated fish relative to controls at each time point. Gene trees were generated using Spearman Rank Correlation and Average Linkage Clustering. The represented categories are (A) 'nervous system development', (B) 'cell fate commitment', (C) 'lipid biosynthetic pathway', (D) 'sequence-specific DNA-binding', (E) 'cell cycle process', (F) 'positive regulation of biological process', (G) 'response to steroid hormone stimulus', and (H) 'iron binding' as detailed in Figure 3.

To compensate for the low number of directly attributed ontological terms the zebrafish genes represented on our array were mapped across to their closest human orthologs. This processes yielded human orthologs for 70% (365) of the differentially expressed zebrafish genes, of which 356 matched to annotated DAVID gene objects. Using these orthologs we analysed the representation bias of gene ontology categories for all differentially expressed genes combined, as well as those regulated at specific time points (Figure 3). The significantly enriched (*P *< 0.01) Gene Ontology (GO) terms associated with Biological Process and Molecular Function among all genes combined were grouped into semantically similar categories and displayed together with the representation bias at each time points (Figure 3). The result confirmed all of the biased gene-function associations found using zebrafish Unigene identifiers (Additional file 1, Table S1), and also detected further enriched annotations. Annotation enrichment analysis with human orthologs reinforced the link between zinc and ontologies relating to 'gene regulation' and 'nuclear receptors', with 33 genes denoted 'transcription factor activity' (*P *= 0.003) and 28 genes as 'sequence specific DNA binding' (*P *= 7.6 × 10^-6^) (Figure 3). Intriguingly, these terms were only significantly overrepresented at days 1 and 7, suggesting the existence of two phases of the transcriptional response to zinc supplementation. The GATA family of transcription factors (TFs) were highly represented, with *gata2*, *3*, and *4 *all differentially regulated in response to zinc (Table 2). In fact, there was a significant bias among regulated genes overall for the InterPro (EMBL-EBI) term 'Zn-Finger, GATA-type' (*P *= 0.0013). TFs with links to the immune system were notable, including those involved in interleukin regulation, *zeb1 *and *nfil3*. Differential expression was observed in other systems where zinc has previously been implicated, including *pax6*, a central TF involved in development of the eye and nervous system. However, the most compelling TF association was that with 'steroid hormone receptor activity' (6 genes, *P *= 0.005), which includes *esr1 *(estrogen receptor 1) and *rxrb *(retinoid × receptor, beta). It is reassuring that there was also a coincidental bias for genes with the ontological description 'response to steroid hormone stimulus' (6 genes, *P *= 6.8 × 10^-4^) (Figures 3 and 4G), suggesting a cascade of events that initiated the alteration of TF expression.

**Table 2:** Genes with Gene Ontology annotation 'positive regulation of transcription, DNA-dependent' significantly regulated in zebrafish gill by zinc supplementation for 8 h to 14 days as indicated.

Time point	Unigene ID	Gene Description	Gene symbol	**Human homologue**^**a**^	**Reg**.^**b**^
8 h	Dr.75386	Hypothetical protein LOC100001897 (LOC100001897)	*LOC100001897*	*THRAP1*	↑
	Dr.113555	Hip2	*hip2*	*HIP2*	↓
	Dr.82162	Polymerase (RNA) II (DNA directed) polypeptide E (polr2e)	*polr2e*	*POLR2E*	↓
	Dr.99315	GLI-Kruppel family member GLI3 (gli3)	*gli3*	*GLI3*	↓
	Dr.80037	TAR (HIV) RNA binding protein 2 (tarbp2)	*tarbp2*	*TARBP2*	↓
	Dr.27060	Nuclear receptor subfamily 1, group I, member 2 (nr1i2)	*nr1i2*	*NR1I2*	↓
					
24 h	Dr.52129	Similar to Pax-family transcription factor 6.2 (LOC557147)	*LOC557147*	*PAX6*	↑
	Dr.81306	GATA-binding protein 4 (gata4)	*gata4*	*GATA4*	↑
	Dr.1064	V-jun sarcoma virus 17 oncogene homolog (avian) (jun)	*Jun*	*JUN*	↑
	Dr.76866	SWI/SNF related, matrix associated, actin dependent regulator of chromatin, subfamily c, member 1 (Smarcc1)	*smarcc1*	*SMARCC1*	↑
	Dr.82597	Homeo box A2b (hoxa2b)	*hoxa2b*	*HOXA2*	↑
	Dr.134300	Si:ch211-239e6.3 (si:ch211-239e6.3)	*ppara*	*PPARA*	↑
	Dr.82680	Sal-like 1a (Drosophila) (sall1a)	*sall1a*	*SALL1*	↑
	Dr.76928	MAD homolog 1 (Drosophila) (smad1)	*smad1*	*SMAD1*	↑
	Dr.42859	Zgc:165628 (LOC560054)	*zgc:165628*	*BCL11B*	↑
	Dr.29	Cyclin E (ccne)	*ccne*	*CCNE1*	↑
	Dr.81131	Wu:fj43h12 (wu:fj43h12)	*wu:fj43h12*	*DPF2*	↑
	Dr.119569	Estrogen receptor 1 (esr1)	*esr1*	*ESR1*	↓
	Dr.13792	Zgc:55307 (zgc:55307)	*zgc:55307*	*NCOA4*	↓
	Dr.108093	Forkhead box P1a (foxp1a)	*foxp1a*	*FOXP1*	↓
	Dr.79098	Methyl-CpG binding domain protein 1 (mbd1)	*mbd1*	*MBD1*	↓
	Dr.41031	Similar to MGC86299 protein (LOC564317)	*LOC564317*	*SMARCD2*	↓
	Dr.78256	Influenza virus NS1A binding protein a (ivns1abpa)	*ivns1abpa*	*IVNS1ABP*	↓
	Dr.76084	Si:dkey-261h15.1 (LOC569156)	*Si:dkey-261h15.1*	*HIPK3*	↓
	Dr.81949	Zgc:77060 (zgc:77060)	*zgc:77060*	*NFIL3*	↓
					
4 d	Dr.82517	Homeo box (H6 family) 3 (hmx3)	*hmx3*	*HMX3*	↑
	Dr.79439	NK2 transcription factor related, locus 9 (Drosophila) (nkx2.9)	*nkx2.9*	*NKX2-8*	↑
	Dr.132864	Wilms tumor 1a (wt1a)	*wt1a*	*WT1*	↑
	Dr.78569	Zinc finger homeobox 1 (zfhx1)	*zfhx1*	*TCF8*	↑
	Dr.351	Retinoid × receptor, beta a (rxrba)	*rxrba*	*RXRA*	↑
	Dr.334	Orthodenticle homolog 2 (otx2)	*otx2*	*OTX2*	↑
	Dr.8104	Nuclear receptor subfamily 6, group A, member 1a (nr6a1a)	*nr6a1a*	*NR6A1*	↑
	Dr.83684	Iroquois homeobox protein 5 (irx5a)	*irx5a*	*IRX5*	↑
	Dr.75342	Similar to sal-like 4 (sb:cb372)	*sall4*	*SALL4*	↓
					
7 d	Dr.1467	Paired box gene 9 (pax9)	*pax9*	*PAX9*	↑
	Dr.77524	GATA-binding protein 3 (gata3)	*gata3*	*GATA3*	↑
	Dr.84720	Zgc:110646 (zgc:110646)	*zgc:110646*	*MED4*	↑
	Dr.132502	Homeo box B3a (hoxb3a)	*hoxb3a*	*HOXB3*	↑
	Dr.119956	Zgc:103566 (zgc:103566)	*irf11*	*IRF1*	↑
	Dr.356	GATA-binding protein 2 (gata2)	*gata2*	*GATA2*	↑
	Dr.120105	Signal transduction and activation of transcription 1 (stat1)	*stat1a*	*STAT1*	↑
	Dr.2	Distal-less homeobox gene 2a (dlx2a)	*dlx2a*	*DLX2*	↑
	Dr.39148	Zgc:158606 (zgc:158606)	*zgc:158441*	*FBXL11*	↑
	Dr.75088	POU domain gene 50 (pou50)	*pou50*	*POU3F1*	↑
	Dr.80575	C20orf24 homolog (human) (c20orf24)	*c20orf24*	*C20orf24*	↓
	Dr.16181	Zgc:77260 (zgc:77260)	*zgc:77260*	*NR2F6*	↓
	Dr.78322	Similar to Epidermal Langerhans cell protein LCP1 (LOC559853)	*LOC559853*	*C14ORF92*	↓
	Dr.719	DR1-associated protein 1 (negative cofactor 2 alpha) (drap1)	*drap1*	*DRAP1*	↓
	Dr.7307	Metastasis associated 1 family, member 2 (mta2)	*mta2*	*MTA2*	↓
	Dr.21307	SET and MYND domain containing 1a (smyd1a)	*smyd1a*	*SMYD1*	↓
	Dr.15663	CCAAT/enhancer binding protein (C/EBP), gamma (cebpg)	*cebpg*	*CEBPG*	↓
					
14 d	Dr.76961	Zgc:158157 (zgc:158157)	*zgc:158157*	*DIDO1*	↑
	Dr.6047	Zgc:73112 (zgc:73112)	*zgc:73112*	*PYCR1*	↑
	Dr.133831	CTD (carboxy-terminal domain, RNA polymerase II, polypeptide A) phosphatase, subunit 1 (ctdp1)	*ctdp1*	*CTDP1*	↑
	Dr.78697	Zgc:91808 (zgc:91808)	*rfx2*	*RFX2*	↑

^a^The closest human homologues with sequence similarity >45% are listed

^b^Arrows pointing up represent up-regulation and those pointing down represent down-regulation.

It is not surprising that some differentially expressed transcriptional regulators, such as *dlx2*, *hox2a*, *smad1*, and *gli3*, are closely associated with development. In general there was a strong overrepresentation of genes linked to the 'developmental process' (96 genes at *P *= 7.4 × 10^-6^). The 'development' cluster is dominated by genes involved in development of anatomical structure (68 genes, *P *= 3.6 × 10^-5^), nervous system (29 genes, *P *= 0.0012), and cellular differentiation (53 genes, *P *= 0.0027; Figure 3). Genes associated with development started being overrepresented at day 1 and were maximally represented at day 7, while no bias for development terms was apparent on day 14. It is evident that zinc is playing a critical role at the level of 'cellular development' (39 genes, *P *= 0.0031), and may contribute to cellular differentiation through zinc regulated genes associated with 'cell fate commitment' (7 genes, *P *= 0.0074) (Figure 3).

The role of zinc as a global regulator was underlined through the myriad of terms associated with closely linked terms of 'positive regulation of biological process' (38 genes, *P *= 7.2 × 10^-4^; Figure 3) and 'positive regulation of cellular process' (33 genes, *P *= 0.0032; Figures 3 and 4F). Again the differential regulation of genes associated with immune system was notable, including genes encoding complement proteins *c8g*, *c3 *and *c7 *and the interferon regulatory factor *irf1 *(Figure 4F). The influence of zinc on the expression of these and other non-genomic regulators demonstrates the involvement of zinc on complex cell signalling systems.

Given the prevalence of global regulators affected together with those associated with functions in cellular development, it is unsurprising that the underlying cellular metabolism was impacted, with over 50% of the differentially regulated genes being associated with 'primary metabolic processes' (197 genes, *P *= 1.1 × 10^-5^). Among more interesting groups of proteins were those categorised as involved in the 'lipid biosynthetic process' (14 genes, *P *= 0.003; Figures 3, 4C) since disruption of lipid metabolism is often associated with low level metal toxicity [39,40] and these changes can be associated with those regulated by the insulin axis. It has recently been shown that zinc can substitute for insulin in cell culture with the two compounds eliciting similar growth and metabolic characteristics [41]. Indeed, four regulated genes, including the insulin receptor itself (*insra*, *ppp1r3b*, *eife1a*, *cblb*), mapped onto the KEGG 'Insulin Signalling Pathway.' The most overrepresented KEGG pathway was otherwise that for 'Pyrimidine metabolism' (9 genes, *P *= 0.0013).

The interface of zinc with the 'cell cycle process' (26 genes, *P *= 0.0055; Figures 3 and 4E) was illustrated through the zinc based regulation of molecules involved in apoptosis (*bax*), cell division (*cdc25a, cdc14a, cdc123, ccnb2*), DNA damage (*gadd45b*), and those like *chek2 *implicated in all of these processes. The response involving cell cycle was maximally significant at day 4 but remained significant at day 7 (Figure 3), correlating with the transition from increased zinc influx across the gills to a return to steady state flux levels (Figure 1B).

The only gene ontology term associated with direct metal binding which passed our significance criteria was 'iron binding' (14 genes, *P *= 0.0073; Figure 3), which included several heme containing proteins, and in particular a selection of cytochrome P450 s (*cyp2c19, cyp2f1, cyp511a, cyp8b1, cyp11a1*; Figure 4H). Analysis of semantic enrichment from other database such as Swissprot PIR keywords and Interpro did provide highly significant enrichment of terms such as 'metal-binding' (67 genes, *P *= 0.0049), 'metalloprotein' (12 genes, *P *= 2 × 10^-5^), 'zinc finger, NHR/GATA-type' (6 genes, *P *= 8.8 × 10^-4^), 'iron' (13 genes, *P *= 0.0023), and 'cytochrome P450' (7 genes, *P *= 0.0019), whilst weaker associations with terms included 'calcium-binding EF-hand' (11 genes, *P *= 0.015) and 'calcium' (22 genes, *P *= 0.029). The cellular exporter for ferrous iron, *slc40a1 *(*ferroportin1*), was up-regulated 2.7-fold by zinc supplementation at the 1 day time point, likely in compensation of compromised iron uptake. Only one zinc transporter, *slc39a13 *(*zip13*), was detected to respond to zinc supplementation; it was down-regulated by 1.9-fold at days 1 and 4, followed by a gradual return to the control level.

The Biological Process term 'detection of abiotic stimulus' was significantly overrepresented when genes whose expression had been significantly impacted by zinc independent of time were considered (5 genes, *P *= 0.0092), but did not seem to gravitate towards any particular time-point (Figures 3). An interesting facet of this group with possible significance is that four of the five genes in this cluster, *gnat1, gnat2, rgr*, and *pdc*, have well-described functions in the outer retina, one of the most zinc-rich tissues of the vertebrate body [42].

### Analysis of transcription factor binding sites (TFBS) in the 5' region of regulated genes

**Figure 5:**
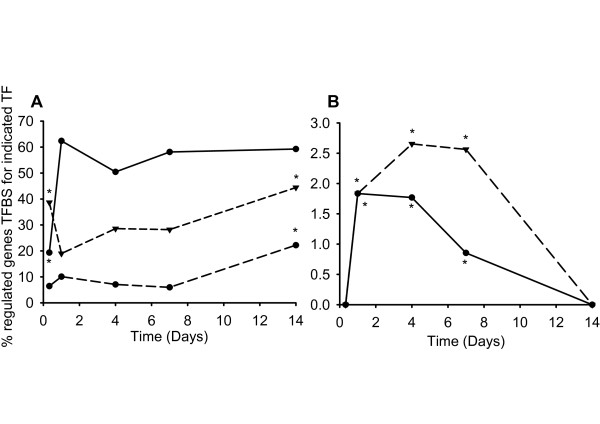
**Frequency of transcription factor binding sites (TFBS), to regulated transcription factors (TFs)**. The TFBS were located within 2000 bp or 500 bp DNA sequence upstream of the ATG of genes regulated by zinc supplementation at different time-points. Panel A represents the % genes regulated at each time point contain TFBS for ppara (circles and solid line), nra61a (gcnf) (circle and dashed line) and mtf1 (triangle and dashed line). Panel B shows the % regulated genes containing stat1 (circles and solid line) and gata3 (triangle and dashed line) binding sites. A significant difference in frequency of occurrence of the respective TFBS, relative to that in a cohort of 198 stable (< 1.8-fold change) genes is indicated by an asterisk (*P *< 0.05, *z*-test).

It was considered that many of the observed changes in gene expression were not caused by zinc itself, but rather by a transcriptional cascade involving a limited number of key regulators. A large proportion of the genes that were regulated correspond to genes involved in regulation of transcription (Table 2) and it is reasonable to assume that changes in expression of TFs would have resulted in further downstream effects on transcription of other genes. To investigate the cascade effects that might result from changes in expression of these specific TFs, we extracted the upstream DNA sequence of all regulated genes and searched for the occurrences of their cognate binding motifs (TFBS) as defined by the Transfac database [43]. Of the TFBS for the transcriptionally regulated TFs mapped to entries in the Transfac (2005) database (using gene names and synonyms), *ppara*, *nr6a1a*, *stat1*, and *gata3 *showed statistically significant difference in frequency of occurrence among regulated genes relative to that of non-regulated control genes (Figure 5). Interestingly, the number of genes with binding sites for Ppara was markedly underrepresented at the 8 hour time-point, compared with the stably expressed gene cohort as well as the genes regulated at all subsequent time-points. Although the expression of *ppara *was only defined as increased (FC > 1.8, FDR = 0.1) at the day 1 sampling, it did show a 1.6-fold higher expression in zinc supplemented fish compared with the control at the 8 hour time-point. There was an overrepresentation of TFBS for Stat1 and Gata3 among genes regulated on days 1 to 7 of the experiment, although the genes for these TFs were significantly up-regulated on only day 7 (Figure 5). However, GATA factors have the same consensus binding site and *gata4 *showed increased expression on day 1.

Although *mtf1 *mRNA expression did not change under zinc supplementation in this experiment, Mtf1 is a well-recognised zinc sensor [44]. Hence, binding motifs for *mtf1 *(MREs) were also included in the analysis. The frequency of MREs among regulated genes was statistically higher than that in non-regulated genes at the 8 hour and day 14 sampling points. Among the genes containing MREs and displaying altered expression in response to zinc were *mt2*, *znt1*, and *zip10*, all known to be regulated by *mtf1 *in zebrafish [10,22,44].

### Pathway analysis of the transcriptional response to zinc supplementation

**Figure 6:**
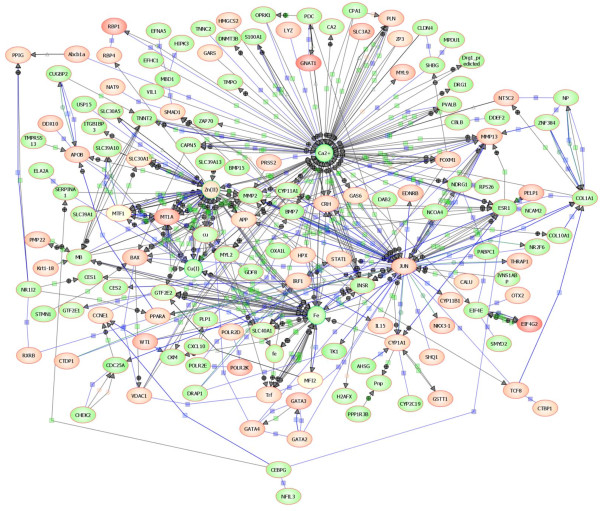
**Molecular interactions between zinc, copper, iron, calcium and proteins encoded by transcripts changed by zinc supplementation**. A Direct Interaction Network was automatically generated based on curated interactions contained within the proprietary PathwayArchitect database. An interactive version of this network is also provided (Additional file 2, Figure S1). Ovals represent proteins and the circles symbolize metal ions. Objects are coloured by their abundance in zebrafish at the time-point they were significantly different from the control is a scale from -4 fold (dark green) to +4 fold (dark red). Where significant differences were found at more than one time-point, the colour overlay shows expression at the first instance. Dark blue squares denote 'binding', and light blue squares 'expression'; green squares stand for 'regulation', green diamonds for 'metabolism', and green circles for 'promoter binding'. Arrow heads indicate directionality of the interaction where annotated.

The molecular interactions between zinc and proteins encoded by regulated transcripts were explored and visualised using the PathwayArchitecht^® ^software. A continuous 'Direct Interaction Network' was generated consisting of 534 nodes and 503 interactions (Figure 6). An interactive version of this network is presented (Additional file 2, Figure S1) where nodes and edges can be individually interrogated. A remarkable number of the proteins corresponding to regulated genes interact with zinc, copper, iron, and calcium, homeostasis of which are all affected by zinc supplementation. By far the highest number of interactions with a protein was found for connections with Jun, which was indicated to be activated by zinc and connected to 71 nodes. Incidentally, the network also implicated Jun to be a transcriptional regulator of *thrp1*, the only transcription factor up-regulated at 8 hours into the zinc supplementation regime.

## Discussion

The present study describes for the first time the cascade of transcriptional responses to zinc supplementation in an animal zinc uptake epithelium. There was a remarkable variation in gene expression over time, with the number of up-regulated genes dominating at days 1 and 4, and down-regulation being predominant at 8 hours and 7 days. There was a steady increase in the number of differentially expressed genes up to day 7, and after 14 days of treatment few genes remained regulated. A similar pattern was observed in gills of zebrafish treated with zinc-depleted water and feed [37]. The conclusion from this global analysis may be that the transcriptional adjustments in the gill required for acclimation to a change in zinc availability culminates within a week and acclimation is established by two weeks. This is in line with studies on rainbow trout (*Oncorhynchus mykiss*) showing physiological acclimation to zinc supplementation within a two-week period [8]. Two previous studies on cultured human cells have investigated the effect of time on transcriptomic responses to altered zinc regimes [30,32]. Although these studies considered much shorter time-scales (up to 24 hours using cell culture) than in the present study, they do confirm that there are large differences in gene expression with time. Taken together these new findings have quite profound implications on how microarray experiments concentrating on a single time-point should be interpreted, and emphasise the need for time-course studies.

Although there was little overlap between genes regulated at adjacent time-points, they tended to belong to the same, or similar ontologies. In addition, several of the enriched functional categories sorting under 'development', 'metabolism', and 'gene expression' were overrepresented, not only among genes regulated by zinc supplementation, but also during zinc depletion [37]. These functional categories represented a catalogue of the diverse roles played by zinc in biology, and tended to focus on embryonic development and regulation of cellular processes, both at genomic and non-genomic levels. This is fully in keeping with the view of zinc as a signalling substance with many essential functions, particularly during organism development, and the present findings fill gaps in the networks of such interactions [25,45-48].

The GO term 'developmental process' was one of the most significantly enriched annotations (*P *= 7.4 × 10^-6^) among genes regulated in gills of zinc supplemented zebrafish (see Figure 3). This indicates a particular importance of zinc dependent gene expression during development. Mtf1 is believed to be the principal mediator of direct transcriptional responses to zinc [44]. In mouse Mtf1 is essential for embryonic development, but not post-parturition, where it is of primary importance to metal tolerance [48]. Accordingly, we previously found that almost half (44%) of a set of 59 candidate Mtf1 targets in zebrafish (with DAVID identifiers) were annotated with the GO term "developmental process", compared with 23% in the whole genome. The degree of enrichment was much smaller in the present study (27% of regulated genes with DAVID identifiers), which would be expected as most of these genes were likely to not be directly regulated by Mtf1. Analysis of the 5' flanking regions of regulated genes showed a statistically significant bias for the Mtf1 cognate binding motif (MRE) at the 8 hour and 14 day time-points, but the functionality of these MREs was not investigated.

Developmental genes tended to have the highest bias towards day 7, possibly indicating a physical remodelling of the gill as a part of the acclimation process to the new zinc level. The fish gill has a phenomenal morphological plasticity and dramatic alterations in cell-types can occur to allow the fish to cope with changes in water chemistry [49]. In support of this idea 31 genes that were regulated on day 7 carried the annotation "developmental process" (32% of the 98 genes recognised by the 'DAVID knowledgebase'), and 18 of these are involved in cell differentiation. Several of the developmental genes that were regulated by zinc supplementation, map onto the KEGG hedgehog signalling pathway. These include *gli3 *(down-regulated 2.0-fold at 8 hours), *bmp7b *(down-regulated 2.6-fold at 8 h), *zic2a *(up-regulated 3.1-fold at day 4), and *ptc1 *(down-regulated 2.3-fold at day 4). In vertebrates the hedgehog family of proteins has a variety of functions during development, but also regulates stem cells involved in maintenance and regeneration of post-embryonic tissues [50]. In zebrafish sonic hedgehog (Shh) binds to its receptor, Ptc1, located at the plasma membrane. This relieves the repression of another plasma membrane protein, Smo, which in turns activates the Gli family of transcription factors. Zebrafish Gli3 mainly functions as a transcriptional repressor suppressed by Shh signalling. Therefore, down-regulation of *gli3 *(as observed in the present study) promotes Shh signalling [50]. *Bmp7 *was one of the regulated downstream targets of the hedgehog pathway in the present study. This may be of special interest because Bmp7 is involved in the development of ion-transporting ionocytes in the zebrafish gill [49], and these cells are responsible for zinc uptake in fish [6]. Two other downstream targets of Shh signalling [51] were also found to be regulated by zinc supplementation, *pax6 *(up-regulated 2.2-fold at day 1), and *dlx2a *(up-regulated 1.9-fold at day 7).

There was only a single zinc transporter, *slc39a13 *(*zip13*), which passed the data filters and was designated as significantly regulated. In mammals Zip13 is located in the Golgi apparatus and is required for connective tissue development through its involvement in the Bone Morphogenic Protein (Bmp) and Transforming Growth Factor Beta (Tgfβ) signalling pathways [45]. Like the hedgehog pathway, BMP-signalling contributes to several developmental processes, but are also important for stem cell fate and proliferation in post-embryonic tissues [52]. In the present study we found that *slc39a13 *expression was down-regulated by zinc supplementation, and related to this we also found altered expression of *bmp7b *(2.6-fold decrease at 8 hours), *bmp3 *(zgc:153939; 2-fold increase at day 7), and *bmp13 *(2.3-fold decrease at day 7). Downstream targets of BMP signalling include *smad1 *(2.8-fold increase at day 1), *gata4 *(2.2-fold increase at day 1), and matrix metalloproteases, Mmp (Mmp2: 2.4-fold decrease at day 4; Mmp13: 2.2-fold increase at day 4). Furthermore, *dlx5 *and *dlx6*, which are part of the same pathway, were regulated in zebrafish gill by zinc depletion [37]. Dlx5 has been shown to induce expression of *Runx2 *in chicken [53], and Runx2, which is important for osteogenic differentiation, was recently found to be downregulated during zinc deficiency [54], and abnormally accumulated in osteoblasts from *Slc39a13 *null mice [45]. These results provide compelling evidence for the involvement of Slc39a13 and zinc signalling in the BMP/TGF-beta pathways, and indicate that this connection is conserved in the vertebrate lineage. Taken together, the present study suggests that homeostatic regulation of zinc uptake at the gill may involve reprogramming of epithelial stem cell fate and restructuring of the gill surface. Specifically, this appears to involve hedgehog and BMP signalling. The present study is therefore delivering unique insight to the molecular mechanisms that might be involved in this process, although more focussed studies will be required to determine the exact signalling pathways and outcomes.

Other zinc transporters did not appear in the gene lists of significantly regulated transcripts, either because their expression was below the detection limits of the array or they did not pass the stringent statistical criteria associated with FDR associated with parallel analysis of >16 K genes. However, we have recently presented qPCR analysis of gills from the same fish showing that zinc supplementation results in down-regulation of the zinc importers *slc30a5 *(*znt5*) and *slc39a10 *(*zip10*), and up-regulation of the basolateral zinc exporter *slc30a1 *(*znt1*) [22]. We further showed that induction of *znt1*, as well as repression of *zip10*, was mediated by the intracellular zinc sensor Mtf1 [22,44]. However, *mtf1 *appeared not to be responsible for changes in *znt5 *expression, and an as yet unidentified transcription factor is thought to be responsible for zinc-mediated down-regulation of *ZnT5 *in humans [55].

Zinc supplementation caused no visible signs of toxicity to zebrafish. However, the increased zinc content of feed and water did have negative effects on copper status, and caused a numerical, but non-significant (*P *= 0.054), decrease in whole body iron content. These are well-known effects of high dietary zinc supplementation [56-59]. There was also a tendency for whole-body calcium content to be reduced and it has likewise been shown previously that zinc is a potent inhibitor of calcium uptake [9]. Although interactions between zinc, copper, iron, and calcium uptake may occur by competition for transporters, our network analysis of regulated genes (Figure 6) clearly show the complex interplay at different levels between zinc and homeostasis of these essential metal ions. This interplay was recently highlighted in a microarray study on cultured cells, in which several copper and iron regulatory genes were regulated by zinc supplementation [30]. In the present study we observed an up-regulation of *slc40a1 *(ferroportin1), which mediates transport of iron from the enterocytes to the blood circulation. Similarly, Kelleher et al. [59] found that in the rat intestine negative effects of zinc supplementation on iron absorption were associated with increased ferroportin-1 expression, retention of iron in the intestinal tissue, and decreased hephaestin levels. Iron binding was also a Gene Ontology Molecular Function that was enriched among regulated genes in the present study (Figure 4). This might be associated with the trend for zinc-induced decrease in whole body iron content, although other explanations are possible.

It is becoming increasingly apparent that labile intracellular Zn^2+ ^plays roles in non-genomic cell signalling [5]. For example, an increase in labile Zn^2+ ^has been shown to activate the ERK1/2 pathway by inhibition of MAPK phosphatases [44]. An early increase in labile Zn^2+ ^in the gill cells as a result of the sudden increase in waterborne zinc at the beginning of the experiment may have led to MAPK-mediated phosphorylation and activation of Jun, which was one of the nodes with the most edges in our Direct Interaction Network (see Figure 6). Expression of *jun *was also increased on day 1 and this may have reinforced the influence of Jun on early gene expression. It has been shown previously that zinc induces *jun *expression in chicken BM2 cells and in human HPR-1 cells [32,60]. Agonists of PPARA (peroxisome proliferator-activated receptor alpha) have also been found to be inducers of *Jun *expression [61], and in the present study *ppara *was up-regulated on day 1, whilst one of its heterodimer partners, *rxrba*, showed increased expression on day 4 (see Table 2; Figure 4D).

Ppara is a nuclear receptor which, along with other members of the PPAR family, is involved in control of lipid metabolism, cell proliferation, cell differentiation, and immune and inflammatory responses. In our study *ppara *expression in gills was up-regulated by zinc supplementation on day 1, but there was already a non-significant 1.6-fold increase in *ppara *mRNA at 8 hours after the start of zinc supplementation. It has been shown previously that the DNA-binding activity of Ppara is regulated by zinc [62,63] and this may have been one of the very first ripples in the transcriptional cascade. The increase in *ppara *expression in our experiment may result from the zinc induced increased Ppara activity because its transcription can be autoregulated [64]. Interestingly, the TFBS for Ppara was significantly underrepresented among genes regulated at the 8 hour sampling point, but was present in about 60% of all regulated genes thereafter. Our data show that at least 22 genes involved in lipid metabolism were regulated by zinc either directly or indirectly, and GO terms, such as "cellular lipid metabolic process" (20 genes, *P *= 0.034) and lipid "biosynthetic process" (14 genes, *P *= 0.003), were overrepresented among regulated genes. Zinc supplementation resulted in up-regulation of *apob*, *slc27a2*, *stard3*, and *hmgcs1*. ApoB (Apolipoprotein B) is the main apolipoprotein of chylomicrons and low density lipoproteins (LDL). Slc27a2 can convert free long-chain fatty acids into fatty acyl-CoA esters and play a key role in lipid biosynthesis and fatty acid degradation. Stard3 is a sterol transporter involved in cholesterol homeostasis, which is regulated, at least in part, by sterol regulatory element (SRE)-binding proteins [65]. Hmgcs1 catalyses the acetyl-CoA to HMG-CoA, which is further involved in ketone metabolism. Hmgcs1 was also down-regulated in zebrafish gill by zinc depletion of water and feed [37]. The interaction between zinc supplementation and lipid metabolism is well established and has been suggested as a major negative side effect of dietary zinc supplementation, especially in an aging population [66], since it results in an adverse affect on HDL cholesterol [67]. In the present study we show that increased zinc accumulation is associated with profound changes in the expression of genes involved in lipid metabolism, helping to explain the molecular background to the known effect of zinc on lipid profiles. Our results echoed the findings in zinc-deficient rats, which showed profound changes in genes involved in hepatic lipid metabolisms, including *ppar *genes, fatty acid transporter genes, genes involved in Acyl-CoA metabolism, and several genes of the cytochrome P450 family [33]. It is intriguing that activation of Ppara has similar effects on lipid metabolism [68,69], and therefore we may postulate this TF provides the mechanistic link between zinc and lipid metabolism.

It has been noted that zinc deficiency is associated with atherosclerosis and development of ischemic heart diseases, and zinc supplementation is used to help treat angina pectoris [70]. PPARs might play a major role in these processes, especially PPARA [69]. PPARA inhibits inflammatory reactions, one of the key factors involved in atherosclerosis, by stopping NFκB signalling, positively regulating IκBα, inhibiting JUN function, negatively regulating COX2, etc [71].

## Conclusion

In conclusion, the transcriptional response to zinc supplementation in the zebrafish gill epithelium goes through several phases that probably involve both genomic and non-genomic signalling. Our attempts to reverse-engineer the very complex time-dependent progression of regulated transcripts resulted in the conclusion that the early phase is characterised by regulation of genes that may be responsive to a few key transcription factors, such as Mtf1, Jun, Stat1, Ppara, and Gata3. These would have been responsible for the expression of other transcription factors as well as the initiation of compensatory measures, such as regulation of zinc transporters as shown previously [37], and, interestingly, remodelling of the gill epithelium through stem-cell differentiation. This reprogramming of stem cell fate likely involved hedgehog and bone morphogenic protein signalling. The crescendo of the transcriptional cascade culminates after a week of zinc supplementation, which coincides with maximum repression of zinc importer (*zip10*, *znt5*) expression [37]. The subsequent damping down of the transcriptional wave suggests that the acclimation process was concluded by the end of the two week experiment.

## Methods

### Animals and zinc supplementation

The zebrafish husbandry and treatment were described previously [10,37]. Juvenile zebrafish, *Danio rerio *(0.44 ± 0.06 g), were divided into three experimental groups with each group held in four identical tanks, and supplied with a continuous flow of aerated reconstituted reverse osmosis water nominally containing 0.25 μM Zn^2+ ^at 26-28°C and fed with a purified mash diet (Fish Nutrition Unit, University of Plymouth, UK) containing an analysed zinc concentration of 233 mg kg^-1 ^(3.56 mmol kg^-1^). After one-week acclimation to laboratory conditions, a nominal zinc concentration of 5 μM (330 μg L^-1^) was introduced for a group of four tanks through a dosing system (zinc supplemented group). The feed for this fish group was replaced with that containing an analysed zinc concentration of 2023 mg kg^-1 ^(30.9 mmol kg^-1^). A group of fish in four separate tanks was kept at the same levels of zinc in water and feed as before (water: 0.25 μM, feed: 233 mg kg^-1^). The two water and feed compositions were otherwise identical. Feed ration was kept at 4% of body mass per day for both groups. The zinc concentrations in the water from all tanks were monitored daily using Inductively Coupled Plasma Mass Spectroscopy (PerkinElmer Elan DRC ICP-MS) on HNO_3 _acidified samples. The measured water zinc concentrations in control and excess groups were 0.25 ± 0.09 μM and 4.02 ± 1.2 μM (mean ± SD, *N *= 14), respectively. The experiment continued for 14 days with no mortalities or necropsies observed.

### Whole body nutritional composition

At the end of two weeks nine fish from each group were killed by overdose of benzocaine (Sigma, USA) and analysed for whole body electrolyte and trace elements as described previously [37,72]. Nutritional parameters for moisture, protein, ash, and lipid were also determined, in triplicate where possible [37,73].

### Zinc influx assay

The unidirectional influx of Zn^2+ ^was measured for each group of fish after 8 hours, 1 day, 4 days, 7 days, and 14 days after treatment at the nominal waterborne zinc concentration of the zinc supplemented group (5 μM; 327 μg L^-1^). Zn^2+ ^influx was analysed based on ^65^Zn uptake across the gill over a 3 hour period as described previously [37,74]. Briefly, the fish were placed in polyethylene bags, each filled with 2 L of the reconstituted water described above with 5 μM Zn^2+ ^containing ^65^Zn^2+^. At the end of the flux the fish were killed, weighed, and counted for radioactivity. Influx of Zn^2+ ^was calculated from accumulation of radioisotope (cts min^-1^) divided by the specific activity of ^65^Zn (cts min^-1 ^pmol^-1^), the weight of the fish (g), and the flux period (h). Data are shown as means ± SD (*N *= 8) and the significance of differences was determined with two-tailed unpaired Student's T-test (*P *< 0.05).

### Microarray analysis

Two-colour microarray analysis was carried out on gills from five fish per group and time point (*N *= 4 or 5, one control sample from day 4 was removed because of failure to pass quality control) using common reference design exactly as detailed in Zheng et al. [37]. Thus, a total of 49 microarrays were hybridised and analysed across the two groups and five time-points. Briefly, total RNA was extracted from dissected gill samples and the amino-allyl indirect labelling method was used to obtain Cy3 (reference) or Cy5 (samples)-labelled cDNA. Targets were hybridised onto in-house spotted 16.5 K zebrafish microarrays featuring 15,806 LEADS™ clusters plus 171 controls (Compugen and Sigma Genesys), as well as 331 customised oligonucleotides and 23 controls from Amersham Lucidea Universal Scorecard™. Hybridised microarrays were scanned by ScanArray (Perkin Elmer), and spot intensities in Cy3 and Cy5 channels quantified using Bluefuse software (BlueGnome). Lowess normalisation was applied and the probe intensities with low confidence (*P *< 0.05) filtered out and the normalised data then uploaded onto GeneSpring 7.3X software (Agilent Technologies). The dataset is available from Gene Expression Omnibus (GEO) under accession numbers GSE21907 and GSE21914. Profiles generated from five fish gills of each group and time point were regarded as biological replicates. Only genes with expression data from at least three replicate samples were considered expressed and used for subsequent differential expression analysis. The differential expression between zinc supplementation and control groups at each time point was determined by combination of a *t*-test with Benjamini-Hochberg multiple testing correction (false discovery rate, FDR) and a fold change (FC). The genes were deemed as differentially expressed with FC greater than 1.8 and an adjusted P-value less than 0.1, controlling for 10% FDR.

### Gene annotation and functional analysis

Differentially expressed genes were subjected to annotation enrichment analysis using the online functional annotation tool DAVID (Database for Annotation, Visualisation and Integrated Discovery, http://david.abcc.ncifcrf.gov/) [38]. Zebrafish gene identifiers as well as the inferred human orthologs were used for this analysis to improve richness of the output [37]. Significance was calculated by a modified Fisher's Exact test (*P *< 0.05).

The interactions between regulated genes and affected minerals were evaluated by network analysis using the PathwayArchitecht^® ^software (Stratagene). The closest human homolog to each of the genes which were designated as significantly regulated (FC > 1.8 and FDR = 0.1) at one or more time points were imported into the software. The zinc transporters, *slc39a *(*zip*) *1*, and *10*, and *slc30a *(*znt1*) and *5*, previously shown to be regulated by zinc using qPCR [10], were added to the list of objects. Because of its established role in zinc regulation metal-responsive transcription factor-1 (Mtf1) was also added. A Direct Interaction Network was generated by adding interactions (edges) between the entered components (nodes) from the PathwayArchitecht^® ^database.

### *In silico *analysis of regulated genes with binding sites for regulated transcription factors

Upstream DNA sequences (500 bp and 2000 bp) of genes differentially regulated at each sampling time were obtained from Ensembl (Zv5) and analysed for enrichment of binding motifs for transcription factors (TF), defined by Transfac database [43], corresponding to the regulated TF (See Table 2). Binding motifs for metal-responsive transcription factor-1 (Mtf1) were also included because of its well-known role in zinc regulation, although the *mtf1 *gene was not itself transcriptionally regulated by zinc in the present study. The number of analysed sequences from the 8 hours, 1, 4, 7, and 14 day time points were 31, 109, 113, 117, and 27, respectively. The frequencies of transcription factor binding sites (TFBS) were compared with those in upstream sequences for a cohort of 198 randomly selected genes not regulated at any sampling point by zinc supplementation or depletion. The differences between the two frequencies were statistically assessed using *z*-test. Other details are as in Zheng et al. [37].

### Real-time quantitative polymerase chain reaction validation

Microarray analysis of differential gene expression was validated by real-time quantitative polymerase chain reaction (qPCR) analysis for 10 genes: *mt2*, *slc30a1 *(*znt1*), *ppara*, *foxl1*, *hmgcs1*, *apob*, *cyp11a1*, *dlx5*, *dlx2*, and *rfx2*. Nine gill samples from each condition and sampling point, including the five replicates analysed by microarray, were analysed by qPCR. qPCR was performed on an ABI prism 7000 sequence detection system (ABI) using a SYBR green protocol. Methods, primers, and other details from this validation have been published [37]. Briefly, total RNA (2 mg) was reverse transcribed and cDNA subjected to qPCR analysis under cycling conditions as follows: 5 minutes of denaturation at 95°C and then 40 cycles of 95°C for 30 s, 60°C for 1 minute. The standard curve was generated for each gene and the relative copy number was calculated using C_t _value. The 18 s rRNA gene was used as a reference gene. The statistical significance of differences between experimental groups was determined with two-tailed unpaired Student's *t*-test (*P *< 0.05).

## List of Abbreviations

MT: Metallothionein; MTF1: metal-responsive transcription factor 1; TF: transcription factor; TFBS: transcription factor binding sites; MRE: metal response element; FDR: False Discovery Rate.

## Authors' contributions

DZ was responsible for the majority of the wet laboratory experimentation presented including both the microarray and physiological components. RDH designed the experimental zebrafish diets and performed whole body lipid, carbohydrate, protein, and elemental analyses. GPF deigned a suite of custom array reporters representing the complete family of zebrafish zinc transporters. PK performed the annotation of the array reporters and PC executed the informatic TFBS quires. CH performed the interpretation of the TFBS query data including the reverse engineering of TF pathways and bioinformatics underpinning the network analysis. DZ, PK and CH were responsible for the statistical analysis of the array data and its subsequent functional interpretation. PK, DZ and CH participated in conception and design and interpretation of the study. PK and CH drafted the manuscript and DZ and RH contributed to the iterative refinement of the article. All authors have read and approved the submitted version.

## Supplementary Material

Additional file 1 **Genes displaying significantly altered expression under zinc supplementation**. An annotated list of genes called significantly regulated with more than 1.8-fold change, compared to control, and FDR = 0.1 (*N *= 4 or 5) at one or more time points between 8 hours and 14 days. The total number of samples analysed by microarray was 50. Annotation provided was determined by mapping the reporter sequences using megablast allowing 2 bp mismatches to Ensembl (Zv5), Refseq and Genebank. Genebank matches were used to retrieve the relevant Unigene Cluster ID and gene names (as provide in the table) and used to determine the associated human orthologues for which the unigene ID and gene symbol is also provided. The fold change in expression at each time point is also provided expressed relative to time match control. Click here for file

Additional file 2 **Interactive Direct Interaction Network representing the molecular interactions between zinc, copper, iron, calcium and proteins encoded by transcripts changed by zinc supplementation**. Mini web-site containing index.html and hyperlinked pages in subdirectory describing a Direct Interaction Network automatically generated based on curated interactions contained within the proprietary PathwayArchitect database. Ovals represent proteins and the circles symbolize metal ions. Objects are coloured by their abundance in zebrafish at the time-point they were significantly different from the control is a scale from -4 fold (dark green) to +4 fold (dark red). Where significant differences were found at more than one time-point, the colour overlay shows expression at the first instance. Dark blue squares denote 'binding', and light blue squares 'expression'; green squares stand for 'regulation', green diamonds for 'metabolism', and green circles for 'promoter binding'. Arrow heads indicate directionality of the interaction where annotated. All nodes and edges can be further interrogated by selecting the relative area of the image. Click here for file

## References

[B1] Williams RJP Chemical selection of elements by cells Coordination Chemistry Reviews 2001 216-217 583 595 10.1016/S0010-8545(00)00398-2

[B2] Eisler R Zinc hazards to fish, wildlife and invertebrates: a synoptic review Book Zinc hazards to fish, wildlife and invertebrates: a synoptic review (Editor ed.^eds.), vol. Biological Report 1993 City: Fish and Wildlife Service 126

[B3] Andreini C Banci L Bertini I Rosato A Counting the Zinc-Proteins Encoded in the Human Genome Journal of Proteome Research 2005 5 196 201 10.1021/pr050361j 16396512

[B4] Krężel A Maret W Thionein/metallothionein control Zn(II) availability and the activity of enzymes Journal of Biological Inorganic Chemistry 2008 13 401 409 10.1007/s00775-007-0330-y 18074158

[B5] Hogstrand C Kille P Nicholson RI Taylor KM Zinc transporters and cancer: a potential role for ZIP7 as a hub for tyrosine kinase activation Trends in Molecular Medicine 2009 15 101 111 10.1016/j.molmed.2009.01.004 19246244

[B6] Hogstrand C Verbost PM Bonga SEW Wood CM Mechanisms of zinc uptake in gills of freshwater rainbow trout: Interplay with calcium transport American Journal of Physiology-Regulatory Integrative and Comparative Physiology 1996 39 R1141 R1147 10.1152/ajpregu.1996.270.5.R1141 8928918

[B7] Comber SDW Merrington G Sturdy L Delbeke K van Assche F Copper and zinc water quality standards under the EU Water Framework Directive: The use of a tiered approach to estimate the levels of failure Science of the Total Environment 2008 403 12 22 10.1016/j.scitotenv.2008.05.017 18599110

[B8] Hogstrand C Reid SD Wood CM Ca^2+ ^versus Zn^2+ ^transport in the gills of fresh-water rainbow-trout and the cost of adapation to waterborne Zn^2+^ Journal of Experimental Biology 1995 198 337 348 9317921 10.1242/jeb.198.2.337

[B9] Hogstrand C Wilson RW Polgar D Wood CM Effects of zinc on the kinetics of branchial calcium-uptake in fresh-water rainbow-trout during adapation to waterbourne zinc Journal of Experimental Biology 1994 186 55 73 7525831 10.1242/jeb.186.1.55

[B10] Zheng DL Feeney GP Kille P Hogstrand C Regulation of ZIP and ZnT zinc transporters in zebrafish gill: zinc repression of ZIP10 transcription by an intronic MRE cluster Physiological Genomics 2008 34 205 214 10.1152/physiolgenomics.90206.2008 18477665 PMC2494845

[B11] Feeney GP Zheng DL Kille P Hogstrand C The phylogeny of teleost ZIP and ZnT zinc transporters and their tissue specific expression and response to zinc in zebrafish Biochimica Et Biophysica Acta-Gene Structure and Expression 2005 1732 88 95 10.1016/j.bbaexp.2005.12.002 16500426

[B12] Palmiter RD Findley SD Cloning and functional-charaterisation of a mammalian zinc transporter that confers resistnace to zinc Embo Journal 1995 14 639 649 7882967 10.1002/j.1460-2075.1995.tb07042.x PMC398127

[B13] Balesaria S Hogstrand C Identification, cloning and characterization of a plasma membrane zinc efflux transporter, TrZnT-1, from fugu pufferfish (Takifuglu ruhripes) Biochemical Journal 2006 394 485 493 10.1042/BJ20050627 16212555 PMC1408679

[B14] Valentine RA Jackson KA Christie GR Mathers JC Taylor PM Ford D ZnT5 variant B is a bidirectional zinc transporter and mediates zinc uptake in human intestinal Caco-2 cells The Journal of Biological Chemistry 2007 282 14389 14393 10.1074/jbc.M701752200 17355957

[B15] Jackson KA Helston RM McKay JA O'Neill ED Mathers JC Ford D Splice variants of the human zinc transporter ZnT5 (SLC30A5) are differentially localized and regulated by zinc through transcription and mRNA stability The Journal of Biological Chemistry 2007 282 10423 10431 10.1074/jbc.M610535200 17234632

[B16] Maret W Molecular aspects of human cellular zinc homeostasis: redox control of zinc potentials and zinc signals Biometals 2009 22 149 157 10.1007/s10534-008-9186-z 19130267

[B17] Kimura T Itoh N Function of metallothionein in gene expression and signal transduction: Newly found protective role of metallothionein Journal of Health Science 2008 54 251 260 10.1248/jhs.54.251

[B18] Cho YS Lee SY Kim K-Y Nam YK Two metallothionein genes from mud loach Misgurnus mizolepis (Teleostei; Cypriniformes): Gene structure, genomic organization, and mRNA expression analysis Comparative Biochemistry and Physiology Part B: Biochemistry and Molecular Biology 2009 153 317 326 10.1016/j.cbpb.2009.04.002 19383548

[B19] Hiu-Mei Yan C Chan KM Characterization of zebrafish metallothionein gene promoter in a zebrafish caudal fin cell-line, SJD.1 Marine Environmental Research 2002 54 335 339 10.1016/S0141-1136(02)00141-1 12408584

[B20] Gonzalez P Baudrimont M Boudou A Bourdineaud J-P Comparative Effects of Direct Cadmium Contamination on Gene Expression in Gills, Liver, Skeletal Muscles and Brain of the Zebrafish (*Danio rerio*) Biometals 2006 19 225 235 10.1007/s10534-005-5670-x 16799861

[B21] Laity JH Andrews GK Understanding the mechanisms of zinc-sensing by metal-response element binding transcription factor-1 (MTF-1) Archives of Biochemistry and Biophysics 2007 463 201 210 10.1016/j.abb.2007.03.019 17462582

[B22] Hogstrand C Zheng D Feeney G Cunningham P Kille P Zinc-controlled gene expression by metal-regulatory transcription factor 1 (MTF1) in a model vertebrate, the zebrafish Biochemical Society Transactions 2008 36 1252 1257 10.1042/BST0361252 19021535

[B23] Wimmer U Wang Y Georgiev O Schaffner W Two major branches of anti-cadmium defense in the mouse: MTF-1/metallothioneins and glutathione Nucleic Acids Research 2005 33 5715 5727 10.1093/nar/gki881 16221973 PMC1253828

[B24] Haase H Maret W Fluctuations of cellular, available zinc modulate insulin signaling via inhibition of protein tyrosine phosphatases Journal of Trace Elements in Medicine and Biology 2005 19 37 42 10.1016/j.jtemb.2005.02.004 16240670

[B25] Beyersmann D Haase H Functions of zinc in signaling, proliferation and differentiation of mammalian cells Biometals 2001 14 331 341 10.1023/A:1012905406548 11831463

[B26] Hogstrand C Verbost PM Bonga SEW Inhibition of human erythrocyte Ca2+-ATPase by Zn2+ Toxicology 1999 133 139 145 10.1016/S0300-483X(99)00020-7 10378480

[B27] Haase H Mazzatti DJ White A Ibs KH Engelhardt G Hebel S Powell JR Rink L Differential gene expression after zinc supplementation and deprivation in human leukocyte subsets Molecular Medicine 2007 13 362 370 10.2119/2007-00049.Haase 17622302 PMC1952668

[B28] Cousins RJ Blanchard RK Popp MP Liu L Cao J Moore JB Green CL A global view of the selectivity of zinc deprivation and excess on genes expressed in human THP-1 mononuclear cells Proceedings of the National Academy of Sciences of the United States of America 2003 100 6952 6957 10.1073/pnas.0732111100 12756304 PMC165811

[B29] Mazzatti DJ Uciechowski P Hebel S Engelhardt G White AJ Powell JR Rink L Haase H Effects of long-term zinc supplementation and deprivation on gene expression in human THP-1 mononuclear cells Journal of Trace Elements in Medicine and Biology 2008 22 325 336 10.1016/j.jtemb.2008.06.002 19013360

[B30] Jackson KA Valentine RA McKay JA Swan DC Mathers JC Ford D Analysis of differential gene-regulatory responses to zinc in human intestinal and placental cell lines British Journal of Nutrition 2009 101 1474 1483 10.1017/S0007114508094634 18947441

[B31] Lin S-f Wei H Maeder D Franklin RB Feng P Profiling of zinc-altered gene expression in human prostate normal vs. cancer cells: a time course study The Journal of Nutritional Biochemistry 2009 20 12 1000 1012 10.1016/j.jnutbio.2008.09.004 19071009 PMC2821158

[B32] Coyle P Tran N Fung JNT Summers BL Rofe AM Maternal dietary zinc supplementation prevents aberrant behaviour in an object recognition task in mice offspring exposed to LPS in early pregnancy Behavioural Brain Research 2009 197 210 218 10.1016/j.bbr.2008.08.022 18793679

[B33] Dieck Ht Doring F Fuchs D Roth HP Daniel H Transcriptome and proteome analysis identifies the pathways that increase hepatic lipid accumulation in zinc-deficient rats JNutr 2005 135 199 205 10.1093/jn/135.2.199 15671213

[B34] Hwang P-P Lee T-H New insights into fish ion regulation and mitochondrion-rich cells Comparative Biochemistry and Physiology - Part A: Molecular & Integrative Physiology 2007 148 479 497 10.1016/j.cbpa.2007.06.416 17689996

[B35] Sun JY Jing MY Wang JF Zi NT Fu LJ Lu MQ Pan L Effect of zinc on biochemical parameters and changes in related gene expression assessed by cDNA microarrays in pituitary of growing rats Nutrition 2006 22 187 196 10.1016/j.nut.2005.07.007 16413754

[B36] Taccioli C Wan SG Liu CG Alder H Volinia S Farber JL Croce CM Fong LYY Zinc Replenishment Reverses Overexpression of the Proinflammatory Mediator S100A8 and Esophageal Preneoplasia in the Rat Gastroenterology 2009 136 953 966 10.1053/j.gastro.2008.11.039 19111725 PMC2650087

[B37] Zheng D Kille P Feeney GP Cunningham P Handy RD Hogstrand C Dynamic transcriptomic profiles of zebrafish gills in response to zinc depletion BMC Genomics 2010 11 548 10.1186/1471-2164-11-553 20932299 PMC3091697

[B38] Dennis G Sherman B Hosack D Yang J Gao W Lane H Lempicki R DAVID: Database for Annotation, Visualization, and Integrated Discovery Genome Biology 2003 4 R60 10.1186/gb-2003-4-9-r60 PMC193660 12734009

[B39] Huster D Purnat TD Burkhead JL Ralle M Fiehn O Stuckert F Olson NE Teupser D Lutsenko S High Copper Selectively Alters Lipid Metabolism and Cell Cycle Machinery in the Mouse Model of Wilson Disease The Journal of Biological Chemistry 2007 282 8343 8355 10.1074/jbc.M607496200 17205981

[B40] Hughes S Samman S The Effect of Zinc Supplementation in Humans on Plasma Lipids, Antioxidant Status and Thrombogenesis Journal of the American College of Nutrition 2006 25 285 291 16943449 10.1080/07315724.2006.10719537

[B41] Wong VVT Nissom PM Sim S-L Yeo JHM Chuah S-H Yap MGS Zinc as an insulin replacement in hybridoma cultures Biotechnology and Bioengineering 2006 93 553 563 10.1002/bit.20746 16224792

[B42] Ugarte M Osborne NN Zinc in the retina Progress in Neurobiology 2001 64 219 249 10.1016/S0301-0082(00)00057-5 11240307

[B43] Wingender E Dietze P Karas H Knuppel R TRANSFAC: A Database on Transcription Factors and Their DNA Binding Sites Nucl Acids Res 1996 24 238 241 10.1093/nar/24.1.238 8594589 PMC145586

[B44] Bury NR Chung MJ Sturm A Walker PA Hogstrand C Cortisol stimulates the zinc signaling pathway and expression of metallothioneins and ZnT1 in rainbow trout gill epithelial cells American Journal of Physiology-Regulatory Integrative and Comparative Physiology 2008 294 R623 R629 10.1152/ajpregu.00646.2007 18077514

[B45] Fukada T Civic N Furuichi T Shimoda S Mishima K Higashiyama H Idaira Y Asada Y Kitamura H Yamasaki S The Zinc Transporter SLC39A13/ZIP13 Is Required for Connective Tissue Development; Its Involvement in BMP/TGF-beta Signaling Pathways PLoS ONE 2008 3 e3642 10.1371/journal.pone.0003642 18985159 PMC2575416

[B46] Murakami M Hirano T Intracellular zinc homeostasis and zinc signaling Cancer Science 2008 99 1515 1522 10.1111/j.1349-7006.2008.00854.x 18754861 PMC11158020

[B47] Yamashita S Miyagi C Fukada T Kagara N Che Y-S Hirano T Zinc transporter LIVI controls epithelial-mesenchymal transition in zebrafish gastrula organizer Nature 2004 429 298 302 10.1038/nature02545 15129296

[B48] Andrews GK Wang HB Dey SK Palmiter RD Mouse zinc transporter 1 gene provides an essential function during early embryonic development Genesis 2004 40 74 81 10.1002/gene.20067 15452870

[B49] Hsiao C-D You M-S Guh Y-J Ma M Jiang Y-J Hwang P-P A Positive Regulatory Loop between *foxi3a *and *foxi3b *Is Essential for Specification and Differentiation of Zebrafish Epidermal Ionocytes PLoS ONE 2007 2 e302 10.1371/journal.pone.0000302 17375188 PMC1810426

[B50] Riobo NA Manning DR Pathways of signal transduction employed by vertebrate Hedgehogs The Biochemical Journal 2007 403 369 379 10.1042/BJ20061723 17419683

[B51] Fuccillo M Rutlin M Fishell G Removal of Pax6 Partially Rescues the Loss of Ventral Structures in Shh Null Mice Cerebral cortex 2006 16 i96 102 10.1093/cercor/bhk023 16766714

[B52] Bailey JM Singh PK Hollingsworth MA Cancer metastasis facilitated by developmental pathways: Sonic hedgehog, Notch, and bone morphogenic proteins Journal of Cellular Biochemistry 2007 102 829 839 10.1002/jcb.21509 17914743

[B53] Holleville N Mateos S Bontoux M Bollerot K Monsoro-Burq AH Dlx5 drives Runx2 expression and osteogenic differentiation in developing cranial suture mesenchyme Dev Biol 2007 304 860 874 10.1016/j.ydbio.2007.01.003 17335796

[B54] Kwun IS Cho YE Lomeda RA Shin HI Choi JY Kang YH Beattie JH Zinc deficiency suppresses matrix mineralization and retards osteogenesis transiently with catch-up possibly through Runx 2 modulation Bone 2010 46 732 741 10.1016/j.bone.2009.11.003 19913120

[B55] Jackson KA Valentine RA Coneyworth LJ Mathers JC Ford D Mechanisms of mammalian zinc-regulated gene expression Biochemical Society Transactions 2008 36 1262 1266 10.1042/BST0361262 19021537

[B56] Sandstrom B Micronutrient interactions: effects on absorption and bioavailability British Journal of Nutrition 2001 85 S181 S185 10.1079/BJN2000312 11509108

[B57] Olivares M Pizarro F Ruz M Zinc inhibits nonheme iron bioavailability in humans Biological Trace Element Research 2007 117 7 14 10.1007/BF02698079 17873388

[B58] Brewer GJ Use of zinc copper metabolic interactions in the treatment of Wilson disease Journal of the American College of Nutrition 1990 9 487 491 2258535 10.1080/07315724.1990.10720405

[B59] Kelleher SL Lonnerdal B Zinc Supplementation Reduces Iron Absorption through Age-Dependent Changes in Small Intestine Iron Transporter Expression in Suckling Rat Pups The Journal of Nutrition 2006 136 1185 1191 16614402 10.1093/jn/136.5.1185

[B60] Kizek R Trnková L Sevcíková S Smarda J Jelen F Silver Electrode as a Sensor for Determination of Zinc in Cell Cultivation Medium Analytical Biochemistry 2002 301 8 13 10.1006/abio.2001.5484 11811961

[B61] Ledwith BJ Johnson TE Wagner LK Pauley CJ Manam S Galloway SM Nichols WW Growth Regulation by Peroxisome Proliferators: Opposing Activities in Early and Late G1 Cancer Research 1996 56 3257 3264 8764118

[B62] Reiterer G Toborek M Hennig B Peroxisome proliferator activated receptors alpha and gamma require zinc for their anti-inflammatory properties in porcine vascular endothelial cells Journal of Nutrition 2004 134 1711 1715 15226458 10.1093/jn/134.7.1711

[B63] Kang X Zhong W Liu J Song Z McClain CJ Kang YJ Zhou Z Zinc supplementation reverses alcohol-induced steatosis in mice through reactivating hepatocyte nuclear factor-4alpha and peroxisome proliferator-activated receptor-alpha Hepatology 2009 19637192 10.1002/hep.23090 PMC2757527

[B64] Bunger M van den Bosch HM van der Meijde J Kersten S Hooiveld G Muller M Genome-wide analysis of PPAR alpha activation in murine small intestine Physiological Genomics 2007 30 192 204 10.1152/physiolgenomics.00198.2006 17426115

[B65] Weber LW Boll M Stampfl A Maintaining cholesterol homeostasis: sterol regulatory element-binding proteins World Journal of Gastroenterology 2004 10 6 10.3748/wjg.v10.i21.3081 PMC4611246 15457548

[B66] Boukaiba N Flament C Acher S Chappuis P Piau A Fusselier M Dardenne M Lemonnier D A Physiological amount of zinc supplementation - effects on nutritional, lipid, and thymic status in an elderly population American Journal of Clinical Nutrition 1993 57 566 572 8460613 10.1093/ajcn/57.4.566

[B67] Fosmire GJ Zinc toxicity American Journal of Clinical Nutrition 1990 51 225 227 2407097 10.1093/ajcn/51.2.225

[B68] Lee CH Olson P Evans RM Minireview: Lipid metabolism, metabolic diseases, and peroxisome proliferator-activated receptors Endocrinology 2003 144 2201 2207 10.1210/en.2003-0288 12746275

[B69] Li AC Glass CK PPAR- and LXR-dependent pathways controlling lipid metabolism and the development of atherosclerosis Journal of Lipid Research 2004 45 2161 2173 10.1194/jlr.R400010-JLR200 15489539

[B70] Eby GA Halcomb WW High-dose zinc to terminate angina pectoris: A review and hypothesis for action by ICAM inhibition Medical Hypotheses 2006 66 169 172 10.1016/j.mehy.2005.06.013 16084666

[B71] Blanquart C Barbier O Fruchart JC Staels B Glineur C Peroxisome proliferator-activated receptors: regulation of transcriptional activities and roles in inflammation The Journal of Steroid Biochemistry and Molecular Biology 2003 85 267 273 10.1016/S0960-0760(03)00214-0 12943712

[B72] Handy R Musonda M Phillips C Falla S Mechanisms of gastrointestinal copper absorption in the African walking catfish: copper dose-effects and a novel anion-dependent pathway in the intestine The Journal of Experimental Biology 2000 203 2365 2377 10887075 10.1242/jeb.203.15.2365

[B73] Baker RTM Davies SJ Changes in tissue alpha-tocopherol status and degree of lipid peroxidation with varying alpha-tocopheryl acetate inclusion in diets for the African catfish Aquaculture Nutrition 1996 2 71 79 10.1111/j.1365-2095.1996.tb00011.x

[B74] Hogstrand C Webb N Wood CM Covariation in regulation of affinity for branchial zinc and calcium uptake in freshwater rainbow trout Journal of Experimental Biology 1998 201 1809 1815 9576891 10.1242/jeb.201.11.1809

