# Supplementary material for: Dynamic transcriptomic profiles of zebrafish gills in response to zinc supplementation
Source: BMC Genomics. 2010 Oct 11;11:553. doi: 10.1186/1471-2164-11-553 (PMC3091702; doi:10.1186/1471-2164-11-553)
Supplement: Additional file 2 — Interactive Direct Interaction Network representing the molecular interactions between zinc, copper, iron, calcium and proteins encoded by transcripts changed by zinc supplementation. Mini web-site containing index.html and hyperlinked pages in subdirectory describing a Direct Interaction Network automatically generated based on curated interactions contained within the proprietary PathwayArchitect database. Ovals represent proteins and the circles symbolize metal ions. Objects are coloured by their abundance in zebrafish at the time-point they were significantly different from the control is a scale from -4 fold (dark green) to +4 fold (dark red). Where significant differences were found at more than one time-point, the colour overlay shows expression at the first instance. Dark blue squares denote 'binding', and light blue squares 'expression'; green squares stand for 'regulation', green diamonds for 'metabolism', and green circles for 'promoter binding'. Arrow heads indicate directionality of the interaction where annotated. All nodes and edges can be further interrogated by selecting the relative area of the image. [file 1471-2164-11-553-S2.zip › PathwayArchitect Zn xs DIN/100931.html]

# PROTEIN: MYL2

|  |  |
| --- | --- |
| Name | MYL2 |
| Type | PROTEIN |
| Description | myosin, light polypeptide 2, regulatory, cardiac, slow |
| Note | MYL2 encodes the regulatory light chain associated with cardiac myosin beta (or slow) heavy chain. Ca+ triggers the phosphorylation of regulatory light chain that in turn triggers contraction. Mutations in MYL2 are associated with mid-left ventricular chamber type hypertrophic cardiomyopathy. |
| Alias | ventricular myosin regulatory light chain |
|  | MYL2 |
|  | myosin light chain 2 |
|  | G2 |
|  | myosin light chain, phosphorylatable, cardiac ventricles |
|  | Myosin light polypeptide 2 alkali; ventricular skeletal slow |
|  | Myolc1 |
|  | Mylpf |
|  | DTNB |
|  | CMH10 |
|  | MLC2 |
|  | MLC-2v |
|  | MLC-2 |
|  | myosin light chain 2v |
|  | Mylpc |
|  | Myosin, light polypeptide 2, alkali; ventricular, skeletal, slow |
|  | Mlc2v |
|  | Myl2 |


---

|  |  |
| --- | --- |
| GO Component | cytoskeleton |
|  | myosin |


---

|  |  |
| --- | --- |
| GO ID | GO:0005856 |
|  | GO:0005509 |
|  | GO:0016459 |
|  | GO:0003774 |
|  | GO:0007010 |
|  | GO:0008307 |
|  | GO:0007517 |
|  | GO:0006942 |


---

|  |  |
| --- | --- |
| MIM | MIM:160781 |
|  | MIM:608758 |


---

|  |  |
| --- | --- |
| Connectivity | 238 |


---

|  |  |
| --- | --- |
| Entrez ID | 17906 |
|  | 4633 |
|  | 24584 |


---

|  |  |
| --- | --- |
| Agilent ID | A\_23\_P162547 |
|  | A\_14\_P200356 |
|  | A\_44\_P1005462 |
|  | A\_14\_P113933 |
|  | A\_51\_P392459 |
|  | A\_53\_P160867 |
|  | A\_43\_P11483 |


---

|  |  |
| --- | --- |
| Cellular Localization | Cytoskeleton |
|  | Organelle |
|  | Cell |


---

|  |  |
| --- | --- |
| Pathway | ERK-PI3K (Collagen) Signaling |
|  | Zn xs inventory |
|  | Zn xs DIN |


---

|  |  |
| --- | --- |
| GO Process | cytoskeleton organization and biogenesis |
|  | regulation of striated muscle contraction |
|  | muscle development |


---

|  |  |
| --- | --- |
| UniGene | Rn.6534 |
|  | Mm.1529 |
|  | Hs.75535 |


---

|  |  |
| --- | --- |
| Affymetrix Probeset ID | 1387787\_at |
|  | 1448394\_at |
|  | 209742\_s\_at |
|  | 36640\_at |
|  | 80815\_at |
|  | 93050\_at |
|  | g2460246\_3p\_a\_at |
|  | M91602\_f\_at |
|  | Msa.1007.0\_f\_at |
|  | Msa.31372.0\_s\_at |
|  | Msa.39105.0\_f\_at |
|  | rc\_AA998118\_at |
|  | rc\_AI230218\_at |
|  | X00975\_at |
|  | X00975\_g\_at |
|  | X66141\_at |
|  | TC28358\_f\_at |


---

|  |  |
| --- | --- |
| GO Function | structural constituent of muscle |
|  | motor activity |
|  | calcium ion binding |


---

|  |  |
| --- | --- |
| Nucleotide | BC061144 |
|  | X66141 |
|  | M91602 |
|  | CR456962 |
|  | AK146674 |
|  | NM\_010861 |
|  | X00975 |
|  | BC031006 |
|  | S69022 |
|  | AF020768 |
|  | NM\_012605 |
|  | J00754 |
|  | AK002367 |
|  | M22815 |
|  | CR541957 |
|  | BC015821 |
|  | AF302688 |
|  | NM\_000432 |
|  | BC031008 |
|  | X57542 |
|  | X14332 |


---

|  |  |
| --- | --- |
| Protein | AAA39796 |
|  | CAA25480 |
|  | P04466 |
|  | AAB29658 |
|  | CAA32510 |
|  | AAG40240 |
|  | AAA41660 |
|  | AAH31008 |
|  | P10916 |
|  | BAB22045 |
|  | NP\_036737 |
|  | CAG33243 |
|  | BAE27350 |
|  | AAH15821 |
|  | AAH31006 |
|  | AAA91832 |
|  | CAG46755 |
|  | NP\_034991 |
|  | AAH61144 |
|  | P51667 |
|  | CAA46931 |
|  | AAB91993 |
|  | NP\_000423 |
|  | CAA40761 |


---

|  |  |
| --- | --- |
| Organism | Mammal |


---

|  |  |
| --- | --- |
| Location | chromosome 1, 1q36 (Rattus norvegicus) |
|  | chromosome 12, 12q23-q24.3 (Homo sapiens) |
|  | chromosome 5, 5 F (Mus musculus) |


---

|  |  |
| --- | --- |
