# Supplementary material for: Dynamic transcriptomic profiles of zebrafish gills in response to zinc supplementation
Source: BMC Genomics. 2010 Oct 11;11:553. doi: 10.1186/1471-2164-11-553 (PMC3091702; doi:10.1186/1471-2164-11-553)
Supplement: Additional file 2 — Interactive Direct Interaction Network representing the molecular interactions between zinc, copper, iron, calcium and proteins encoded by transcripts changed by zinc supplementation. Mini web-site containing index.html and hyperlinked pages in subdirectory describing a Direct Interaction Network automatically generated based on curated interactions contained within the proprietary PathwayArchitect database. Ovals represent proteins and the circles symbolize metal ions. Objects are coloured by their abundance in zebrafish at the time-point they were significantly different from the control is a scale from -4 fold (dark green) to +4 fold (dark red). Where significant differences were found at more than one time-point, the colour overlay shows expression at the first instance. Dark blue squares denote 'binding', and light blue squares 'expression'; green squares stand for 'regulation', green diamonds for 'metabolism', and green circles for 'promoter binding'. Arrow heads indicate directionality of the interaction where annotated. All nodes and edges can be further interrogated by selecting the relative area of the image. [file 1471-2164-11-553-S2.zip › PathwayArchitect Zn xs DIN/101279.html]

# PROTEIN: GSTT1

|  |  |
| --- | --- |
| Name | GSTT1 |
| Type | PROTEIN |
| Description | glutathione S-transferase theta 1 |
| Note | Glutathione S-transferase (GST) theta 1 (GSTT1) is a member of a superfamily of proteins that catalyze the conjugation of reduced glutathione to a variety of electrophilic and hydrophobic compounds. Human GSTs can be divided into five main classes: alpha, mu, pi, theta, and zeta. The theta class includes GSTT1 and GSTT2. The GSTT1 and GSTT2 share 55% amino acid sequence identity and both of them were claimed to have an important role in human carcinogenesis. The GSTT1 gene is located approximately 50kb away from the GSTT2 gene. The GSTT1 and GSTT2 genes have a similar structure, being composed of five exons with identical exon/intron boundaries. |
| Alias | GSTYRS |
|  | GST class-theta 1 |
|  | Glutathione S-transferase 5 |
|  | AI255817 |
|  | Glutathione S-transferase 1 (theta) |
|  | Glutathione transferase T1-1 |
|  | GST 5-5 |
|  | GSTT1 |
|  | Gstt1 |
|  | glutathione S-transferase, theta 1 |
|  | Gstt1-1 |


---

|  |  |
| --- | --- |
| GO Component | nucleus |


---

|  |  |
| --- | --- |
| GO ID | GO:0004364 |
|  | GO:0006749 |
|  | GO:0005634 |
|  | GO:0006950 |
|  | GO:0016740 |


---

|  |  |
| --- | --- |
| MIM | MIM:600436 |


---

|  |  |
| --- | --- |
| Connectivity | 105 |


---

|  |  |
| --- | --- |
| Entrez ID | 2952 |
|  | 14871 |
|  | 25260 |


---

|  |  |
| --- | --- |
| Agilent ID | A\_53\_P106454 |
|  | A\_51\_P179664 |
|  | A\_51\_P179663 |
|  | A\_42\_P701582 |
|  | A\_24\_P319001 |
|  | A\_53\_P160260 |
|  | A\_14\_P111340 |
|  | A\_32\_P69284 |
|  | A\_23\_P254944 |


---

|  |  |
| --- | --- |
| Cellular Localization | Nucleus |
|  | Organelle |
|  | Cell |


---

|  |  |
| --- | --- |
| DbXref | KEGG pathway##00480##Glutathione metabolism##http://www.genome.jp/dbget-bin/show\_pathway?hsa00480+2952 |
|  | KEGG pathway##00480##Glutathione metabolism##http://www.genome.jp/dbget-bin/show\_pathway?mmu00480+14871 |
|  | KEGG pathway##00480##Glutathione metabolism##http://www.genome.jp/dbget-bin/show\_pathway?rno00480+25260 |


---

|  |  |
| --- | --- |
| Pathway | Zn xs inventory |
|  | Zn xs DIN |


---

|  |  |
| --- | --- |
| GO Process | glutathione metabolism |
|  | response to stress |


---

|  |  |
| --- | --- |
| UniGene | Mm.2746 |
|  | Rn.11122 |
|  | Hs.268573 |


---

|  |  |
| --- | --- |
| Affymetrix Probeset ID | 1368354\_at |
|  | 1418186\_at |
|  | 203815\_at |
|  | 232193\_3p\_at |
|  | 232193\_at |
|  | 37222\_at |
|  | 375\_at |
|  | 54188\_i\_at |
|  | 95019\_at |
|  | g4504184\_3p\_at |
|  | Hs.268573.0.S1\_3p\_at |
|  | Hs.268573.0.S1\_3p\_x\_at |
|  | X67654\_at |
|  | X98055\_s\_at |
|  | Z84718\_cds1\_at |
|  | 373\_at |
|  | 83743\_at |
|  | RC\_T96690\_at |
|  | TC24078\_at |


---

|  |  |
| --- | --- |
| EC Number | EC 2.5.1.18 |


---

|  |  |
| --- | --- |
| GO Function | glutathione transferase activity |
|  | transferase activity |


---

|  |  |
| --- | --- |
| Nucleotide | Z84718 |
|  | AB057594 |
|  | BC086426 |
|  | BC055020 |
|  | BC050410 |
|  | AF240786 |
|  | BC007065 |
|  | BT019951 |
|  | X79389 |
|  | X98055 |
|  | AF435971 |
|  | AK002338 |
|  | CR456499 |
|  | NM\_053293 |
|  | NM\_008185 |
|  | X67654 |
|  | BC012254 |
|  | NM\_000853 |


---

|  |  |
| --- | --- |
| Protein | BAB22023 |
|  | Q01579 |
|  | AAG02374 |
|  | NP\_000844 |
|  | CAA55935 |
|  | Q64471 |
|  | CAA66665 |
|  | CAA47896 |
|  | P30711 |
|  | CAG30385 |
|  | NP\_445745 |
|  | AAH12254 |
|  | AAH86426 |
|  | AAH07065 |
|  | AAV38754 |
|  | BAB39498 |
|  | NP\_032211 |
|  | AAH55020 |
|  | AAL31549 |


---

|  |  |
| --- | --- |
| Organism | Mammal |


---

|  |  |
| --- | --- |
| Location | 10 40.7 cM (Mus musculus) |
|  | chromosome 22, 22q11.23 (Homo sapiens) |
|  | chromosome 20, 20p12 (Rattus norvegicus) |
|  | chromosome 10, 10 40.7 cM, 10 B5-C1 (Mus musculus) |


---

|  |  |
| --- | --- |
