# Supplementary material for: Dynamic transcriptomic profiles of zebrafish gills in response to zinc supplementation
Source: BMC Genomics. 2010 Oct 11;11:553. doi: 10.1186/1471-2164-11-553 (PMC3091702; doi:10.1186/1471-2164-11-553)
Supplement: Additional file 2 — Interactive Direct Interaction Network representing the molecular interactions between zinc, copper, iron, calcium and proteins encoded by transcripts changed by zinc supplementation. Mini web-site containing index.html and hyperlinked pages in subdirectory describing a Direct Interaction Network automatically generated based on curated interactions contained within the proprietary PathwayArchitect database. Ovals represent proteins and the circles symbolize metal ions. Objects are coloured by their abundance in zebrafish at the time-point they were significantly different from the control is a scale from -4 fold (dark green) to +4 fold (dark red). Where significant differences were found at more than one time-point, the colour overlay shows expression at the first instance. Dark blue squares denote 'binding', and light blue squares 'expression'; green squares stand for 'regulation', green diamonds for 'metabolism', and green circles for 'promoter binding'. Arrow heads indicate directionality of the interaction where annotated. All nodes and edges can be further interrogated by selecting the relative area of the image. [file 1471-2164-11-553-S2.zip › PathwayArchitect Zn xs DIN/101302.html]

# PROTEIN: SERPINA1

|  |  |
| --- | --- |
| Name | SERPINA1 |
| Type | PROTEIN |
| Description | serpin peptidase inhibitor, clade A (alpha-1 antiproteinase, antitrypsin), member 1 |
| Note | Alpha-1-antitrypsin is a protease inhibitor, deficiency of which is associated with emphysema and liver disease. The protein is encoded by a gene (PI) located on the distal long arm of chromosome 14.[supplied by OMIM] |
| Alias | serine (or cysteine) proteinase inhibitor, clade A (alpha-1 antiproteinase, antitrypsin), member 1 |
|  | PRO2209 |
|  | Alpha-1- proteinase inhibitor |
|  | serine protease inhibitor alpha 1 |
|  | PI1 |
|  | MGC23330 |
|  | Serpina1 |
|  | alpha-1-protease inhibitor |
|  | Alpha-1-antitrypsin |
|  | serine (or cysteine) proteinase inhibitor, clade A, member 1 |
|  | PRO2275 |
|  | alpha-1-antitrypsin |
|  | serine (or cysteine) proteinase inhibitor clade A member 1 |
|  | Alpha-1 protease inhibitor |
|  | SERPINA1 |
|  | Alpha-1- antiproteinase |
|  | A1AT |
|  | AAT |
|  | MGC9222 |
|  | Pi |
|  | protease inhibitor 1 (anti-elastase), alpha-1-antitrypsin |
|  | Spi1 |
|  | PI |
|  | PI;ORFNames=PRO0684 |
|  | alpha-1-antitrypsin (protease inhibitor) |
|  | A1A |
|  | serine (or cysteine) proteinase inhibitor clade A (alpha-1 antiproteinase antitrypsin) member 1 |


---

|  |  |
| --- | --- |
| GO Component | extracellular region |


---

|  |  |
| --- | --- |
| GO ID | GO:0005515 |
|  | GO:0004867 |
|  | GO:0005576 |
|  | GO:0006953 |


---

|  |  |
| --- | --- |
| MIM | MIM:107400 |


---

|  |  |
| --- | --- |
| Connectivity | 585 |


---

|  |  |
| --- | --- |
| Entrez ID | 5265 |
|  | 24648 |


---

|  |  |
| --- | --- |
| Agilent ID | A\_23\_P218111 |
|  | A\_14\_P115012 |
|  | A\_14\_P133662 |
|  | A\_44\_P524718 |
|  | A\_43\_P15291 |


---

|  |  |
| --- | --- |
| Cellular Localization | Extracellular region |


---

|  |  |
| --- | --- |
| DbXref | Reactome##109582##Hemostasis##http://www.reactome.org/cgi-bin/eventbrowser?DB=gk\_current&ID=109582 |
|  | KEGG pathway##04610##Complement and coagulation cascades##http://www.genome.jp/dbget-bin/show\_pathway?rno04610+24648 |
|  | KEGG pathway##04610##Complement and coagulation cascades##http://www.genome.jp/dbget-bin/show\_pathway?hsa04610+5265 |


---

|  |  |
| --- | --- |
| Pathway | Zn xs inventory |
|  | Zn xs DIN |


---

|  |  |
| --- | --- |
| GO Process | acute-phase response |


---

|  |  |
| --- | --- |
| UniGene | Rn.1419 |
|  | Hs.525557 |


---

|  |  |
| --- | --- |
| Affymetrix Probeset ID | 1367647\_at |
|  | 202833\_s\_at |
|  | 211428\_at |
|  | 211429\_s\_at |
|  | 230318\_at |
|  | 36781\_at |
|  | 68033\_at |
|  | 1381067\_at |
|  | g4505792\_3p\_a\_at |
|  | K01396\_at |
|  | rc\_AI010453\_at |
|  | X16273cds\_at |
|  | X16273cds\_g\_at |
|  | Hs.297681.4.A1\_3p\_at |
|  | RC\_C20919\_at |
|  | RC\_R06726\_s\_at |


---

|  |  |
| --- | --- |
| GO Function | serine-type endopeptidase inhibitor activity |
|  | protein binding |


---

|  |  |
| --- | --- |
| Nucleotide | BG483969 |
|  | AA317159 |
|  | X05826 |
|  | NM\_022519 |
|  | BX477352 |
|  | BC011991 |
|  | K02212 |
|  | BX161449 |
|  | J00064 |
|  | BX247968 |
|  | BM923322 |
|  | X17122 |
|  | BX248257 |
|  | NM\_001002235 |
|  | J00067 |
|  | BM844817 |
|  | V00496 |
|  | AK026174 |
|  | AF119873 |
|  | CR590437 |
|  | M32247 |
|  | X01683 |
|  | BC070163 |
|  | J02619 |
|  | M11465 |
|  | BC015642 |
|  | K01396 |
|  | X02920 |
|  | X16273 |
|  | D00675 |
|  | X05827 |
|  | D17206 |
|  | BC078824 |
|  | NM\_000295 |
|  | M26123 |
|  | BT019455 |
|  | NM\_001002236 |
|  | BX248002 |
|  | J00066 |


---

|  |  |
| --- | --- |
| Protein | CAA29267 |
|  | AAH15642 |
|  | CAA34349 |
|  | AAA51547 |
|  | CAA25838 |
|  | NP\_001002236 |
|  | P01009 |
|  | CAD62585 |
|  | CAA26677 |
|  | NP\_071964 |
|  | AAV38262 |
|  | NP\_000286 |
|  | CAD62334 |
|  | BAA00579 |
|  | AAB59495 |
|  | AAH78824 |
|  | AAB59371 |
|  | AAA51545 |
|  | P17475 |
|  | CAD62306 |
|  | AAB59375 |
|  | CAD61914 |
|  | AAF69627 |
|  | AAB59369 |
|  | NP\_001002235 |
|  | CAA23755 |
|  | AAA40788 |
|  | CAA34982 |
|  | AAH11991 |
|  | AAB59370 |
|  | AAA51546 |


---

|  |  |
| --- | --- |
| Organism | Mammal |


---

|  |  |
| --- | --- |
| Location | chromosome 14, 14q32.1 (Homo sapiens) |
|  | chromosome 6, 6q32 (Rattus norvegicus) |


---

|  |  |
| --- | --- |
