# Supplementary material for: Dynamic transcriptomic profiles of zebrafish gills in response to zinc supplementation
Source: BMC Genomics. 2010 Oct 11;11:553. doi: 10.1186/1471-2164-11-553 (PMC3091702; doi:10.1186/1471-2164-11-553)
Supplement: Additional file 2 — Interactive Direct Interaction Network representing the molecular interactions between zinc, copper, iron, calcium and proteins encoded by transcripts changed by zinc supplementation. Mini web-site containing index.html and hyperlinked pages in subdirectory describing a Direct Interaction Network automatically generated based on curated interactions contained within the proprietary PathwayArchitect database. Ovals represent proteins and the circles symbolize metal ions. Objects are coloured by their abundance in zebrafish at the time-point they were significantly different from the control is a scale from -4 fold (dark green) to +4 fold (dark red). Where significant differences were found at more than one time-point, the colour overlay shows expression at the first instance. Dark blue squares denote 'binding', and light blue squares 'expression'; green squares stand for 'regulation', green diamonds for 'metabolism', and green circles for 'promoter binding'. Arrow heads indicate directionality of the interaction where annotated. All nodes and edges can be further interrogated by selecting the relative area of the image. [file 1471-2164-11-553-S2.zip › PathwayArchitect Zn xs DIN/101452.html]

# PROTEIN: ESR1

|  |  |
| --- | --- |
| Name | ESR1 |
| Type | PROTEIN |
| Description | estrogen receptor 1 |
| Note | The estrogen receptor (ESR) is a ligand-activated transcription factor composed of several domains important for hormone binding, DNA binding, and activation of transcription. Alternative splicing results in several ESR1 mRNA transcripts, which differ primarily in their 5-prime untranslated regions. The translated receptors show less variability.[supplied by OMIM] |
| Alias | dJ443C4.1.1 (estrogen receptor 1) |
|  | major ORF |
|  | Era |
|  | ESR |
|  | estrogen receptor protein |
|  | ER[a] |
|  | RNESTROR |
|  | ER |
|  | NR3A1 |
|  | ESRA |
|  | estrogen receptor 1 (alpha) |
|  | Estrogen receptor alpha |
|  | Er alpha |
|  | DKFZp686N23123 |
|  | ER-alpha |
|  | ERalpha |
|  | Esr |
|  | oestrogen receptor |
|  | estrogen receptor alpha |
|  | AA420328 |
|  | steroid hormone receptor |
|  | Estra |
|  | AU041214 |
|  | Estradiol receptor |
|  | Esr1 |
|  | Nr3a1 |
|  | ERa |
|  | Estr |


---

|  |  |
| --- | --- |
| GO Component | nucleus |
|  | chromatin remodeling complex |
|  | membrane |
|  | cytoplasm |


---

|  |  |
| --- | --- |
| GO ID | GO:0030235 |
|  | GO:0003707 |
|  | GO:0016020 |
|  | GO:0016585 |
|  | GO:0003677 |
|  | GO:0006355 |
|  | GO:0030528 |
|  | GO:0005737 |
|  | GO:0016049 |
|  | GO:0005515 |
|  | GO:0008270 |
|  | GO:0003700 |
|  | GO:0005496 |
|  | GO:0030520 |
|  | GO:0005634 |
|  | GO:0007165 |
|  | GO:0004872 |
|  | GO:0030284 |
|  | GO:0004879 |
|  | GO:0046872 |
|  | GO:0045944 |
|  | GO:0006350 |
|  | GO:0045839 |


---

|  |  |
| --- | --- |
| MIM | MIM:157300 |
|  | MIM:133430 |


---

|  |  |
| --- | --- |
| Connectivity | 2471 |


---

|  |  |
| --- | --- |
| Entrez ID | 13982 |
|  | 2099 |
|  | 24890 |


---

|  |  |
| --- | --- |
| Agilent ID | A\_53\_P159636 |
|  | A\_53\_P128821 |
|  | A\_43\_P15442 |
|  | A\_53\_P164384 |
|  | A\_24\_P383478 |
|  | A\_23\_P309739 |
|  | A\_14\_P121989 |
|  | A\_44\_P401615 |
|  | A\_24\_P325215 |
|  | A\_51\_P474658 |
|  | A\_53\_P161413 |
|  | A\_14\_P100488 |
|  | A\_14\_P136162 |
|  | A\_52\_P237077 |
|  | A\_23\_P59308 |
|  | A\_14\_P119986 |
|  | A\_53\_P109210 |


---

|  |  |
| --- | --- |
| Cellular Localization | Cytoplasm |
|  | Nucleus |
|  | Membrane |
|  | Cell |
|  | Organelle |


---

|  |  |
| --- | --- |
| Pathway | Breast Cancer |
|  | Zn xs inventory |
|  | Zn xs DIN |


---

|  |  |
| --- | --- |
| GO Process | estrogen receptor signaling pathway |
|  | signal transduction |
|  | negative regulation of mitosis |
|  | regulation of transcription, DNA-dependent |
|  | cell growth |
|  | transcription |
|  | positive regulation of transcription from RNA polymerase II promoter |


---

|  |  |
| --- | --- |
| UniGene | Mm.9213 |
|  | Hs.208124 |
|  | Rn.10595 |


---

|  |  |
| --- | --- |
| Affymetrix Probeset ID | 115428\_at |
|  | 1298\_at |
|  | 133838\_at |
|  | 1387704\_at |
|  | 1421244\_at |
|  | 1435663\_at |
|  | 1457877\_at |
|  | 1460591\_at |
|  | 161113\_at |
|  | 1681\_at |
|  | 205225\_at |
|  | 211233\_x\_at |
|  | 211234\_x\_at |
|  | 211235\_s\_at |
|  | 211627\_3p\_x\_at |
|  | 211627\_x\_at |
|  | 215551\_at |
|  | 215552\_s\_at |
|  | 217163\_at |
|  | 217190\_x\_at |
|  | 234275\_at |
|  | 33670\_at |
|  | 1384868\_at |
|  | 93945\_at |
|  | 93946\_at |
|  | 93947\_g\_at |
|  | g11907834\_3p\_a\_at |
|  | g4503602\_3p\_at |
|  | Hs.247976.0.S1\_3p\_s\_at |
|  | Hs.272288.0.S1\_3p\_at |
|  | Hs.306314.0.S1\_3p\_at |
|  | Msa.36383.0\_f\_at |
|  | Msa.625.0\_at |
|  | S52128\_s\_at |
|  | X03635\_at |
|  | X86816\_at |
|  | Hs.247938.0.S1\_3p\_at |
|  | 97093\_at |
|  | RC\_AA164586\_s\_at |
|  | RC\_AA291749\_s\_at |
|  | TC32734\_at |
|  | rc\_AA866269\_at |


---

|  |  |
| --- | --- |
| GO Function | ligand-dependent nuclear receptor activity |
|  | transcription regulator activity |
|  | DNA binding |
|  | estrogen receptor activity |
|  | nitric-oxide synthase regulator activity |
|  | protein binding |
|  | transcription factor activity |
|  | steroid hormone receptor activity |
|  | steroid binding |
|  | receptor activity |
|  | zinc ion binding |
|  | metal ion binding |


---

|  |  |
| --- | --- |
| Nucleotide | AJ272162 |
|  | AF326912 |
|  | AK039911 |
|  | M12674 |
|  | AL049821 |
|  | AL590993 |
|  | NM\_007956 |
|  | AJ276597 |
|  | X73067 |
|  | AF258449 |
|  | AK041525 |
|  | AF258451 |
|  | Y00102 |
|  | AF258450 |
|  | AF128221 |
|  | AJ272165 |
|  | X62462 |
|  | X61098 |
|  | NM\_012689 |
|  | M69297 |
|  | AK054182 |
|  | AK033270 |
|  | AL078582 |
|  | AJ272166 |
|  | X03635 |
|  | AY425004 |
|  | BX640939 |
|  | U68068 |
|  | U68067 |
|  | NM\_000125 |
|  | AJ272163 |
|  | AK139745 |
|  | AK136228 |
|  | AK134392 |
|  | AF169237 |
|  | M38652 |
|  | AL356311 |
|  | X98236 |
|  | AY750962 |
|  | U47678 |
|  | AJ272164 |
|  | AF120105 |
|  | AK036627 |
|  | AJ272161 |
|  | AF128220 |
|  | AF123500 |
|  | M38651 |
|  | AK077236 |
|  | AA023625 |
|  | AK087638 |
|  | AU041214 |
|  | Z75126 |


---

|  |  |
| --- | --- |
| Protein | NP\_036821 |
|  | CAA68287 |
|  | CAI42286 |
|  | CAE45969 |
|  | CAI22123 |
|  | AAA52399 |
|  | AAA58462 |
|  | BAE22884 |
|  | CAA44322 |
|  | AAF22562 |
|  | AAG41359 |
|  | CAI14237 |
|  | NP\_031982 |
|  | NP\_000116 |
|  | CAI21013 |
|  | AAG41360 |
|  | AAF22561 |
|  | P03372 |
|  | CAI21011 |
|  | AAQ91815 |
|  | P06211 |
|  | AAD52984 |
|  | CAA99436 |
|  | BAE24122 |
|  | CAI42285 |
|  | CAI22124 |
|  | AAG41358 |
|  | CAA51528 |
|  | AAW69860 |
|  | AAC51875 |
|  | BAC30973 |
|  | BAC29510 |
|  | AAA37580 |
|  | AAD23565 |
|  | AAB00115 |
|  | CAI21012 |
|  | CAA43411 |
|  | AAG42501 |
|  | P19785 |
|  | AAC51874 |
|  | CAB85618 |
|  | CAA66888 |
|  | CAA27284 |
|  | CAI14238 |


---

|  |  |
| --- | --- |
| Organism | Mammal |


---

|  |  |
| --- | --- |
| Location | 10 12.0 cM (Mus musculus) |
|  | chromosome 6, 6q25.1 (Homo sapiens) |
|  | chromosome 10, 10 12.0 cM, 10 A1 (Mus musculus) |
|  | chromosome 1, 1q12 (Rattus norvegicus) |


---

|  |  |
| --- | --- |
