# Supplementary material for: Dynamic transcriptomic profiles of zebrafish gills in response to zinc supplementation
Source: BMC Genomics. 2010 Oct 11;11:553. doi: 10.1186/1471-2164-11-553 (PMC3091702; doi:10.1186/1471-2164-11-553)
Supplement: Additional file 2 — Interactive Direct Interaction Network representing the molecular interactions between zinc, copper, iron, calcium and proteins encoded by transcripts changed by zinc supplementation. Mini web-site containing index.html and hyperlinked pages in subdirectory describing a Direct Interaction Network automatically generated based on curated interactions contained within the proprietary PathwayArchitect database. Ovals represent proteins and the circles symbolize metal ions. Objects are coloured by their abundance in zebrafish at the time-point they were significantly different from the control is a scale from -4 fold (dark green) to +4 fold (dark red). Where significant differences were found at more than one time-point, the colour overlay shows expression at the first instance. Dark blue squares denote 'binding', and light blue squares 'expression'; green squares stand for 'regulation', green diamonds for 'metabolism', and green circles for 'promoter binding'. Arrow heads indicate directionality of the interaction where annotated. All nodes and edges can be further interrogated by selecting the relative area of the image. [file 1471-2164-11-553-S2.zip › PathwayArchitect Zn xs DIN/1016449.html]

# BINDING:

|  |  |
| --- | --- |
| Type | BINDING |
| Effect | None |


---

|  |  |
| --- | --- |
| Score | 0 |


---

|  |  |
| --- | --- |
| Reference Count | 10 |


---

|  |  |
| --- | --- |
| Mechanism | Unknown |


---

|  |  |
| --- | --- |
| Reference:0 || Sentence | "To determine some of the mechanisms which regulate the collagenase response, synovial cells were exposed to a cyclooxygenase inhibitor (indomethacin) and substances which alter the cytoskeleton (cytochalasin B or colchicine) or interact with Ca2+ X calmodulin (trifluoperazine)." |
| PMID | 6089839 |
| Year | 1984 |
| Species | Human |
| Journal | Biochem Pharmacol |
| RefScore | 0 |
| Source | PArchNLP |
  |
|


---

|  |  |
| --- | --- |
 Reference:1 || PMID | 10356396 |
| SourceID | 229135 |
| Species | Human |
| Experimental Condition | in-vitro |
| Description | The three dimensional structure of Gelatinase A (Full-Length) was determined by crystallography. The crystal was grown by using Gelatinase A, pH 7.8, at 100K. X-ray data were collected using synchrotron radiation and were 99.7% complete to a resolution limit of 2.800 angstroms and with an overall Rsym of 11.7%. This crystal belonged to the space group I 41 2 2. The structure was determined using Molecular Replacement and the modeled structure had an R-free = 32.7%. PDB: 1CK7, MMDB: 10832. |
| Detection Method | three-dimensional-structure |
| Source | BIND |
  ||


---

|  |  |
| --- | --- |
 Reference:2 || PMID | 12032297 |
| SourceID | 229135 |
| Experimental Condition | in-vitro |
| Description | The three dimensional structure of Prommp-2TIMP-2 Complex was determined by crystallography. The crystal was grown by using 72 Kda Type IV Collagenase, Metalloproteinase Inhibitor 2, pH 8.5, at 100K. X-ray data were collected using synchrotron radiation and were 94.1% complete to a resolution limit of 3.1 angstroms with an overall Rsym of 1.1%. This crystal contained 4 chains per asymmetric unit and belonged to the space group C 2 2 21. The structure was determined using Molecular Replacement and the modeled structure was refined to an R-factor = 27.8% and had an R-free = 33.3%. PDB: 1GXD, MMDB: 20042. |
| Detection Method | three-dimensional-structure |
| Source | BIND |
  ||


---

|  |  |
| --- | --- |
 Reference:3 || PMID | 10356396 |
| SourceID | 229137 |
| Species | Human |
| Experimental Condition | in-vitro |
| Description | The three dimensional structure of Gelatinase A (Full-Length) was determined by crystallography. The crystal was grown by using Gelatinase A, pH 7.8, at 100K. X-ray data were collected using synchrotron radiation and were 99.7% complete to a resolution limit of 2.800 angstroms and with an overall Rsym of 11.7%. This crystal belonged to the space group I 41 2 2. The structure was determined using Molecular Replacement and the modeled structure had an R-free = 32.7%. PDB: 1CK7, MMDB: 10832. |
| Detection Method | three-dimensional-structure |
| Source | BIND |
  ||


---

|  |  |
| --- | --- |
 Reference:4 || SourceID | 229985 |
| Experimental Condition | in-vitro |
| Description | The three dimensional structure of Gelatinase A Catalytic Domain was determined by crystallography. The crystal was grown by using Gelatinase A, pH 6.0, at 293K. X-ray data were collected using synchrotron radiation and were 99.5% complete to a resolution limit of 2.800 angstroms and with an overall Rsym of 13.0%. This crystal contained 1 chain per asymmetric unit and belonged to the space group P 41 2 2. The structure was determined using Molecular Replacement and the modeled structure was refined to an R-factor = 20.4% and had an R-free = 24.4%. PDB: 1QIB, MMDB: 11540. |
| Detection Method | three-dimensional-structure |
| Source | BIND |
  ||


---

|  |  |
| --- | --- |
 Reference:5 || SourceID | 229986 |
| Experimental Condition | in-vitro |
| Description | The three dimensional structure of Gelatinase A Catalytic Domain was determined by crystallography. The crystal was grown by using Gelatinase A, pH 6.0, at 293K. X-ray data were collected using synchrotron radiation and were 99.5% complete to a resolution limit of 2.800 angstroms and with an overall Rsym of 13.0%. This crystal contained 1 chain per asymmetric unit and belonged to the space group P 41 2 2. The structure was determined using Molecular Replacement and the modeled structure was refined to an R-factor = 20.4% and had an R-free = 24.4%. PDB: 1QIB, MMDB: 11540. |
| Detection Method | three-dimensional-structure |
| Source | BIND |
  ||


---

|  |  |
| --- | --- |
 Reference:6 || SourceID | 229987 |
| Experimental Condition | in-vitro |
| Description | The three dimensional structure of Gelatinase A Catalytic Domain was determined by crystallography. The crystal was grown by using Gelatinase A, pH 6.0, at 293K. X-ray data were collected using synchrotron radiation and were 99.5% complete to a resolution limit of 2.800 angstroms and with an overall Rsym of 13.0%. This crystal contained 1 chain per asymmetric unit and belonged to the space group P 41 2 2. The structure was determined using Molecular Replacement and the modeled structure was refined to an R-factor = 20.4% and had an R-free = 24.4%. PDB: 1QIB, MMDB: 11540. |
| Detection Method | three-dimensional-structure |
| Source | BIND |
  ||


---

|  |  |
| --- | --- |
 Reference:7 || PMID | 12077439 |
| SourceID | 241548 |
| Species | Human |
| Experimental Condition | in-vitro |
| Description | The three dimensional structure of Human Matrix Metalloproteinase Mmp9 (Gelatinase B) was determined by crystallography. The crystal was grown by using Matrix Metalloproteinase-9, Sodium Chloride, Tris, HEPES pH 8.2, X-ray data were collected using synchrotron radiation and were 89.1% complete to a resolution limit of 2.500 angstroms with an overall Rsym of 7.7%. This crystal contained 1 chain per asymmetric unit and belonged to the space group P 65. The structure was determined using MAD and the modeled structure was refined to an R-factor = 18.7% and had an R-free = 23.0%. PDB: 1L6J, MMDB: 19924. |
| Detection Method | three-dimensional-structure |
| Source | BIND |
  ||


---

|  |  |
| --- | --- |
 Reference:8 || PMID | 12077439 |
| SourceID | 241550 |
| Species | Human |
| Experimental Condition | in-vitro |
| Description | The three dimensional structure of Human Matrix Metalloproteinase Mmp9 (Gelatinase B) was determined by crystallography. The crystal was grown by using Matrix Metalloproteinase-9, Sodium Chloride, Tris, HEPES pH 8.2, X-ray data were collected using synchrotron radiation and were 89.1% complete to a resolution limit of 2.500 angstroms with an overall Rsym of 7.7%. This crystal contained 1 chain per asymmetric unit and belonged to the space group P 65. The structure was determined using MAD and the modeled structure was refined to an R-factor = 18.7% and had an R-free = 23.0%. PDB: 1L6J, MMDB: 19924. |
| Detection Method | three-dimensional-structure |
| Source | BIND |
  ||


---

|  |  |
| --- | --- |
 Reference:9 || SourceID | 242774 |
| Experimental Condition | in-vitro |
| Description | The three dimensional structure of Catalytic Domain of Prommp-2 E404q Mutant was determined by crystallography. The crystal was grown by using 72 Kda Type IV Collagenase, Inhibitor Peptide, pH 8.5, at 100K. X-ray data were collected using synchrotron radiation and were 93.7% complete to a resolution limit of 2.65 angstroms with an overall Rsym of 9.3%. This crystal contained 4 chains per asymmetric unit and belonged to the space group P 21 21 21. The structure was determined using Molecular Replacement and the modeled structure was refined to an R-factor = 27.1% and had an R-free = 30.3%. PDB: 1EAK, MMDB: 20323. |
| Detection Method | three-dimensional-structure |
| Source | BIND |
  |


---

|  |  |
| --- | --- |
