# Supplementary material for: Dynamic transcriptomic profiles of zebrafish gills in response to zinc supplementation
Source: BMC Genomics. 2010 Oct 11;11:553. doi: 10.1186/1471-2164-11-553 (PMC3091702; doi:10.1186/1471-2164-11-553)
Supplement: Additional file 2 — Interactive Direct Interaction Network representing the molecular interactions between zinc, copper, iron, calcium and proteins encoded by transcripts changed by zinc supplementation. Mini web-site containing index.html and hyperlinked pages in subdirectory describing a Direct Interaction Network automatically generated based on curated interactions contained within the proprietary PathwayArchitect database. Ovals represent proteins and the circles symbolize metal ions. Objects are coloured by their abundance in zebrafish at the time-point they were significantly different from the control is a scale from -4 fold (dark green) to +4 fold (dark red). Where significant differences were found at more than one time-point, the colour overlay shows expression at the first instance. Dark blue squares denote 'binding', and light blue squares 'expression'; green squares stand for 'regulation', green diamonds for 'metabolism', and green circles for 'promoter binding'. Arrow heads indicate directionality of the interaction where annotated. All nodes and edges can be further interrogated by selecting the relative area of the image. [file 1471-2164-11-553-S2.zip › PathwayArchitect Zn xs DIN/1022478.html]

# REGULATION:

|  |  |
| --- | --- |
| Type | REGULATION |
| Effect | None |


---

|  |  |
| --- | --- |
| Score | 0 |


---

|  |  |
| --- | --- |
| Reference Count | 4 |


---

|  |  |
| --- | --- |
| Mechanism | Unknown |


---

|  |  |
| --- | --- |
| Reference:0 || Sentence | "This analysis has focused on cation transporter gene families for which initial characterizations have been achieved for individual members, including potassium transporters and channels, sodium transporters, calcium antiporters, cyclic nucleotide-gated channels, cation diffusion facilitator proteins, natural resistance-associated macrophage proteins (NRAMP), and Zn-regulated transporter Fe-regulated transporter-like proteins." |
| PMID | 11500563 |
| Year | 2001 |
| Journal | Plant Physiol |
| RefScore | 0 |
| Source | PArchNLP |
  |
|


---

|  |  |
| --- | --- |
 Reference:1 || Sentence | PURPOSE: To examine the prospective association of serum iron, copper, and zinc with cancer mortality. |
| Year | 2004 |
| PMID | 15036223 |
| Species | Human |
| Journal | Ann Epidemiol |
| RefScore | 1 |
| Source | PArchNLP |
  ||


---

|  |  |
| --- | --- |
 Reference:2 || Sentence | Plasma zinc indicated positive association with zinc, thiamin and riboflavin intakes (p < 0.05) and SOD showed negative association with iron and family size. |
| PMID | 16192257 |
| Year | 2005 |
| Species | Human |
| Journal | J Am Coll Nutr |
| RefScore | 1 |
| Source | PArchNLP |
  ||


---

|  |  |
| --- | --- |
 Reference:3 || Sentence | During late infancy, both DMT1 and FPN were appropriately localized to the apical and basolateral membrane, respectively, and negative effects of Zn supplementation on Fe absorption were absent. |
| PMID | 16614402 |
| Year | 2006 |
| Species | Rat |
| Journal | J Nutr |
| RefScore | 0 |
| Source | PArchNLP |
  |


---

|  |  |
| --- | --- |
