# Supplementary material for: Dynamic transcriptomic profiles of zebrafish gills in response to zinc supplementation
Source: BMC Genomics. 2010 Oct 11;11:553. doi: 10.1186/1471-2164-11-553 (PMC3091702; doi:10.1186/1471-2164-11-553)
Supplement: Additional file 2 — Interactive Direct Interaction Network representing the molecular interactions between zinc, copper, iron, calcium and proteins encoded by transcripts changed by zinc supplementation. Mini web-site containing index.html and hyperlinked pages in subdirectory describing a Direct Interaction Network automatically generated based on curated interactions contained within the proprietary PathwayArchitect database. Ovals represent proteins and the circles symbolize metal ions. Objects are coloured by their abundance in zebrafish at the time-point they were significantly different from the control is a scale from -4 fold (dark green) to +4 fold (dark red). Where significant differences were found at more than one time-point, the colour overlay shows expression at the first instance. Dark blue squares denote 'binding', and light blue squares 'expression'; green squares stand for 'regulation', green diamonds for 'metabolism', and green circles for 'promoter binding'. Arrow heads indicate directionality of the interaction where annotated. All nodes and edges can be further interrogated by selecting the relative area of the image. [file 1471-2164-11-553-S2.zip › PathwayArchitect Zn xs DIN/1027462.html]

# REGULATION:

|  |  |
| --- | --- |
| Type | REGULATION |
| Effect | None |


---

|  |  |
| --- | --- |
| Score | 0 |


---

|  |  |
| --- | --- |
| Reference Count | 3 |


---

|  |  |
| --- | --- |
| Mechanism | Unknown |


---

|  |  |
| --- | --- |
| Reference:0 || Sentence | "Therefore, Ab binding to Pl reduces specifically the activation energy for a slow transition triggered by Ca2+ binding, with consequent enhancement of overall kinetics under conditions enhancing the rate-limiting contribution of this transition." |
| PMID | 8349590 |
| Year | 1993 |
| Journal | J Biol Chem |
| RefScore | 0 |
| Source | PArchNLP |
  |
|


---

|  |  |
| --- | --- |
 Reference:1 || Sentence | "Gene-targeted or gene-ablated (knockout) mice have been reported for the sarcoplasmic reticulum (SR) Ca2+ pump isoforms SERCA2, SERCA2a and SERCA3, the plasma membrane Ca2+ pump isoforms, PMCA1, PMCA2 and PMCA4, and the SR-associated protein, phospholamban (PLB), an inhibitor of SERCA2." |
| PMID | 12164311 |
| Year | 2002 |
| Species | Mouse |
| Journal | Novartis Found Symp |
| RefScore | 1 |
| Source | PArchNLP |
  ||


---

|  |  |
| --- | --- |
 Reference:2 || Sentence | Mechanistically, PKCalpha directly regulates Ca2+ handling by altering the phosphorylation status of inhibitor-1, which in turn suppresses protein phosphatase-1 activity, thus modulating phospholamban activity and secondarily, the sarcoplasmic reticulum Ca2+ ATPase. |
| PMID | 16880328 |
| Year | 2006 |
| Species | Mouse |
|  | Rat |
| Journal | Circulation |
| RefScore | 2 |
| Source | PArchNLP |
  |


---

|  |  |
| --- | --- |
