# Supplementary material for: Dynamic transcriptomic profiles of zebrafish gills in response to zinc supplementation
Source: BMC Genomics. 2010 Oct 11;11:553. doi: 10.1186/1471-2164-11-553 (PMC3091702; doi:10.1186/1471-2164-11-553)
Supplement: Additional file 2 — Interactive Direct Interaction Network representing the molecular interactions between zinc, copper, iron, calcium and proteins encoded by transcripts changed by zinc supplementation. Mini web-site containing index.html and hyperlinked pages in subdirectory describing a Direct Interaction Network automatically generated based on curated interactions contained within the proprietary PathwayArchitect database. Ovals represent proteins and the circles symbolize metal ions. Objects are coloured by their abundance in zebrafish at the time-point they were significantly different from the control is a scale from -4 fold (dark green) to +4 fold (dark red). Where significant differences were found at more than one time-point, the colour overlay shows expression at the first instance. Dark blue squares denote 'binding', and light blue squares 'expression'; green squares stand for 'regulation', green diamonds for 'metabolism', and green circles for 'promoter binding'. Arrow heads indicate directionality of the interaction where annotated. All nodes and edges can be further interrogated by selecting the relative area of the image. [file 1471-2164-11-553-S2.zip › PathwayArchitect Zn xs DIN/103255.html]

# PROTEIN: XRCC5

|  |  |
| --- | --- |
| Name | XRCC5 |
| Type | PROTEIN |
| Description | X-ray repair complementing defective repair in Chinese hamster cells 5 (double-strand-break rejoining; Ku autoantigen, 80kDa) |
| Note | The protein encoded by this gene is the 80-kilodalton subunit of the Ku heterodimer protein which is also known as ATP-dependant DNA helicase II or DNA repair protein XRCC5. Ku is the DNA-binding component of the DNA-dependent protein kinase, and it functions together with the DNA ligase IV-XRCC4 complex in the repair of DNA double-strand break by non-homologous end joining and the completion of V(D)J recombination events. This gene functionally complements Chinese hamster xrs-6, a mutant defective in DNA double-strand break repair and in ability to undergo V(D)J recombination. A rare microsatellite polymorphism in this gene is associated with cancer in patients of varying radiosensitivity. |
| Alias | Ku80 |
|  | X-ray repair cross complementation (double-strand-break rejoining; Ku autoantigen, 80kD) |
|  | Ku autoantigen protein p86 homolog |
|  | CTC85 |
|  | CTC box binding factor 85 kDa subunit |
|  | Ku p80 |
|  | X-ray repair, complementing defective, repair in Chinese hamster |
|  | Ku86 |
|  | CTCBF |
|  | Nuclear factor IV |
|  | Xrcc5 |
|  | Kup80 |
|  | KARP1 |
|  | Ku86 autoantigen related protein 1 |
|  | KARP-1 |
|  | NFIV |
|  | X-ray repair complementing defective repair in Chinese hamster cells 5 (double-strand-break rejoining; Ku autoantigen, 80kD) |
|  | KU80 |
|  | Ku autoantigen |
|  | G22p2 |
|  | AI314015 |
|  | DNA repair protein XRCC5 |
|  | ATP-dependent DNA helicase II |
|  | DNA-repair protein XRCC5 |


---

|  |  |
| --- | --- |
| GO Component | DNA-dependent protein kinase complex |
|  | nucleus |
|  | cytoplasm |


---

|  |  |
| --- | --- |
| GO ID | GO:0005634 |
|  | GO:0003677 |
|  | GO:0006281 |
|  | GO:0005524 |
|  | GO:0004386 |
|  | GO:0003690 |
|  | GO:0005737 |
|  | GO:0004003 |
|  | GO:0006302 |
|  | GO:0005958 |
|  | GO:0005515 |
|  | GO:0016787 |
|  | GO:0000166 |
|  | GO:0006310 |
|  | GO:0006303 |
|  | GO:0006282 |


---

|  |  |
| --- | --- |
| MIM | MIM:194364 |


---

|  |  |
| --- | --- |
| Connectivity | 165 |


---

|  |  |
| --- | --- |
| Entrez ID | 363247 |
|  | 22596 |
|  | 7520 |


---

|  |  |
| --- | --- |
| Agilent ID | A\_14\_P100941 |
|  | A\_53\_P132505 |
|  | A\_53\_P105058 |
|  | A\_23\_P79738 |
|  | A\_51\_P201661 |
|  | A\_24\_P345498 |
|  | A\_14\_P111005 |
|  | A\_14\_P105832 |
|  | A\_44\_P264460 |
|  | A\_43\_P15672 |


---

|  |  |
| --- | --- |
| Cellular Localization | Nucleus |
|  | Cytoplasm |
|  | Organelle |
|  | Cell |


---

|  |  |
| --- | --- |
| DbXref | Reactome##73894##DNA Repair##http://www.reactome.org/cgi-bin/eventbrowser?DB=gk\_current&ID=73894 |


---

|  |  |
| --- | --- |
| Pathway | Zn xs inventory |
|  | Zn xs DIN |


---

|  |  |
| --- | --- |
| GO Process | DNA repair |
|  | double-strand break repair |
|  | regulation of DNA repair |
|  | DNA recombination |
|  | double-strand break repair via nonhomologous end-joining |


---

|  |  |
| --- | --- |
| UniGene | Hs.388739 |
|  | Mm.246952 |
|  | Rn.52078 |


---

|  |  |
| --- | --- |
| Affymetrix Probeset ID | 1370931\_at |
|  | 1451968\_at |
|  | 161686\_i\_at |
|  | 208642\_s\_at |
|  | 208643\_s\_at |
|  | 2093\_s\_at |
|  | 232633\_at |
|  | 233007\_at |
|  | 243460\_at |
|  | 38733\_at |
|  | 42521\_at |
|  | 584\_s\_at |
|  | 585\_at |
|  | 72814\_at |
|  | 75171\_at |
|  | 81611\_at |
|  | 86290\_r\_at |
|  | 86761\_at |
|  | 96063\_at |
|  | g186791\_3p\_a\_at |
|  | Hs.240075.0.S1\_3p\_at |
|  | Hs.257082.0.A1\_3p\_at |
|  | Hs.293923.0.S1\_3p\_at |
|  | Hs.84981.0.S1\_3p\_a\_at |
|  | M30938\_at |
|  | Msa.13085.0\_f\_at |
|  | rc\_AA893188\_at |
|  | X66323\_s\_at |
|  | rc\_AI059772\_at |
|  | 97590\_i\_at |
|  | 97591\_r\_at |
|  | RC\_T70070\_at |
|  | TC21003\_f\_at |


---

|  |  |
| --- | --- |
| GO Function | hydrolase activity |
|  | helicase activity |
|  | DNA binding |
|  | nucleotide binding |
|  | ATP binding |
|  | ATP-dependent DNA helicase activity |
|  | protein binding |
|  | double-stranded DNA binding |


---

|  |  |
| --- | --- |
| Nucleotide | BC061576 |
|  | AK167312 |
|  | BC095442 |
|  | AK026166 |
|  | BC029218 |
|  | AF039597 |
|  | AK169838 |
|  | AK089944 |
|  | AK165470 |
|  | BC051660 |
|  | AK182445 |
|  | AC010686 |
|  | NM\_009533 |
|  | X57500 |
|  | NM\_177419 |
|  | AB066103 |
|  | NM\_021141 |
|  | J04977 |
|  | AK010416 |
|  | AK177313 |
|  | AK084263 |
|  | BC019027 |
|  | X66323 |
|  | AF166486 |
|  | AK081633 |
|  | AK199633 |
|  | AK222603 |
|  | M30938 |
|  | AK168913 |


---

|  |  |
| --- | --- |
| Protein | AAH19027 |
|  | BAB26920 |
|  | BAE41402 |
|  | BAC38276 |
|  | AAH51660 |
|  | BAD96323 |
|  | BAE38207 |
|  | AAA59475 |
|  | NP\_033559 |
|  | AAC52087 |
|  | AAH95442 |
|  | CAA40736 |
|  | BAE40726 |
|  | AAH61576 |
|  | AAY14659 |
|  | AAD49720 |
|  | BAB83859 |
|  | NP\_066964 |
|  | P27641 |
|  | NP\_803154 |
|  | BAE39415 |
|  | P13010 |
|  | CAA46999 |
|  | AAH29218 |
|  | AAA36154 |


---

|  |  |
| --- | --- |
| Organism | Mammal |


---

|  |  |
| --- | --- |
| Location | chromosome 2, 2q35 (Homo sapiens) |
|  | chromosome 1, 1 42.0 cM, 1 E (Mus musculus) |
|  | chromosome 9, 9q34 (Rattus norvegicus) |
|  | 1 42.0 cM (Mus musculus) |


---

|  |  |
| --- | --- |
