# Supplementary material for: Dynamic transcriptomic profiles of zebrafish gills in response to zinc supplementation
Source: BMC Genomics. 2010 Oct 11;11:553. doi: 10.1186/1471-2164-11-553 (PMC3091702; doi:10.1186/1471-2164-11-553)
Supplement: Additional file 2 — Interactive Direct Interaction Network representing the molecular interactions between zinc, copper, iron, calcium and proteins encoded by transcripts changed by zinc supplementation. Mini web-site containing index.html and hyperlinked pages in subdirectory describing a Direct Interaction Network automatically generated based on curated interactions contained within the proprietary PathwayArchitect database. Ovals represent proteins and the circles symbolize metal ions. Objects are coloured by their abundance in zebrafish at the time-point they were significantly different from the control is a scale from -4 fold (dark green) to +4 fold (dark red). Where significant differences were found at more than one time-point, the colour overlay shows expression at the first instance. Dark blue squares denote 'binding', and light blue squares 'expression'; green squares stand for 'regulation', green diamonds for 'metabolism', and green circles for 'promoter binding'. Arrow heads indicate directionality of the interaction where annotated. All nodes and edges can be further interrogated by selecting the relative area of the image. [file 1471-2164-11-553-S2.zip › PathwayArchitect Zn xs DIN/104801.html]

# PROTEIN: EFHC1

|  |  |
| --- | --- |
| Name | EFHC1 |
| Type | PROTEIN |
| Description | EF-hand domain (C-terminal) containing 1 |
| Alias | FLJ10466 |
|  | EJM1 |
|  | EF-hand domain containing 1 |
|  | dJ304B14.2 |
|  | mRib72-1 |
|  | 1700029F22Rik |


---

|  |  |
| --- | --- |
| GO Component | flagellum |
|  | cilium |


---

|  |  |
| --- | --- |
| GO ID | GO:0005929 |
|  | GO:0005509 |
|  | GO:0019861 |


---

|  |  |
| --- | --- |
| MIM | MIM:608815 |
|  | MIM:254770 |


---

|  |  |
| --- | --- |
| Connectivity | 4 |


---

|  |  |
| --- | --- |
| Entrez ID | 114327 |
|  | 71877 |


---

|  |  |
| --- | --- |
| Agilent ID | A\_32\_P86242 |
|  | A\_14\_P129987 |
|  | A\_51\_P440327 |
|  | A\_14\_P118897 |
|  | A\_23\_P214291 |
|  | A\_32\_P86245 |


---

|  |  |
| --- | --- |
| Cellular Localization | Flagellum |
|  | Cilium |
|  | Cell projection |
|  | Cell |
|  | Organelle |


---

|  |  |
| --- | --- |
| Pathway | Zn xs inventory |
|  | Zn xs DIN |


---

|  |  |
| --- | --- |
| UniGene | Mm.29178 |
|  | Hs.403171 |


---

|  |  |
| --- | --- |
| Affymetrix Probeset ID | 115277\_at |
|  | 1453159\_at |
|  | 219833\_s\_at |
|  | 225656\_at |
|  | 231026\_at |
|  | 60750\_at |
|  | 61217\_at |
|  | 74121\_at |
|  | 78489\_at |
|  | 84070\_at |
|  | 88690\_at |
|  | g8922435\_3p\_a\_at |
|  | Hs.121073.1.S1\_3p\_at |
|  | Hs.160927.0.A1\_3p\_at |


---

|  |  |
| --- | --- |
| GO Function | calcium ion binding |


---

|  |  |
| --- | --- |
| Nucleotide | AK001328 |
|  | AI413430 |
|  | AL136125 |
|  | AY608689 |
|  | BC020210 |
|  | AL122084 |
|  | XM\_129694 |
|  | BC012921 |
|  | NM\_018100 |
|  | AK006489 |
|  | AL049611 |


---

|  |  |
| --- | --- |
| Protein | XP\_129694 |
|  | AAH12921 |
|  | AAT67418 |
|  | Q9D9T8 |
|  | BAB24614 |
|  | BAA91628 |
|  | CAI19693 |
|  | AAH20210 |
|  | CAI20107 |
|  | NP\_060570 |


---

|  |  |
| --- | --- |
| Organism | Mammal |


---

|  |  |
| --- | --- |
| Location | chromosome 6, 6p12.3 (Homo sapiens) |
|  | chromosome 1, 1 A5 (Mus musculus) |


---

|  |  |
| --- | --- |
