# Supplementary material for: Dynamic transcriptomic profiles of zebrafish gills in response to zinc supplementation
Source: BMC Genomics. 2010 Oct 11;11:553. doi: 10.1186/1471-2164-11-553 (PMC3091702; doi:10.1186/1471-2164-11-553)
Supplement: Additional file 2 — Interactive Direct Interaction Network representing the molecular interactions between zinc, copper, iron, calcium and proteins encoded by transcripts changed by zinc supplementation. Mini web-site containing index.html and hyperlinked pages in subdirectory describing a Direct Interaction Network automatically generated based on curated interactions contained within the proprietary PathwayArchitect database. Ovals represent proteins and the circles symbolize metal ions. Objects are coloured by their abundance in zebrafish at the time-point they were significantly different from the control is a scale from -4 fold (dark green) to +4 fold (dark red). Where significant differences were found at more than one time-point, the colour overlay shows expression at the first instance. Dark blue squares denote 'binding', and light blue squares 'expression'; green squares stand for 'regulation', green diamonds for 'metabolism', and green circles for 'promoter binding'. Arrow heads indicate directionality of the interaction where annotated. All nodes and edges can be further interrogated by selecting the relative area of the image. [file 1471-2164-11-553-S2.zip › PathwayArchitect Zn xs DIN/105179.html]

# PROTEIN: CAPN5

|  |  |
| --- | --- |
| Name | CAPN5 |
| Type | PROTEIN |
| Description | calpain 5 |
| Note | Calpains are calcium-dependent cysteine proteases involved in signal transduction in a variety of cellular processes. A functional calpain protein consists of an invariant small subunit and 1 of a family of large subunits. CAPN5 is one of the large subunits. Unlike some of the calpains, CAPN5 and CAPN6 lack a calmodulin-like domain IV. Because of the significant similarity to Caenorhabditis elegans sex determination gene tra-3, CAPN5 is also called as HTRA3. Multiple alternatively spliced transcript variants have been found for this gene, but the full-length nature of some variants has not been defined. |
| Alias | Capn5 |
|  | high-temperature requirement factor A3 |
|  | FLJ46245 |
|  | nCL-3 |
|  | Htra3 |
|  | HTRA3 |


---

|  |  |
| --- | --- |
| GO Component | intracellular |


---

|  |  |
| --- | --- |
| GO ID | GO:0004198 |
|  | GO:0008233 |
|  | GO:0007165 |
|  | GO:0005622 |
|  | GO:0008219 |
|  | GO:0006508 |
|  | GO:0016787 |
|  | GO:0008234 |
|  | GO:0004197 |


---

|  |  |
| --- | --- |
| MIM | MIM:602537 |


---

|  |  |
| --- | --- |
| Connectivity | 339 |


---

|  |  |
| --- | --- |
| Entrez ID | 12337 |
|  | 171495 |
|  | 726 |


---

|  |  |
| --- | --- |
| Agilent ID | A\_53\_P176348 |
|  | A\_53\_P125259 |
|  | A\_53\_P125583 |
|  | A\_52\_P683441 |
|  | A\_23\_P105197 |
|  | A\_14\_P118609 |
|  | A\_14\_P119579 |
|  | A\_51\_P375111 |
|  | A\_43\_P15397 |
|  | A\_53\_P161861 |


---

|  |  |
| --- | --- |
| Cellular Localization | Cell |


---

|  |  |
| --- | --- |
| DbXref | KEGG pathway##04210##Apoptosis##http://www.genome.jp/dbget-bin/show\_pathway?rno04210+171495 |
|  | KEGG pathway##04210##Apoptosis##http://www.genome.jp/dbget-bin/show\_pathway?hsa04210+726 |
|  | KEGG pathway##04210##Apoptosis##http://www.genome.jp/dbget-bin/show\_pathway?mmu04210+12337 |
|  | KEGG pathway##04510##Focal adhesion##http://www.genome.jp/dbget-bin/show\_pathway?mmu04510+12337 |
|  | KEGG pathway##04510##Focal adhesion##http://www.genome.jp/dbget-bin/show\_pathway?hsa04510+726 |


---

|  |  |
| --- | --- |
| Pathway | Zn xs inventory |
|  | Zn xs DIN |


---

|  |  |
| --- | --- |
| GO Process | proteolysis and peptidolysis |
|  | signal transduction |
|  | proteolysis |
|  | cell death |


---

|  |  |
| --- | --- |
| UniGene | Rn.15526 |
|  | Hs.248153 |
|  | Mm.326847 |


---

|  |  |
| --- | --- |
| Affymetrix Probeset ID | 102316\_at |
|  | 109830\_at |
|  | 1369441\_at |
|  | 1384532\_at |
|  | 1385851\_at |
|  | 1418671\_at |
|  | 1449165\_at |
|  | 205166\_at |
|  | 226292\_at |
|  | 38504\_at |
|  | 53942\_at |
|  | 78506\_at |
|  | g6552324\_3p\_at |
|  | Hs.6133.1.S1\_3p\_at |
|  | rc\_AA925441\_at |
|  | Y10656\_s\_at |
|  | RC\_H81543\_at |
|  | RC\_Z39842\_at |
|  | TC15986\_at |
|  | TC20174\_at |
|  | TC30215\_at |
|  | TC30215\_g\_at |


---

|  |  |
| --- | --- |
| EC Number | EC 3.4.22.- |


---

|  |  |
| --- | --- |
| GO Function | hydrolase activity |
|  | peptidase activity |
|  | cysteine-type peptidase activity |
|  | cysteine-type endopeptidase activity |
|  | calpain activity |


---

|  |  |
| --- | --- |
| Nucleotide | AK129588 |
|  | BC018123 |
|  | U94346 |
|  | AK092880 |
|  | Y10656 |
|  | AF484958 |
|  | BM679546 |
|  | AK164293 |
|  | NM\_134461 |
|  | AK094150 |
|  | AK139476 |
|  | NM\_004055 |
|  | AK128124 |
|  | A91669 |
|  | NM\_007602 |
|  | AB209087 |
|  | BC014767 |
|  | Y10552 |
|  | AK170034 |
|  | U85020 |
|  | AK155179 |


---

|  |  |
| --- | --- |
| Protein | NP\_604456 |
|  | AAL92024 |
|  | AAD00559 |
|  | BAE24027 |
|  | BAD92324 |
|  | BAE41522 |
|  | AAH14767 |
|  | AAH18123 |
|  | BAC85188 |
|  | Q8R4C0 |
|  | BAC87282 |
|  | BAE33097 |
|  | CAB69426 |
|  | O15484 |
|  | NP\_004046 |
|  | O08688 |
|  | CAA71666 |
|  | CAA71584 |
|  | AAC51869 |
|  | BAE37722 |
|  | NP\_031628 |


---

|  |  |
| --- | --- |
| Organism | Mammal |


---

|  |  |
| --- | --- |
| Location | chromosome 7, 7 E1 (Mus musculus) |
|  | chromosome 11, 11q14 (Homo sapiens) |
|  | chromosome 1, 1q32 (Rattus norvegicus) |


---

|  |  |
| --- | --- |
