# Supplementary material for: Dynamic transcriptomic profiles of zebrafish gills in response to zinc supplementation
Source: BMC Genomics. 2010 Oct 11;11:553. doi: 10.1186/1471-2164-11-553 (PMC3091702; doi:10.1186/1471-2164-11-553)
Supplement: Additional file 2 — Interactive Direct Interaction Network representing the molecular interactions between zinc, copper, iron, calcium and proteins encoded by transcripts changed by zinc supplementation. Mini web-site containing index.html and hyperlinked pages in subdirectory describing a Direct Interaction Network automatically generated based on curated interactions contained within the proprietary PathwayArchitect database. Ovals represent proteins and the circles symbolize metal ions. Objects are coloured by their abundance in zebrafish at the time-point they were significantly different from the control is a scale from -4 fold (dark green) to +4 fold (dark red). Where significant differences were found at more than one time-point, the colour overlay shows expression at the first instance. Dark blue squares denote 'binding', and light blue squares 'expression'; green squares stand for 'regulation', green diamonds for 'metabolism', and green circles for 'promoter binding'. Arrow heads indicate directionality of the interaction where annotated. All nodes and edges can be further interrogated by selecting the relative area of the image. [file 1471-2164-11-553-S2.zip › PathwayArchitect Zn xs DIN/105738.html]

# PROTEIN: PABPC1

|  |  |
| --- | --- |
| Name | PABPC1 |
| Type | PROTEIN |
| Description | poly(A) binding protein, cytoplasmic 1 |
| Note | The poly(A)-binding protein (PABP), which is found complexed to the 3-prime poly(A) tail of eukaryotic mRNA, is required for poly(A) shortening and translation initiation. In humans, the PABPs comprise a small nuclear isoform and a conserved gene family that displays at least 3 functional proteins: PABP1 (PABPC1), inducible PABP (iPABP, or PABPC4; MIM 603407), and PABP3 (PABPC3; MIM 604680). In addition, there are at least 4 pseudogenes, PABPCP1 to PABPCP4.[supplied by OMIM] |
| Alias | PABP1 |
|  | Pabp1 |
|  | MGC91496 |
|  | PABP 1 |
|  | poly A binding protein 1 |
|  | Pabp |
|  | PABPC2 |
|  | PABPL1 |
|  | Pabpc1 |
|  | PabpI |
|  | PAB1 |
|  | Poly(A)-binding protein 1 |
|  | poly(A)-binding protein, cytoplasmic 2 |
|  | PABP |
|  | poly(A) binding protein, cytoplasmic 2 |
|  | Pabpl1 |
|  | PABPC1 |


---

|  |  |
| --- | --- |
| GO Component | cytoplasm |


---

|  |  |
| --- | --- |
| GO ID | GO:0008143 |
|  | GO:0003729 |
|  | GO:0003723 |
|  | GO:0045946 |
|  | GO:0006378 |
|  | GO:0005737 |
|  | GO:0003676 |
|  | GO:0005515 |
|  | GO:0000166 |
|  | GO:0008494 |
|  | GO:0048255 |


---

|  |  |
| --- | --- |
| MIM | MIM:604679 |


---

|  |  |
| --- | --- |
| Connectivity | 82 |


---

|  |  |
| --- | --- |
| Entrez ID | 18458 |
|  | 171350 |
|  | 26986 |


---

|  |  |
| --- | --- |
| Agilent ID | A\_53\_P152392 |
|  | A\_51\_P119106 |
|  | A\_43\_P13245 |
|  | A\_23\_P82693 |
|  | A\_53\_P133957 |
|  | A\_32\_P152673 |
|  | A\_52\_P204023 |
|  | A\_53\_P124901 |
|  | A\_14\_P201438 |
|  | A\_32\_P38136 |
|  | A\_14\_P102899 |
|  | A\_14\_P138619 |


---

|  |  |
| --- | --- |
| Cellular Localization | Cytoplasm |
|  | Cell |


---

|  |  |
| --- | --- |
| DbXref | Reactome##157279##157279##http://www.reactome.org/cgi-bin/eventbrowser?DB=gk\_current&ID=157279 |
|  | Reactome##74160##Gene Expression##http://www.reactome.org/cgi-bin/eventbrowser?DB=gk\_current&ID=74160 |


---

|  |  |
| --- | --- |
| Pathway | Zn xs inventory |
|  | Zn xs DIN |


---

|  |  |
| --- | --- |
| GO Process | positive regulation of translation |
|  | mRNA polyadenylylation |
|  | mRNA stabilization |


---

|  |  |
| --- | --- |
| UniGene | Mm.321828 |
|  | Mm.371570 |
|  | Hs.387804 |
|  | Rn.127813 |


---

|  |  |
| --- | --- |
| Affymetrix Probeset ID | 100720\_at |
|  | 131455\_at |
|  | 1369952\_at |
|  | 1379728\_at |
|  | 1418883\_a\_at |
|  | 1453840\_at |
|  | 1560770\_at |
|  | 215157\_3p\_x\_at |
|  | 215157\_x\_at |
|  | 215823\_x\_at |
|  | 236907\_at |
|  | 31950\_at |
|  | 31951\_s\_at |
|  | 44806\_at |
|  | 59809\_f\_at |
|  | 64962\_r\_at |
|  | 1391105\_at |
|  | 82682\_at |
|  | Hs.144923.0.A1\_3p\_at |
|  | Hs.172182.2.A1\_3p\_s\_at |
|  | Hs2.351295.1.S1\_3p\_at |
|  | Hs.283767.0.S1\_3p\_x\_at |
|  | Msa.2609.0\_f\_at |
|  | Msa.32108.0\_s\_at |
|  | rc\_AA899197\_at |
|  | U68105\_s\_at |
|  | X65553\_f\_at |
|  | Z48501\_s\_at |
|  | 1559509\_at |
|  | Hs2.378497.1.A1\_3p\_at |
|  | Msa.22333.0\_s\_at |
|  | 1380405\_at |
|  | R66920\_at |
|  | RC\_H98809\_at |
|  | TC16723\_at |
|  | TC30867\_at |
|  | TC30868\_f\_at |
|  | TC32243\_at |
|  | TC35459\_at |
|  | TC41032\_at |
|  | TC41920\_f\_at |
|  | rc\_AI059569\_at |


---

|  |  |
| --- | --- |
| GO Function | protein binding |
|  | mRNA binding |
|  | RNA binding |
|  | translation activator activity |
|  | poly(A) binding |
|  | nucleotide binding |
|  | nucleic acid binding |


---

|  |  |
| --- | --- |
| Nucleotide | AK212193 |
|  | AK133196 |
|  | NM\_008774 |
|  | BC046233 |
|  | AK159703 |
|  | BC003928 |
|  | X65553 |
|  | U68104 |
|  | BC023145 |
|  | BC083176 |
|  | AK168466 |
|  | BC004587 |
|  | BC003870 |
|  | NM\_134353 |
|  | AK197858 |
|  | BC011207 |
|  | AK095194 |
|  | AK160883 |
|  | AK195565 |
|  | BC023217 |
|  | AK199222 |
|  | NM\_002568 |
|  | AK005009 |
|  | AJ298278 |
|  | AK161671 |
|  | AK161123 |
|  | Z48501 |
|  | AK089747 |
|  | AK214914 |
|  | AJ279847 |
|  | BC041863 |
|  | Y00345 |
|  | BC015958 |
|  | AK160968 |
|  | AK159733 |
|  | AK152064 |
|  | AK044829 |
|  | AK130523 |
|  | BC023520 |
|  | AK150839 |


---

|  |  |
| --- | --- |
| Protein | AAH23145 |
|  | CAC21554 |
|  | CAA68428 |
|  | AAH15958 |
|  | BAC40951 |
|  | CAA88401 |
|  | AAH03870 |
|  | BAE21553 |
|  | AAH04587 |
|  | BAE36203 |
|  | AAH11207 |
|  | BAE35327 |
|  | P11940 |
|  | P29341 |
|  | AAD08718 |
|  | BAE36522 |
|  | BAB23742 |
|  | AAH46233 |
|  | BAE35302 |
|  | AAH23520 |
|  | BAC32110 |
|  | NP\_032800 |
|  | BAE30919 |
|  | AAH41863 |
|  | AAH83176 |
|  | Q9EPH8 |
|  | BAE36120 |
|  | CAA46522 |
|  | NP\_002559 |
|  | BAE40360 |
|  | NP\_599180 |


---

|  |  |
| --- | --- |
| Organism | Mammal |


---

|  |  |
| --- | --- |
| Location | chromosome 8, 8q22.2-q23 (Homo sapiens) |
|  | chromosome 15, 15 C (Mus musculus) |
|  | chromosome 7, 7q22 (Rattus norvegicus) |


---

|  |  |
| --- | --- |
