# Supplementary material for: Dynamic transcriptomic profiles of zebrafish gills in response to zinc supplementation
Source: BMC Genomics. 2010 Oct 11;11:553. doi: 10.1186/1471-2164-11-553 (PMC3091702; doi:10.1186/1471-2164-11-553)
Supplement: Additional file 2 — Interactive Direct Interaction Network representing the molecular interactions between zinc, copper, iron, calcium and proteins encoded by transcripts changed by zinc supplementation. Mini web-site containing index.html and hyperlinked pages in subdirectory describing a Direct Interaction Network automatically generated based on curated interactions contained within the proprietary PathwayArchitect database. Ovals represent proteins and the circles symbolize metal ions. Objects are coloured by their abundance in zebrafish at the time-point they were significantly different from the control is a scale from -4 fold (dark green) to +4 fold (dark red). Where significant differences were found at more than one time-point, the colour overlay shows expression at the first instance. Dark blue squares denote 'binding', and light blue squares 'expression'; green squares stand for 'regulation', green diamonds for 'metabolism', and green circles for 'promoter binding'. Arrow heads indicate directionality of the interaction where annotated. All nodes and edges can be further interrogated by selecting the relative area of the image. [file 1471-2164-11-553-S2.zip › PathwayArchitect Zn xs DIN/107179.html]

# PROTEIN: EFNA5

|  |  |
| --- | --- |
| Name | EFNA5 |
| Type | PROTEIN |
| Description | ephrin-A5 |
| Note | Ephrin-A5, a member of the ephrin gene family, prevents axon bundling in cocultures of cortical neurons with astrocytes, a model of late stage nervous system development and differentiation. The EPH and EPH-related receptors comprise the largest subfamily of receptor protein-tyrosine kinases and have been implicated in mediating developmental events, particularly in the nervous system. EPH receptors typically have a single kinase domain and an extracellular region containing a Cys-rich domain and 2 fibronectin type III repeats. The ephrin ligands and receptors have been named by the Eph Nomenclature Committee (1997). Based on their structures and sequence relationships, ephrins are divided into the ephrin-A (EFNA) class, which are anchored to the membrane by a glycosylphosphatidylinositol linkage, and the ephrin-B (EFNB) class, which are transmembrane proteins. The Eph family of receptors are similarly divided into 2 groups based on the similarity of their extracellular domain sequences and their affinities for binding ephrin-A and ephrin-B ligands. |
| Alias | AL-1 |
|  | Epl7 |
|  | AV158822 |
|  | RAGS |
|  | LERK7 |
|  | EPH-related receptor tyrosine kinase ligand 7 |
|  | eph-related receptor tyrosine kinase ligand 7 |
|  | EFNA5 |
|  | Eplg7 |
|  | EFL-5 |
|  | Lerk7 |
|  | EPLG7 |
|  | Ephrin-A5 |
|  | LERK-7 |
|  | AF1 |
|  | Efna5 |
|  | EFL5 |


---

|  |  |
| --- | --- |
| GO Component | extracellular space |
|  | anchored to plasma membrane |
|  | membrane |


---

|  |  |
| --- | --- |
| GO ID | GO:0007399 |
|  | GO:0007413 |
|  | GO:0016020 |
|  | GO:0005615 |
|  | GO:0030154 |
|  | GO:0007267 |
|  | GO:0007417 |
|  | GO:0007420 |
|  | GO:0004713 |
|  | GO:0046875 |
|  | GO:0007275 |
|  | GO:0046658 |


---

|  |  |
| --- | --- |
| MIM | MIM:601535 |


---

|  |  |
| --- | --- |
| Connectivity | 60 |


---

|  |  |
| --- | --- |
| Entrez ID | 1946 |
|  | 116683 |
|  | 13640 |


---

|  |  |
| --- | --- |
| Agilent ID | A\_14\_P110004 |
|  | A\_53\_P145617 |
|  | A\_51\_P218009 |
|  | A\_53\_P110401 |
|  | A\_14\_P102734 |
|  | A\_53\_P157625 |
|  | A\_14\_P111716 |
|  | A\_14\_P135896 |
|  | A\_43\_P12976 |
|  | A\_53\_P114516 |
|  | A\_53\_P147408 |
|  | A\_53\_P167582 |
|  | A\_51\_P144319 |
|  | A\_14\_P116001 |
|  | A\_23\_P167497 |
|  | A\_53\_P160381 |


---

|  |  |
| --- | --- |
| Cellular Localization | Membrane |
|  | Cell |
|  | Extracellular region |
|  | Plasma membrane |


---

|  |  |
| --- | --- |
| DbXref | KEGG pathway##04360##Axon guidance##http://www.genome.jp/dbget-bin/show\_pathway?mmu04360+13640 |


---

|  |  |
| --- | --- |
| Pathway | Zn xs inventory |
|  | Zn xs DIN |


---

|  |  |
| --- | --- |
| GO Process | cell differentiation |
|  | cell-cell signaling |
|  | brain development |
|  | central nervous system development |
|  | axonal fasciculation |
|  | neurogenesis |
|  | development |
|  | nervous system development |


---

|  |  |
| --- | --- |
| UniGene | Mm.7978 |
|  | Hs.128518 |
|  | Mm.45351 |
|  | Rn.10714 |


---

|  |  |
| --- | --- |
| Affymetrix Probeset ID | 1041\_at |
|  | 117030\_at |
|  | 1369594\_at |
|  | 1385355\_at |
|  | 1391407\_at |
|  | 1391718\_at |
|  | 1421796\_a\_at |
|  | 1436866\_at |
|  | 1440052\_at |
|  | 1446383\_at |
|  | 1460133\_at |
|  | 1559360\_at |
|  | 207301\_at |
|  | 42523\_at |
|  | 47449\_at |
|  | 77389\_at |
|  | 99887\_at |
|  | g4503486\_3p\_at |
|  | U26403\_at |
|  | U69279\_at |
|  | w91777\_at |
|  | 1451930\_at |
|  | 139594\_at |
|  | RC\_F10639\_at |
|  | RC\_H57130\_at |
|  | TC15998\_at |
|  | TC18576\_at |


---

|  |  |
| --- | --- |
| GO Function | ephrin receptor binding |
|  | protein-tyrosine kinase activity |


---

|  |  |
| --- | --- |
| Nucleotide | AK160691 |
|  | AV158822 |
|  | AK050989 |
|  | NM\_010109 |
|  | BC075054 |
|  | U69279 |
|  | BC040218 |
|  | CF579029 |
|  | U26403 |
|  | AK148690 |
|  | NM\_001962 |
|  | NM\_207654 |
|  | NM\_053903 |
|  | BC075055 |
|  | U90664 |
|  | U90665 |


---

|  |  |
| --- | --- |
| Protein | NP\_034239 |
|  | AAH75055 |
|  | P52803 |
|  | AAH75054 |
|  | NP\_446355 |
|  | NP\_001953 |
|  | P97605 |
|  | NP\_997537 |
|  | AAB50240 |
|  | BAE28643 |
|  | AAC05801 |
|  | AAH40218 |
|  | BAC34487 |
|  | O08543 |
|  | AAB60377 |
|  | AAB50239 |


---

|  |  |
| --- | --- |
| Organism | Mammal |


---

|  |  |
| --- | --- |
| Location | chromosome 17, 17 33.5 cM, 17 E1.1 (Mus musculus) |
|  | 17 33.5 cM (Mus musculus) |
|  | chromosome 9, 9q37 (Rattus norvegicus) |
|  | chromosome 5, 5q21 (Homo sapiens) |


---

|  |  |
| --- | --- |
