# Supplementary material for: Dynamic transcriptomic profiles of zebrafish gills in response to zinc supplementation
Source: BMC Genomics. 2010 Oct 11;11:553. doi: 10.1186/1471-2164-11-553 (PMC3091702; doi:10.1186/1471-2164-11-553)
Supplement: Additional file 2 — Interactive Direct Interaction Network representing the molecular interactions between zinc, copper, iron, calcium and proteins encoded by transcripts changed by zinc supplementation. Mini web-site containing index.html and hyperlinked pages in subdirectory describing a Direct Interaction Network automatically generated based on curated interactions contained within the proprietary PathwayArchitect database. Ovals represent proteins and the circles symbolize metal ions. Objects are coloured by their abundance in zebrafish at the time-point they were significantly different from the control is a scale from -4 fold (dark green) to +4 fold (dark red). Where significant differences were found at more than one time-point, the colour overlay shows expression at the first instance. Dark blue squares denote 'binding', and light blue squares 'expression'; green squares stand for 'regulation', green diamonds for 'metabolism', and green circles for 'promoter binding'. Arrow heads indicate directionality of the interaction where annotated. All nodes and edges can be further interrogated by selecting the relative area of the image. [file 1471-2164-11-553-S2.zip › PathwayArchitect Zn xs DIN/108729.html]

# PROTEIN: PPARA

|  |  |
| --- | --- |
| Name | PPARA |
| Type | PROTEIN |
| Description | peroxisome proliferative activated receptor, alpha |
| Note | Peroxisome proliferators include hypolipidemic drugs, herbicides, leukotriene antagonists, and plasticizers; this term arises because they induce an increase in the size and number of peroxisomes. Peroxisomes are subcellular organelles found in plants and animals which contain enzymes for respiration and for cholesterol and lipid metabolism. The action of peroxisome proliferators is thought to be mediated via specific receptors, called PPARs, which belong to the steroid hormone receptor superfamily. PPARs affect the expression of target genes involved in cell proliferation, cell differentiation and in immune and inflammation responses. Three closely related subtypes (alpha, beta/delta, and gamma) have been identified. This gene encodes the subtype PPAR-alpha, which is a nuclear transcription factor. Multiple alternatively spliced transcript variants have been described for this gene, although the full-nature of only some have been defined. |
| Alias | PPAR |
|  | OTTHUMP00000042872 |
|  | hPPAR |
|  | MGC2452 |
|  | OTTHUMP00000028713 |
|  | PPARA |
|  | NR1C1 |
|  | PPAR-alpha |
|  | Ppara |
|  | MGC2237 |
|  | PPARalpha |
|  | Nr1c1 |
|  | Ppar |


---

|  |  |
| --- | --- |
| GO Component | nucleus |


---

|  |  |
| --- | --- |
| GO ID | GO:0003707 |
|  | GO:0003677 |
|  | GO:0003706 |
|  | GO:0006355 |
|  | GO:0005515 |
|  | GO:0006631 |
|  | GO:0003700 |
|  | GO:0006006 |
|  | GO:0019217 |
|  | GO:0005634 |
|  | GO:0004872 |
|  | GO:0006091 |
|  | GO:0016563 |
|  | GO:0004879 |
|  | GO:0006366 |
|  | GO:0046872 |
|  | GO:0008544 |
|  | GO:0019904 |
|  | GO:0006350 |
|  | GO:0045944 |
|  | GO:0006629 |


---

|  |  |
| --- | --- |
| MIM | MIM:170998 |


---

|  |  |
| --- | --- |
| Connectivity | 1277 |


---

|  |  |
| --- | --- |
| Entrez ID | 19013 |
|  | 5465 |
|  | 25747 |


---

|  |  |
| --- | --- |
| Agilent ID | A\_23\_P306730 |
|  | A\_14\_P133767 |
|  | A\_14\_P119181 |
|  | A\_24\_P570049 |
|  | A\_51\_P348334 |
|  | A\_23\_P211643 |
|  | A\_14\_P139634 |
|  | A\_32\_P398516 |
|  | A\_24\_P417036 |
|  | A\_53\_P104891 |
|  | A\_53\_P180432 |
|  | A\_14\_P109098 |
|  | A\_23\_P40724 |
|  | A\_42\_P509766 |
|  | A\_23\_P211640 |


---

|  |  |
| --- | --- |
| Cellular Localization | Nucleus |
|  | Organelle |
|  | Cell |


---

|  |  |
| --- | --- |
| DbXref | KEGG pathway##04920##Adipocytokine signaling pathway##http://www.genome.jp/dbget-bin/show\_pathway?mmu04920+19013 |


---

|  |  |
| --- | --- |
| Pathway | MTF1 pathway NLP-enriched |
|  | Zn xs inventory |
|  | Zn xs DIN |


---

|  |  |
| --- | --- |
| GO Process | regulation of fatty acid metabolism |
|  | glucose metabolism |
|  | regulation of transcription, DNA-dependent |
|  | transcription |
|  | transcription from RNA polymerase II promoter |
|  | generation of precursor metabolites and energy |
|  | lipid metabolism |
|  | fatty acid metabolism |
|  | positive regulation of transcription from RNA polymerase II promoter |
|  | epidermis development |


---

|  |  |
| --- | --- |
| UniGene | Hs.534037 |
|  | Mm.212789 |
|  | Hs.275711 |
|  | Rn.9753 |
|  | Hs.103110 |


---

|  |  |
| --- | --- |
| Affymetrix Probeset ID | 102668\_at |
|  | 1387278\_at |
|  | 1391847\_at |
|  | 1449051\_at |
|  | 1558631\_at |
|  | 1560981\_a\_at |
|  | 206870\_at |
|  | 210771\_at |
|  | 223437\_at |
|  | 223438\_s\_at |
|  | 226978\_at |
|  | 244689\_at |
|  | 42375\_at |
|  | 44231\_at |
|  | 47512\_at |
|  | 1394800\_at |
|  | 59974\_r\_at |
|  | 90256\_at |
|  | 929\_at |
|  | g12652612\_3p\_at |
|  | g12652612\_3p\_x\_at |
|  | g13278779\_3p\_a\_at |
|  | g7549810\_3p\_at |
|  | Hs2.103110.1.S1\_3p\_s\_at |
|  | 1372619\_at |
|  | Hs2.391179.1.A1\_3p\_at |
|  | Hs.272006.0.A1\_3p\_at |
|  | Hs.272006.0.A1\_3p\_x\_at |
|  | Hs.275711.0.S2\_3p\_at |
|  | Hs.286014.0.S1\_3p\_at |
|  | L02932\_at |
|  | M88592\_at |
|  | rc\_AI102942\_at |
|  | x57638\_s\_at |
|  | 209491\_s\_at |
|  | 70524\_r\_at |
|  | Hs.83918.1.S1\_3p\_a\_at |
|  | RC\_AA461080\_at |
|  | RC\_N48315\_at |
|  | RC\_R10075\_at |
|  | RC\_R10702\_at |
|  | RC\_W57774\_at |
|  | rc\_AI172185\_at |


---

|  |  |
| --- | --- |
| GO Function | ligand-dependent nuclear receptor activity |
|  | ligand-regulated transcription factor activity |
|  | DNA binding |
|  | transcriptional activator activity |
|  | protein binding |
|  | transcription factor activity |
|  | steroid hormone receptor activity |
|  | receptor activity |
|  | metal ion binding |
|  | protein domain specific binding |


---

|  |  |
| --- | --- |
| Nucleotide | AY258327 |
|  | BC009069 |
|  | AK149460 |
|  | AK027101 |
|  | X75289 |
|  | AY258326 |
|  | NM\_001001929 |
|  | BQ024839 |
|  | M88592 |
|  | X75292 |
|  | AY258330 |
|  | AY258329 |
|  | AY258331 |
|  | L02932 |
|  | S74349 |
|  | X75290 |
|  | NM\_005036 |
|  | NM\_001001928 |
|  | AY206718 |
|  | X75291 |
|  | CR457435 |
|  | AL049856 |
|  | BC004162 |
|  | AY258328 |
|  | X75294 |
|  | AF086231 |
|  | BC000052 |
|  | Y16186 |
|  | AK091885 |
|  | BC071932 |
|  | X75293 |
|  | AK024738 |
|  | BC016892 |
|  | NM\_011144 |
|  | X89577 |
|  | X75287 |
|  | CR456547 |
|  | AL078611 |
|  | AK081709 |
|  | NM\_001001930 |
|  | NM\_013196 |
|  | AK035676 |
|  | X75288 |
|  | NM\_032644 |
|  | AU099251 |
|  | Y07619 |
|  | X57638 |


---

|  |  |
| --- | --- |
| Protein | Q07869 |
|  | AAO89522 |
|  | AAB32649 |
|  | BAC38303 |
|  | CAA61754 |
|  | NP\_035274 |
|  | BAC29149 |
|  | NP\_001001929 |
|  | AAO89524 |
|  | AAO89525 |
|  | AAH71932 |
|  | CAA68898 |
|  | NP\_116033 |
|  | AAO13489 |
|  | AAA41918 |
|  | P23204 |
|  | AAH00052 |
|  | AAA36468 |
|  | CAG30433 |
|  | P37230 |
|  | NP\_001001928 |
|  | AAO89521 |
|  | AAH16892 |
|  | NP\_001001930 |
|  | CAA40856 |
|  | AAO89526 |
|  | CAG33716 |
|  | NP\_005027 |
|  | CAI22450 |
|  | CAA53042 |
|  | NP\_037328 |
|  | BAE28893 |
|  | CAA76112 |
|  | CAI18764 |
|  | AAO89523 |


---

|  |  |
| --- | --- |
| Organism | Mammal |


---

|  |  |
| --- | --- |
| Location | 15 48.8 cM (Mus musculus) |
|  | chromosome 7, 7q34 (Rattus norvegicus) |
|  | chromosome 22, 22q12-q13.1, 22q12-q13.1 (Homo sapiens) |
|  | chromosome 15, 15 48.8 cM, 15 E2 (Mus musculus) |
|  | 22q13.31 (Homo sapiens) |


---

|  |  |
| --- | --- |
