# Supplementary material for: Dynamic transcriptomic profiles of zebrafish gills in response to zinc supplementation
Source: BMC Genomics. 2010 Oct 11;11:553. doi: 10.1186/1471-2164-11-553 (PMC3091702; doi:10.1186/1471-2164-11-553)
Supplement: Additional file 2 — Interactive Direct Interaction Network representing the molecular interactions between zinc, copper, iron, calcium and proteins encoded by transcripts changed by zinc supplementation. Mini web-site containing index.html and hyperlinked pages in subdirectory describing a Direct Interaction Network automatically generated based on curated interactions contained within the proprietary PathwayArchitect database. Ovals represent proteins and the circles symbolize metal ions. Objects are coloured by their abundance in zebrafish at the time-point they were significantly different from the control is a scale from -4 fold (dark green) to +4 fold (dark red). Where significant differences were found at more than one time-point, the colour overlay shows expression at the first instance. Dark blue squares denote 'binding', and light blue squares 'expression'; green squares stand for 'regulation', green diamonds for 'metabolism', and green circles for 'promoter binding'. Arrow heads indicate directionality of the interaction where annotated. All nodes and edges can be further interrogated by selecting the relative area of the image. [file 1471-2164-11-553-S2.zip › PathwayArchitect Zn xs DIN/108806.html]

# PROTEIN: INCENP

|  |  |
| --- | --- |
| Name | INCENP |
| Type | PROTEIN |
| Description | inner centromere protein antigens 135/155kDa |
| Note | In mammalian cells, 2 broad groups of centromere-interacting proteins have been described: constitutively binding centromere proteins and 'passenger,' or transiently interacting, proteins (reviewed by Choo, 1997). The constitutive proteins include CENPA (centromere protein A; MIM 117139), CENPB (MIM 117140), CENPC1 (MIM 117141), and CENPD (MIM 117142). The term 'passenger proteins' encompasses a broad collection of proteins that localize to the centromere during specific stages of the cell cycle (Earnshaw and Mackay, 1994). These include CENPE (MIM 117143); MCAK (MIM 604538); KID (MIM 603213); cytoplasmic dynein (e.g., MIM 600112); CliPs (e.g., MIM 179838); and CENPF/mitosin (MIM 600236). The inner centromere proteins (INCENPs) (Earnshaw and Cooke, 1991), the initial members of the passenger protein group, display a broad localization along chromosomes in the early stages of mitosis but gradually become concentrated at centromeres as the cell cycle progresses into mid-metaphase. During telophase, the proteins are located within the midbody in the intercellular bridge, where they are discarded after cytokinesis (Cutts et al., 1999).[supplied by OMIM] |
| Alias | inner centromere protein INCENP |
|  | C77457 |
|  | INCENP |
|  | AU019509 |
|  | 2700067E22Rik |
|  | Inner centromere protein |
|  | Incenp |
|  | C130081E20 |
|  | chromosomal passenger protein |
|  | binds and activates aurora-B and -C in vivo and in vitro |
|  | inner centromere protein antigens (135kD, 155kD) |
|  | MGC111393 |


---

|  |  |
| --- | --- |
| GO Component | centric heterochromatin |
|  | central element |
|  | synaptonemal complex |
|  | midbody |
|  | chromosome, pericentric region |
|  | microtubule |
|  | nucleus |
|  | spindle |


---

|  |  |
| --- | --- |
| GO ID | GO:0005634 |
|  | GO:0005874 |
|  | GO:0000775 |
|  | GO:0007067 |
|  | GO:0030496 |
|  | GO:0005515 |
|  | GO:0000801 |
|  | GO:0005721 |
|  | GO:0000069 |
|  | GO:0051301 |
|  | GO:0005819 |
|  | GO:0000795 |
|  | GO:0007049 |


---

|  |  |
| --- | --- |
| MIM | MIM:604411 |


---

|  |  |
| --- | --- |
| Connectivity | 45 |


---

|  |  |
| --- | --- |
| Entrez ID | 16319 |
|  | 3619 |


---

|  |  |
| --- | --- |
| Agilent ID | A\_14\_P201658 |
|  | A\_14\_P131263 |
|  | A\_53\_P166847 |
|  | A\_51\_P264064 |
|  | A\_53\_P151062 |
|  | A\_23\_P116387 |
|  | A\_53\_P155766 |
|  | A\_53\_P166632 |


---

|  |  |
| --- | --- |
| Cellular Localization | Nucleus |
|  | Microtubule |
|  | Cytoskeleton |
|  | Chromosome |
|  | Cell |
|  | Organelle |


---

|  |  |
| --- | --- |
| Pathway | Master Regulators |
|  | Zn def RIN |
|  | Zn xs inventory |
|  | Zn xs DIN |


---

|  |  |
| --- | --- |
| GO Process | mitosis |
|  | centromere and kinetochore complex maturation |
|  | cell cycle |
|  | cell division |


---

|  |  |
| --- | --- |
| UniGene | Mm.29755 |
|  | Hs.142179 |


---

|  |  |
| --- | --- |
| Affymetrix Probeset ID | 104468\_at |
|  | 113149\_at |
|  | 1423092\_at |
|  | 1423093\_at |
|  | 1439252\_at |
|  | 1439436\_x\_at |
|  | 1441314\_at |
|  | 1566043\_at |
|  | 1566044\_at |
|  | 164272\_at |
|  | 166306\_at |
|  | 167338\_i\_at |
|  | 170509\_at |
|  | 219769\_at |
|  | 242787\_at |
|  | 244862\_at |
|  | 43768\_at |
|  | 76984\_at |
|  | 88327\_at |
|  | 93758\_at |
|  | aa014535\_s\_at |
|  | aa139030\_s\_at |
|  | g9910375\_3p\_at |
|  | Hs.142179.0.A1\_3p\_at |
|  | Hs2.384653.1.S1\_3p\_at |
|  | 140409\_f\_at |
|  | 128626\_f\_at |
|  | 112046\_at |
|  | 60489\_r\_at |
|  | 85405\_at |
|  | RC\_AA132545\_at |
|  | RC\_AA149634\_at |
|  | TC40529\_at |


---

|  |  |
| --- | --- |
| GO Function | protein binding |


---

|  |  |
| --- | --- |
| Nucleotide | BC021761 |
|  | BC052414 |
|  | AK081841 |
|  | AB100433 |
|  | AF282265 |
|  | AK034764 |
|  | AF117610 |
|  | BC098576 |
|  | NM\_016692 |
|  | AK088627 |
|  | NM\_020238 |
|  | AK056195 |
|  | AB100432 |
|  | AY714053 |
|  | BC032678 |
|  | AA823653 |
|  | BC037011 |
|  | AK012497 |
|  | AF116187 |


---

|  |  |
| --- | --- |
| Protein | AAU04398 |
|  | AAD26202 |
|  | AAF87584 |
|  | BAC55880 |
|  | AAD32094 |
|  | NP\_064623 |
|  | Q9WU62 |
|  | AAH98576 |
|  | BAC40462 |
|  | BAC38346 |
|  | AAH37011 |
|  | Q9NQS7 |
|  | NP\_057901 |
|  | BAC55879 |
|  | AAH52414 |


---

|  |  |
| --- | --- |
| Organism | Mammal |


---

|  |  |
| --- | --- |
| Location | chromosome 11, 11q12-q13 (Homo sapiens) |
|  | chromosome 19, 19 0.0 cM, 19 A (Mus musculus) |
|  | 19 0.0 cM (Mus musculus) |


---

|  |  |
| --- | --- |
