# Supplementary material for: Dynamic transcriptomic profiles of zebrafish gills in response to zinc supplementation
Source: BMC Genomics. 2010 Oct 11;11:553. doi: 10.1186/1471-2164-11-553 (PMC3091702; doi:10.1186/1471-2164-11-553)
Supplement: Additional file 2 — Interactive Direct Interaction Network representing the molecular interactions between zinc, copper, iron, calcium and proteins encoded by transcripts changed by zinc supplementation. Mini web-site containing index.html and hyperlinked pages in subdirectory describing a Direct Interaction Network automatically generated based on curated interactions contained within the proprietary PathwayArchitect database. Ovals represent proteins and the circles symbolize metal ions. Objects are coloured by their abundance in zebrafish at the time-point they were significantly different from the control is a scale from -4 fold (dark green) to +4 fold (dark red). Where significant differences were found at more than one time-point, the colour overlay shows expression at the first instance. Dark blue squares denote 'binding', and light blue squares 'expression'; green squares stand for 'regulation', green diamonds for 'metabolism', and green circles for 'promoter binding'. Arrow heads indicate directionality of the interaction where annotated. All nodes and edges can be further interrogated by selecting the relative area of the image. [file 1471-2164-11-553-S2.zip › PathwayArchitect Zn xs DIN/109502.html]

# PROTEIN: IRF1

|  |  |
| --- | --- |
| Name | IRF1 |
| Type | PROTEIN |
| Description | interferon regulatory factor 1 |
| Note | IRF1 encodes interferon regulatory factor 1, a member of the interferon regulatory transcription factor (IRF) family. IRF1 serves as an activator of interferons alpha and beta transcription, and in mouse it has been shown to be required for double-stranded RNA induction of these genes. IRF1 also functions as a transcription activator of genes induced by interferons alpha, beta, and gamma. Further, IRF1 has been shown to play roles in regulating apoptosis and tumor-suppressoion. |
| Alias | Irf1 |
|  | MAR |
|  | IRF1 |
|  | IRF-1 |
|  | AU020929 |
|  | Irf-1 |


---

|  |  |
| --- | --- |
| GO Component | nucleus |


---

|  |  |
| --- | --- |
| GO ID | GO:0045084 |
|  | GO:0005634 |
|  | GO:0003677 |
|  | GO:0045786 |
|  | GO:0006355 |
|  | GO:0045893 |
|  | GO:0006366 |
|  | GO:0006350 |
|  | GO:0006955 |
|  | GO:0003700 |
|  | GO:0007049 |


---

|  |  |
| --- | --- |
| MIM | MIM:147575 |
|  | MIM:153550 |
|  | MIM:137215 |


---

|  |  |
| --- | --- |
| Connectivity | 564 |


---

|  |  |
| --- | --- |
| Entrez ID | 16362 |
|  | 24508 |
|  | 3659 |


---

|  |  |
| --- | --- |
| Agilent ID | A\_52\_P175685 |
|  | A\_53\_P155688 |
|  | A\_14\_P105696 |
|  | A\_53\_P126951 |
|  | A\_23\_P41765 |
|  | A\_44\_P306602 |
|  | A\_51\_P146103 |
|  | A\_43\_P11477 |


---

|  |  |
| --- | --- |
| Cellular Localization | Nucleus |
|  | Organelle |
|  | Cell |


---

|  |  |
| --- | --- |
| Pathway | Zn xs inventory |
|  | Zn xs DIN |


---

|  |  |
| --- | --- |
| GO Process | negative regulation of progression through cell cycle |
|  | immune response |
|  | regulation of transcription, DNA-dependent |
|  | transcription |
|  | transcription from RNA polymerase II promoter |
|  | positive regulation of interleukin-12 biosynthesis |
|  | positive regulation of transcription, DNA-dependent |
|  | cell cycle |


---

|  |  |
| --- | --- |
| UniGene | Mm.105218 |
|  | Rn.6396 |
|  | Hs.436061 |


---

|  |  |
| --- | --- |
| Affymetrix Probeset ID | 102401\_at |
|  | 1368073\_at |
|  | 1448436\_a\_at |
|  | 202531\_at |
|  | 669\_s\_at |
|  | g4504720\_3p\_at |
|  | L05072\_s\_at |
|  | M21065\_s\_at |
|  | M34253\_at |
|  | M34253\_g\_at |
|  | 1386145\_at |
|  | TC25661\_at |
|  | rc\_AI236118\_at |


---

|  |  |
| --- | --- |
| GO Function | transcription factor activity |
|  | DNA binding |


---

|  |  |
| --- | --- |
| Nucleotide | L05072 |
|  | M21066 |
|  | AC003959 |
|  | AK152193 |
|  | X14454 |
|  | NM\_012591 |
|  | AK153514 |
|  | AK152104 |
|  | BC003821 |
|  | BC009483 |
|  | NM\_008390 |
|  | CR541713 |
|  | AK157347 |
|  | NM\_002198 |
|  | AL596182 |
|  | BT019755 |
|  | AB209624 |
|  | AB103081 |
|  | AK155983 |
|  | M34253 |
|  | AF410808 |
|  | BT019756 |
|  | AF410807 |
|  | AY225160 |
|  | M21065 |
|  | AF410809 |
|  | BC076382 |
|  | CT010234 |
|  | AK152005 |


---

|  |  |
| --- | --- |
| Protein | BAE30951 |
|  | NP\_002189 |
|  | AAV38561 |
|  | CAA32624 |
|  | AAA36043 |
|  | P15314 |
|  | NP\_036723 |
|  | AAN39136 |
|  | BAE34056 |
|  | P10914 |
|  | AAN39137 |
|  | P23570 |
|  | AAH09483 |
|  | CAJ18442 |
|  | CAI25046 |
|  | CAI25045 |
|  | BAE33535 |
|  | BAE32057 |
|  | AAP49221 |
|  | BAD92861 |
|  | CAI25043 |
|  | AAH03821 |
|  | AAV38560 |
|  | NP\_032416 |
|  | AAB93488 |
|  | CAG46514 |
|  | BAE31023 |
|  | CAI25044 |
|  | AAA41450 |
|  | AAH76382 |
|  | BAD89424 |
|  | BAE30869 |
|  | AAA39334 |


---

|  |  |
| --- | --- |
| Organism | Mammal |


---

|  |  |
| --- | --- |
| Location | chromosome 11, 11 29.0 cM, 11 B1.3 (Mus musculus) |
|  | chromosome 5, 5q31.1 (Homo sapiens) |
|  | chromosome 10, 10q22 (Rattus norvegicus) |
|  | 11 29.0 cM (Mus musculus) |


---

|  |  |
| --- | --- |
