# Supplementary material for: Dynamic transcriptomic profiles of zebrafish gills in response to zinc supplementation
Source: BMC Genomics. 2010 Oct 11;11:553. doi: 10.1186/1471-2164-11-553 (PMC3091702; doi:10.1186/1471-2164-11-553)
Supplement: Additional file 2 — Interactive Direct Interaction Network representing the molecular interactions between zinc, copper, iron, calcium and proteins encoded by transcripts changed by zinc supplementation. Mini web-site containing index.html and hyperlinked pages in subdirectory describing a Direct Interaction Network automatically generated based on curated interactions contained within the proprietary PathwayArchitect database. Ovals represent proteins and the circles symbolize metal ions. Objects are coloured by their abundance in zebrafish at the time-point they were significantly different from the control is a scale from -4 fold (dark green) to +4 fold (dark red). Where significant differences were found at more than one time-point, the colour overlay shows expression at the first instance. Dark blue squares denote 'binding', and light blue squares 'expression'; green squares stand for 'regulation', green diamonds for 'metabolism', and green circles for 'promoter binding'. Arrow heads indicate directionality of the interaction where annotated. All nodes and edges can be further interrogated by selecting the relative area of the image. [file 1471-2164-11-553-S2.zip › PathwayArchitect Zn xs DIN/111092.html]

# PROTEIN: CLDN4

|  |  |
| --- | --- |
| Name | CLDN4 |
| Type | PROTEIN |
| Description | claudin 4 |
| Note | This gene encodes an integral membrane protein, which belongs to the claudin family. The protein is a component of tight junction strands and may play a role in internal organ development and function during pre- and postnatal life. This gene is deleted in Williams-Beuren syndrome, a neurodevelopmental disorder affecting multiple systems. |
| Alias | Cper |
|  | CLDN4 |
|  | Clostridium perfringens enterotoxin receptor 1 |
|  | Williams-Beuren syndrome chromosome region 8 protein |
|  | hCPE-R |
|  | CPETR |
|  | CPETR1 |
|  | WBSCR8 |
|  | CEP-R |
|  | CPE-R |
|  | Cpetr1 |
|  | CPE- receptor |
|  | CPER |
|  | Cldn4 |
|  | Cpetr |
|  | Clostridium perfringens enterotoxin receptor |


---

|  |  |
| --- | --- |
| GO Component | tight junction |
|  | extracellular space |
|  | integral to membrane |
|  | plasma membrane |
|  | integral to plasma membrane |
|  | membrane |


---

|  |  |
| --- | --- |
| GO ID | GO:0016020 |
|  | GO:0005615 |
|  | GO:0042802 |
|  | GO:0016338 |
|  | GO:0005887 |
|  | GO:0016021 |
|  | GO:0005923 |
|  | GO:0004888 |
|  | GO:0005198 |
|  | GO:0005886 |
|  | GO:0009405 |


---

|  |  |
| --- | --- |
| MIM | MIM:602909 |


---

|  |  |
| --- | --- |
| Connectivity | 53 |


---

|  |  |
| --- | --- |
| Entrez ID | 1364 |
|  | 12740 |


---

|  |  |
| --- | --- |
| Agilent ID | A\_53\_P152063 |
|  | A\_24\_P115183 |
|  | A\_14\_P101946 |
|  | A\_51\_P356467 |
|  | A\_23\_P19944 |


---

|  |  |
| --- | --- |
| Cellular Localization | Plasma membrane |
|  | Membrane |
|  | Cell |
|  | Extracellular region |


---

|  |  |
| --- | --- |
| DbXref | KEGG pathway##04514##Cell adhesion molecules (CAMs)##http://www.genome.jp/dbget-bin/show\_pathway?mmu04514+12740 |
|  | KEGG pathway##04530##Tight junction##http://www.genome.jp/dbget-bin/show\_pathway?mmu04530+12740 |
|  | KEGG pathway##04530##Tight junction##http://www.genome.jp/dbget-bin/show\_pathway?hsa04530+1364 |


---

|  |  |
| --- | --- |
| Pathway | Zn xs inventory |
|  | Zn xs DIN |


---

|  |  |
| --- | --- |
| GO Process | pathogenesis |
|  | calcium-independent cell-cell adhesion |


---

|  |  |
| --- | --- |
| UniGene | Mm.7339 |
|  | Hs.520942 |


---

|  |  |
| --- | --- |
| Affymetrix Probeset ID | 101410\_at |
|  | 1418283\_at |
|  | 1569421\_at |
|  | 201428\_at |
|  | 35276\_at |
|  | 87864\_i\_at |
|  | 87867\_r\_at |
|  | AB000713\_at |
|  | AB000713\_g\_at |
|  | g4502876\_3p\_at |
|  | Hs2.352221.1.S1\_3p\_at |
|  | AA427468\_s\_at |
|  | TC36323\_s\_at |


---

|  |  |
| --- | --- |
| GO Function | transmembrane receptor activity |
|  | structural molecule activity |
|  | protein self binding |


---

|  |  |
| --- | --- |
| Nucleotide | AK126315 |
|  | BC000671 |
|  | BT006989 |
|  | NM\_001305 |
|  | AB000713 |
|  | AF087822 |
|  | AK145152 |
|  | BC032172 |
|  | AK124076 |
|  | AB000712 |
|  | AK026651 |
|  | NM\_009903 |
|  | BM768799 |
|  | AK162721 |
|  | AC093168 |
|  | AK126462 |


---

|  |  |
| --- | --- |
| Protein | AAP35635 |
|  | O14493 |
|  | BAE26263 |
|  | NP\_034033 |
|  | BAA22984 |
|  | AAD09757 |
|  | BAA22985 |
|  | AAH00671 |
|  | AAS07556 |
|  | NP\_001296 |
|  | O35054 |


---

|  |  |
| --- | --- |
| Organism | Mammal |


---

|  |  |
| --- | --- |
| Location | chromosome 5, 5 75.0 cM, 5 G2 (Mus musculus) |
|  | chromosome 7, 7q11.23 (Homo sapiens) |
|  | 5 75.0 cM (Mus musculus) |


---

|  |  |
| --- | --- |
