# Supplementary material for: Dynamic transcriptomic profiles of zebrafish gills in response to zinc supplementation
Source: BMC Genomics. 2010 Oct 11;11:553. doi: 10.1186/1471-2164-11-553 (PMC3091702; doi:10.1186/1471-2164-11-553)
Supplement: Additional file 2 — Interactive Direct Interaction Network representing the molecular interactions between zinc, copper, iron, calcium and proteins encoded by transcripts changed by zinc supplementation. Mini web-site containing index.html and hyperlinked pages in subdirectory describing a Direct Interaction Network automatically generated based on curated interactions contained within the proprietary PathwayArchitect database. Ovals represent proteins and the circles symbolize metal ions. Objects are coloured by their abundance in zebrafish at the time-point they were significantly different from the control is a scale from -4 fold (dark green) to +4 fold (dark red). Where significant differences were found at more than one time-point, the colour overlay shows expression at the first instance. Dark blue squares denote 'binding', and light blue squares 'expression'; green squares stand for 'regulation', green diamonds for 'metabolism', and green circles for 'promoter binding'. Arrow heads indicate directionality of the interaction where annotated. All nodes and edges can be further interrogated by selecting the relative area of the image. [file 1471-2164-11-553-S2.zip › PathwayArchitect Zn xs DIN/111751.html]

# PROTEIN: MYL9

|  |  |
| --- | --- |
| Name | MYL9 |
| Type | PROTEIN |
| Description | myosin, light polypeptide 9, regulatory |
| Note | Myosin, a structural component of muscle, consists of two heavy chains and four light chains. The protein encoded by this gene is a myosin light chain that may regulate muscle contraction by modulating the ATPase activity of myosin heads. The encoded protein binds calcium and is activated by myosin light chain kinase. Two transcript variants encoding different isoforms have been found for this gene. |
| Alias | AI327049 |
|  | myosin regulatory light chain 2, smooth muscle isoform |
|  | myosin regulatory light polypeptide 9 |
|  | MYL9 |
|  | Myl9 |
|  | MLC2 |
|  | Myrl2 |
|  | LC20 |
|  | MRLC1 |
|  | MYRL2 |
|  | Myosin RLC |
|  | myosin RLC |
|  | MGC3505 |


---

|  |  |
| --- | --- |
| GO Component | muscle myosin |
|  | myosin |


---

|  |  |
| --- | --- |
| GO ID | GO:0006937 |
|  | GO:0005859 |
|  | GO:0005509 |
|  | GO:0016459 |
|  | GO:0003774 |
|  | GO:0008307 |
|  | GO:0007517 |


---

|  |  |
| --- | --- |
| Connectivity | 45 |


---

|  |  |
| --- | --- |
| Entrez ID | 10398 |
|  | 98932 |


---

|  |  |
| --- | --- |
| Agilent ID | A\_23\_P210425 |
|  | A\_14\_P108459 |
|  | A\_23\_P210428 |
|  | A\_51\_P308298 |


---

|  |  |
| --- | --- |
| Cellular Localization | Cytoskeleton |
|  | Cytoplasm |
|  | Organelle |
|  | Cell |


---

|  |  |
| --- | --- |
| Pathway | Zn xs inventory |
|  | Zn xs DIN |


---

|  |  |
| --- | --- |
| GO Process | regulation of muscle contraction |
|  | muscle development |


---

|  |  |
| --- | --- |
| UniGene | Hs.504687 |
|  | Mm.271770 |


---

|  |  |
| --- | --- |
| Affymetrix Probeset ID | 1452670\_at |
|  | 201058\_3p\_s\_at |
|  | 201058\_s\_at |
|  | 39145\_at |
|  | 96939\_at |
|  | g5174602\_3p\_at |
|  | g5174602\_3p\_x\_at |
|  | J02854\_at |
|  | Msa.15674.0\_f\_at |
|  | Msa.18350.0\_s\_at |
|  | Msa.24189.0\_f\_at |
|  | Msa.4408.0\_f\_at |
|  | 244149\_at |
|  | 78579\_at |
|  | Hs.134192.0.A1\_3p\_at |
|  | TC40775\_at |
|  | TC40775\_g\_at |


---

|  |  |
| --- | --- |
| GO Function | structural constituent of muscle |
|  | motor activity |
|  | calcium ion binding |


---

|  |  |
| --- | --- |
| Nucleotide | CR456843 |
|  | BC055439 |
|  | AL050318 |
|  | D82057 |
|  | AF176042 |
|  | AI842649 |
|  | NM\_181526 |
|  | BC002648 |
|  | AK130879 |
|  | NM\_006097 |
|  | AK159880 |
|  | AK097235 |
|  | AK020258 |
|  | AI327049 |
|  | AA473002 |
|  | AK007972 |
|  | AF176043 |
|  | BC049974 |
|  | J02854 |
|  | XM\_485171 |


---

|  |  |
| --- | --- |
| Protein | AAQ13654 |
|  | BAB88917 |
|  | CAG33124 |
|  | BAE35452 |
|  | BAB32043 |
|  | CAB75369 |
|  | BAB25381 |
|  | AAH02648 |
|  | NP\_852667 |
|  | AAH49974 |
|  | AAA59852 |
|  | Q9CQ19 |
|  | P24844 |
|  | AAQ13653 |
|  | CAC34440 |
|  | XP\_485171 |
|  | NP\_006088 |
|  | AAH55439 |


---

|  |  |
| --- | --- |
| Organism | Mammal |


---

|  |  |
| --- | --- |
| Location | chromosome 20, 20q11.23 (Homo sapiens) |
|  | chromosome 2, 2 H1 (Mus musculus) |


---

|  |  |
| --- | --- |
