# Supplementary material for: Dynamic transcriptomic profiles of zebrafish gills in response to zinc supplementation
Source: BMC Genomics. 2010 Oct 11;11:553. doi: 10.1186/1471-2164-11-553 (PMC3091702; doi:10.1186/1471-2164-11-553)
Supplement: Additional file 2 — Interactive Direct Interaction Network representing the molecular interactions between zinc, copper, iron, calcium and proteins encoded by transcripts changed by zinc supplementation. Mini web-site containing index.html and hyperlinked pages in subdirectory describing a Direct Interaction Network automatically generated based on curated interactions contained within the proprietary PathwayArchitect database. Ovals represent proteins and the circles symbolize metal ions. Objects are coloured by their abundance in zebrafish at the time-point they were significantly different from the control is a scale from -4 fold (dark green) to +4 fold (dark red). Where significant differences were found at more than one time-point, the colour overlay shows expression at the first instance. Dark blue squares denote 'binding', and light blue squares 'expression'; green squares stand for 'regulation', green diamonds for 'metabolism', and green circles for 'promoter binding'. Arrow heads indicate directionality of the interaction where annotated. All nodes and edges can be further interrogated by selecting the relative area of the image. [file 1471-2164-11-553-S2.zip › PathwayArchitect Zn xs DIN/112290.html]

# PROTEIN: CES1

|  |  |
| --- | --- |
| Name | CES1 |
| Type | PROTEIN |
| Description | carboxylesterase 1 (monocyte/macrophage serine esterase 1) |
| Note | Carboxylesterase 1 is a member of a large multigene family. The enzymes encoded by these genes are responsible for the hydrolysis of ester- and amide-bond-containing drugs such as cocaine and heroin. They also hydrolize long-chain fatty acid esters and thioesters. This enzyme is known to hydrolyze aromatic and aliphatic esters and is necessary for cellular cholesterol esterification. It may also play a role in detoxification in the lung and/or protection of the central nervous system from ester or amide compounds. Carboxylesterase deficiency may be associated with non-Hodgkin lymphoma or B-cell lymphocytic leukemia. Three transcript variants encoding three different isoforms have been found for this gene. |
| Alias | HMSE1 |
|  | cholesteryl ester hydrolase |
|  | CES2 |
|  | TGH |
|  | egasyn |
|  | SES1 |
|  | Ces1 |
|  | Carboxyesterase ES-4 |
|  | pI 5.5 esterase |
|  | acyl coenzyme A:cholesterol acyltransferase |
|  | ACAT |
|  | Microsomal palmitoyl-CoA hydrolase |
|  | HMSE |
|  | Kidney microsomal carboxylesterase |
|  | Ces-1 |
|  | Liver microsomal carboxylesterase |
|  | Carboxyesterase ES-3 |
|  | carboxylesterase 1 |
|  | ES-x |
|  | carboxylesterase 2 (liver) |
|  | Ses-1 |
|  | liver carboxylesterase |
|  | triacylglycerol hydrolase |
|  | ES-HTEL |
|  | CEH |
|  | Acyl coenzyme A:cholesterol acyltransferase |


---

|  |  |
| --- | --- |
| GO Component | extracellular space |
|  | endoplasmic reticulum |


---

|  |  |
| --- | --- |
| GO ID | GO:0005783 |
|  | GO:0005615 |
|  | GO:0008152 |
|  | GO:0003824 |
|  | GO:0004759 |
|  | GO:0009636 |
|  | GO:0016787 |
|  | GO:0004091 |
|  | GO:0016789 |
|  | GO:0016290 |


---

|  |  |
| --- | --- |
| MIM | MIM:114835 |


---

|  |  |
| --- | --- |
| Connectivity | 298 |


---

|  |  |
| --- | --- |
| Entrez ID | 29225 |
|  | 12623 |
|  | 1066 |


---

|  |  |
| --- | --- |
| Agilent ID | A\_53\_P147513 |
|  | A\_43\_P15849 |
|  | A\_23\_P206733 |
|  | A\_52\_P245962 |
|  | A\_44\_P498795 |
|  | A\_51\_P253481 |
|  | A\_51\_P253484 |
|  | A\_53\_P180477 |
|  | A\_42\_P688604 |


---

|  |  |
| --- | --- |
| Cellular Localization | Endoplasmic reticulum |
|  | Cytoplasm |
|  | Extracellular region |
|  | Organelle |
|  | Cell |


---

|  |  |
| --- | --- |
| DbXref | KEGG pathway##00960##Alkaloid biosynthesis II##http://www.genome.jp/dbget-bin/show\_pathway?hsa00960+1066 |
|  | KEGG pathway##00960##Alkaloid biosynthesis II##http://www.genome.jp/dbget-bin/show\_pathway?mmu00960+12623 |
|  | KEGG pathway##00960##Alkaloid biosynthesis II##http://www.genome.jp/dbget-bin/show\_pathway?rno00960+29225 |


---

|  |  |
| --- | --- |
| Pathway | Zn xs inventory |
|  | Zn xs DIN |


---

|  |  |
| --- | --- |
| GO Process | metabolism |
|  | response to toxin |


---

|  |  |
| --- | --- |
| UniGene | Hs.535486 |
|  | Hs.499222 |
|  | Mm.22720 |
|  | Rn.82692 |


---

|  |  |
| --- | --- |
| Affymetrix Probeset ID | 103519\_at |
|  | 231672\_at |
|  | 1387214\_at |
|  | 1449486\_at |
|  | 164228\_at |
|  | 209616\_s\_at |
|  | 37203\_at |
|  | g688112\_3p\_at |
|  | L07765\_at |
|  | 110127\_at |
|  | U10697\_s\_at |
|  | X81395\_at |
|  | Hs.76688.1.A1\_3p\_at |
|  | RC\_T68878\_f\_at |
|  | 1370352\_at |


---

|  |  |
| --- | --- |
| EC Number | EC 3.1.1.1 |


---

|  |  |
| --- | --- |
| GO Function | hydrolase activity |
|  | palmitoyl-CoA hydrolase activity |
|  | serine esterase activity |
|  | carboxylesterase activity |
|  | catalytic activity |
|  | carboxylic ester hydrolase activity |


---

|  |  |
| --- | --- |
| Nucleotide | BC026897 |
|  | X81825 |
|  | L07764 |
|  | AK162328 |
|  | AB119997 |
|  | NM\_031565 |
|  | AB119996 |
|  | S73751 |
|  | NM\_001266 |
|  | AB119995 |
|  | BC012418 |
|  | X65294 |
|  | AB119998 |
|  | AF177775 |
|  | NM\_001025194 |
|  | AB025026 |
|  | U10698 |
|  | NM\_021456 |
|  | X81395 |
|  | AY268104 |
|  | M55509 |
|  | M65261 |
|  | AK149446 |
|  | AK136446 |
|  | AY033590 |
|  | BC009706 |
|  | U10697 |
|  | X52973 |
|  | Y12887 |
|  | AB023630 |
|  | X96751 |
|  | L07765 |
|  | D21088 |
|  | AK136588 |
|  | M73499 |
|  | NM\_001025195 |
|  | X65295 |
|  | BC021150 |


---

|  |  |
| --- | --- |
| Protein | CAA57419 |
|  | AAA64638 |
|  | Q64573 |
|  | CAA65527 |
|  | Q8VCC2 |
|  | Q63108 |
|  | BAA04650 |
|  | BAE36856 |
|  | NP\_001257 |
|  | BAA84995 |
|  | CAA73388 |
|  | BAC87748 |
|  | AAA83932 |
|  | AAH12418 |
|  | P23141 |
|  | AAA35649 |
|  | BAC87751 |
|  | BAB60697 |
|  | NP\_001020365 |
|  | BAE28882 |
|  | AAH09706 |
|  | AAA64639 |
|  | NP\_113753 |
|  | Q63010 |
|  | BAC87750 |
|  | CAA57158 |
|  | AAD53175 |
|  | BAE23063 |
|  | AAP20868 |
|  | NP\_067431 |
|  | NP\_001020366 |
|  | CAA37147 |
|  | AAH21150 |
|  | AAA35650 |
|  | CAA46389 |
|  | AAH26897 |
|  | AAA16036 |
|  | BAE22982 |
|  | BAC87749 |
|  | AAA35711 |
|  | AAC60631 |
|  | CAA46390 |


---

|  |  |
| --- | --- |
| Organism | Mammal |


---

|  |  |
| --- | --- |
| Location | 8 43.0 cM (Mus musculus) |
|  | chromosome 19, 19p11 (Rattus norvegicus) |
|  | chromosome 8, 8 43.0 cM, 8 C5 (Mus musculus) |
|  | chromosome 16, 16q13-q22.1 (Homo sapiens) |


---

|  |  |
| --- | --- |
