# Supplementary material for: Dynamic transcriptomic profiles of zebrafish gills in response to zinc supplementation
Source: BMC Genomics. 2010 Oct 11;11:553. doi: 10.1186/1471-2164-11-553 (PMC3091702; doi:10.1186/1471-2164-11-553)
Supplement: Additional file 2 — Interactive Direct Interaction Network representing the molecular interactions between zinc, copper, iron, calcium and proteins encoded by transcripts changed by zinc supplementation. Mini web-site containing index.html and hyperlinked pages in subdirectory describing a Direct Interaction Network automatically generated based on curated interactions contained within the proprietary PathwayArchitect database. Ovals represent proteins and the circles symbolize metal ions. Objects are coloured by their abundance in zebrafish at the time-point they were significantly different from the control is a scale from -4 fold (dark green) to +4 fold (dark red). Where significant differences were found at more than one time-point, the colour overlay shows expression at the first instance. Dark blue squares denote 'binding', and light blue squares 'expression'; green squares stand for 'regulation', green diamonds for 'metabolism', and green circles for 'promoter binding'. Arrow heads indicate directionality of the interaction where annotated. All nodes and edges can be further interrogated by selecting the relative area of the image. [file 1471-2164-11-553-S2.zip › PathwayArchitect Zn xs DIN/1128016.html]

# EXPRESSION:

|  |  |
| --- | --- |
| Type | EXPRESSION |
| Effect | None |


---

|  |  |
| --- | --- |
| Score | 0 |


---

|  |  |
| --- | --- |
| Reference Count | 4 |


---

|  |  |
| --- | --- |
| Mechanism | Unknown |


---

|  |  |
| --- | --- |
| Reference:0 || Sentence | "WT1 variants that contain the KTS insert are impaired in their ability to bind to the IGF-IR promoter and are unable to suppress IGF-IR promoter." |
| PMID | 12444079 |
| Year | 2002 |
| Species | Human |
| Journal | J Biol Chem |
| RefScore | 2 |
| Source | PArchNLP |
  |
|


---

|  |  |
| --- | --- |
 Reference:1 || Sentence | "Coimmunoprecipitation experiments showed that p53 and WT1 physically interact, whereas electrophoretic mobility shift assay studies revealed that p53 modulates the ability of WT1 to bind to the IGF-IR promoter." |
| PMID | 12444079 |
| Year | 2002 |
| Species | Human |
| Journal | J Biol Chem |
| RefScore | 1 |
| Source | PArchNLP |
  ||


---

|  |  |
| --- | --- |
 Reference:2 || Sentence | "P69 clones stably transfected with a WT1 expression vector exhibited decreased expression of the endogenous IGF-IR gene and decreased promoter activity in transient transfection assays with IGF-IR promoter constructs containing multiple WT1 binding sites." |
| PMID | 11145562 |
| Year | 2001 |
| Species | Mouse |
|  | Human |
| Journal | Endocrinology |
| RefScore | 1 |
| Source | PArchNLP |
  ||


---

|  |  |
| --- | --- |
 Reference:3 || Sentence | "These results suggest that underexpression, deletion, or mutation of WT1 may result in increased expression of the IGF-IR, whose activation by IGF-II may be an important aspect of the biology of Wilms tumor." |
| PMID | 8390684 |
| Year | 1993 |
| Species | Human |
|  | Rat |
| Journal | Proc Natl Acad Sci U S A |
| RefScore | 2 |
| Source | PArchNLP |
  |


---

|  |  |
| --- | --- |
