# Supplementary material for: Dynamic transcriptomic profiles of zebrafish gills in response to zinc supplementation
Source: BMC Genomics. 2010 Oct 11;11:553. doi: 10.1186/1471-2164-11-553 (PMC3091702; doi:10.1186/1471-2164-11-553)
Supplement: Additional file 2 — Interactive Direct Interaction Network representing the molecular interactions between zinc, copper, iron, calcium and proteins encoded by transcripts changed by zinc supplementation. Mini web-site containing index.html and hyperlinked pages in subdirectory describing a Direct Interaction Network automatically generated based on curated interactions contained within the proprietary PathwayArchitect database. Ovals represent proteins and the circles symbolize metal ions. Objects are coloured by their abundance in zebrafish at the time-point they were significantly different from the control is a scale from -4 fold (dark green) to +4 fold (dark red). Where significant differences were found at more than one time-point, the colour overlay shows expression at the first instance. Dark blue squares denote 'binding', and light blue squares 'expression'; green squares stand for 'regulation', green diamonds for 'metabolism', and green circles for 'promoter binding'. Arrow heads indicate directionality of the interaction where annotated. All nodes and edges can be further interrogated by selecting the relative area of the image. [file 1471-2164-11-553-S2.zip › PathwayArchitect Zn xs DIN/1132471.html]

# REGULATION:

|  |  |
| --- | --- |
| Type | REGULATION |
| Effect | None |


---

|  |  |
| --- | --- |
| Score | 0 |


---

|  |  |
| --- | --- |
| Reference Count | 12 |


---

|  |  |
| --- | --- |
| Mechanism | Unknown |


---

|  |  |
| --- | --- |
| Reference:0 || Sentence | "These results strongly suggest that pyoverdin competes directly with transferrin for iron and that it is an essential element for in vivo iron gathering and virulence expression in P. aeruginosa." |
| PMID | 8550201 |
| Year | 1996 |
| Species | Mouse |
|  | Human |
| Journal | Infect Immun |
| RefScore | 2 |
| Source | PArchNLP |
  |
|


---

|  |  |
| --- | --- |
 Reference:1 || Sentence | "The natural resistance associated macrophage protein 2 (Nramp2) is a transporter that is involved in iron (Fe) uptake from transferrin (Tf) and low molecular mass Fe complexes." |
| PMID | 11054110 |
| Year | 2000 |
| Species | Mouse |
| Journal | Eur J Biochem |
| RefScore | 1 |
| Source | PArchNLP |
  ||


---

|  |  |
| --- | --- |
 Reference:2 || Sentence | Strains from environmental or clinical sources were similar in virulence-associated phenotypes (protease activity, utilization of transferrin-bound iron, hemolysis, and inactivation in serum) and susceptibility to various stresses (4 and 52 degrees C, 0.1 and 10% NaCl, and pH 3.2), except freeze-thaw treatment. |
| Year | 2005 |
| PMID | 16355823 |
| Species | Human |
| Journal | J Food Prot |
| RefScore | 1 |
| Source | PArchNLP |
  ||


---

|  |  |
| --- | --- |
 Reference:3 || Sentence | CONCLUSIONS: Increased mRNA and protein levels of the iron-regulating proteins transferrin, ceruloplasmin, and ferritin are present in glaucoma. |
| Year | 2004 |
| PMID | 15111596 |
| Species | Human |
| Journal | Invest Ophthalmol Vis Sci |
| RefScore | 0 |
| Source | PArchNLP |
  ||


---

|  |  |
| --- | --- |
 Reference:4 || Sentence | Two structurally related sulfonamides were found to be potent and reversible inhibitors of transferrin-mediated iron uptake. |
| Year | 2004 |
| PMID | 15180825 |
| Species | Human |
| Journal | Traffic |
| RefScore | 1 |
| Source | PArchNLP |
  ||


---

|  |  |
| --- | --- |
 Reference:5 || Sentence | Transferrin, the major plasma iron carrier, mediates iron entry into cells through interaction with its receptor. |
| Year | 2004 |
| PMID | 15270724 |
| Species | Mouse |
| Journal | Immunology |
| RefScore | 1 |
| Source | PArchNLP |
  ||


---

|  |  |
| --- | --- |
 Reference:6 || Sentence | Homozygous mutations of TfR2 cause haemochromatosis, suggesting that TfR2 alpha may not be a simple iron transporter, but a regulator of iron by identifying iron-Tf. |
| PMID | 15521925 |
| Year | 2004 |
| Species | Human |
| Journal | Br J Haematol |
| RefScore | 1 |
| Source | PArchNLP |
  ||


---

|  |  |
| --- | --- |
 Reference:7 || Sentence | It affects cellular acquisition of iron by binding to transferrin, and it interacts with the iron-dependent enzyme ribonucleotide reductase, resulting in reduced dNTP pools and inhibition of DNA synthesis. |
| PMID | 15579097 |
| Year | 2004 |
| Species | Human |
| Journal | Curr Top Med Chem |
| RefScore | 1 |
| Source | PArchNLP |
  ||


---

|  |  |
| --- | --- |
 Reference:8 || Sentence | Aluminum exposure affects transferrin-dependent and -independent iron uptake by K562 cells. |
| PMID | 16085060 |
| Year | 2005 |
| Species | Human |
| Journal | Biochim Biophys Acta |
| RefScore | 2 |
| Source | PArchNLP |
  ||


---

|  |  |
| --- | --- |
 Reference:9 || Sentence | Experiments using cells over-expressing wild-type HFE confirm the existence of beta2microglobulin(beta2m)/HFE and HFE/transferrin receptor 1 (TfR1) interactions, as well as the capacity of HFE to reduce transferrin-mediated iron uptake. |
| PMID | 15965644 |
| Year | 2005 |
| Species | Human |
| Journal | Hum Genet |
| RefScore | 1 |
| Source | PArchNLP |
  ||


---

|  |  |
| --- | --- |
 Reference:10 || Sentence | The trafficking of transferrin-TfR1-PCP complex during the process of transferrin-mediated iron uptake was imaged by fluorescence resonance energy transfer between the fluorescently labeled transferrin ligand and TfR1 receptor. |
| PMID | 16183024 |
| Year | 2005 |
| Journal | Chem Biol |
| RefScore | 1 |
| Source | PArchNLP |
  ||


---

|  |  |
| --- | --- |
 Reference:11 || Sentence | We hypothesized that a labile iron component associated with non-transferrin-bound iron (NTBI) that appears in individuals with overt or cryptic iron overload might be more suitable for establishing correlations with CHD. |
| PMID | 16618820 |
| Year | 2006 |
| Species | Human |
| Journal | Circulation |
| RefScore | 1 |
| Source | PArchNLP |
  |


---

|  |  |
| --- | --- |
