# Supplementary material for: Dynamic transcriptomic profiles of zebrafish gills in response to zinc supplementation
Source: BMC Genomics. 2010 Oct 11;11:553. doi: 10.1186/1471-2164-11-553 (PMC3091702; doi:10.1186/1471-2164-11-553)
Supplement: Additional file 2 — Interactive Direct Interaction Network representing the molecular interactions between zinc, copper, iron, calcium and proteins encoded by transcripts changed by zinc supplementation. Mini web-site containing index.html and hyperlinked pages in subdirectory describing a Direct Interaction Network automatically generated based on curated interactions contained within the proprietary PathwayArchitect database. Ovals represent proteins and the circles symbolize metal ions. Objects are coloured by their abundance in zebrafish at the time-point they were significantly different from the control is a scale from -4 fold (dark green) to +4 fold (dark red). Where significant differences were found at more than one time-point, the colour overlay shows expression at the first instance. Dark blue squares denote 'binding', and light blue squares 'expression'; green squares stand for 'regulation', green diamonds for 'metabolism', and green circles for 'promoter binding'. Arrow heads indicate directionality of the interaction where annotated. All nodes and edges can be further interrogated by selecting the relative area of the image. [file 1471-2164-11-553-S2.zip › PathwayArchitect Zn xs DIN/1134928.html]

# BINDING:

|  |  |
| --- | --- |
| Type | BINDING |
| Effect | None |


---

|  |  |
| --- | --- |
| Score | 0 |


---

|  |  |
| --- | --- |
| Reference Count | 18 |


---

|  |  |
| --- | --- |
| Mechanism | Unknown |


---

|  |  |
| --- | --- |
| Reference:0 || Sentence | "Using in vitro expressed human receptors, we now show that ER-beta binds to a panel of six endogenous hormone response elements (vitellogenin, c-fos, c-jun, pS2, cathepsin D, and choline acetyltransferase) already known to bind ER-alpha and confer estrogen inducibility to reporter constructs." |
| PMID | 10037443 |
| Year | 1999 |
| Species | Mouse |
|  | Human |
| Journal | Biochem Pharmacol |
| RefScore | 0 |
| Source | PArchNLP |
  |
|


---

|  |  |
| --- | --- |
 Reference:1 || PMID | 11477071 |
| SourceID | 183847 |
| Species | Human |
| Experimental Condition | in-vitro |
| Description | An interaction between human ER-alpha and mouse c-Jun was demonstrated by GST pull-down assay. [35S]-methionine labelled in vitro transcribed and translated ER-alpha[Gly400Val] (HEO) was isolated using purified GST-c-Jun immobilized on glutathione-Sepharose beads. Eluates were resolved by SDS-PAGE and detected by autoradiography. Fig. 1B. The mutants [35S]-ER-alpha[Gly400Val,179-595] (HE19), and [35S]-ER-alpha[Gly400Val,1-185,250-595]] (HE11) were isolated by GST-c-Jun. Fig. 1B. The mutant [35S]-ER-alpha[Gly400Val,1-282] (HE19) was not isolated by GST-c-Jun. Fig 1B. [35S]-c-Jun was detected by autoradiography of GST-ER-alpha complexes eluted from glutathione-Sepharose beads. Data not shown, p. 36363. |
| Detection Method | affinity-chromatography |
| Source | BIND |
  ||


---

|  |  |
| --- | --- |
 Reference:2 || PMID | 11477071 |
| SourceID | 183847 |
| Species | Human |
| Experimental Condition | in-vitro |
| Description | An interaction between human ER-alpha and mouse c-Jun was demonstrated by GST pull-down assay. [35S]-methionine labelled in vitro transcribed and translated c-Jun was isolated using purified GST-ER-alpha[259-302] immobilized on glutathione-Sepharose beads. Eluates were resolved by SDS-PAGE and detected by autoradiography. Fig. 2B. [35S]-c-Jun was isolated by the mutants GST- ER-alpha[179-312], GST-ER-alpha[251-312], GST-ER-alpha[251- 595], and mouse GST-ER-alpha[313-599]. Fig. 2B, 6. [35S]-c-Jun was isolated by the mutant GST-ER-alpha[251- 595] independent of treatment with estradiol (E2), 4-hydroxytamoxifen (OHT) or ICI164,384 (ICI). Fig. 6. The mutants [35S]-c-Jun[239-334], [35S]-c-Jun- [1-145,222-334], and [35S]-c-Jun[1-5,195-334] were isolated by the mutant GST-ER-alpha[251-595]. Fig. 3B. [35S]-c-Jun was not isolated by the mutants GST- ER-alpha[2-184] and GST-ER- alpha[283-330]. Fig 2B. The mutant [35S]-c-Jun[1-223] was not isolated by the mutant GST-ER-alpha[251-595]. Fig. 3B. |
| Detection Method | affinity-chromatography |
| Source | BIND |
  ||


---

|  |  |
| --- | --- |
 Reference:3 || PMID | 11477071 |
| SourceID | 183847 |
| Species | Human |
| Experimental Condition | in-vitro |
| Description | An interaction between human ER-alpha and mouse c-Jun was demonstrated by GST pull-down assay. [35S]-methionine labelled in vitro transcribed and translated c-Jun bound to a[32P]-labelled oligonucleotide containing the collagenase transcription regulatory element (TRE) was isolated using purified GST-ER-alpha[251-595] (GST-ER) immobilized on glutathione-Sepharose beads. Eluates were resolved by SDS-PAGE and detected by autoradiography. Fig. 5. |
| Detection Method | affinity-chromatography |
| Source | BIND |
  ||


---

|  |  |
| --- | --- |
 Reference:4 || PMID | 11477071 |
| SourceID | 183847 |
| Species | Human |
| Experimental Condition | in-vitro |
| Description | An interaction between human ER-alpha and mouse c-Jun was demonstrated by mammalian two hybrid assay. c-Jun, bait, fused to the GAL4 DNA binding domain (DBD) and ER-alpha, prey, fused to the VP16 activation domain (AD) were co-transfected into COS cells. Interactions were detected by luciferase expression. Fig. 7A. GAL4-DBD-c-Jun interacted detectably with VP16-AD-ER-alpha only after treatment with estradiol (E2). Fig. 7A. GAL4-DBD-c-Jun did not interact detectably with the mutant VP16-AD-ER-alpha[1-249,304-595] (VP16-AD-ER241G). Fig. 7A. GAL4-DBD-c-Jun did not interact detectably with VP16-AD-ER-alpha after treatment with estradiol (E2), 4-hydroxytamoxifen (OHT) or ICI164,384 (ICI). Fig. 7A. |
| Detection Method | two-hybrid-test |
| Source | BIND |
  ||


---

|  |  |
| --- | --- |
 Reference:5 || PMID | 11477071 |
| SourceID | 183847 |
| Species | Human |
| Experimental Condition | in-vivo |
| Description | An interaction between human ER-alpha and mouse c-Jun was demonstrated by co-immunoprecipitation. ER-alpha was immunoprecipitated from extracts of COS cells transfected with ER-alpha (HEGO) and c-Jun using anti-ER-alpha and precipitates were resolved by SDS-PAGE. c-Jun was detected by western blot using anti-c-Jun. Fig. 7B. c-Jun did not co-immunoprecipitate with the mutant ER-alpha[1-249,304-595] (ER241G). Fig. 7B. |
| Detection Method | immunoprecipitation |
| Source | BIND |
  ||


---

|  |  |
| --- | --- |
 Reference:6 || PMID | 11477071 |
| Species | Human |
| MINT interaction detection method | coimmunoprecipitation |
| Source | MINT |
  ||


---

|  |  |
| --- | --- |
 Reference:7 || PMID | 11477071 |
| Species | Human |
| MINT interaction detection method | beta lactamase complementation |
| Source | MINT |
  ||


---

|  |  |
| --- | --- |
 Reference:8 || PMID | 11477071 |
| Species | Human |
| MINT interaction detection method | pull down |
| Source | MINT |
  ||


---

|  |  |
| --- | --- |
 Reference:9 || PMID | 11477071 |
| Species | Human |
| MINT interaction detection method | pull down |
| Source | MINT |
  ||


---

|  |  |
| --- | --- |
 Reference:10 || PMID | 11477071 |
| Species | Human |
| MINT interaction detection method | beta lactamase complementation |
| Source | MINT |
  ||


---

|  |  |
| --- | --- |
 Reference:11 || PMID | 11477071 |
| Species | Human |
| MINT interaction detection method | coimmunoprecipitation |
| Source | MINT |
  ||


---

|  |  |
| --- | --- |
 Reference:12 || PMID | 11477071 |
| Species | Human |
| MINT interaction detection method | pull down |
| Source | MINT |
  ||


---

|  |  |
| --- | --- |
 Reference:13 || PMID | 11477071 |
| Species | Human |
| MINT interaction detection method | beta lactamase complementation |
| Source | MINT |
  ||


---

|  |  |
| --- | --- |
 Reference:14 || Sentence | An estrogen receptor-alpha/p300 complex activates the BRCA-1 promoter at an AP-1 site that binds Jun/Fos transcription factors: repressive effects of p53 on BRCA-1 transcription. |
| Year | 2005 |
| PMID | 16229810 |
| Species | Human |
| Journal | Neoplasia |
| RefScore | 0 |
| Source | PArchNLP |
  ||


---

|  |  |
| --- | --- |
 Reference:15 || Sentence | Estrogen receptor inhibits c-Jun-dependent stress-induced cell death by binding and modifying c-Jun activity in human breast cancer cells. |
| Year | 2004 |
| PMID | 14638681 |
| Species | Human |
|  | Mouse |
| Journal | J Biol Chem |
| RefScore | 2 |
| Source | PArchNLP |
  ||


---

|  |  |
| --- | --- |
 Reference:16 || Sentence | This is because ER binds c-Jun in breast cancer cells, stress treatment further increases the ER-bound phosphorylated c-Jun, and the c-Jun binding-deficient ER mutant fails to protect stress-induced cell death. |
| Year | 2004 |
| PMID | 14638681 |
| Species | Human |
|  | Mouse |
| Journal | J Biol Chem |
| RefScore | 0 |
| Source | PArchNLP |
  ||


---

|  |  |
| --- | --- |
 Reference:17 || Sentence | We further show that the effect of c-jun requires its DNA-binding domain and that c-jun interacts with Sf-1 and ERalpha and exerts synergistic effects on promoter activity with Sf-1, ERalpha, and Pitx1. |
| PMID | 16627584 |
| Year | 2006 |
| Species | Rat |
| Journal | Endocrinology |
| RefScore | 1 |
| Source | PArchNLP |
  |


---

|  |  |
| --- | --- |
