# Supplementary material for: Dynamic transcriptomic profiles of zebrafish gills in response to zinc supplementation
Source: BMC Genomics. 2010 Oct 11;11:553. doi: 10.1186/1471-2164-11-553 (PMC3091702; doi:10.1186/1471-2164-11-553)
Supplement: Additional file 2 — Interactive Direct Interaction Network representing the molecular interactions between zinc, copper, iron, calcium and proteins encoded by transcripts changed by zinc supplementation. Mini web-site containing index.html and hyperlinked pages in subdirectory describing a Direct Interaction Network automatically generated based on curated interactions contained within the proprietary PathwayArchitect database. Ovals represent proteins and the circles symbolize metal ions. Objects are coloured by their abundance in zebrafish at the time-point they were significantly different from the control is a scale from -4 fold (dark green) to +4 fold (dark red). Where significant differences were found at more than one time-point, the colour overlay shows expression at the first instance. Dark blue squares denote 'binding', and light blue squares 'expression'; green squares stand for 'regulation', green diamonds for 'metabolism', and green circles for 'promoter binding'. Arrow heads indicate directionality of the interaction where annotated. All nodes and edges can be further interrogated by selecting the relative area of the image. [file 1471-2164-11-553-S2.zip › PathwayArchitect Zn xs DIN/113666.html]

# PROTEIN: APOB

|  |  |
| --- | --- |
| Name | APOB |
| Type | PROTEIN |
| Description | apolipoprotein B (including Ag(x) antigen) |
| Note | Apolipoprotein B (ApoB) is the main apolipoprotein of chylomicrons and low density lipoproteins (LDL). The protein occurs in the plasma in 2 main isoforms, apoB-48 and apoB-100. The first is synthesized exclusively by the gut, the second by the liver. The intestinal (B-48) and hepatic (B-100) forms of apoB are coded by a single gene and by a single mRNA transcript larger than 16 kb. The 2 proteins share a common amino terminal sequence. From structural studies, it is thought that apoB-48 represents the amino-terminal 47% of apoB-100 and that the carboxyl terminus of apoB-48 is in the vicinity of residue 2151 of mature apoB-100. Apolipoprotein B-48, a shortened form of apoB-100 lacking the LDL-receptor region, is a product generated when a stop codon (UAA) at residue 2180 is created by RNA editing. |
| Alias | Aa1064 |
|  | AI315052 |
|  | APOB |
|  | Ac1-060 |
|  | apolipoprotein B |
|  | apolipoprotein B PI |
|  | apolipoprotein B48 |
|  | apoB-48 |
|  | apob-100 |
|  | Apo B-100 |
|  | FLDB |
|  | apoB-100 |
|  | apob-48 |


---

|  |  |
| --- | --- |
| GO Component | extracellular region |
|  | endoplasmic reticulum |
|  | soluble fraction |
|  | microsome |
|  | chylomicron |


---

|  |  |
| --- | --- |
| GO ID | GO:0005783 |
|  | GO:0006642 |
|  | GO:0008203 |
|  | GO:0006869 |
|  | GO:0005102 |
|  | GO:0030301 |
|  | GO:0005319 |
|  | GO:0008202 |
|  | GO:0042627 |
|  | GO:0005576 |
|  | GO:0008201 |
|  | GO:0005792 |
|  | GO:0007165 |
|  | GO:0005625 |
|  | GO:0008015 |
|  | GO:0006629 |


---

|  |  |
| --- | --- |
| MIM | MIM:107730 |
|  | MIM:144010 |


---

|  |  |
| --- | --- |
| Connectivity | 1178 |


---

|  |  |
| --- | --- |
| Entrez ID | 238055 |
|  | 54225 |
|  | 338 |


---

|  |  |
| --- | --- |
| Agilent ID | A\_51\_P470542 |
|  | A\_44\_P552692 |
|  | A\_14\_P109396 |
|  | A\_44\_P402339 |
|  | A\_51\_P413088 |
|  | A\_43\_P15626 |
|  | A\_44\_P553791 |
|  | A\_44\_P219789 |
|  | A\_51\_P436689 |
|  | A\_14\_P117773 |
|  | A\_51\_P436690 |
|  | A\_23\_P79591 |
|  | A\_44\_P402346 |
|  | A\_14\_P108104 |
|  | A\_14\_P201934 |
|  | A\_14\_P200977 |
|  | A\_53\_P177034 |
|  | A\_52\_P441070 |
|  | A\_51\_P380650 |


---

|  |  |
| --- | --- |
| Cellular Localization | Endoplasmic reticulum |
|  | Extracellular region |
|  | Cytoplasm |
|  | Organelle |
|  | Cell |


---

|  |  |
| --- | --- |
| Pathway | Atherosclerosis |
|  | Zn xs inventory |
|  | Zn xs DIN |


---

|  |  |
| --- | --- |
| GO Process | triacylglycerol mobilization |
|  | signal transduction |
|  | cholesterol metabolism |
|  | cholesterol transport |
|  | lipid metabolism |
|  | lipid transport |
|  | steroid metabolism |
|  | circulation |


---

|  |  |
| --- | --- |
| UniGene | Hs.120759 |
|  | Mm.221239 |
|  | Rn.33815 |


---

|  |  |
| --- | --- |
| Affymetrix Probeset ID | 1371157\_at |
|  | 1388190\_at |
|  | 1455593\_at |
|  | 1457554\_at |
|  | 205108\_s\_at |
|  | 223579\_s\_at |
|  | 240511\_at |
|  | 261\_s\_at |
|  | 35332\_at |
|  | 76265\_at |
|  | 96792\_at |
|  | g4502152\_3p\_a\_at |
|  | g7770246\_3p\_s\_at |
|  | Hs.120759.0.A1\_3p\_at |
|  | M14952mRNA\_at |
|  | M19828\_s\_at |
|  | M21842\_at |
|  | M27440\_at |
|  | Msa.19808.0\_s\_at |
|  | Msa.20048.0\_s\_at |
|  | rc\_AA997806\_at |
|  | U53873cds\_at |
|  | RC\_H82966\_s\_at |
|  | RC\_R06764\_s\_at |
|  | TC23462\_at |


---

|  |  |
| --- | --- |
| GO Function | heparin binding |
|  | lipid transporter activity |
|  | receptor binding |


---

|  |  |
| --- | --- |
| Nucleotide | M21842 |
|  | M18471 |
|  | AY318958 |
|  | M36676 |
|  | AB208846 |
|  | AK147540 |
|  | BC051278 |
|  | M27440 |
|  | M12681 |
|  | AY321317 |
|  | J04838 |
|  | M17367 |
|  | M17779 |
|  | M10374 |
|  | M15053 |
|  | M14081 |
|  | M18036 |
|  | M31030 |
|  | X04506 |
|  | X55969 |
|  | M12413 |
|  | M11227 |
|  | X15191 |
|  | X04868 |
|  | M19828 |
|  | M14162 |
|  | XM\_137955 |
|  | X03325 |
|  | X04867 |
|  | M14952 |
|  | AY324608 |
|  | AI315052 |
|  | AK160920 |
|  | X03324 |
|  | AF187728 |
|  | K03175 |
|  | X04870 |
|  | M19734 |
|  | AL118236 |
|  | BC038263 |
|  | AC115619 |
|  | M15421 |
|  | NM\_019287 |
|  | M12480 |
|  | NM\_000384 |
|  | M23049 |
|  | AC010872 |
|  | J02610 |
|  | M35186 |
|  | X03045 |
|  | X04869 |
|  | X04714 |
|  | X03326 |
|  | U53873 |
|  | BC028880 |


---

|  |  |
| --- | --- |
| Protein | AAA51756 |
|  | AAB60718 |
|  | AAB04636 |
|  | CAA28420 |
|  | AAA51750 |
|  | AAA51753 |
|  | AAA35549 |
|  | AAA51741 |
|  | AAA40752 |
|  | CAA27043 |
|  | AAP85369 |
|  | NP\_062160 |
|  | AAX93246 |
|  | AAA51751 |
|  | NP\_000375 |
|  | CAA39440 |
|  | CAA27045 |
|  | AAH38263 |
|  | AAA37246 |
|  | AAA51755 |
|  | AAA74690 |
|  | CAA28561 |
|  | AAA53374 |
|  | XP\_137955 |
|  | AAA51759 |
|  | AAA51758 |
|  | AAA35541 |
|  | AAA51754 |
|  | CAA28560 |
|  | AAA98613 |
|  | CAA27044 |
|  | CAA33265 |
|  | AAP72970 |
|  | P04114 |
|  | AAX88848 |
|  | AAA40753 |
|  | AAA51742 |
|  | BAE27983 |
|  | CAA26850 |
|  | AAA35548 |
|  | AAA35544 |
|  | CAA28559 |
|  | CAA28191 |
|  | BAD92083 |
|  | AAA53373 |
|  | AAA40751 |
|  | AAA51752 |
|  | AAH51278 |
|  | AAP86249 |
|  | AAB00481 |
|  | CAA28558 |


---

|  |  |
| --- | --- |
| Organism | Mammal |


---

|  |  |
| --- | --- |
| Location | chromosome 12, 12 A1.1 (Mus musculus) |
|  | chromosome 6, 6q14 (Rattus norvegicus) |
|  | chromosome 2, 2p24-p23 (Homo sapiens) |


---

|  |  |
| --- | --- |
