# Supplementary material for: Dynamic transcriptomic profiles of zebrafish gills in response to zinc supplementation
Source: BMC Genomics. 2010 Oct 11;11:553. doi: 10.1186/1471-2164-11-553 (PMC3091702; doi:10.1186/1471-2164-11-553)
Supplement: Additional file 2 — Interactive Direct Interaction Network representing the molecular interactions between zinc, copper, iron, calcium and proteins encoded by transcripts changed by zinc supplementation. Mini web-site containing index.html and hyperlinked pages in subdirectory describing a Direct Interaction Network automatically generated based on curated interactions contained within the proprietary PathwayArchitect database. Ovals represent proteins and the circles symbolize metal ions. Objects are coloured by their abundance in zebrafish at the time-point they were significantly different from the control is a scale from -4 fold (dark green) to +4 fold (dark red). Where significant differences were found at more than one time-point, the colour overlay shows expression at the first instance. Dark blue squares denote 'binding', and light blue squares 'expression'; green squares stand for 'regulation', green diamonds for 'metabolism', and green circles for 'promoter binding'. Arrow heads indicate directionality of the interaction where annotated. All nodes and edges can be further interrogated by selecting the relative area of the image. [file 1471-2164-11-553-S2.zip › PathwayArchitect Zn xs DIN/1145346.html]

# REGULATION:

|  |  |
| --- | --- |
| Type | REGULATION |
| Effect | None |


---

|  |  |
| --- | --- |
| Score | 0 |


---

|  |  |
| --- | --- |
| Reference Count | 10 |


---

|  |  |
| --- | --- |
| Mechanism | Unknown |


---

|  |  |
| --- | --- |
| Reference:0 || Sentence | "Recent studies suggest that the transferrin receptor mediates the intracellular delivery and transport of iron bound to transferrin in the CNS." |
| PMID | 1915737 |
| Year | 1991 |
| Species | Human |
|  | Mouse |
| Journal | Exp Neurol |
| RefScore | 1 |
| Source | PArchNLP |
  |
|


---

|  |  |
| --- | --- |
 Reference:1 || Sentence | "Transferrin (TF) is a plasma protein that transports and is regulated by iron." |
| PMID | 2376597 |
| Year | 1990 |
| Species | Human |
|  | Mouse |
| Journal | J Biol Chem |
| RefScore | 1 |
| Source | PArchNLP |
  ||


---

|  |  |
| --- | --- |
 Reference:2 || Sentence | "Transgenic mouse lines carrying human TF chimeric genes will be useful models for analyzing the regulation of human transferrin by iron and for determining the molecular basis of transferrin regulation throughout mammalian development into the aging process." |
| PMID | 2376597 |
| Year | 1990 |
| Species | Human |
|  | Mouse |
| Journal | J Biol Chem |
| RefScore | 1 |
| Source | PArchNLP |
  ||


---

|  |  |
| --- | --- |
 Reference:3 || Sentence | "Results described here indicate that transferrin, like ferritin, is regulated by iron at the level of translation." |
| PMID | 8490018 |
| Year | 1993 |
| Species | Human |
|  | Mouse |
| Journal | Biochemistry |
| RefScore | 1 |
| Source | PArchNLP |
  ||


---

|  |  |
| --- | --- |
 Reference:4 || Sentence | "Subsequently, iron may be linked to brain transferrin synthesized within oligodendrocytes and choroid plexus epithelial cells followed by a concomitant uptake of iron-transferrin in neurons expressing transferrin receptors." |
| PMID | 8930792 |
| Year | 1996 |
| Species | Mouse |
| Journal | J Comp Neurol |
| RefScore | 1 |
| Source | PArchNLP |
  ||


---

|  |  |
| --- | --- |
 Reference:5 || Sentence | "In order to better understand the cellular delivery of iron from serum transferrin (Tf), we compared iron release from receptor-bound and free Tf." |
| PMID | 2022630 |
| Year | 1991 |
| Species | Mouse |
|  | Human |
| Journal | J Biol Chem |
| RefScore | 1 |
| Source | PArchNLP |
  ||


---

|  |  |
| --- | --- |
 Reference:6 || Sentence | DMT1 is expressed at the duodenal brush border where it controls uptake of dietary iron and is present at the plasma membrane and in recycling endosomes of most cells, where it is necessary for acquisition of transferrin-associated iron. |
| Year | 2005 |
| PMID | 16142913 |
| Species | Mouse |
| Journal | Biochemistry |
| RefScore | 0 |
| Source | PArchNLP |
  ||


---

|  |  |
| --- | --- |
 Reference:7 || Sentence | OBJECTIVE: To confirm the increase in non-transferrin bound iron (NTBI) after packed red cell (PRC) transfusion and to evaluate the association with increased oxidative stress in preterm infants. |
| Year | 2004 |
| PMID | 15321958 |
| Species | Human |
| Journal | Arch Dis Child Fetal Neonatal Ed |
| RefScore | 0 |
| Source | PArchNLP |
  ||


---

|  |  |
| --- | --- |
 Reference:8 || Sentence | The H(+) -coupled divalent metal-ion transporter DMT1 serves as both the primary entry point for iron into the body (intestinal brush-border uptake) and the route by which transferrin-associated iron is mobilized from endosomes to cytosol in erythroid precursors and other cells. |
| PMID | 16091957 |
| Year | 2006 |
| Species | Rat |
| Journal | Pflugers Arch |
| RefScore | 1 |
| Source | PArchNLP |
  ||


---

|  |  |
| --- | --- |
 Reference:9 || Sentence | The cleavage results in the release of iron, a regulator of transferrin, ferritin, and nitric oxide (NO) synthase gene expression. |
| PMID | 16467964 |
| Year | 2006 |
| Species | Rat |
| Journal | J Neural Transm |
| RefScore | 1 |
| Source | PArchNLP |
  |


---

|  |  |
| --- | --- |
