# Supplementary material for: Dynamic transcriptomic profiles of zebrafish gills in response to zinc supplementation
Source: BMC Genomics. 2010 Oct 11;11:553. doi: 10.1186/1471-2164-11-553 (PMC3091702; doi:10.1186/1471-2164-11-553)
Supplement: Additional file 2 — Interactive Direct Interaction Network representing the molecular interactions between zinc, copper, iron, calcium and proteins encoded by transcripts changed by zinc supplementation. Mini web-site containing index.html and hyperlinked pages in subdirectory describing a Direct Interaction Network automatically generated based on curated interactions contained within the proprietary PathwayArchitect database. Ovals represent proteins and the circles symbolize metal ions. Objects are coloured by their abundance in zebrafish at the time-point they were significantly different from the control is a scale from -4 fold (dark green) to +4 fold (dark red). Where significant differences were found at more than one time-point, the colour overlay shows expression at the first instance. Dark blue squares denote 'binding', and light blue squares 'expression'; green squares stand for 'regulation', green diamonds for 'metabolism', and green circles for 'promoter binding'. Arrow heads indicate directionality of the interaction where annotated. All nodes and edges can be further interrogated by selecting the relative area of the image. [file 1471-2164-11-553-S2.zip › PathwayArchitect Zn xs DIN/1145532.html]

# TRANSPORT:

|  |  |
| --- | --- |
| Type | TRANSPORT |
| Effect | None |


---

|  |  |
| --- | --- |
| Score | 0 |


---

|  |  |
| --- | --- |
| Reference Count | 14 |


---

|  |  |
| --- | --- |
| Mechanism | Unknown |


---

|  |  |
| --- | --- |
| Reference:0 || Sentence | "Transferrin (TF), a major plasma protein, binds and transports ferric iron." |
| PMID | 2079722 |
| Year | 1990 |
| Species | Human |
|  | Mouse |
| Journal | J Neurosci Res |
| RefScore | 0 |
| Source | PArchNLP |
  |
|


---

|  |  |
| --- | --- |
 Reference:1 || Sentence | "We tested the hypothesis that the toxicity of 6-OHDA is caused by its interaction with serum ferric transferrin (Fe-TF) resulting in release of iron." |
| PMID | 10727233 |
| Year | 2000 |
| Species | Mouse |
|  | Human |
| Journal | Biochemistry |
| RefScore | 1 |
| Source | PArchNLP |
  ||


---

|  |  |
| --- | --- |
 Reference:2 || Sentence | "Haemophilus influenzae acquires iron from the iron-transporting glycoprotein transferrin via a receptor-mediated process." |
| PMID | 9004513 |
| Year | 1996 |
| Species | Mouse |
|  | Human |
| Journal | Microbiology |
| RefScore | 0 |
| Source | PArchNLP |
  ||


---

|  |  |
| --- | --- |
 Reference:3 || Sentence | "The iron-transporting serum glycoprotein, transferrin, is necessary for the cell proliferation, morphogenesis, and differentiation of mouse embryonic teeth and kidneys in organ culture." |
| PMID | 3609530 |
| Year | 1987 |
| Species | Mouse |
| Journal | Differentiation |
| RefScore | 0 |
| Source | PArchNLP |
  ||


---

|  |  |
| --- | --- |
 Reference:4 || Sentence | "While free Tf did not release all iron until below pH 4.6, receptor-bound Tf released significantly more iron at mildly acidic pH, with essentially all iron released between pH 5.6 and 6.0." |
| PMID | 2022630 |
| Year | 1991 |
| Species | Mouse |
|  | Human |
| Journal | J Biol Chem |
| RefScore | 2 |
| Source | PArchNLP |
  ||


---

|  |  |
| --- | --- |
 Reference:5 || Sentence | "Transferrin transports iron into cells via the transferrin receptor: thus, iron content of resident cells is low, of peptone- and FCS-elicited cells is intermediate, and of thioglycollate-elicited cells is high." |
| PMID | 1906922 |
| Year | 1991 |
| Species | Mouse |
| Journal | J Exp Med |
| RefScore | 1 |
| Source | PArchNLP |
  ||


---

|  |  |
| --- | --- |
 Reference:6 || Sentence | "OBJECTIVE: This study was undertaken to assess the role of p97 (also known as melanotransferrin) in the transfer of iron into the brain, because the passage of most large molecules is limited by the presence of the blood-brain barrier, including that of the serum iron transporter transferrin." |
| PMID | 14745458 |
| Year | 2003 |
| Species | Mouse |
|  | Human |
| Journal | Microcirculation |
| RefScore | 2 |
| Source | PArchNLP |
  ||


---

|  |  |
| --- | --- |
 Reference:7 || Sentence | "Divalent metal transporter 1 (DMT1) is the major transferrin-independent iron uptake system at the apical pole of intestinal cells, but it may also transport iron across the membrane of acidified endosomes in peripheral tissues." |
| PMID | 11739192 |
| Year | 2001 |
| Species | Mouse |
| Journal | Blood |
| RefScore | 2 |
| Source | PArchNLP |
  ||


---

|  |  |
| --- | --- |
 Reference:8 || Sentence | "The abundant binding of transferrin in areas of active cell proliferation in bell-stage teeth also suggests that transferrin is still needed and used for the transport of iron into proliferating cells." |
| PMID | 3609531 |
| Year | 1987 |
| Species | Mouse |
| Journal | Differentiation |
| RefScore | 1 |
| Source | PArchNLP |
  ||


---

|  |  |
| --- | --- |
 Reference:9 || Sentence | "The addition of either methylamine or ammonium chloride, both known blockers of transferrin-iron release through their lysosomotropic properties, inhibited total iron uptake." |
| PMID | 6465862 |
| Year | 1984 |
| Species | Mouse |
| Journal | Ann Neurol |
| RefScore | 0 |
| Source | PArchNLP |
  ||


---

|  |  |
| --- | --- |
 Reference:10 || Sentence | "The data suggest that neuronal iron transport, much like that in other mammalian tissues, is transferrin mediated and that blockers of transferrin-iron release may be of value in conditions in which there is brain iron overload." |
| PMID | 6465862 |
| Year | 1984 |
| Species | Mouse |
| Journal | Ann Neurol |
| RefScore | 1 |
| Source | PArchNLP |
  ||


---

|  |  |
| --- | --- |
 Reference:11 || Sentence | "Presumably, Tf transports the iron found in Hp oligodendrocytes." |
| PMID | 7636015 |
| Year | 1995 |
| Species | Mouse |
| Journal | J Comp Neurol |
| RefScore | 0 |
| Source | PArchNLP |
  ||


---

|  |  |
| --- | --- |
 Reference:12 || Sentence | Transferrin (TF), a 76-80 kDa glycoprotein, is responsible for the transport of iron to cells within both the fetal and maternal systems, but it does not cross the multiple cell layer barrier of the placenta. |
| Year | 2004 |
| PMID | 15013638 |
| Species | Human |
|  | Rat |
| Journal | Placenta |
| RefScore | 1 |
| Source | PArchNLP |
  ||


---

|  |  |
| --- | --- |
 Reference:13 || Sentence | Recent findings that both rat and human placental cells produce TF indicated that placental TF may function in some manner to transport or regulate iron passage across this barrier. |
| Year | 2004 |
| PMID | 15013638 |
| Species | Human |
|  | Rat |
| Journal | Placenta |
| RefScore | 1 |
| Source | PArchNLP |
  |


---

|  |  |
| --- | --- |
