# Supplementary material for: Dynamic transcriptomic profiles of zebrafish gills in response to zinc supplementation
Source: BMC Genomics. 2010 Oct 11;11:553. doi: 10.1186/1471-2164-11-553 (PMC3091702; doi:10.1186/1471-2164-11-553)
Supplement: Additional file 2 — Interactive Direct Interaction Network representing the molecular interactions between zinc, copper, iron, calcium and proteins encoded by transcripts changed by zinc supplementation. Mini web-site containing index.html and hyperlinked pages in subdirectory describing a Direct Interaction Network automatically generated based on curated interactions contained within the proprietary PathwayArchitect database. Ovals represent proteins and the circles symbolize metal ions. Objects are coloured by their abundance in zebrafish at the time-point they were significantly different from the control is a scale from -4 fold (dark green) to +4 fold (dark red). Where significant differences were found at more than one time-point, the colour overlay shows expression at the first instance. Dark blue squares denote 'binding', and light blue squares 'expression'; green squares stand for 'regulation', green diamonds for 'metabolism', and green circles for 'promoter binding'. Arrow heads indicate directionality of the interaction where annotated. All nodes and edges can be further interrogated by selecting the relative area of the image. [file 1471-2164-11-553-S2.zip › PathwayArchitect Zn xs DIN/115947.html]

# PROTEIN: BAX

|  |  |
| --- | --- |
| Name | BAX |
| Type | PROTEIN |
| Description | BCL2-associated X protein |
| Note | The protein encoded by this gene belongs to the BCL2 protein family. BCL2 family members form hetero- or homodimers and act as anti- or pro-apoptotic regulators that are involved in a wide variety of cellular activities. This protein forms a heterodimer with BCL2, and functions as an apoptotic activator. This protein is reported to interact with, and increase the opening of, the mitochondrial voltage-dependent anion channel (VDAC), which leads to the loss in membrane potential and the release of cytochrome c. The expression of this gene is regulated by the tumor suppressor P53 and has been shown to be involved in P53-mediated apoptosis. Six alternatively spliced transcript variants, which encode different isoforms, have been reported for this gene. |
| Alias | Apoptosis regulator BAX, membrane isoform alpha |
|  | apoptosis regulator BAX |
|  | BAX protein, cytoplasmic isoform gamma |
|  | Apoptosis regulator BAX, cytoplasmic isoform beta |
|  | BAX |
|  | Bax zeta |
|  | BAX protein, cytoplasmic isoform delta |
|  | bax protein |
|  | Bax |


---

|  |  |
| --- | --- |
| GO Component | mitochondrial outer membrane |
|  | cytosol |
|  | integral to membrane |


---

|  |  |
| --- | --- |
| GO ID | GO:0007399 |
|  | GO:0001844 |
|  | GO:0045786 |
|  | GO:0046666 |
|  | GO:0008637 |
|  | GO:0008634 |
|  | GO:0005741 |
|  | GO:0051260 |
|  | GO:0005515 |
|  | GO:0005829 |
|  | GO:0006917 |
|  | GO:0016021 |
|  | GO:0045333 |
|  | GO:0006974 |
|  | GO:0030264 |
|  | GO:0001836 |
|  | GO:0009611 |
|  | GO:0007007 |
|  | GO:0048147 |
|  | GO:0006915 |
|  | GO:0008629 |
|  | GO:0007281 |
|  | GO:0008635 |
|  | GO:0042981 |
|  | GO:0008624 |
|  | GO:0007049 |
|  | GO:0043026 |
|  | GO:0007008 |


---

|  |  |
| --- | --- |
| MIM | MIM:600040 |


---

|  |  |
| --- | --- |
| Connectivity | 1984 |


---

|  |  |
| --- | --- |
| Entrez ID | 12028 |
|  | 24887 |
|  | 581 |


---

|  |  |
| --- | --- |
| Agilent ID | A\_51\_P254534 |
|  | A\_14\_P111079 |
|  | A\_23\_P346311 |
|  | A\_43\_P11800 |
|  | A\_14\_P116143 |
|  | A\_23\_P346309 |
|  | A\_23\_P208706 |
|  | A\_14\_P135790 |
|  | A\_51\_P254541 |
|  | A\_44\_P489512 |


---

|  |  |
| --- | --- |
| Cellular Localization | Cytosol |
|  | Cytoplasm |
|  | Membrane |
|  | Mitochondrion |
|  | Organelle |
|  | Cell |


---

|  |  |
| --- | --- |
| DbXref | Reactome##172753##Apoptosis##http://www.reactome.org/cgi-bin/eventbrowser?DB=gk\_current&ID=172753 |
|  | KEGG pathway##05030##Amyotrophic lateral sclerosis (ALS)##http://www.genome.jp/dbget-bin/show\_pathway?hsa05030+581 |
|  | KEGG pathway##04210##Apoptosis##http://www.genome.jp/dbget-bin/show\_pathway?hsa04210+581 |
|  | KEGG pathway##05030##Amyotrophic lateral sclerosis (ALS)##http://www.genome.jp/dbget-bin/show\_pathway?rno05030+24887 |
|  | Reactome##109581##Apoptosis##http://www.reactome.org/cgi-bin/eventbrowser?DB=gk\_current&ID=109581 |
|  | KEGG pathway##04210##Apoptosis##http://www.genome.jp/dbget-bin/show\_pathway?rno04210+24887 |
|  | KEGG pathway##05030##Amyotrophic lateral sclerosis (ALS)##http://www.genome.jp/dbget-bin/show\_pathway?mmu05030+12028 |
|  | KEGG pathway##01510##Neurodegenerative Disorders##http://www.genome.jp/dbget-bin/show\_pathway?mmu01510+12028 |
|  | KEGG pathway##04210##Apoptosis##http://www.genome.jp/dbget-bin/show\_pathway?mmu04210+12028 |


---

|  |  |
| --- | --- |
| Pathway | P53 Signaling |
|  | Mitochondrial Apoptosis Control |
|  | Breast Cancer |
|  | Apoptosis |
|  | Zn xs inventory |
|  | Zn xs DIN |


---

|  |  |
| --- | --- |
| GO Process | negative regulation of progression through cell cycle |
|  | nuclear fragmentation |
|  | germ cell development |
|  | response to wounding |
|  | caspase activation via cytochrome c |
|  | apoptosis |
|  | release of cytochrome c from mitochondria |
|  | induction of apoptosis |
|  | negative regulation of fibroblast proliferation |
|  | negative regulation of survival gene product activity |
|  | neurogenesis |
|  | outer mitochondrial membrane organization and biogenesis |
|  | protein insertion into mitochondrial membrane during induction of apoptosis |
|  | induction of apoptosis by extracellular signals |
|  | apoptotic mitochondrial changes |
|  | inner mitochondrial membrane organization and biogenesis |
|  | regulation of apoptosis |
|  | response to DNA damage stimulus |
|  | regulation of caspase activation |
|  | cellular respiration |
|  | induction of apoptosis by intracellular signals |
|  | protein homooligomerization |
|  | retinal cell programmed cell death |
|  | cell cycle |
|  | nervous system development |


---

|  |  |
| --- | --- |
| UniGene | Hs.159428 |
|  | Rn.10668 |
|  | Mm.19904 |


---

|  |  |
| --- | --- |
| Affymetrix Probeset ID | 1369122\_at |
|  | 1416837\_at |
|  | 1997\_s\_at |
|  | 1998\_i\_at |
|  | 2065\_s\_at |
|  | 2066\_at |
|  | 2067\_f\_at |
|  | 208478\_s\_at |
|  | 211833\_s\_at |
|  | 217029\_at |
|  | 93536\_at |
|  | g841237\_3p\_a\_at |
|  | Msa.126.0\_s\_at |
|  | rc\_AI044221\_at |
|  | S76511\_s\_at |
|  | U59184\_at |
|  | TC37268\_at |
|  | U49729\_at |


---

|  |  |
| --- | --- |
| GO Function | protein binding |


---

|  |  |
| --- | --- |
| Nucleotide | AK149994 |
|  | NM\_138764 |
|  | AK013298 |
|  | NM\_138761 |
|  | L22472 |
|  | AF339055 |
|  | AJ417988 |
|  | L22475 |
|  | AF007826 |
|  | U19599 |
|  | AF008195 |
|  | U49729 |
|  | AF020360 |
|  | BC014175 |
|  | AK159233 |
|  | NM\_138763 |
|  | L22473 |
|  | AB029557 |
|  | AY095934 |
|  | NM\_007527 |
|  | AF235993 |
|  | AJ586910 |
|  | BC053380 |
|  | AY217036 |
|  | U59184 |
|  | BM706954 |
|  | BE396495 |
|  | AF339054 |
|  | AJ586909 |
|  | NM\_017059 |
|  | BC018228 |
|  | NM\_138765 |
|  | U32098 |
|  | AF247393 |
|  | NM\_004324 |
|  | NM\_138762 |
|  | L22474 |
|  | S76511 |
|  | AF250190 |
|  | AF008196 |


---

|  |  |
| --- | --- |
| Protein | BAE34919 |
|  | AAD22706 |
|  | P55269 |
|  | NP\_058755 |
|  | BAA82406 |
|  | CAE52909 |
|  | NP\_004315 |
|  | AAO22992 |
|  | Q07812 |
|  | AAH18228 |
|  | Q63690 |
|  | NP\_620116 |
|  | AAC60700 |
|  | AAA75200 |
|  | CAD10744 |
|  | NP\_620117 |
|  | AAF71267 |
|  | AAD01416 |
|  | AAL73334 |
|  | AAH14175 |
|  | AAL73333 |
|  | BAE29222 |
|  | CAE52910 |
|  | AAC50142 |
|  | AAF36411 |
|  | AAD01415 |
|  | AAM34436 |
|  | Q07814 |
|  | NP\_620120 |
|  | AAF82094 |
|  | AAC26327 |
|  | Q07813 |
|  | AAC52998 |
|  | AAA03621 |
|  | BAB28776 |
|  | NP\_031553 |
|  | AAH53380 |
|  | NP\_620119 |
|  | Q07815 |
|  | NP\_620118 |
|  | AAA03619 |
|  | AAA03622 |
|  | AAA03620 |


---

|  |  |
| --- | --- |
| Organism | Mammal |


---

|  |  |
| --- | --- |
| Location | 7 23.0 cM (Mus musculus) |
|  | chromosome 1, 1q31.2 (Rattus norvegicus) |
|  | chromosome 19, 19q13.3-q13.4 (Homo sapiens) |
|  | chromosome 7, 7 23.0 cM, 7 B5 (Mus musculus) |


---

|  |  |
| --- | --- |
