# Supplementary material for: Dynamic transcriptomic profiles of zebrafish gills in response to zinc supplementation
Source: BMC Genomics. 2010 Oct 11;11:553. doi: 10.1186/1471-2164-11-553 (PMC3091702; doi:10.1186/1471-2164-11-553)
Supplement: Additional file 2 — Interactive Direct Interaction Network representing the molecular interactions between zinc, copper, iron, calcium and proteins encoded by transcripts changed by zinc supplementation. Mini web-site containing index.html and hyperlinked pages in subdirectory describing a Direct Interaction Network automatically generated based on curated interactions contained within the proprietary PathwayArchitect database. Ovals represent proteins and the circles symbolize metal ions. Objects are coloured by their abundance in zebrafish at the time-point they were significantly different from the control is a scale from -4 fold (dark green) to +4 fold (dark red). Where significant differences were found at more than one time-point, the colour overlay shows expression at the first instance. Dark blue squares denote 'binding', and light blue squares 'expression'; green squares stand for 'regulation', green diamonds for 'metabolism', and green circles for 'promoter binding'. Arrow heads indicate directionality of the interaction where annotated. All nodes and edges can be further interrogated by selecting the relative area of the image. [file 1471-2164-11-553-S2.zip › PathwayArchitect Zn xs DIN/115955.html]

# PROTEIN: RXRB

|  |  |
| --- | --- |
| Name | RXRB |
| Type | PROTEIN |
| Description | retinoid X receptor, beta |
| Note | This gene encodes a member of the retinoid X receptor (RXR) family of nuclear receptors which are involved in mediating the effects of retinoic acid (RA). This receptor forms homodimers with the retinoic acid, thyroid hormone, and vitamin D receptors, increasing both DNA binding and transcriptional function on their respective response elements. The gene lies within the major histocompatibility complex (MHC) class II region on chromosome 6. An alternatively spliced transcript variant has been described, but its full length sequence has not been determined. |
| Alias | Nr2b2 |
|  | Rcor-1 |
|  | Retinoic acid receptor, beta |
|  | RXR-beta |
|  | MHC class I regulatory element binding protein H-2RIIBP |
|  | Nuclear receptor coregulator 1 |
|  | H-2RIIBP |
|  | nuclear receptor co-regulator 1 |
|  | Retinoid X receptor beta |
|  | Retinoic acid receptor beta |
|  | NR2B2 |
|  | AL023085 |
|  | MGC1831 |
|  | RCoR-1 |
|  | Rub |
|  | MHC class I promoter binding protein |
|  | DAUDI6 |
|  | Fragment |
|  | Rxrb |
|  | RXRB |


---

|  |  |
| --- | --- |
| GO Component | nucleus |


---

|  |  |
| --- | --- |
| GO ID | GO:0003707 |
|  | GO:0042809 |
|  | GO:0003677 |
|  | GO:0046966 |
|  | GO:0006355 |
|  | GO:0046982 |
|  | GO:0005515 |
|  | GO:0005496 |
|  | GO:0003700 |
|  | GO:0005634 |
|  | GO:0042974 |
|  | GO:0004872 |
|  | GO:0004879 |
|  | GO:0030375 |
|  | GO:0004886 |
|  | GO:0046872 |
|  | GO:0003713 |
|  | GO:0006350 |


---

|  |  |
| --- | --- |
| MIM | MIM:180246 |


---

|  |  |
| --- | --- |
| Connectivity | 271 |


---

|  |  |
| --- | --- |
| Entrez ID | 361801 |
|  | 6257 |
|  | 20182 |


---

|  |  |
| --- | --- |
| Agilent ID | A\_53\_P158243 |
|  | A\_51\_P314830 |
|  | A\_14\_P112536 |
|  | A\_44\_P402675 |
|  | A\_32\_P463219 |
|  | A\_23\_P59179 |
|  | A\_52\_P519689 |


---

|  |  |
| --- | --- |
| Cellular Localization | Nucleus |
|  | Organelle |
|  | Cell |


---

|  |  |
| --- | --- |
| DbXref | KEGG pathway##04920##Adipocytokine signaling pathway##http://www.genome.jp/dbget-bin/show\_pathway?mmu04920+20182 |


---

|  |  |
| --- | --- |
| Pathway | Zn xs inventory |
|  | Zn xs DIN |


---

|  |  |
| --- | --- |
| GO Process | regulation of transcription, DNA-dependent |
|  | transcription |


---

|  |  |
| --- | --- |
| UniGene | Mm.1243 |
|  | Hs.388034 |
|  | Rn.137466 |


---

|  |  |
| --- | --- |
| Affymetrix Probeset ID | 102398\_at |
|  | 1362\_s\_at |
|  | 1416990\_at |
|  | 209148\_at |
|  | 215098\_at |
|  | 215099\_s\_at |
|  | 37763\_at |
|  | g12654658\_3p\_at |
|  | Hs.79372.1.A1\_3p\_a\_at |
|  | M81766\_at |
|  | M84820\_s\_at |
|  | X63522\_s\_at |
|  | X65463\_at |
|  | x66224\_s\_at |
|  | 1389051\_at |
|  | TC40404\_s\_at |


---

|  |  |
| --- | --- |
| GO Function | thyroid hormone receptor coactivator activity |
|  | ligand-dependent nuclear receptor activity |
|  | retinoic acid receptor binding |
|  | retinoid-X receptor activity |
|  | protein heterodimerization activity |
|  | DNA binding |
|  | vitamin D receptor binding |
|  | transcription coactivator activity |
|  | protein binding |
|  | transcription factor activity |
|  | steroid binding |
|  | steroid hormone receptor activity |
|  | thyroid hormone receptor binding |
|  | receptor activity |
|  | metal ion binding |


---

|  |  |
| --- | --- |
| Nucleotide | X72017 |
|  | AK013718 |
|  | AL844527 |
|  | AF120161 |
|  | X66224 |
|  | X63522 |
|  | AL662824 |
|  | M84818 |
|  | M81766 |
|  | X70190 |
|  | BC049773 |
|  | AF065396 |
|  | BC019432 |
|  | BM982876 |
|  | BX883042 |
|  | NM\_021976 |
|  | BT007280 |
|  | BC099776 |
|  | M26804 |
|  | X65463 |
|  | NM\_011306 |
|  | AL645940 |
|  | AK140371 |
|  | D21831 |
|  | X66424 |
|  | AL031228 |
|  | NM\_206849 |
|  | AB209244 |
|  | BC001167 |
|  | M84820 |
|  | AF100956 |
|  | AK159671 |


---

|  |  |
| --- | --- |
| Protein | BAE35275 |
|  | AAC69904 |
|  | P28704 |
|  | BAA04858 |
|  | AAA60293 |
|  | P28702 |
|  | CAA50896 |
|  | CAA46456 |
|  | AAH19432 |
|  | AAC18599 |
|  | CAI18066 |
|  | CAI17612 |
|  | BAA04859 |
|  | CAA45087 |
|  | NP\_996731 |
|  | AAD13794 |
|  | NP\_035436 |
|  | CAA46963 |
|  | CAI95622 |
|  | AAP35944 |
|  | CAI18064 |
|  | NP\_068811 |
|  | BAD92481 |
|  | AAA40081 |
|  | AAA37772 |
|  | AAA42025 |
|  | AAH49773 |
|  | AAH99776 |
|  | CAI17614 |
|  | CAI41837 |
|  | AAH01167 |
|  | CAI41836 |
|  | CAA20239 |
|  | CAE83933 |
|  | BAE24359 |
|  | P49743 |


---

|  |  |
| --- | --- |
| Organism | Mammal |


---

|  |  |
| --- | --- |
| Location | chromosome 6, 6p21.3 (Homo sapiens) |
|  | chromosome 17, 17 18.49 cM, 17 B1 (Mus musculus) |
|  | chromosome 20, 20p12 (Rattus norvegicus) |
|  | 17 18.49 cM (Mus musculus) |


---

|  |  |
| --- | --- |
