# Supplementary material for: Dynamic transcriptomic profiles of zebrafish gills in response to zinc supplementation
Source: BMC Genomics. 2010 Oct 11;11:553. doi: 10.1186/1471-2164-11-553 (PMC3091702; doi:10.1186/1471-2164-11-553)
Supplement: Additional file 2 — Interactive Direct Interaction Network representing the molecular interactions between zinc, copper, iron, calcium and proteins encoded by transcripts changed by zinc supplementation. Mini web-site containing index.html and hyperlinked pages in subdirectory describing a Direct Interaction Network automatically generated based on curated interactions contained within the proprietary PathwayArchitect database. Ovals represent proteins and the circles symbolize metal ions. Objects are coloured by their abundance in zebrafish at the time-point they were significantly different from the control is a scale from -4 fold (dark green) to +4 fold (dark red). Where significant differences were found at more than one time-point, the colour overlay shows expression at the first instance. Dark blue squares denote 'binding', and light blue squares 'expression'; green squares stand for 'regulation', green diamonds for 'metabolism', and green circles for 'promoter binding'. Arrow heads indicate directionality of the interaction where annotated. All nodes and edges can be further interrogated by selecting the relative area of the image. [file 1471-2164-11-553-S2.zip › PathwayArchitect Zn xs DIN/116679.html]

# PROTEIN: VDAC1

|  |  |
| --- | --- |
| Name | VDAC1 |
| Type | PROTEIN |
| Description | voltage-dependent anion channel 1 |
| Note | Large diameter (2.5 3 nm) channel, permeable to ions and small molecules; molecular component of Ca2+ transport across the mitochondrial membranes [RGD] |
| Alias | rVDAC1 |
|  | Porin 31HL |
|  | AL033343 |
|  | VDAC1 |
|  | VDAC-1 |
|  | VDAC |
|  | Plasmalemmal porin |
|  | mVDAC5 |
|  | PORIN |
|  | Porin 31HM |
|  | hVDAC1 |
|  | PORIN-31-HL |
|  | Vdac5 |
|  | MGC111064 |
|  | Outer mitochondrial membrane protein porin 1 |
|  | mVDAC1 |
|  | Vdac1 |


---

|  |  |
| --- | --- |
| GO Component | mitochondrial outer membrane |
|  | extracellular space |
|  | mitochondrion |
|  | integral to membrane |
|  | outer membrane |
|  | mitochondrial inner membrane |
|  | membrane |


---

|  |  |
| --- | --- |
| GO ID | GO:0016020 |
|  | GO:0005615 |
|  | GO:0019867 |
|  | GO:0015482 |
|  | GO:0005741 |
|  | GO:0005253 |
|  | GO:0016021 |
|  | GO:0015283 |
|  | GO:0005739 |
|  | GO:0007270 |
|  | GO:0006810 |
|  | GO:0006851 |
|  | GO:0006820 |
|  | GO:0008308 |
|  | GO:0007612 |
|  | GO:0006811 |
|  | GO:0001662 |
|  | GO:0008632 |
|  | GO:0015288 |
|  | GO:0005743 |
|  | GO:0007268 |


---

|  |  |
| --- | --- |
| MIM | MIM:604492 |


---

|  |  |
| --- | --- |
| Connectivity | 270 |


---

|  |  |
| --- | --- |
| Entrez ID | 83529 |
|  | 7416 |
|  | 22333 |


---

|  |  |
| --- | --- |
| Agilent ID | A\_53\_P114264 |
|  | A\_14\_P133152 |
|  | A\_44\_P213415 |
|  | A\_44\_P505885 |
|  | A\_44\_P237886 |
|  | A\_52\_P909310 |
|  | A\_42\_P758471 |
|  | A\_32\_P163169 |
|  | A\_23\_P144816 |
|  | A\_43\_P14213 |
|  | A\_14\_P138698 |
|  | A\_51\_P130459 |
|  | A\_53\_P148735 |


---

|  |  |
| --- | --- |
| Cellular Localization | Mitochondrion |
|  | Membrane |
|  | Cytoplasm |
|  | Cell |
|  | Extracellular region |
|  | Organelle |


---

|  |  |
| --- | --- |
| DbXref | KEGG pathway##04020##Calcium signaling pathway##http://www.genome.jp/dbget-bin/show\_pathway?mmu04020+22333 |
|  | KEGG pathway##04020##Calcium signaling pathway##http://www.genome.jp/dbget-bin/show\_pathway?rno04020+83529 |
|  | KEGG pathway##04020##Calcium signaling pathway##http://www.genome.jp/dbget-bin/show\_pathway?hsa04020+7416 |


---

|  |  |
| --- | --- |
| Pathway | Zn xs inventory |
|  | Zn xs DIN |


---

|  |  |
| --- | --- |
| GO Process | behavioral fear response |
|  | transport |
|  | mitochondrial calcium ion transport |
|  | ion transport |
|  | apoptotic program |
|  | nerve-nerve synaptic transmission |
|  | synaptic transmission |
|  | anion transport |
|  | learning |


---

|  |  |
| --- | --- |
| UniGene | Rn.115392 |
|  | Rn.54594 |
|  | Hs.519320 |
|  | Mm.3555 |
|  | Hs.202085 |


---

|  |  |
| --- | --- |
| Affymetrix Probeset ID | 1367706\_at |
|  | 1386909\_a\_at |
|  | 1415998\_at |
|  | 1437192\_x\_at |
|  | 1437452\_x\_at |
|  | 171033\_r\_at |
|  | 212038\_s\_at |
|  | 217140\_s\_at |
|  | 1436992\_x\_at |
|  | 31608\_g\_at |
|  | 40198\_at |
|  | 49354\_at |
|  | 75551\_at |
|  | 98139\_at |
|  | 1380782\_at |
|  | AA848348\_at |
|  | AF048828\_at |
|  | AF048828\_g\_at |
|  | Hs.149155.2.A1\_3p\_a\_at |
|  | L06132\_at |
|  | Msa.3230.0\_s\_at |
|  | rc\_AI176266\_at |
|  | U30840\_s\_at |
|  | 1437947\_x\_at |
|  | 217139\_at |
|  | 31607\_at |
|  | 66573\_at |
|  | Hs.201553.0.S1\_3p\_at |
|  | 132990\_r\_at |


---

|  |  |
| --- | --- |
| GO Function | apoptogenic cytochrome c release channel activity |
|  | voltage-gated ion-selective channel activity |
|  | anion channel activity |
|  | voltage-gated anion channel porin activity |
|  | porin activity |


---

|  |  |
| --- | --- |
| Nucleotide | AK192648 |
|  | AK122953 |
|  | AK207573 |
|  | BC087657 |
|  | U89987 |
|  | AK189657 |
|  | AK195288 |
|  | AF268467 |
|  | AK217498 |
|  | BC060558 |
|  | BC087573 |
|  | BC071168 |
|  | BC008482 |
|  | AK002740 |
|  | AK219630 |
|  | AK153094 |
|  | AB039662 |
|  | AK169354 |
|  | AK210208 |
|  | AK191450 |
|  | AL645589 |
|  | AK215454 |
|  | AK183628 |
|  | U30840 |
|  | AJ250032 |
|  | AK168672 |
|  | AK169671 |
|  | BC072484 |
|  | AK169160 |
|  | NM\_003374 |
|  | AK167860 |
|  | NM\_011694 |
|  | AK095989 |
|  | AF048828 |
|  | BC092257 |
|  | AF151097 |
|  | AK146239 |
|  | BC090042 |
|  | AK168225 |
|  | L06132 |
|  | AK169282 |
|  | AC005200 |
|  | NM\_031353 |


---

|  |  |
| --- | --- |
| Protein | CAI24939 |
|  | BAE40179 |
|  | AAH92257 |
|  | CAB58127 |
|  | BAE41103 |
|  | AAH60558 |
|  | BAB13473 |
|  | BAE27004 |
|  | P21796 |
|  | Q9Z2L0 |
|  | AAH87657 |
|  | AAD02476 |
|  | AAH72484 |
|  | AAD54939 |
|  | AAF80115 |
|  | NP\_003365 |
|  | Q60932 |
|  | AAC24723 |
|  | BAE31716 |
|  | NP\_112643 |
|  | AAH71168 |
|  | BAE40939 |
|  | BAE40522 |
|  | BAE39878 |
|  | AAA61272 |
|  | AAB47777 |
|  | NP\_035824 |
|  | AAH87573 |
|  | BAE41040 |
|  | AAH90042 |
|  | BAE41292 |
|  | AAH08482 |


---

|  |  |
| --- | --- |
| Organism | Mammal |


---

|  |  |
| --- | --- |
| Location | chromosome 5, 5q31 (Homo sapiens) |
|  | chromosome 11, 11 29.0 cM, 11 B1.3 (Mus musculus) |
|  | chromosome 10, 10q22 (Rattus norvegicus) |
|  | 11 29.0 cM (Mus musculus) |


---

|  |  |
| --- | --- |
