# Supplementary material for: Dynamic transcriptomic profiles of zebrafish gills in response to zinc supplementation
Source: BMC Genomics. 2010 Oct 11;11:553. doi: 10.1186/1471-2164-11-553 (PMC3091702; doi:10.1186/1471-2164-11-553)
Supplement: Additional file 2 — Interactive Direct Interaction Network representing the molecular interactions between zinc, copper, iron, calcium and proteins encoded by transcripts changed by zinc supplementation. Mini web-site containing index.html and hyperlinked pages in subdirectory describing a Direct Interaction Network automatically generated based on curated interactions contained within the proprietary PathwayArchitect database. Ovals represent proteins and the circles symbolize metal ions. Objects are coloured by their abundance in zebrafish at the time-point they were significantly different from the control is a scale from -4 fold (dark green) to +4 fold (dark red). Where significant differences were found at more than one time-point, the colour overlay shows expression at the first instance. Dark blue squares denote 'binding', and light blue squares 'expression'; green squares stand for 'regulation', green diamonds for 'metabolism', and green circles for 'promoter binding'. Arrow heads indicate directionality of the interaction where annotated. All nodes and edges can be further interrogated by selecting the relative area of the image. [file 1471-2164-11-553-S2.zip › PathwayArchitect Zn xs DIN/116835.html]

# PROTEIN: ZIC2

|  |  |
| --- | --- |
| Name | ZIC2 |
| Type | PROTEIN |
| Description | Zic family member 2 (odd-paired homolog, Drosophila) |
| Note | This gene encodes a member of the ZIC family of C2H2-type zinc finger proteins. This protein functions as a transcriptional repressor and may regulate tissue specific expression of dopamine receptor D1. Mutations in this gene cause holoprosencephaly type 5. Holoprosencephaly is the most common structural anomaly of the human brain. A polyhistidine tract polymorphism in this gene may be associated with increased risk of neural tube defects. This gene is closely linked to a gene encoding zinc finger protein of the cerebellum 5, a related family member on chromosome 13. |
| Alias | Zic family member 2 (odd-paired homolog, Drosophila) |
|  | odd-paired homolog |
|  | Zic2 |
|  | Ku |
|  | zinc finger protein of the cerebellum 2 |
|  | Zinc finger protein of the cerebellum 2 |
|  | HPE5 |
|  | ZIC2 |
|  | kumba |
|  | Zic family member 2 (odd-paired Drosophila homolog) |
|  | GENA 29 |


---

|  |  |
| --- | --- |
| GO Component | voltage-gated potassium channel complex |
|  | nucleus |


---

|  |  |
| --- | --- |
| GO ID | GO:0007399 |
|  | GO:0003677 |
|  | GO:0007420 |
|  | GO:0048066 |
|  | GO:0005515 |
|  | GO:0008270 |
|  | GO:0008076 |
|  | GO:0006813 |
|  | GO:0005634 |
|  | GO:0005249 |
|  | GO:0030154 |
|  | GO:0007417 |
|  | GO:0003676 |
|  | GO:0046872 |
|  | GO:0007275 |
|  | GO:0001843 |


---

|  |  |
| --- | --- |
| MIM | MIM:603073 |


---

|  |  |
| --- | --- |
| Connectivity | 129 |


---

|  |  |
| --- | --- |
| Entrez ID | 7546 |
|  | 22772 |


---

|  |  |
| --- | --- |
| Agilent ID | A\_53\_P153084 |
|  | A\_23\_P36972 |
|  | A\_14\_P102721 |
|  | A\_51\_P364592 |
|  | A\_51\_P364596 |
|  | A\_14\_P131633 |


---

|  |  |
| --- | --- |
| Cellular Localization | Nucleus |
|  | Plasma membrane |
|  | Membrane |
|  | Organelle |
|  | Cell |


---

|  |  |
| --- | --- |
| DbXref | KEGG pathway##04340##Hedgehog signaling pathway##http://www.genome.jp/dbget-bin/show\_pathway?mmu04340+22772 |
|  | KEGG pathway##04340##Hedgehog signaling pathway##http://www.genome.jp/dbget-bin/show\_pathway?hsa04340+7546 |


---

|  |  |
| --- | --- |
| Pathway | Hedgehog Signaling |
|  | Zn xs inventory |
|  | Zn xs DIN |


---

|  |  |
| --- | --- |
| GO Process | cell differentiation |
|  | potassium ion transport |
|  | neural tube closure |
|  | brain development |
|  | central nervous system development |
|  | pigmentation during development |
|  | neurogenesis |
|  | development |
|  | nervous system development |


---

|  |  |
| --- | --- |
| UniGene | Mm.257301 |
|  | Mm.308936 |
|  | Hs.369063 |


---

|  |  |
| --- | --- |
| Affymetrix Probeset ID | 129209\_r\_at |
|  | 1421301\_at |
|  | 1458548\_at |
|  | 223642\_at |
|  | 35554\_f\_at |
|  | 35555\_r\_at |
|  | 60440\_at |
|  | 71325\_f\_at |
|  | 71326\_r\_at |
|  | 71328\_f\_at |
|  | 98843\_at |
|  | 98844\_at |
|  | D70848\_s\_at |
|  | g11065969\_3p\_at |
|  | RC\_AA007202\_at |


---

|  |  |
| --- | --- |
| GO Function | protein binding |
|  | voltage-gated potassium channel activity |
|  | DNA binding |
|  | zinc ion binding |
|  | nucleic acid binding |
|  | metal ion binding |


---

|  |  |
| --- | --- |
| Nucleotide | AF188733 |
|  | AK158319 |
|  | D70848 |
|  | AL355338 |
|  | AF193855 |
|  | NM\_009574 |
|  | AK135125 |
|  | AF104902 |
|  | NM\_007129 |
|  | AK051032 |
|  | AK161979 |


---

|  |  |
| --- | --- |
| Protein | NP\_033600 |
|  | AAC96325 |
|  | AAG28409 |
|  | BAA11115 |
|  | CAH70367 |
|  | BAC34504 |
|  | AAG38995 |
|  | Q62520 |
|  | O95409 |
|  | NP\_009060 |


---

|  |  |
| --- | --- |
| Organism | Mammal |


---

|  |  |
| --- | --- |
| Location | 14 62.0 cM (Mus musculus) |
|  | chromosome 13, 13q32 (Homo sapiens) |
|  | chromosome 14, 14 62.0 cM, 14 E5 (Mus musculus) |


---

|  |  |
| --- | --- |
