# Supplementary material for: Dynamic transcriptomic profiles of zebrafish gills in response to zinc supplementation
Source: BMC Genomics. 2010 Oct 11;11:553. doi: 10.1186/1471-2164-11-553 (PMC3091702; doi:10.1186/1471-2164-11-553)
Supplement: Additional file 2 — Interactive Direct Interaction Network representing the molecular interactions between zinc, copper, iron, calcium and proteins encoded by transcripts changed by zinc supplementation. Mini web-site containing index.html and hyperlinked pages in subdirectory describing a Direct Interaction Network automatically generated based on curated interactions contained within the proprietary PathwayArchitect database. Ovals represent proteins and the circles symbolize metal ions. Objects are coloured by their abundance in zebrafish at the time-point they were significantly different from the control is a scale from -4 fold (dark green) to +4 fold (dark red). Where significant differences were found at more than one time-point, the colour overlay shows expression at the first instance. Dark blue squares denote 'binding', and light blue squares 'expression'; green squares stand for 'regulation', green diamonds for 'metabolism', and green circles for 'promoter binding'. Arrow heads indicate directionality of the interaction where annotated. All nodes and edges can be further interrogated by selecting the relative area of the image. [file 1471-2164-11-553-S2.zip › PathwayArchitect Zn xs DIN/118620.html]

# PROTEIN: CTDP1

|  |  |
| --- | --- |
| Name | CTDP1 |
| Type | PROTEIN |
| Description | CTD (carboxy-terminal domain, RNA polymerase II, polypeptide A) phosphatase, subunit 1 |
| Note | This gene encodes a protein which interacts with the carboxy-terminus of transcription initiation factor TFIIF, a transcription factor which regulates elongation as well as initiation by RNA polymerase II. The protein may also represent a component of an RNA polymerase II holoenzyme complex. Alternative splicing of this gene results in two transcript variants encoding 2 different isoforms. |
| Alias | transcription factor IIF-associating CTD phosphatase 1 |
|  | Ctdp1 |
|  | CCFDN |
|  | CTD (carboxy-terminal domina, RNA polymerase II, polypeptide A) phosphatase, subunit 1 |
|  | Congenital cataract, facial dysmorphism, and neuropathy syndrome |
|  | AW553592 |
|  | CTD of POLR2A, phosphatase of, subunit 1 |
|  | serine phosphatase FCP1a |
|  | TFIIF-associating CTD phosphatase |
|  | 4930563P03Rik |
|  | TFIIF-associating CTD phosphatase 1 |
|  | Fcp1 |
|  | FCP1 |


---

|  |  |
| --- | --- |
| GO Component | cellular component unknown |
|  | intracellular |
|  | nucleus |


---

|  |  |
| --- | --- |
| GO ID | GO:0005634 |
|  | GO:0005622 |
|  | GO:0000004 |
|  | GO:0006366 |
|  | GO:0008372 |
|  | GO:0003899 |
|  | GO:0016787 |
|  | GO:0005515 |
|  | GO:0004721 |
|  | GO:0006470 |


---

|  |  |
| --- | --- |
| MIM | MIM:604927 |
|  | MIM:604168 |


---

|  |  |
| --- | --- |
| Connectivity | 42 |


---

|  |  |
| --- | --- |
| Entrez ID | 67655 |
|  | 9150 |


---

|  |  |
| --- | --- |
| Agilent ID | A\_53\_P126341 |
|  | A\_51\_P292951 |
|  | A\_14\_P125783 |
|  | A\_53\_P121647 |
|  | A\_23\_P101111 |
|  | A\_53\_P101080 |
|  | A\_53\_P148858 |
|  | A\_23\_P89727 |
|  | A\_51\_P313467 |


---

|  |  |
| --- | --- |
| Cellular Localization | Nucleus |
|  | Cell |
|  | Organelle |


---

|  |  |
| --- | --- |
| DbXref | Reactome##112386##Pausing and recovery of elongation##http://www.reactome.org/cgi-bin/eventbrowser?DB=gk\_current&ID=112386 |
|  | Reactome##113429##Elongating transcript encounters a lesion in the template##http://www.reactome.org/cgi-bin/eventbrowser?DB=gk\_current&ID=113429 |
|  | Reactome##74159##Transcription##http://www.reactome.org/cgi-bin/eventbrowser?DB=gk\_current&ID=74159 |
|  | Reactome##74160##Gene Expression##http://www.reactome.org/cgi-bin/eventbrowser?DB=gk\_current&ID=74160 |
|  | Reactome##113409##Abortive termination of early transcription elongation by DSIF##http://www.reactome.org/cgi-bin/eventbrowser?DB=gk\_current&ID=113409 |


---

|  |  |
| --- | --- |
| Pathway | Zn xs inventory |
|  | Zn xs DIN |


---

|  |  |
| --- | --- |
| GO Process | biological process unknown |
|  | protein amino acid dephosphorylation |
|  | transcription from RNA polymerase II promoter |


---

|  |  |
| --- | --- |
| UniGene | Mm.312893 |
|  | Hs.465490 |


---

|  |  |
| --- | --- |
| Affymetrix Probeset ID | 1452697\_at |
|  | 160576\_at |
|  | 205035\_at |
|  | 35979\_at |
|  | 83883\_at |
|  | g4758093\_3p\_at |
|  | AA282312\_s\_at |
|  | RC\_W45328\_at |
|  | TC24324\_at |
|  | TC41269\_s\_at |


---

|  |  |
| --- | --- |
| EC Number | EC 3.1.3.16 |


---

|  |  |
| --- | --- |
| GO Function | hydrolase activity |
|  | protein binding |
|  | DNA-directed RNA polymerase activity |
|  | phosphoprotein phosphatase activity |


---

|  |  |
| --- | --- |
| Nucleotide | BC047999 |
|  | AK186350 |
|  | AF081287 |
|  | CR602644 |
|  | BC052576 |
|  | AF154115 |
|  | BC015010 |
|  | AK206583 |
|  | BC052934 |
|  | AK044530 |
|  | AK080231 |
|  | BC053435 |
|  | BC032515 |
|  | AK017665 |
|  | AK156617 |
|  | NM\_048368 |
|  | AK016213 |
|  | NM\_004715 |
|  | BC063447 |
|  | NM\_026295 |


---

|  |  |
| --- | --- |
| Protein | NP\_430255 |
|  | AAH15010 |
|  | AAH52576 |
|  | Q7TSG2 |
|  | Q9Y5B0 |
|  | AAC64549 |
|  | NP\_080571 |
|  | NP\_004706 |
|  | AAH52934 |
|  | AAH63447 |
|  | AAH53435 |
|  | AAD42088 |
|  | BAB30150 |
|  | BAE33777 |


---

|  |  |
| --- | --- |
| Organism | Mammal |


---

|  |  |
| --- | --- |
| Location | 18 54.0 cM (Mus musculus) |
|  | chromosome 18, 18q23 (Homo sapiens) |
|  | chromosome 18, 18 54.0 cM, 18 E3 (Mus musculus) |


---

|  |  |
| --- | --- |
