# Supplementary material for: Dynamic transcriptomic profiles of zebrafish gills in response to zinc supplementation
Source: BMC Genomics. 2010 Oct 11;11:553. doi: 10.1186/1471-2164-11-553 (PMC3091702; doi:10.1186/1471-2164-11-553)
Supplement: Additional file 2 — Interactive Direct Interaction Network representing the molecular interactions between zinc, copper, iron, calcium and proteins encoded by transcripts changed by zinc supplementation. Mini web-site containing index.html and hyperlinked pages in subdirectory describing a Direct Interaction Network automatically generated based on curated interactions contained within the proprietary PathwayArchitect database. Ovals represent proteins and the circles symbolize metal ions. Objects are coloured by their abundance in zebrafish at the time-point they were significantly different from the control is a scale from -4 fold (dark green) to +4 fold (dark red). Where significant differences were found at more than one time-point, the colour overlay shows expression at the first instance. Dark blue squares denote 'binding', and light blue squares 'expression'; green squares stand for 'regulation', green diamonds for 'metabolism', and green circles for 'promoter binding'. Arrow heads indicate directionality of the interaction where annotated. All nodes and edges can be further interrogated by selecting the relative area of the image. [file 1471-2164-11-553-S2.zip › PathwayArchitect Zn xs DIN/119161.html]

# PROTEIN: TNNT2

|  |  |
| --- | --- |
| Name | TNNT2 |
| Type | PROTEIN |
| Description | troponin T2, cardiac |
| Note | The protein encoded by this gene is the tropomyosin-binding subunit of the troponin complex, which is located on the thin filament of striated muscles and regulates muscle contraction in response to alterations in intracellular calcium ion concentration. Mutations in this gene have been associated with familial hypertrophic cardiomyopathy as well as with dilated cardiomyopathy. Transcripts for this gene undergo alternative splicing that results in many tissue-specific isoforms, however, the full length nature of some of these variants has not yet been determined. |
| Alias | troponin T |
|  | cTnT |
|  | troponin T, cardiac muscle |
|  | CTTG |
|  | CMD1D |
|  | TnTc |
|  | CMH2 |
|  | TNNT2 |
|  | Ctt |
|  | Troponin T cardiac |
|  | cardiac TnT |
|  | Tnnt3 |
|  | troponin-T2, cardiac |
|  | cardiac troponin T |
|  | Cardiac muscle troponin T |
|  | TnTC |
|  | RATCTTG |
|  | Tnt |
|  | MGC3889 |
|  | Tnnt2 |


---

|  |  |
| --- | --- |
| GO Component | troponin complex |
|  | sarcomere |
|  | cytoplasm |


---

|  |  |
| --- | --- |
| GO ID | GO:0006936 |
|  | GO:0005200 |
|  | GO:0007517 |
|  | GO:0005737 |
|  | GO:0030017 |
|  | GO:0006937 |
|  | GO:0008016 |
|  | GO:0005861 |


---

|  |  |
| --- | --- |
| MIM | MIM:191045 |
|  | MIM:115195 |
|  | MIM:601494 |


---

|  |  |
| --- | --- |
| Connectivity | 203 |


---

|  |  |
| --- | --- |
| Entrez ID | 7139 |
|  | 24837 |
|  | 21956 |


---

|  |  |
| --- | --- |
| Agilent ID | A\_44\_P994686 |
|  | A\_53\_P146062 |
|  | A\_14\_P135734 |
|  | A\_53\_P150185 |
|  | A\_24\_P927304 |
|  | A\_23\_P34700 |
|  | A\_51\_P338262 |
|  | A\_24\_P257022 |
|  | A\_14\_P129845 |
|  | A\_53\_P111826 |
|  | A\_52\_P430110 |
|  | A\_42\_P542380 |


---

|  |  |
| --- | --- |
| Cellular Localization | Cytoplasm |
|  | Cytoskeleton |
|  | Cell |
|  | Organelle |


---

|  |  |
| --- | --- |
| Pathway | Zn def RIN |
|  | Zn xs inventory |
|  | Zn xs DIN |


---

|  |  |
| --- | --- |
| GO Process | regulation of heart contraction rate |
|  | muscle contraction |
|  | regulation of muscle contraction |
|  | muscle development |


---

|  |  |
| --- | --- |
| UniGene | Hs.533613 |
|  | Rn.9965 |
|  | Mm.247470 |


---

|  |  |
| --- | --- |
| Affymetrix Probeset ID | 100593\_at |
|  | 1367592\_at |
|  | 1390061\_at |
|  | 1418726\_a\_at |
|  | 1424967\_x\_at |
|  | 1440424\_at |
|  | 1563655\_3p\_at |
|  | 1563655\_at |
|  | 171189\_r\_at |
|  | 215389\_s\_at |
|  | 38793\_at |
|  | AFFX-hum\_alu\_at |
|  | Hs2.407132.1.S1\_3p\_at |
|  | Hs2.407132.1.S1\_3p\_x\_at |
|  | Hs.296865.1.S1\_3p\_a\_at |
|  | hum\_alu\_at |
|  | l47599\_s\_at |
|  | M80829\_at |
|  | X74819\_at |
|  | rc\_AA924146\_at |


---

|  |  |
| --- | --- |
| GO Function | structural constituent of cytoskeleton |


---

|  |  |
| --- | --- |
| Nucleotide | Y09628 |
|  | NM\_012676 |
|  | S71128 |
|  | X79859 |
|  | L47599 |
|  | L47600 |
|  | AY160216 |
|  | AB052890 |
|  | AL832707 |
|  | L47570 |
|  | X83743 |
|  | AK164592 |
|  | AK146945 |
|  | X74819 |
|  | NM\_001001432 |
|  | L46872 |
|  | L47553 |
|  | Y09627 |
|  | L47549 |
|  | S64668 |
|  | L47550 |
|  | AY277394 |
|  | AK125236 |
|  | Y09626 |
|  | L47551 |
|  | AK168393 |
|  | BC063753 |
|  | L47552 |
|  | AY044273 |
|  | NM\_001001430 |
|  | NM\_011619 |
|  | X79856 |
|  | AK163786 |
|  | M26051 |
|  | AF004415 |
|  | M26052 |
|  | X79858 |
|  | M80829 |
|  | AK169061 |
|  | X79855 |
|  | L40162 |
|  | NM\_000364 |
|  | AK055533 |
|  | S71126 |
|  | BC002653 |
|  | NM\_001001431 |
|  | X79861 |
|  | AF004422 |


---

|  |  |
| --- | --- |
| Protein | CAA70840 |
|  | AAA85347 |
|  | AAA85351 |
|  | AAB07676 |
|  | BAE37494 |
|  | NP\_001001432 |
|  | AAB30957 |
|  | AAC39590 |
|  | P50753 |
|  | AAA85346 |
|  | AAH63753 |
|  | AAK92231 |
|  | BAB19881 |
|  | NP\_001001431 |
|  | AAA67422 |
|  | AAP96757 |
|  | CAA52818 |
|  | CAA70841 |
|  | BAC86093 |
|  | CAA56240 |
|  | AAA42297 |
|  | AAA85350 |
|  | AAB27731 |
|  | CAA56239 |
|  | AAB30956 |
|  | NP\_000355 |
|  | AAA42296 |
|  | AAA85348 |
|  | AAA85345 |
|  | AAN71651 |
|  | NP\_035749 |
|  | P50752 |
|  | NP\_036808 |
|  | AAH02653 |
|  | CAA56235 |
|  | AAA85349 |
|  | CAA56238 |
|  | BAE27554 |
|  | BAE40318 |
|  | P45379 |
|  | CAA70839 |
|  | CAA56236 |
|  | AAA85352 |
|  | NP\_001001430 |


---

|  |  |
| --- | --- |
| Organism | Mammal |


---

|  |  |
| --- | --- |
| Location | chromosome 1, 1 60.0 cM, 1 E4 (Mus musculus) |
|  | 1 60.0 cM (Mus musculus) |
|  | chromosome 13, 13q13 (Rattus norvegicus) |
|  | chromosome 1, 1q32 (Homo sapiens) |


---

|  |  |
| --- | --- |
