# Supplementary material for: Dynamic transcriptomic profiles of zebrafish gills in response to zinc supplementation
Source: BMC Genomics. 2010 Oct 11;11:553. doi: 10.1186/1471-2164-11-553 (PMC3091702; doi:10.1186/1471-2164-11-553)
Supplement: Additional file 2 — Interactive Direct Interaction Network representing the molecular interactions between zinc, copper, iron, calcium and proteins encoded by transcripts changed by zinc supplementation. Mini web-site containing index.html and hyperlinked pages in subdirectory describing a Direct Interaction Network automatically generated based on curated interactions contained within the proprietary PathwayArchitect database. Ovals represent proteins and the circles symbolize metal ions. Objects are coloured by their abundance in zebrafish at the time-point they were significantly different from the control is a scale from -4 fold (dark green) to +4 fold (dark red). Where significant differences were found at more than one time-point, the colour overlay shows expression at the first instance. Dark blue squares denote 'binding', and light blue squares 'expression'; green squares stand for 'regulation', green diamonds for 'metabolism', and green circles for 'promoter binding'. Arrow heads indicate directionality of the interaction where annotated. All nodes and edges can be further interrogated by selecting the relative area of the image. [file 1471-2164-11-553-S2.zip › PathwayArchitect Zn xs DIN/121001.html]

# PROTEIN: SLC3A2

|  |  |
| --- | --- |
| Name | SLC3A2 |
| Type | PROTEIN |
| Description | solute carrier family 3 (activators of dibasic and neutral amino acid transport), member 2 |
| Note | This gene is a member of the solute carrier family and encodes a cell surface, transmembrane protein with an alpha amylase domain. The protein exists as the heavy chain of a heterodimer, covalently bound through di-sulfide bonds to one of several possible light chains. It associates with integrins and mediates integrin-dependent signaling related to normal cell growth and tumorigenesis. Alternate transcriptional splice variants, encoding different isoforms, have been characterized. |
| Alias | solute carrier family 3, member 2 |
|  | antigen defined by monoclonal antibody 4F2, heavy chain |
|  | CD98 heavy chain |
|  | antigen identified by monoclonal antibodies 4F2, TRA1.10, TROP4, and T43 |
|  | CD98 antigen |
|  | 4F2hc |
|  | SLC3A2 |
|  | 4F2 cell-surface antigen heavy chain |
|  | Lymphocyte activation antigen 4F2 large subunit |
|  | lymphocyte activation antigen 4F2 large subunit |
|  | 4F2HC |
|  | Mdu1 |
|  | Ly-m10 |
|  | 4F2 |
|  | CD98 |
|  | CD98HC |
|  | monoclonal antibody 44D7 |
|  | 4F2 heavy chain |
|  | 4T2HC |
|  | 4F2 heavy chain antigen |
|  | MDU1 |
|  | NACAE |
|  | AI314110 |
|  | Slc3a2 |
|  | antigen identified by monoclonal antibodies 4F2 |
|  | solute carrier family 3 member 2 |
|  | Ly10 |
|  | type II transmembrane protein |
|  | Ly-10 |
|  | Mgp-2hc |
|  | Cd98 |


---

|  |  |
| --- | --- |
| GO Component | integral to membrane |
|  | plasma membrane |
|  | cell surface |


---

|  |  |
| --- | --- |
| GO ID | GO:0005432 |
|  | GO:0015175 |
|  | GO:0004556 |
|  | GO:0006816 |
|  | GO:0016049 |
|  | GO:0009986 |
|  | GO:0006865 |
|  | GO:0005515 |
|  | GO:0006952 |
|  | GO:0015804 |
|  | GO:0016021 |
|  | GO:0005975 |
|  | GO:0005886 |


---

|  |  |
| --- | --- |
| MIM | MIM:158070 |


---

|  |  |
| --- | --- |
| Connectivity | 160 |


---

|  |  |
| --- | --- |
| Entrez ID | 6520 |
|  | 50567 |
|  | 17254 |


---

|  |  |
| --- | --- |
| Agilent ID | A\_53\_P157063 |
|  | A\_14\_P124364 |
|  | A\_43\_P12007 |
|  | A\_14\_P130429 |
|  | A\_51\_P472901 |
|  | A\_23\_P87216 |
|  | A\_52\_P245648 |
|  | A\_23\_P75808 |
|  | A\_23\_P75811 |


---

|  |  |
| --- | --- |
| Cellular Localization | Plasma membrane |
|  | Cell surface |
|  | Membrane |
|  | Cell |


---

|  |  |
| --- | --- |
| Pathway | Zn xs inventory |
|  | Zn xs DIN |


---

|  |  |
| --- | --- |
| GO Process | neutral amino acid transport |
|  | amino acid transport |
|  | carbohydrate metabolism |
|  | calcium ion transport |
|  | cell growth |
|  | defense response |


---

|  |  |
| --- | --- |
| UniGene | Hs.502769 |
|  | Rn.5801 |
|  | Mm.4114 |


---

|  |  |
| --- | --- |
| Affymetrix Probeset ID | 129627\_at |
|  | 1398771\_at |
|  | 1425364\_a\_at |
|  | 200924\_3p\_s\_at |
|  | 200924\_s\_at |
|  | 38029\_at |
|  | 73232\_s\_at |
|  | 73338\_s\_at |
|  | 80495\_s\_at |
|  | 99133\_at |
|  | AB015433\_s\_at |
|  | g4505140\_3p\_a\_at |
|  | M21904\_at |
|  | Msa.14568.0\_f\_at |
|  | Msa.19973.0\_f\_at |
|  | Msa.2134.0\_f\_at |
|  | Msa.26416.0\_f\_at |
|  | x14309\_f\_at |
|  | X89225cds\_s\_at |
|  | Msa.8856.0\_s\_at |
|  | TC33706\_f\_at |
|  | TC38777\_f\_at |
|  | c79507\_rc\_f\_at |


---

|  |  |
| --- | --- |
| GO Function | protein binding |
|  | calcium:sodium antiporter activity |
|  | neutral amino acid transporter activity |
|  | alpha-amylase activity |


---

|  |  |
| --- | --- |
| Nucleotide | J02769 |
|  | AK206213 |
|  | AK218144 |
|  | AK180963 |
|  | AK156285 |
|  | AK025584 |
|  | BC003000 |
|  | CR607545 |
|  | CR604593 |
|  | AK165417 |
|  | AK184227 |
|  | AK090046 |
|  | CR611645 |
|  | AK210586 |
|  | CR615144 |
|  | NM\_019283 |
|  | AK215848 |
|  | BC065173 |
|  | NM\_008577 |
|  | CR623482 |
|  | AK094620 |
|  | BC061989 |
|  | NM\_001012664 |
|  | AK165550 |
|  | AB023408 |
|  | CR596156 |
|  | CR614278 |
|  | X14309 |
|  | AK212448 |
|  | CR604303 |
|  | AK090758 |
|  | NM\_001012662 |
|  | NM\_001012663 |
|  | NM\_001013251 |
|  | CR603627 |
|  | AB018010 |
|  | CR625807 |
|  | NM\_001012661 |
|  | CR599471 |
|  | CR608039 |
|  | AB015433 |
|  | AF055031 |
|  | CR605685 |
|  | AK215373 |
|  | AK189041 |
|  | CR606258 |
|  | AK198642 |
|  | AK167773 |
|  | AK161280 |
|  | J02939 |
|  | M21904 |
|  | CR591236 |
|  | NM\_002394 |
|  | U59324 |
|  | CR594426 |
|  | CR599523 |
|  | AK186746 |
|  | U25708 |
|  | AK197569 |
|  | BC001061 |
|  | AK183775 |
|  | CR625870 |
|  | CR623964 |
|  | J03569 |
|  | AK186309 |


---

|  |  |
| --- | --- |
| Protein | BAC41063 |
|  | BAA90555 |
|  | BAA84649 |
|  | NP\_001012680 |
|  | NP\_002385 |
|  | BAE36291 |
|  | BAE33656 |
|  | AAH01061 |
|  | CAA32490 |
|  | AAA35489 |
|  | P10852 |
|  | AAH61989 |
|  | AAH65173 |
|  | NP\_001012679 |
|  | BAE39805 |
|  | P08195 |
|  | AAA52497 |
|  | AAH03000 |
|  | NP\_062156 |
|  | NP\_001012681 |
|  | AAC53560 |
|  | AAA51540 |
|  | BAE38250 |
|  | NP\_001012682 |
|  | NP\_001013269 |
|  | AAA35536 |
|  | BAE38172 |
|  | BAA33036 |
|  | NP\_032603 |
|  | AAB03769 |


---

|  |  |
| --- | --- |
| Organism | Mammal |


---

|  |  |
| --- | --- |
| Location | chromosome 11, 11q13 (Homo sapiens) |
|  | chromosome 19, 19 0.0 cM, 19 A (Mus musculus) |
|  | chromosome 1, 1q43 (Rattus norvegicus) |
|  | 19 0.0 cM (Mus musculus) |


---

|  |  |
| --- | --- |
