# Supplementary material for: Dynamic transcriptomic profiles of zebrafish gills in response to zinc supplementation
Source: BMC Genomics. 2010 Oct 11;11:553. doi: 10.1186/1471-2164-11-553 (PMC3091702; doi:10.1186/1471-2164-11-553)
Supplement: Additional file 2 — Interactive Direct Interaction Network representing the molecular interactions between zinc, copper, iron, calcium and proteins encoded by transcripts changed by zinc supplementation. Mini web-site containing index.html and hyperlinked pages in subdirectory describing a Direct Interaction Network automatically generated based on curated interactions contained within the proprietary PathwayArchitect database. Ovals represent proteins and the circles symbolize metal ions. Objects are coloured by their abundance in zebrafish at the time-point they were significantly different from the control is a scale from -4 fold (dark green) to +4 fold (dark red). Where significant differences were found at more than one time-point, the colour overlay shows expression at the first instance. Dark blue squares denote 'binding', and light blue squares 'expression'; green squares stand for 'regulation', green diamonds for 'metabolism', and green circles for 'promoter binding'. Arrow heads indicate directionality of the interaction where annotated. All nodes and edges can be further interrogated by selecting the relative area of the image. [file 1471-2164-11-553-S2.zip › PathwayArchitect Zn xs DIN/1213890.html]

# REGULATION:

|  |  |
| --- | --- |
| Type | REGULATION |
| Effect | Positive |


---

|  |  |
| --- | --- |
| Score | 0 |


---

|  |  |
| --- | --- |
| Reference Count | 5 |


---

|  |  |
| --- | --- |
| Mechanism | Unknown |


---

|  |  |
| --- | --- |
| Reference:0 || Sentence | "Establishment of stable tranfectants expressing the membrane-type MMPs, MT-1 MMP and MT-2 MMP did restore fusion in the MMP-poor cell line TE671 after transfection with GALV M40, thus indicating that both membrane-type MMPs and soluble MMPs activate the MMP cleavable constructs." |
| PMID | 12704411 |
| Year | 2003 |
| Species | Mouse |
|  | Human |
| Journal | Gene Ther |
| RefScore | 1 |
| Source | PArchNLP |
  |
|


---

|  |  |
| --- | --- |
 Reference:1 || Sentence | "MMP-like enzymes of 62 kDa, probably activated MMP-2, were upregulated in the chronic phase, whereas components of 92, 85, or 67 kDa were highly induced in the acute phase." |
| PMID | 10594729 |
| Year | 1999 |
| Species | Mouse |
| Journal | J Invest Dermatol |
| RefScore | 1 |
| Source | PArchNLP |
  ||


---

|  |  |
| --- | --- |
 Reference:2 || Sentence | "METHODS: Plasma levels for the gelatinases MMP-2 and MMP-9, and for the collagenases MMP-8 and MMP-13, as well as TIMP-1 profiles were examined (by enzyme-linked immunosorbent assay) at baseline and serially up to 60 h following alcohol injection into the septal perforator artery in order to induce an MI in 51 patients with HOCM (age 55 +/- 2 years)." |
| PMID | 12505230 |
| Year | 2002 |
| Species | Human |
| Journal | J Am Coll Cardiol |
| RefScore | 2 |
| Source | PArchNLP |
  ||


---

|  |  |
| --- | --- |
 Reference:3 || Sentence | "Collagenase mRNA is synthesized when the latent extracellular pool of MMP-1 is reduced through the activation of latent collagenases and gelatinases." |
| PMID | 8531210 |
| Year | 1995 |
| Species | Rat |
| Journal | J Mol Cell Cardiol |
| RefScore | 2 |
| Source | PArchNLP |
  ||


---

|  |  |
| --- | --- |
 Reference:4 || Sentence | "The recombinant mMT4-MMP catalytic domain was also unable to activate pro-MMP2 and was very poor at hydrolyzing components of the extracellular matrix with the exception of fibrinogen and fibrin." |
| PMID | 10799478 |
| Year | 2000 |
| Species | Human |
|  | Mouse |
| Journal | J Biol Chem |
| RefScore | 1 |
| Source | PArchNLP |
  |


---

|  |  |
| --- | --- |
