# Supplementary material for: Dynamic transcriptomic profiles of zebrafish gills in response to zinc supplementation
Source: BMC Genomics. 2010 Oct 11;11:553. doi: 10.1186/1471-2164-11-553 (PMC3091702; doi:10.1186/1471-2164-11-553)
Supplement: Additional file 2 — Interactive Direct Interaction Network representing the molecular interactions between zinc, copper, iron, calcium and proteins encoded by transcripts changed by zinc supplementation. Mini web-site containing index.html and hyperlinked pages in subdirectory describing a Direct Interaction Network automatically generated based on curated interactions contained within the proprietary PathwayArchitect database. Ovals represent proteins and the circles symbolize metal ions. Objects are coloured by their abundance in zebrafish at the time-point they were significantly different from the control is a scale from -4 fold (dark green) to +4 fold (dark red). Where significant differences were found at more than one time-point, the colour overlay shows expression at the first instance. Dark blue squares denote 'binding', and light blue squares 'expression'; green squares stand for 'regulation', green diamonds for 'metabolism', and green circles for 'promoter binding'. Arrow heads indicate directionality of the interaction where annotated. All nodes and edges can be further interrogated by selecting the relative area of the image. [file 1471-2164-11-553-S2.zip › PathwayArchitect Zn xs DIN/122328.html]

# PROTEIN: DDEF2

|  |  |
| --- | --- |
| Name | DDEF2 |
| Type | PROTEIN |
| Description | development and differentiation enhancing factor 2 |
| Note | This gene encodes a multidomain protein containing an N-terminal alpha-helical region with a coiled-coil motif, followed by a pleckstrin homology (PH) domain, an Arf-GAP domain, an ankyrin homology region, a proline-rich region, and a C-terminal Src homology 3 (SH3) domain. The protein localizes in the Golgi apparatus and at the plasma membrane, where it colocalizes with protein tyrosine kinase 2-beta (PYK2). The encoded protein forms a stable complex with PYK2 in vivo. This interaction appears to be mediated by binding of its SH3 domain to the C-terminal proline-rich domain of PYK2. The encoded protein is tyrosine phosphorylated by activated PYK2. In vitro it shows strong GTPase-activating protein (GAP) activity towards the small GTPases ADP-ribosylation factor (ARF) 1 and ARF5 and weak activity towards ARF6. The encoded protein is believed to function as an ARF GAP that controls ARF-mediated vesicle budding when recruited to Golgi membranes. In addition, it functions as a substrate and downstream target for PYK2 and SRC, a pathway that may be involved in the regulation of vesicular transport. |
| Alias | PAG3 |
|  | PYK2 C terminus-associated protein |
|  | Pap-alpha |
|  | development- and differentiation-enhancing factor 2 |
|  | Gm1523 |
|  | SHAG1 |
|  | PAP |
|  | MGC90837 |
|  | KIAA0400 |
|  | Gm592 |
|  | AMAP2 |


---

|  |  |
| --- | --- |
| GO Component | Golgi stack |


---

|  |  |
| --- | --- |
| GO ID | GO:0005795 |
|  | GO:0046872 |
|  | GO:0043087 |
|  | GO:0005096 |


---

|  |  |
| --- | --- |
| MIM | MIM:603817 |


---

|  |  |
| --- | --- |
| Connectivity | 21 |


---

|  |  |
| --- | --- |
| Entrez ID | 8853 |
|  | 211914 |


---

|  |  |
| --- | --- |
| Agilent ID | A\_23\_P40088 |
|  | A\_51\_P427080 |
|  | A\_23\_P40090 |
|  | A\_14\_P124632 |
|  | A\_24\_P362540 |
|  | A\_52\_P536731 |
|  | A\_14\_P112224 |
|  | A\_14\_P110854 |


---

|  |  |
| --- | --- |
| Cellular Localization | Golgi apparatus |
|  | Cytoplasm |
|  | Organelle |
|  | Cell |


---

|  |  |
| --- | --- |
| Pathway | Zn xs inventory |
|  | Zn xs DIN |


---

|  |  |
| --- | --- |
| GO Process | regulation of GTPase activity |


---

|  |  |
| --- | --- |
| UniGene | Hs.555902 |
|  | Mm.379332 |


---

|  |  |
| --- | --- |
| Affymetrix Probeset ID | 112496\_at |
|  | 1431369\_at |
|  | 1436181\_at |
|  | 1445697\_at |
|  | 206414\_s\_at |
|  | 140935\_r\_at |
|  | 140934\_i\_at |
|  | 39410\_at |
|  | 69698\_at |
|  | g4502248\_3p\_s\_at |
|  | Hs.200850.0.A1\_3p\_at |
|  | 232476\_at |
|  | 244319\_at |
|  | 70082\_at |
|  | RC\_AA412251\_at |
|  | TC42045\_at |


---

|  |  |
| --- | --- |
| GO Function | GTPase activator activity |
|  | metal ion binding |


---

|  |  |
| --- | --- |
| Nucleotide | AC093904 |
|  | AB007860 |
|  | BC038615 |
|  | AK172944 |
|  | BC063308 |
|  | AK147656 |
|  | AK078798 |
|  | NM\_001004364 |
|  | BC080847 |
|  | AK162242 |
|  | NM\_003887 |
|  | AC080162 |
|  | AC079782 |
|  | AK091121 |
|  | BC096022 |


---

|  |  |
| --- | --- |
| Protein | BAC03588 |
|  | Q7SIG6 |
|  | AAY24112 |
|  | BAA23696 |
|  | AAY14904 |
|  | AAY14856 |
|  | BAD32222 |
|  | AAH80847 |
|  | NP\_001004364 |
|  | NP\_003878 |
|  | AAH63308 |
|  | AAH96022 |
|  | BAE28052 |
|  | O43150 |
|  | BAE36812 |


---

|  |  |
| --- | --- |
| Organism | Mammal |


---

|  |  |
| --- | --- |
| Location | chromosome 12 (Mus musculus) |
|  | 2p24 (Homo sapiens) |
|  | chromosome 2, 2p25, 2p25 (Homo sapiens) |


---

|  |  |
| --- | --- |
