# Supplementary material for: Dynamic transcriptomic profiles of zebrafish gills in response to zinc supplementation
Source: BMC Genomics. 2010 Oct 11;11:553. doi: 10.1186/1471-2164-11-553 (PMC3091702; doi:10.1186/1471-2164-11-553)
Supplement: Additional file 2 — Interactive Direct Interaction Network representing the molecular interactions between zinc, copper, iron, calcium and proteins encoded by transcripts changed by zinc supplementation. Mini web-site containing index.html and hyperlinked pages in subdirectory describing a Direct Interaction Network automatically generated based on curated interactions contained within the proprietary PathwayArchitect database. Ovals represent proteins and the circles symbolize metal ions. Objects are coloured by their abundance in zebrafish at the time-point they were significantly different from the control is a scale from -4 fold (dark green) to +4 fold (dark red). Where significant differences were found at more than one time-point, the colour overlay shows expression at the first instance. Dark blue squares denote 'binding', and light blue squares 'expression'; green squares stand for 'regulation', green diamonds for 'metabolism', and green circles for 'promoter binding'. Arrow heads indicate directionality of the interaction where annotated. All nodes and edges can be further interrogated by selecting the relative area of the image. [file 1471-2164-11-553-S2.zip › PathwayArchitect Zn xs DIN/122864.html]

# PROTEIN: CYP1A1

|  |  |
| --- | --- |
| Name | CYP1A1 |
| Type | PROTEIN |
| Description | cytochrome P450, family 1, subfamily A, polypeptide 1 |
| Note | This gene, CYP1A1, encodes a member of the cytochrome P450 superfamily of enzymes. The cytochrome P450 proteins are monooxygenases which catalyze many reactions involved in drug metabolism and synthesis of cholesterol, steroids and other lipids. This protein localizes to the endoplasmic reticulum and its expression is induced by some polycyclic aromatic hydrocarbons (PAHs), some of which are found in cigarette smoke. The enzyme's endogenous substrate is unknown; however, it is able to metabolize some PAHs to carcinogenic intermediates. The gene has been associated with lung cancer risk. A related family member, CYP1A2, is located approximately 25 kb away from CYP1A1 on chromosome 15. |
| Alias | P450form6 |
|  | P450 form 6 |
|  | cytochrome P450, 1a1, aromatic compound inducible |
|  | Cyp1a1 |
|  | Cypc45c |
|  | Cytochrome P450 subfamily I (aromatic compound-inducible) member A1 (C6 form c) |
|  | cytochrome P450, subfamily I (aromatic compound-inducible), member A1 (C6, form c) (C6, form c) |
|  | aryl hydrocarbon hydroxylase |
|  | Cyp45c |
|  | P-450MC |
|  | cytochrome P450 subfamily I, polypeptide 1 |
|  | P450DX |
|  | P450-P1 |
|  | AHRR |
|  | cytochrome P450 subfamily I (aromatic compound-inducible) member A1 (C6 form c) (C6 form c) |
|  | cytochrome P450, subfamily I (aromatic compound-inducible), polypeptide 1 |
|  | CYP1A1 |
|  | CP11 |
|  | Cyp1a-1 |
|  | cytochrome P450, 1a1 |
|  | cytochrome P1-450, dioxin-inducible |
|  | aromatic compound inducible |
|  | P1-450 |
|  | flavoprotein-linked monooxygenase |
|  | CYPIA1 |
|  | cytochrome P450 1a1 |
|  | cytochrome P1-450 |
|  | microsomal monooxygenase |
|  | dioxin-inducible |
|  | P450MT2 |
|  | xenobiotic monooxygenase |
|  | P450-C |
|  | P450-1 |
|  | CYP1 |
|  | AHH |


---

|  |  |
| --- | --- |
| GO Component | mitochondrion |
|  | extracellular space |
|  | endoplasmic reticulum |
|  | microsome |
|  | membrane |


---

|  |  |
| --- | --- |
| GO ID | GO:0018894 |
|  | GO:0006810 |
|  | GO:0016020 |
|  | GO:0020037 |
|  | GO:0005783 |
|  | GO:0005792 |
|  | GO:0005615 |
|  | GO:0005506 |
|  | GO:0006118 |
|  | GO:0019825 |
|  | GO:0046872 |
|  | GO:0005739 |
|  | GO:0004497 |
|  | GO:0016712 |
|  | GO:0050381 |
|  | GO:0016491 |


---

|  |  |
| --- | --- |
| MIM | MIM:108330 |


---

|  |  |
| --- | --- |
| Connectivity | 1305 |


---

|  |  |
| --- | --- |
| Entrez ID | 13076 |
|  | 1543 |
|  | 24296 |


---

|  |  |
| --- | --- |
| Agilent ID | A\_51\_P279693 |
|  | A\_23\_P163402 |
|  | A\_44\_P321009 |
|  | A\_14\_P106416 |
|  | A\_53\_P178769 |
|  | A\_43\_P11453 |


---

|  |  |
| --- | --- |
| Cellular Localization | Membrane |
|  | Endoplasmic reticulum |
|  | Mitochondrion |
|  | Cytoplasm |
|  | Extracellular region |
|  | Cell |
|  | Organelle |


---

|  |  |
| --- | --- |
| DbXref | Reactome##140192##Xenobiotic metabolism##http://www.reactome.org/cgi-bin/eventbrowser?DB=gk\_current&ID=140192 |
|  | KEGG pathway##00361##gamma-Hexachlorocyclohexane degradation##http://www.genome.jp/dbget-bin/show\_pathway?rno00361+24296 |
|  | KEGG pathway##00380##Tryptophan metabolism##http://www.genome.jp/dbget-bin/show\_pathway?mmu00380+13076 |
|  | Reactome##172464##Xenobiotic metabolism##http://www.reactome.org/cgi-bin/eventbrowser?DB=gk\_current&ID=172464 |
|  | KEGG pathway##00071##Fatty acid metabolism##http://www.genome.jp/dbget-bin/show\_pathway?hsa00071+1543 |
|  | KEGG pathway##00380##Tryptophan metabolism##http://www.genome.jp/dbget-bin/show\_pathway?hsa00380+1543 |
|  | KEGG pathway##00071##Fatty acid metabolism##http://www.genome.jp/dbget-bin/show\_pathway?rno00071+24296 |
|  | KEGG pathway##00380##Tryptophan metabolism##http://www.genome.jp/dbget-bin/show\_pathway?rno00380+24296 |
|  | KEGG pathway##00361##gamma-Hexachlorocyclohexane degradation##http://www.genome.jp/dbget-bin/show\_pathway?hsa00361+1543 |


---

|  |  |
| --- | --- |
| Pathway | AHR Signal Transduction |
|  | Zn xs inventory |
|  | Zn xs DIN |


---

|  |  |
| --- | --- |
| GO Process | transport |
|  | electron transport |
|  | dibenzo-p-dioxin metabolism |


---

|  |  |
| --- | --- |
| UniGene | Hs.72912 |
|  | Rn.10352 |
|  | Mm.14089 |


---

|  |  |
| --- | --- |
| Affymetrix Probeset ID | 1024\_at |
|  | 1025\_g\_at |
|  | 1370269\_at |
|  | 1422217\_a\_at |
|  | 161831\_at |
|  | 205749\_at |
|  | 36767\_at |
|  | 94715\_at |
|  | E00717UTR#1\_s\_at |
|  | E00778cds\_s\_at |
|  | g13325053\_3p\_at |
|  | x01681\_s\_at |
|  | X02612\_at |
|  | 98252\_r\_at |
|  | RC\_AA418907\_s\_at |


---

|  |  |
| --- | --- |
| EC Number | EC 1.14.14.1 |


---

|  |  |
| --- | --- |
| GO Function | oxidoreductase activity, acting on paired donors, with incorporation or reduction of molecular oxygen, reduced flavin or flavoprotein as one donor, and incorporation of one atom of oxygen |
|  | oxidoreductase activity |
|  | monooxygenase activity |
|  | iron ion binding |
|  | heme binding |
|  | oxygen binding |
|  | unspecific monooxygenase activity |
|  | metal ion binding |


---

|  |  |
| --- | --- |
| Nucleotide | M10021 |
|  | M26129 |
|  | M12079 |
|  | X01681 |
|  | NM\_012540 |
|  | M33935 |
|  | X02612 |
|  | AK005000 |
|  | NM\_009992 |
|  | Y00071 |
|  | D10855 |
|  | BC023019 |
|  | K02588 |
|  | NM\_000499 |
|  | M25623 |
|  | X00469 |
|  | X17160 |
|  | K03191 |
|  | AK223113 |
|  | X04300 |
|  | AK223108 |
|  | AF040259 |
|  | K02246 |
|  | AF253322 |


---

|  |  |
| --- | --- |
| Protein | BAB23734 |
|  | CAA27843 |
|  | AAA52139 |
|  | P04798 |
|  | AAH23019 |
|  | NP\_000490 |
|  | CAA25836 |
|  | NP\_036672 |
|  | AAD10199 |
|  | AAA52152 |
|  | AAA41027 |
|  | CAA68277 |
|  | AAA37506 |
|  | P00185 |
|  | BAD96833 |
|  | BAD96828 |
|  | CAA26458 |
|  | AAA37507 |
|  | CAA35039 |
|  | AAA39868 |
|  | AAK25727 |
|  | CAA25153 |
|  | NP\_034122 |
|  | P00184 |
|  | AAA41025 |


---

|  |  |
| --- | --- |
| Organism | Mammal |


---

|  |  |
| --- | --- |
| Location | chromosome 9, 9 31.0 cM, 9 B (Mus musculus) |
|  | 9 31.0 cM (Mus musculus) |
|  | chromosome 8, 8q24 (Rattus norvegicus) |
|  | chromosome 15, 15q22-q24 (Homo sapiens) |


---

|  |  |
| --- | --- |
