# Supplementary material for: Dynamic transcriptomic profiles of zebrafish gills in response to zinc supplementation
Source: BMC Genomics. 2010 Oct 11;11:553. doi: 10.1186/1471-2164-11-553 (PMC3091702; doi:10.1186/1471-2164-11-553)
Supplement: Additional file 2 — Interactive Direct Interaction Network representing the molecular interactions between zinc, copper, iron, calcium and proteins encoded by transcripts changed by zinc supplementation. Mini web-site containing index.html and hyperlinked pages in subdirectory describing a Direct Interaction Network automatically generated based on curated interactions contained within the proprietary PathwayArchitect database. Ovals represent proteins and the circles symbolize metal ions. Objects are coloured by their abundance in zebrafish at the time-point they were significantly different from the control is a scale from -4 fold (dark green) to +4 fold (dark red). Where significant differences were found at more than one time-point, the colour overlay shows expression at the first instance. Dark blue squares denote 'binding', and light blue squares 'expression'; green squares stand for 'regulation', green diamonds for 'metabolism', and green circles for 'promoter binding'. Arrow heads indicate directionality of the interaction where annotated. All nodes and edges can be further interrogated by selecting the relative area of the image. [file 1471-2164-11-553-S2.zip › PathwayArchitect Zn xs DIN/123927.html]

# PROTEIN: PLN

|  |  |
| --- | --- |
| Name | PLN |
| Type | PROTEIN |
| Description | phospholamban |
| Note | The protein encoded by this gene is found as a pentamer and is a major substrate for the cAMP-dependent protein kinase in cardiac muscle. The encoded protein is an inhibitor of cardiac muscle sarcoplasmic reticulum Ca(2+)-ATPase in the unphosphorylated state, but inhibition is relieved upon phosphorylation of the protein. The subsequent activation of the Ca(2+) pump leads to enhanced muscle relaxation rates, thereby contributing to the inotropic response elicited in heart by beta-agonists. The encoded protein is a key regulator of cardiac diastolic function. Mutations in this gene are a cause of inherited human dilated cardiomyopathy with refractory congestive heart failure. |
| Alias | cardiac phospholamban |
|  | Pln |
|  | PLB |
|  | Plm |


---

|  |  |
| --- | --- |
| GO Component | integral to membrane |
|  | membrane |
|  | smooth endoplasmic reticulum |


---

|  |  |
| --- | --- |
| GO ID | GO:0016020 |
|  | GO:0005790 |
|  | GO:0042030 |
|  | GO:0006936 |
|  | GO:0006816 |
|  | GO:0008015 |
|  | GO:0005246 |
|  | GO:0016021 |


---

|  |  |
| --- | --- |
| MIM | MIM:172405 |
|  | MIM:115200 |


---

|  |  |
| --- | --- |
| Connectivity | 383 |


---

|  |  |
| --- | --- |
| Entrez ID | 5350 |
|  | 18821 |
|  | 64672 |


---

|  |  |
| --- | --- |
| Agilent ID | A\_23\_P30614 |
|  | A\_53\_P122673 |
|  | A\_32\_P213418 |
|  | A\_51\_P149885 |
|  | A\_44\_P742449 |
|  | A\_52\_P22324 |
|  | A\_44\_P226941 |
|  | A\_43\_P12307 |
|  | A\_53\_P130302 |
|  | A\_24\_P414803 |


---

|  |  |
| --- | --- |
| Cellular Localization | Membrane |
|  | Endoplasmic reticulum |
|  | Cell |
|  | Cytoplasm |
|  | Organelle |


---

|  |  |
| --- | --- |
| DbXref | KEGG pathway##04020##Calcium signaling pathway##http://www.genome.jp/dbget-bin/show\_pathway?mmu04020+18821 |
|  | KEGG pathway##04020##Calcium signaling pathway##http://www.genome.jp/dbget-bin/show\_pathway?hsa04020+5350 |
|  | KEGG pathway##04020##Calcium signaling pathway##http://www.genome.jp/dbget-bin/show\_pathway?rno04020+64672 |


---

|  |  |
| --- | --- |
| Pathway | Zn xs inventory |
|  | Zn xs DIN |


---

|  |  |
| --- | --- |
| GO Process | calcium ion transport |
|  | muscle contraction |
|  | circulation |


---

|  |  |
| --- | --- |
| UniGene | Rn.9740 |
|  | Mm.34145 |
|  | Hs.170839 |


---

|  |  |
| --- | --- |
| Affymetrix Probeset ID | 108397\_r\_at |
|  | 111363\_at |
|  | 1384689\_at |
|  | 1388876\_at |
|  | 1423359\_at |
|  | 1450952\_at |
|  | 1460332\_at |
|  | 204938\_s\_at |
|  | 204939\_s\_at |
|  | 204940\_at |
|  | 228202\_at |
|  | 38734\_at |
|  | 87826\_s\_at |
|  | 89608\_r\_at |
|  | g190018\_3p\_a\_at |
|  | g4505886\_3p\_a\_at |
|  | Hs.85050.1.A1\_3p\_at |
|  | M63603\_at |
|  | rc\_AI231802\_at |
|  | 77958\_at |
|  | TC37324\_at |
|  | TC38499\_at |


---

|  |  |
| --- | --- |
| GO Function | ATPase inhibitor activity |
|  | calcium channel regulator activity |


---

|  |  |
| --- | --- |
| Nucleotide | AK142708 |
|  | AF177764 |
|  | X71068 |
|  | NM\_023129 |
|  | AA840570 |
|  | L03382 |
|  | BC005269 |
|  | AF214653 |
|  | BC061097 |
|  | NM\_022707 |
|  | S95853 |
|  | S46792 |
|  | AA422329 |
|  | AK040718 |
|  | Z99496 |
|  | AK052199 |
|  | AK002622 |
|  | NM\_002667 |
|  | S95849 |
|  | M60411 |
|  | M63603 |


---

|  |  |
| --- | --- |
| Protein | BAC30680 |
|  | AAB21903 |
|  | P61014 |
|  | AAN86727 |
|  | AAA60083 |
|  | CAA50394 |
|  | BAE25168 |
|  | AAA41849 |
|  | NP\_002658 |
|  | NP\_075618 |
|  | BAB22237 |
|  | AAH05269 |
|  | AAD55950 |
|  | P26678 |
|  | AAB23706 |
|  | NP\_073198 |
|  | AAH61097 |
|  | CAI21610 |
|  | BAC34880 |
|  | P61016 |
|  | AAA60109 |


---

|  |  |
| --- | --- |
| Organism | Mammal |


---

|  |  |
| --- | --- |
| Location | chromosome 6, 6q22.1 (Homo sapiens) |
|  | chromosome 20, 20q11 (Rattus norvegicus) |
|  | chromosome 10, 10 B3 (Mus musculus) |


---

|  |  |
| --- | --- |
