# Supplementary material for: Dynamic transcriptomic profiles of zebrafish gills in response to zinc supplementation
Source: BMC Genomics. 2010 Oct 11;11:553. doi: 10.1186/1471-2164-11-553 (PMC3091702; doi:10.1186/1471-2164-11-553)
Supplement: Additional file 2 — Interactive Direct Interaction Network representing the molecular interactions between zinc, copper, iron, calcium and proteins encoded by transcripts changed by zinc supplementation. Mini web-site containing index.html and hyperlinked pages in subdirectory describing a Direct Interaction Network automatically generated based on curated interactions contained within the proprietary PathwayArchitect database. Ovals represent proteins and the circles symbolize metal ions. Objects are coloured by their abundance in zebrafish at the time-point they were significantly different from the control is a scale from -4 fold (dark green) to +4 fold (dark red). Where significant differences were found at more than one time-point, the colour overlay shows expression at the first instance. Dark blue squares denote 'binding', and light blue squares 'expression'; green squares stand for 'regulation', green diamonds for 'metabolism', and green circles for 'promoter binding'. Arrow heads indicate directionality of the interaction where annotated. All nodes and edges can be further interrogated by selecting the relative area of the image. [file 1471-2164-11-553-S2.zip › PathwayArchitect Zn xs DIN/126303.html]

# PROTEIN: CTBP1

|  |  |
| --- | --- |
| Name | CTBP1 |
| Type | PROTEIN |
| Description | C-terminal binding protein 1 |
| Note | This gene encodes a protein that binds to the C-terminus of adenovirus E1A proteins. This phosphoprotein is a transcriptional repressor and may play a role during cellular proliferation. This protein and the product of a second closely related gene, CTBP2, can dimerize. Both proteins can also interact with a polycomb group protein complex which participates in regulation of gene expression during development. Alternative splicing of transcripts from this gene results in multiple transcript variants. |
| Alias | CtBP1 |
|  | CtBP3/BARS |
|  | 50 kDa BFA-dependent ADP-ribosylation substrate |
|  | CTBP |
|  | BARS-50 |
|  | CTBP1 |
|  | BARS |
|  | Ctbp3 |
|  | MGC104684 |
|  | Bars |
|  | D4S115h |
|  | C-terminal binding protein 3 |
|  | D5H4S115E |
|  | 50-kDaBFA-inducedADP-ribosylatedsubstrate |
|  | brefeldin A-ADP-riboslyated substrate |
|  | D5H4S115 |
|  | C-terminus binding protein 3/brefeldin A (BFA) adenosine diphosphate-ribosylated substrate |
|  | MGC93318 |
|  | Ctbp1 |
|  | 50-kDa BFA-induced ADP-ribosylated substrate |
|  | CtBP3 |


---

|  |  |
| --- | --- |
| GO Component | Golgi stack |
|  | Golgi apparatus |
|  | nucleus |


---

|  |  |
| --- | --- |
| GO ID | GO:0005634 |
|  | GO:0016616 |
|  | GO:0019079 |
|  | GO:0008152 |
|  | GO:0006468 |
|  | GO:0051287 |
|  | GO:0005795 |
|  | GO:0005515 |
|  | GO:0008022 |
|  | GO:0005794 |
|  | GO:0006564 |
|  | GO:0016491 |
|  | GO:0008285 |


---

|  |  |
| --- | --- |
| MIM | MIM:602618 |


---

|  |  |
| --- | --- |
| Connectivity | 131 |


---

|  |  |
| --- | --- |
| Entrez ID | 13016 |
|  | 1487 |
|  | 29382 |


---

|  |  |
| --- | --- |
| Agilent ID | A\_32\_P118655 |
|  | A\_14\_P117618 |
|  | A\_42\_P477073 |
|  | A\_14\_P139932 |
|  | A\_51\_P482019 |
|  | A\_24\_P208992 |
|  | A\_23\_P41286 |
|  | A\_23\_P41292 |
|  | A\_43\_P11974 |
|  | A\_53\_P105074 |
|  | A\_53\_P172847 |
|  | A\_53\_P137069 |
|  | A\_51\_P482023 |


---

|  |  |
| --- | --- |
| Cellular Localization | Nucleus |
|  | Golgi apparatus |
|  | Cytoplasm |
|  | Organelle |
|  | Cell |


---

|  |  |
| --- | --- |
| DbXref | KEGG pathway##04330##Notch signaling pathway##http://www.genome.jp/dbget-bin/show\_pathway?rno04330+29382 |
|  | KEGG pathway##04330##Notch signaling pathway##http://www.genome.jp/dbget-bin/show\_pathway?mmu04330+13016 |
|  | KEGG pathway##04330##Notch signaling pathway##http://www.genome.jp/dbget-bin/show\_pathway?hsa04330+1487 |
|  | KEGG pathway##04310##Wnt signaling pathway##http://www.genome.jp/dbget-bin/show\_pathway?rno04310+29382 |
|  | KEGG pathway##04310##Wnt signaling pathway##http://www.genome.jp/dbget-bin/show\_pathway?mmu04310+13016 |
|  | KEGG pathway##04310##Wnt signaling pathway##http://www.genome.jp/dbget-bin/show\_pathway?hsa04310+1487 |


---

|  |  |
| --- | --- |
| Pathway | Zn xs inventory |
|  | Zn xs DIN |


---

|  |  |
| --- | --- |
| GO Process | negative regulation of cell proliferation |
|  | metabolism |
|  | protein amino acid phosphorylation |
|  | L-serine biosynthesis |
|  | viral genome replication |


---

|  |  |
| --- | --- |
| UniGene | Mm.7286 |
|  | Rn.3946 |
|  | Hs.208597 |


---

|  |  |
| --- | --- |
| Affymetrix Probeset ID | 101081\_at |
|  | 1370029\_at |
|  | 1415702\_a\_at |
|  | 1557714\_at |
|  | 203392\_s\_at |
|  | 212863\_x\_at |
|  | 213980\_s\_at |
|  | 243180\_at |
|  | 41308\_at |
|  | 41309\_g\_at |
|  | 43382\_s\_at |
|  | 55310\_at |
|  | 61328\_at |
|  | 61329\_g\_at |
|  | 70136\_r\_at |
|  | aa271294\_s\_at |
|  | AF067795\_at |
|  | AF067795\_g\_at |
|  | g4557496\_3p\_a\_at |
|  | Hs.239737.2.A1\_3p\_a\_at |
|  | Hs.239737.2.A1\_3p\_x\_at |
|  | Hs2.431402.1.S1\_3p\_at |
|  | Hs.252854.0.A1\_3p\_at |
|  | U37408\_at |
|  | 213979\_s\_at |
|  | Hs.239737.1.A1\_3p\_at |
|  | RC\_AA258801\_i\_at |
|  | RC\_AA258801\_s\_at |
|  | RC\_R87296\_at |
|  | TC29839\_at |


---

|  |  |
| --- | --- |
| EC Number | EC 1.1.1.- |


---

|  |  |
| --- | --- |
| GO Function | protein binding |
|  | oxidoreductase activity |
|  | NAD binding |
|  | protein C-terminus binding |
|  | oxidoreductase activity, acting on the CH-OH group of donors, NAD or NADP as acceptor |


---

|  |  |
| --- | --- |
| Nucleotide | AF067795 |
|  | AJ010483 |
|  | AK195486 |
|  | NM\_013502 |
|  | AK179724 |
|  | BC013702 |
|  | CR605200 |
|  | AK189146 |
|  | BC072021 |
|  | AK219771 |
|  | AC092535 |
|  | AK210217 |
|  | AK182443 |
|  | AK206815 |
|  | AK160658 |
|  | AK190732 |
|  | AK133816 |
|  | AK178530 |
|  | NM\_001012614 |
|  | AF091555 |
|  | U37408 |
|  | BC078778 |
|  | NM\_019201 |
|  | BC098400 |
|  | AK218288 |
|  | AK207288 |
|  | AK165276 |
|  | BC064333 |
|  | AK183752 |
|  | AK214870 |
|  | AK188829 |
|  | BC011655 |
|  | AK171650 |
|  | AK217104 |
|  | AK149906 |
|  | BC053320 |
|  | AK213613 |
|  | AB033122 |
|  | AK170133 |
|  | BC015071 |
|  | BC042425 |
|  | NM\_001328 |
|  | AL137653 |


---

|  |  |
| --- | --- |
| Protein | Q13363 |
|  | AAH15071 |
|  | Q9Z2F5 |
|  | AAH42425 |
|  | AAC62822 |
|  | AAH98400 |
|  | AAH11655 |
|  | AAH13702 |
|  | BAE42587 |
|  | O88712 |
|  | BAE21859 |
|  | CAB70861 |
|  | AAY40989 |
|  | BAA85180 |
|  | BAE41586 |
|  | CAA09219 |
|  | NP\_062074 |
|  | AAH53320 |
|  | AAC79427 |
|  | BAE38115 |
|  | NP\_038530 |
|  | NP\_001012632 |
|  | BAE35946 |
|  | AAH78778 |
|  | AAD14597 |
|  | AAH72021 |
|  | NP\_001319 |


---

|  |  |
| --- | --- |
| Organism | Mammal |


---

|  |  |
| --- | --- |
| Location | chromosome 4, 4p16 (Homo sapiens) |
|  | chromosome 14, 14q21 (Rattus norvegicus) |
|  | 5 19.0 cM (Mus musculus) |
|  | chromosome 5, 5 19.0 cM, 5 B1 (Mus musculus) |


---

|  |  |
| --- | --- |
