# Supplementary material for: Dynamic transcriptomic profiles of zebrafish gills in response to zinc supplementation
Source: BMC Genomics. 2010 Oct 11;11:553. doi: 10.1186/1471-2164-11-553 (PMC3091702; doi:10.1186/1471-2164-11-553)
Supplement: Additional file 2 — Interactive Direct Interaction Network representing the molecular interactions between zinc, copper, iron, calcium and proteins encoded by transcripts changed by zinc supplementation. Mini web-site containing index.html and hyperlinked pages in subdirectory describing a Direct Interaction Network automatically generated based on curated interactions contained within the proprietary PathwayArchitect database. Ovals represent proteins and the circles symbolize metal ions. Objects are coloured by their abundance in zebrafish at the time-point they were significantly different from the control is a scale from -4 fold (dark green) to +4 fold (dark red). Where significant differences were found at more than one time-point, the colour overlay shows expression at the first instance. Dark blue squares denote 'binding', and light blue squares 'expression'; green squares stand for 'regulation', green diamonds for 'metabolism', and green circles for 'promoter binding'. Arrow heads indicate directionality of the interaction where annotated. All nodes and edges can be further interrogated by selecting the relative area of the image. [file 1471-2164-11-553-S2.zip › PathwayArchitect Zn xs DIN/1268336.html]

# EXPRESSION:

|  |  |
| --- | --- |
| Type | EXPRESSION |
| Effect | None |


---

|  |  |
| --- | --- |
| Score | 0 |


---

|  |  |
| --- | --- |
| Reference Count | 3 |


---

|  |  |
| --- | --- |
| Mechanism | Unknown |


---

|  |  |
| --- | --- |
| Reference:0 || Sentence | "Consistent with this indication of a possible direct autoactivation pathway, we also observed that heterologous GATA family proteins GATA-1, GATA-2, and GATA-4 were also capable of inducing GATA-3 expression in developing Stat6-deficient T cells and promote Th2 development." |
| PMID | 11283251 |
| Year | 2001 |
| Species | Mouse |
| Journal | Mol Cell Biol |
| RefScore | 1 |
| Source | PArchNLP |
  |
|


---

|  |  |
| --- | --- |
 Reference:1 || Sentence | "Expression analyses performed on GATA-2 -/- mouse embryos between E9.5 and 10.5 dpc established that: (i) the expression of GATA-3 in the developing CNS of the mouse embryo is dependent on the presence of GATA-2 and (ii) loss of GATA-2 leads to severe defects in neurogenesis, which strongly suggests that GATA-2 is involved, as in hematopoiesis, in the maintenance of the pool of ventral neuronal progenitors." |
| PMID | 10357893 |
| Year | 1999 |
| Species | Mouse |
| Journal | Dev Biol |
| RefScore | 2 |
| Source | PArchNLP |
  ||


---

|  |  |
| --- | --- |
 Reference:2 || Sentence | "In particular, the absence of Hoxb1 results in the loss of GATA2 expression in r4 and the absence of GATA2 results in the loss of GATA3 expression." |
| PMID | 10556076 |
| Year | 1999 |
| Species | Mouse |
| Journal | Development |
| RefScore | 1 |
| Source | PArchNLP |
  |


---

|  |  |
| --- | --- |
