# Supplementary material for: Dynamic transcriptomic profiles of zebrafish gills in response to zinc supplementation
Source: BMC Genomics. 2010 Oct 11;11:553. doi: 10.1186/1471-2164-11-553 (PMC3091702; doi:10.1186/1471-2164-11-553)
Supplement: Additional file 2 — Interactive Direct Interaction Network representing the molecular interactions between zinc, copper, iron, calcium and proteins encoded by transcripts changed by zinc supplementation. Mini web-site containing index.html and hyperlinked pages in subdirectory describing a Direct Interaction Network automatically generated based on curated interactions contained within the proprietary PathwayArchitect database. Ovals represent proteins and the circles symbolize metal ions. Objects are coloured by their abundance in zebrafish at the time-point they were significantly different from the control is a scale from -4 fold (dark green) to +4 fold (dark red). Where significant differences were found at more than one time-point, the colour overlay shows expression at the first instance. Dark blue squares denote 'binding', and light blue squares 'expression'; green squares stand for 'regulation', green diamonds for 'metabolism', and green circles for 'promoter binding'. Arrow heads indicate directionality of the interaction where annotated. All nodes and edges can be further interrogated by selecting the relative area of the image. [file 1471-2164-11-553-S2.zip › PathwayArchitect Zn xs DIN/126923.html]

# PROTEIN: USP15

|  |  |
| --- | --- |
| Name | USP15 |
| Type | PROTEIN |
| Description | ubiquitin specific peptidase 15 |
| Note | Ubiquitin (MIM 191339), a highly conserved protein involved in the regulation of intracellular protein breakdown, cell cycle regulation, and stress response, is released from degraded proteins by disassembly of the polyubiquitin chains. The disassembly process is mediated by ubiquitin-specific proteases (USPs). Also see USP1 (MIM 603478).[supplied by OMIM] |
| Alias | AI327321 |
|  | ubiquitin specific protease 15 |
|  | mKIAA0529 |
|  | Unph-2 |
|  | 4921514G19Rik |
|  | Ubiquitin-specific processing protease 15 |
|  | E430033I05Rik |
|  | deubiquitinating enzyme |
|  | granule cell antiserum positive 18 |
|  | Ubiquitin thiolesterase 15 |
|  | KIAA0529 |
|  | Unph4 |
|  | Deubiquitinating enzyme 15 |
|  | USP15 |
|  | UNPH4 |
|  | Gcap18 |
|  | Ubp109 |
|  | Usp15 |
|  | MGC74854 |


---

|  |  |
| --- | --- |
| GO ID | GO:0004843 |
|  | GO:0008233 |
|  | GO:0008234 |
|  | GO:0006511 |
|  | GO:0016787 |
|  | GO:0004197 |
|  | GO:0004221 |
|  | GO:0006512 |


---

|  |  |
| --- | --- |
| MIM | MIM:604731 |


---

|  |  |
| --- | --- |
| Connectivity | 33 |


---

|  |  |
| --- | --- |
| Entrez ID | 14479 |
|  | 171329 |
|  | 9958 |


---

|  |  |
| --- | --- |
| Agilent ID | A\_53\_P148895 |
|  | A\_53\_P138352 |
|  | A\_14\_P125673 |
|  | A\_51\_P213898 |
|  | A\_23\_P204269 |
|  | A\_51\_P213896 |
|  | A\_14\_P130232 |
|  | A\_53\_P141957 |
|  | A\_44\_P225493 |
|  | A\_52\_P1036554 |
|  | A\_52\_P639122 |
|  | A\_52\_P475033 |
|  | A\_14\_P133076 |
|  | A\_14\_P130406 |
|  | A\_32\_P57057 |
|  | A\_52\_P6073 |
|  | A\_52\_P124812 |
|  | A\_23\_P325802 |
|  | A\_43\_P15373 |
|  | A\_14\_P119912 |


---

|  |  |
| --- | --- |
| Pathway | Zn xs inventory |
|  | Zn xs DIN |


---

|  |  |
| --- | --- |
| GO Process | ubiquitin-dependent protein catabolism |
|  | ubiquitin cycle |


---

|  |  |
| --- | --- |
| UniGene | Mm.244209 |
|  | Hs.434951 |
|  | Rn.6294 |


---

|  |  |
| --- | --- |
| Affymetrix Probeset ID | 116702\_at |
|  | 1370460\_at |
|  | 1377004\_at |
|  | 1446186\_at |
|  | 1454036\_a\_at |
|  | 161870\_at |
|  | 209475\_at |
|  | 210681\_s\_at |
|  | 231990\_at |
|  | 233468\_at |
|  | 233473\_3p\_x\_at |
|  | 233473\_x\_at |
|  | 34295\_at |
|  | 43244\_at |
|  | 55389\_r\_at |
|  | 56384\_i\_at |
|  | 56386\_r\_at |
|  | 67717\_at |
|  | 74038\_at |
|  | 78173\_at |
|  | 79745\_at |
|  | 95416\_at |
|  | aa261680\_s\_at |
|  | g5231132\_3p\_a\_at |
|  | g5814096\_3p\_at |
|  | Hs.132288.0.S1\_3p\_at |
|  | Hs.268843.0.S1\_3p\_x\_at |
|  | Msa.11511.0\_at |
|  | Msa.22007.0\_s\_at |
|  | rc\_AA800184\_at |
|  | 115236\_at |
|  | 1436891\_at |
|  | 1455842\_x\_at |
|  | 242695\_at |
|  | 244028\_at |
|  | 46464\_at |
|  | Hs.103173.0.A1\_3p\_at |
|  | RC\_AA253442\_at |
|  | RC\_H58004\_at |
|  | RC\_N62461\_at |
|  | RC\_N64852\_at |
|  | RC\_T66889\_at |
|  | TC41489\_at |


---

|  |  |
| --- | --- |
| EC Number | EC 3.1.2.15 |


---

|  |  |
| --- | --- |
| GO Function | hydrolase activity |
|  | ubiquitin-specific protease activity |
|  | peptidase activity |
|  | cysteine-type peptidase activity |
|  | cysteine-type endopeptidase activity |
|  | ubiquitin thiolesterase activity |


---

|  |  |
| --- | --- |
| Nucleotide | AK202182 |
|  | AK084098 |
|  | BC050042 |
|  | AF468037 |
|  | AF106657 |
|  | BC063454 |
|  | AK083303 |
|  | AK216706 |
|  | AK046332 |
|  | BC003978 |
|  | AF106069 |
|  | AA824120 |
|  | NM\_006313 |
|  | AF013990 |
|  | AK052398 |
|  | AK088515 |
|  | AK161957 |
|  | AK163315 |
|  | L10903 |
|  | AK088952 |
|  | AK163284 |
|  | AF153604 |
|  | AK122301 |
|  | AB011101 |
|  | NM\_027604 |
|  | AK168755 |
|  | BC020688 |
|  | AK164382 |
|  | AK133852 |
|  | AK161771 |
|  | AK023703 |
|  | AK145749 |
|  | NM\_145184 |
|  | AK161469 |
|  | AK014891 |


---

|  |  |
| --- | --- |
| Protein | BAE40593 |
|  | NP\_006304 |
|  | AAL77418 |
|  | NP\_660185 |
|  | AAH20688 |
|  | Q8R5H1 |
|  | AAG28973 |
|  | AAD41086 |
|  | Q9Y4E8 |
|  | BAC65583 |
|  | BAE26626 |
|  | BAE36413 |
|  | BAC38854 |
|  | BAC32683 |
|  | AAF14188 |
|  | AAD52099 |
|  | BAE37763 |
|  | BAB14648 |
|  | BAE37275 |
|  | BAE36566 |
|  | AAH63454 |
|  | BAE21887 |
|  | AAH50042 |
|  | NP\_081880 |
|  | BAA25455 |


---

|  |  |
| --- | --- |
| Organism | Mammal |


---

|  |  |
| --- | --- |
| Location | chromosome 12, 12q14 (Homo sapiens) |
|  | chromosome 7, 7q22 (Rattus norvegicus) |
|  | chromosome 10, 10 D3 (Mus musculus) |


---

|  |  |
| --- | --- |
