# Supplementary material for: Dynamic transcriptomic profiles of zebrafish gills in response to zinc supplementation
Source: BMC Genomics. 2010 Oct 11;11:553. doi: 10.1186/1471-2164-11-553 (PMC3091702; doi:10.1186/1471-2164-11-553)
Supplement: Additional file 2 — Interactive Direct Interaction Network representing the molecular interactions between zinc, copper, iron, calcium and proteins encoded by transcripts changed by zinc supplementation. Mini web-site containing index.html and hyperlinked pages in subdirectory describing a Direct Interaction Network automatically generated based on curated interactions contained within the proprietary PathwayArchitect database. Ovals represent proteins and the circles symbolize metal ions. Objects are coloured by their abundance in zebrafish at the time-point they were significantly different from the control is a scale from -4 fold (dark green) to +4 fold (dark red). Where significant differences were found at more than one time-point, the colour overlay shows expression at the first instance. Dark blue squares denote 'binding', and light blue squares 'expression'; green squares stand for 'regulation', green diamonds for 'metabolism', and green circles for 'promoter binding'. Arrow heads indicate directionality of the interaction where annotated. All nodes and edges can be further interrogated by selecting the relative area of the image. [file 1471-2164-11-553-S2.zip › PathwayArchitect Zn xs DIN/1273919.html]

# BINDING:

|  |  |
| --- | --- |
| Type | BINDING |
| Effect | None |


---

|  |  |
| --- | --- |
| Score | 0 |


---

|  |  |
| --- | --- |
| Reference Count | 5 |


---

|  |  |
| --- | --- |
| Mechanism | Unknown |


---

|  |  |
| --- | --- |
| Reference:0 || Sentence | "We have studied the individual roles and the interaction of Cu,Zn-superoxide dismutase (SOD) and catalase (CAT) in transfectants with human cDNAs of mouse epidermal cells JB6 clone 41." |
| PMID | 1654093 |
| Year | 1991 |
| Species | Mouse |
| Journal | Biochemistry |
| RefScore | 0 |
| Source | PArchNLP |
  |
|


---

|  |  |
| --- | --- |
 Reference:1 || Sentence | "Nine chlorin complexes of Co, Cu and Zn were synthesized for the first time." |
| PMID | 12016927 |
| Year | 1998 |
| Species | Mouse |
| Journal | Yao Xue Xue Bao |
| RefScore | 0 |
| Source | PArchNLP |
  ||


---

|  |  |
| --- | --- |
 Reference:2 || Sentence | For example, in addition to the well-characterized soluble Cu/Zn enzyme (Sod) and mitochondrial manganese-containing form (Sod2), Drosophila melanogaster is found to contain a putative copper chaperone (CCS), an extracellular Cu/Zn enzyme (Sod3), and an extracellular protein distantly related to the Cu/Zn forms (Sodq). |
| PMID | 15664623 |
| Year | 2005 |
| Species | Human |
| Journal | Mech Ageing Dev |
| RefScore | 0 |
| Source | PArchNLP |
  ||


---

|  |  |
| --- | --- |
 Reference:3 || Sentence | One explanation for these observations is that Zn induces the synthesis of metallothionein, which binds Cu for which it has a higher affinity. |
| PMID | 16083871 |
| Year | 2005 |
| Species | Rat |
| Journal | Chem Biol Interact |
| RefScore | 1 |
| Source | PArchNLP |
  ||


---

|  |  |
| --- | --- |
 Reference:4 || Sentence | Many proteins require the binding of trace metals such as Ca, Fe, Cu, or Zn, which may modulate their structure, function, or activity. |
| PMID | 16495082 |
| Year | 2006 |
| Species | Human |
| Journal | J Struct Biol |
| RefScore | 1 |
| Source | PArchNLP |
  |


---

|  |  |
| --- | --- |
