# Supplementary material for: Dynamic transcriptomic profiles of zebrafish gills in response to zinc supplementation
Source: BMC Genomics. 2010 Oct 11;11:553. doi: 10.1186/1471-2164-11-553 (PMC3091702; doi:10.1186/1471-2164-11-553)
Supplement: Additional file 2 — Interactive Direct Interaction Network representing the molecular interactions between zinc, copper, iron, calcium and proteins encoded by transcripts changed by zinc supplementation. Mini web-site containing index.html and hyperlinked pages in subdirectory describing a Direct Interaction Network automatically generated based on curated interactions contained within the proprietary PathwayArchitect database. Ovals represent proteins and the circles symbolize metal ions. Objects are coloured by their abundance in zebrafish at the time-point they were significantly different from the control is a scale from -4 fold (dark green) to +4 fold (dark red). Where significant differences were found at more than one time-point, the colour overlay shows expression at the first instance. Dark blue squares denote 'binding', and light blue squares 'expression'; green squares stand for 'regulation', green diamonds for 'metabolism', and green circles for 'promoter binding'. Arrow heads indicate directionality of the interaction where annotated. All nodes and edges can be further interrogated by selecting the relative area of the image. [file 1471-2164-11-553-S2.zip › PathwayArchitect Zn xs DIN/127409.html]

# PROTEIN: H2AFX

|  |  |
| --- | --- |
| Name | H2AFX |
| Type | PROTEIN |
| Description | H2A histone family, member X |
| Note | Histones are basic nuclear proteins that are responsible for the nucleosome structure of the chromosomal fiber in eukaryotes. Two molecules of each of the four core histones (H2A, H2B, H3, and H4) form an octamer, around which approximately 146 bp of DNA is wrapped in repeating units, called nucleosomes. The linker histone, H1, interacts with linker DNA between nucleosomes and functions in the compaction of chromatin into higher order structures. This gene encodes a member of the histone H2A family, and generates two transcripts through the use of the conserved stem-loop termination motif, and the polyA addition motif. |
| Alias | H2AX histone |
|  | histone 5 protein 2ax |
|  | Hist5-2ax |
|  | H2a/x |
|  | H2AX |
|  | H2a.x |
|  | Dpagt1 |
|  | H2AFX |
|  | Histone H2A.X |
|  | H2afx |
|  | AW228881 |
|  | H2A/X |
|  | H2A.X |
|  | H2ax |


---

|  |  |
| --- | --- |
| GO Component | chromatin |
|  | chromosome |
|  | nucleus |
|  | replication fork |
|  | nucleosome |


---

|  |  |
| --- | --- |
| GO ID | GO:0003677 |
|  | GO:0007001 |
|  | GO:0007283 |
|  | GO:0006974 |
|  | GO:0000786 |
|  | GO:0005634 |
|  | GO:0006281 |
|  | GO:0000785 |
|  | GO:0000077 |
|  | GO:0006334 |
|  | GO:0005694 |
|  | GO:0003684 |
|  | GO:0007126 |
|  | GO:0000724 |
|  | GO:0007049 |
|  | GO:0006310 |
|  | GO:0005657 |


---

|  |  |
| --- | --- |
| MIM | MIM:601772 |


---

|  |  |
| --- | --- |
| Connectivity | 215 |


---

|  |  |
| --- | --- |
| Entrez ID | 15270 |
|  | 300668 |
|  | 3014 |


---

|  |  |
| --- | --- |
| Agilent ID | A\_14\_P103190 |
|  | A\_14\_P121155 |
|  | A\_23\_P319432 |
|  | A\_51\_P245275 |
|  | A\_44\_P266776 |
|  | A\_32\_P775491 |
|  | A\_24\_P38895 |
|  | A\_23\_P47370 |


---

|  |  |
| --- | --- |
| Cellular Localization | Nucleus |
|  | Chromosome |
|  | Organelle |
|  | Cell |


---

|  |  |
| --- | --- |
| DbXref | Reactome##73894##DNA Repair##http://www.reactome.org/cgi-bin/eventbrowser?DB=gk\_current&ID=73894 |


---

|  |  |
| --- | --- |
| Pathway | Zn xs inventory |
|  | Zn xs DIN |


---

|  |  |
| --- | --- |
| GO Process | NOT DNA damage checkpoint |
|  | response to DNA damage stimulus |
|  | DNA repair |
|  | meiosis |
|  | spermatogenesis |
|  | cell cycle |
|  | nucleosome assembly |
|  | double-strand break repair via homologous recombination |
|  | DNA recombination |
|  | chromosome organization and biogenesis (sensu Eukaryota) |


---

|  |  |
| --- | --- |
| UniGene | Hs.477879 |
|  | Rn.129301 |
|  | Mm.245931 |


---

|  |  |
| --- | --- |
| Affymetrix Probeset ID | 1372270\_at |
|  | 1398147\_at |
|  | 1416746\_at |
|  | 205436\_s\_at |
|  | 212524\_x\_at |
|  | 212525\_3p\_s\_at |
|  | 212525\_s\_at |
|  | 213344\_s\_at |
|  | 40195\_at |
|  | 93019\_at |
|  | aa269806\_s\_at |
|  | g4504252\_3p\_a\_at |
|  | Hs.147097.1.A1\_3p\_a\_at |
|  | Hs.147097.1.S1\_3p\_at |
|  | Hs.147097.1.S1\_3p\_x\_at |
|  | Hs.147097.2.S1\_3p\_a\_at |
|  | rc\_AA963476\_at |
|  | X14850\_at |
|  | X58069\_s\_at |
|  | AA252929\_at |


---

|  |  |
| --- | --- |
| GO Function | damaged DNA binding |
|  | DNA binding |


---

|  |  |
| --- | --- |
| Nucleotide | D43966 |
|  | NM\_010436 |
|  | BC004915 |
|  | X58069 |
|  | X14850 |
|  | BC013416 |
|  | BC063184 |
|  | BC010336 |
|  | BC011694 |
|  | BC005468 |
|  | CR457079 |
|  | AK008124 |
|  | NM\_199388 |
|  | Z35401 |
|  | DQ015918 |
|  | NM\_002105 |


---

|  |  |
| --- | --- |
| Protein | AAH11694 |
|  | P16104 |
|  | CAA32968 |
|  | AAH13416 |
|  | P27661 |
|  | AAH10336 |
|  | CAA84585 |
|  | NP\_034566 |
|  | CAG33360 |
|  | AAY22178 |
|  | AAH63184 |
|  | AAH05468 |
|  | BAA21042 |
|  | NP\_002096 |
|  | NP\_955420 |
|  | CAA41099 |
|  | AAH04915 |


---

|  |  |
| --- | --- |
| Organism | Mammal |


---

|  |  |
| --- | --- |
| Location | chromosome 9, 9 26.0 cM, 9 A5.2 (Mus musculus) |
|  | chromosome 11, 11q23.2-q23.3 (Homo sapiens) |
|  | chromosome 8, 8q22 (Rattus norvegicus) |
|  | 9 26.0 cM (Mus musculus) |


---

|  |  |
| --- | --- |
