# Supplementary material for: Dynamic transcriptomic profiles of zebrafish gills in response to zinc supplementation
Source: BMC Genomics. 2010 Oct 11;11:553. doi: 10.1186/1471-2164-11-553 (PMC3091702; doi:10.1186/1471-2164-11-553)
Supplement: Additional file 2 — Interactive Direct Interaction Network representing the molecular interactions between zinc, copper, iron, calcium and proteins encoded by transcripts changed by zinc supplementation. Mini web-site containing index.html and hyperlinked pages in subdirectory describing a Direct Interaction Network automatically generated based on curated interactions contained within the proprietary PathwayArchitect database. Ovals represent proteins and the circles symbolize metal ions. Objects are coloured by their abundance in zebrafish at the time-point they were significantly different from the control is a scale from -4 fold (dark green) to +4 fold (dark red). Where significant differences were found at more than one time-point, the colour overlay shows expression at the first instance. Dark blue squares denote 'binding', and light blue squares 'expression'; green squares stand for 'regulation', green diamonds for 'metabolism', and green circles for 'promoter binding'. Arrow heads indicate directionality of the interaction where annotated. All nodes and edges can be further interrogated by selecting the relative area of the image. [file 1471-2164-11-553-S2.zip › PathwayArchitect Zn xs DIN/129519.html]

# PROTEIN: EIF4G2

|  |  |
| --- | --- |
| Name | EIF4G2 |
| Type | PROTEIN |
| Description | eukaryotic translation initiation factor 4 gamma, 2 |
| Note | Translation initiation is mediated by specific recognition of the cap structure by eukaryotic translation initiation factor 4F (eIF4F), which is a cap binding protein complex that consists of three subunits: eIF4A, eIF4E and eIF4G. The protein encoded by this gene shares similarity with the C-terminal region of eIF4G, that contains the binding sites for eIF4A and eIF3; eIF4G in addition, contains a binding site for eIF4E at the N-terminus. Unlike eIF4G which supports cap-dependent and independent translation, this gene product functions as a general repressor of translation by forming translationally inactive complexes. In vitro and in vivo studies indicate that translation of this mRNA initiates exclusively at a non-AUG (GUG) codon. |
| Alias | apobec-1 target 1 |
|  | death-associated protein 5 |
|  | NAT1 |
|  | p97 |
|  | DAP-5 |
|  | eukaryotic translation initiation factor 4G-like 1 |
|  | Nat1 |
|  | Death associated protein 5 |
|  | eIF-4G 2 |
|  | DAP5;ORFNames=OK/SW-cl.75 |
|  | translation repressor Nat1 |
|  | DAP5 |
|  | DRCF-6 |
|  | eIF4G 2 |
|  | MGC109314 |
|  | eIF-4-gamma 2 |
|  | Natm1 |
|  | EIF4G2 |


---

|  |  |
| --- | --- |
| GO Component | eukaryotic translation initiation factor 4F complex |
|  | cytoplasm |


---

|  |  |
| --- | --- |
| GO ID | GO:0003723 |
|  | GO:0008219 |
|  | GO:0005737 |
|  | GO:0007050 |
|  | GO:0006417 |
|  | GO:0003743 |
|  | GO:0006446 |
|  | GO:0006412 |
|  | GO:0006445 |
|  | GO:0016281 |


---

|  |  |
| --- | --- |
| MIM | MIM:602325 |


---

|  |  |
| --- | --- |
| Connectivity | 48 |


---

|  |  |
| --- | --- |
| Entrez ID | 171362 |
|  | 13690 |
|  | 1982 |


---

|  |  |
| --- | --- |
| Agilent ID | A\_53\_P106467 |
|  | A\_51\_P306066 |
|  | A\_14\_P107003 |
|  | A\_52\_P41668 |
|  | A\_23\_P104892 |
|  | A\_52\_P460526 |
|  | A\_14\_P103841 |
|  | A\_53\_P127024 |


---

|  |  |
| --- | --- |
| Cellular Localization | Cytoplasm |
|  | Cell |


---

|  |  |
| --- | --- |
| Pathway | Zn def RIN |
|  | Master Regulators |
|  | Zn xs inventory |
|  | Zn xs DIN |


---

|  |  |
| --- | --- |
| GO Process | cell death |
|  | regulation of protein biosynthesis |
|  | cell cycle arrest |
|  | regulation of translational initiation |
|  | regulation of translation |
|  | protein biosynthesis |


---

|  |  |
| --- | --- |
| UniGene | Rn.103276 |
|  | Rn.121213 |
|  | Mm.185453 |
|  | Hs.183684 |


---

|  |  |
| --- | --- |
| Affymetrix Probeset ID | 240381\_at |
|  | 1415863\_at |
|  | 1452758\_s\_at |
|  | 1458431\_at |
|  | 1557964\_at |
|  | 165103\_f\_at |
|  | 200004\_at |
|  | 217607\_x\_at |
|  | 41785\_at |
|  | 1392715\_at |
|  | 80747\_at |
|  | 87748\_r\_at |
|  | 89110\_at |
|  | g4503538\_3p\_at |
|  | Hs.150904.0.S1\_3p\_at |
|  | Hs.150904.0.S1\_3p\_x\_at |
|  | Hs2.190503.1.S1\_3p\_at |
|  | u63323\_s\_at |
|  | U73824\_at |
|  | 1428363\_at |
|  | 1428362\_at |
|  | 100535\_at |
|  | 66990\_at |
|  | 68648\_at |
|  | 68650\_g\_at |
|  | 92014\_s\_at |
|  | 92017\_r\_at |
|  | 92018\_at |
|  | 92021\_g\_at |
|  | Hs.192440.0.A1\_3p\_at |
|  | 1388359\_at |
|  | TC33211\_at |
|  | TC33212\_at |
|  | TC33212\_g\_at |
|  | TC33213\_at |
|  | 1397520\_at |
|  | 1367469\_at |
|  | U95052UTR#1\_s\_at |
|  | rc\_AI101150\_at |
|  | rc\_AI179327\_at |


---

|  |  |
| --- | --- |
| GO Function | RNA binding |
|  | translation initiation factor activity |


---

|  |  |
| --- | --- |
| Nucleotide | AK151637 |
|  | BC039274 |
|  | BC039851 |
|  | BC057673 |
|  | BC092521 |
|  | BC040391 |
|  | AI874618 |
|  | BC056387 |
|  | BC010654 |
|  | U76111 |
|  | U95052 |
|  | BC043149 |
|  | NM\_001418 |
|  | U63323 |
|  | AK195045 |
|  | X89713 |
|  | AK223548 |
|  | BC018746 |
|  | AK144309 |
|  | BC018975 |
|  | AK141512 |
|  | BC065276 |
|  | NM\_001017374 |
|  | AB209267 |
|  | BC064810 |
|  | U73824 |
|  | BC091330 |
|  | BC014930 |
|  | AK150745 |
|  | BX647799 |
|  | AB063323 |
|  | BC043034 |
|  | U76112 |
|  | NM\_013507 |


---

|  |  |
| --- | --- |
| Protein | AAH10654 |
|  | AAH40391 |
|  | BAE30567 |
|  | CAA61857 |
|  | BAE24708 |
|  | BAD92504 |
|  | NP\_038535 |
|  | AAH64810 |
|  | NP\_001409 |
|  | AAH39851 |
|  | P78344 |
|  | AAH14930 |
|  | AAB49973 |
|  | NP\_001017374 |
|  | AAH43149 |
|  | AAC53095 |
|  | AAC53030 |
|  | BAE29817 |
|  | BAE25826 |
|  | AAH43034 |
|  | BAD97268 |
|  | Q62448 |
|  | AAH91330 |
|  | AAC51166 |
|  | BAB93515 |


---

|  |  |
| --- | --- |
| Organism | Mammal |


---

|  |  |
| --- | --- |
| Location | 7 51.52 cM (Mus musculus) |
|  | chromosome 7, 7 51.52 cM, 7 E3 (Mus musculus) |
|  | chromosome 1, 1q33 (Rattus norvegicus) |
|  | chromosome 11, 11p15 (Homo sapiens) |


---

|  |  |
| --- | --- |
