# Supplementary material for: Dynamic transcriptomic profiles of zebrafish gills in response to zinc supplementation
Source: BMC Genomics. 2010 Oct 11;11:553. doi: 10.1186/1471-2164-11-553 (PMC3091702; doi:10.1186/1471-2164-11-553)
Supplement: Additional file 2 — Interactive Direct Interaction Network representing the molecular interactions between zinc, copper, iron, calcium and proteins encoded by transcripts changed by zinc supplementation. Mini web-site containing index.html and hyperlinked pages in subdirectory describing a Direct Interaction Network automatically generated based on curated interactions contained within the proprietary PathwayArchitect database. Ovals represent proteins and the circles symbolize metal ions. Objects are coloured by their abundance in zebrafish at the time-point they were significantly different from the control is a scale from -4 fold (dark green) to +4 fold (dark red). Where significant differences were found at more than one time-point, the colour overlay shows expression at the first instance. Dark blue squares denote 'binding', and light blue squares 'expression'; green squares stand for 'regulation', green diamonds for 'metabolism', and green circles for 'promoter binding'. Arrow heads indicate directionality of the interaction where annotated. All nodes and edges can be further interrogated by selecting the relative area of the image. [file 1471-2164-11-553-S2.zip › PathwayArchitect Zn xs DIN/130672.html]

# PROTEIN: RPS26

|  |  |
| --- | --- |
| Name | RPS26 |
| Type | PROTEIN |
| Description | ribosomal protein S26 |
| Note | Ribosomes, the organelles that catalyze protein synthesis, consist of a small 40S subunit and a large 60S subunit. Together these subunits are composed of 4 RNA species and approximately 80 structurally distinct proteins. This gene encodes a ribosomal protein that is a component of the 40S subunit. The protein belongs to the S26E family of ribosomal proteins. It is located in the cytoplasm. As is typical for genes encoding ribosomal proteins, there are multiple processed pseudogenes of this gene dispersed through the genome. |
| Alias | 40S ribosomal protein S26 |
|  | Rps26 |
|  | RPS26 |


---

|  |  |
| --- | --- |
| GO Component | ribosome |
|  | ribonucleoprotein complex |
|  | cellular component unknown |
|  | intracellular |
|  | cytosolic small ribosomal subunit (sensu Eukaryota) |


---

|  |  |
| --- | --- |
| GO ID | GO:0005840 |
|  | GO:0003723 |
|  | GO:0005622 |
|  | GO:0000004 |
|  | GO:0008372 |
|  | GO:0030529 |
|  | GO:0005554 |
|  | GO:0006412 |
|  | GO:0005843 |
|  | GO:0003735 |


---

|  |  |
| --- | --- |
| MIM | MIM:603701 |


---

|  |  |
| --- | --- |
| Connectivity | 9 |


---

|  |  |
| --- | --- |
| Entrez ID | 27139 |
|  | 6231 |
|  | 27370 |


---

|  |  |
| --- | --- |
| Agilent ID | A\_32\_P812234 |
|  | A\_32\_P52261 |
|  | A\_24\_P638294 |
|  | A\_44\_P388170 |
|  | A\_24\_P289404 |
|  | A\_23\_P320082 |
|  | A\_23\_P116694 |
|  | A\_53\_P162246 |
|  | A\_14\_P133425 |
|  | A\_51\_P302675 |
|  | A\_32\_P47994 |
|  | A\_32\_P70834 |
|  | A\_32\_P75256 |
|  | A\_43\_P11759 |
|  | A\_53\_P121113 |
|  | A\_32\_P129148 |
|  | A\_32\_P140440 |


---

|  |  |
| --- | --- |
| Cellular Localization | Ribosome |
|  | Cytoplasm |
|  | Cell |
|  | Cytosol |
|  | Organelle |


---

|  |  |
| --- | --- |
| DbXref | Reactome##157279##157279##http://www.reactome.org/cgi-bin/eventbrowser?DB=gk\_current&ID=157279 |
|  | KEGG pathway##03010##Ribosome##http://www.genome.jp/dbget-bin/show\_pathway?rno03010+27139 |
|  | KEGG pathway##03010##Ribosome##http://www.genome.jp/dbget-bin/show\_pathway?mmu03010+27370 |
|  | Reactome##74160##Gene Expression##http://www.reactome.org/cgi-bin/eventbrowser?DB=gk\_current&ID=74160 |
|  | KEGG pathway##03010##Ribosome##http://www.genome.jp/dbget-bin/show\_pathway?hsa03010+6231 |


---

|  |  |
| --- | --- |
| Pathway | Zn def RIN |
|  | Master Regulators |
|  | Zn xs inventory |
|  | Zn xs DIN |


---

|  |  |
| --- | --- |
| GO Process | biological process unknown |
|  | protein biosynthesis |


---

|  |  |
| --- | --- |
| UniGene | Mm.261679 |
|  | Hs.355957 |
|  | Rn.2388 |
|  | Hs.137367 |
|  | Hs.447562 |
|  | Rn.1059 |


---

|  |  |
| --- | --- |
| Affymetrix Probeset ID | 1367596\_at |
|  | 137217\_f\_at |
|  | 217753\_s\_at |
|  | 1415876\_a\_at |
|  | 37382\_at |
|  | 98564\_f\_at |
|  | C76830\_rc\_f\_at |
|  | Msa.3011.0\_r\_at |
|  | Msa.3011.0\_s\_at |
|  | rc\_AI014087\_at |
|  | U67770\_f\_at |
|  | 98565\_at |
|  | D55051\_i\_at |
|  | TC39510\_f\_at |
|  | X69654\_at |
|  | g4506708\_3p\_a\_at |


---

|  |  |
| --- | --- |
| GO Function | structural constituent of ribosome |
|  | RNA binding |
|  | molecular function unknown |


---

|  |  |
| --- | --- |
| Nucleotide | U41448 |
|  | BC081452 |
|  | NM\_013224 |
|  | U67770 |
|  | AK012722 |
|  | AK152132 |
|  | NM\_001029 |
|  | BC036987 |
|  | AB007161 |
|  | BC013215 |
|  | AK010689 |
|  | BC070220 |
|  | BC100456 |
|  | X69654 |
|  | AK139113 |
|  | AK018702 |
|  | NM\_013765 |
|  | AK008301 |
|  | BC061561 |
|  | X79236 |
|  | X02414 |
|  | AB007160 |
|  | BC015832 |
|  | BC002604 |
|  | X77770 |


---

|  |  |
| --- | --- |
| Protein | NP\_037356 |
|  | P62856 |
|  | BAE30973 |
|  | CAA54808 |
|  | P62855 |
|  | CAA55818 |
|  | BAB31353 |
|  | AAI00457 |
|  | AAC26987 |
|  | AAH36987 |
|  | CAA26264 |
|  | CAA49345 |
|  | AAH70220 |
|  | AAB07729 |
|  | BAB25586 |
|  | AAH02604 |
|  | BAB28433 |
|  | AAH61561 |
|  | AAH81452 |
|  | AAH15832 |
|  | BAA25823 |
|  | NP\_001020 |
|  | BAA25824 |
|  | P62854 |
|  | BAB27121 |
|  | NP\_038793 |


---

|  |  |
| --- | --- |
| Organism | Mammal |


---

|  |  |
| --- | --- |
| Location | chromosome 7, 7q11 (Rattus norvegicus) |
|  | chromosome 12, 12q13 (Homo sapiens) |
|  | chromosome 10, 10 D3 (Mus musculus) |


---

|  |  |
| --- | --- |
