# Supplementary material for: Dynamic transcriptomic profiles of zebrafish gills in response to zinc supplementation
Source: BMC Genomics. 2010 Oct 11;11:553. doi: 10.1186/1471-2164-11-553 (PMC3091702; doi:10.1186/1471-2164-11-553)
Supplement: Additional file 2 — Interactive Direct Interaction Network representing the molecular interactions between zinc, copper, iron, calcium and proteins encoded by transcripts changed by zinc supplementation. Mini web-site containing index.html and hyperlinked pages in subdirectory describing a Direct Interaction Network automatically generated based on curated interactions contained within the proprietary PathwayArchitect database. Ovals represent proteins and the circles symbolize metal ions. Objects are coloured by their abundance in zebrafish at the time-point they were significantly different from the control is a scale from -4 fold (dark green) to +4 fold (dark red). Where significant differences were found at more than one time-point, the colour overlay shows expression at the first instance. Dark blue squares denote 'binding', and light blue squares 'expression'; green squares stand for 'regulation', green diamonds for 'metabolism', and green circles for 'promoter binding'. Arrow heads indicate directionality of the interaction where annotated. All nodes and edges can be further interrogated by selecting the relative area of the image. [file 1471-2164-11-553-S2.zip › PathwayArchitect Zn xs DIN/131016.html]

# PROTEIN: MB

|  |  |
| --- | --- |
| Name | MB |
| Type | PROTEIN |
| Description | myoglobin |
| Note | This gene encodes a member of the globin superfamily and is expressed in skeletal and cardiac muscles. The encoded protein is a haemoprotein contributing to intracellular oxygen storage and transcellular facilitated diffusion of oxygen. At least three alternatively spliced transcript variants encoding the same protein have been reported. |
| Alias | MGC13548 |
|  | AI325109 |
|  | PVALB |
|  | MB |
|  | Mb |
|  | Myoglobin |


---

|  |  |
| --- | --- |
| GO ID | GO:0006810 |
|  | GO:0015671 |
|  | GO:0020037 |
|  | GO:0005506 |
|  | GO:0005489 |
|  | GO:0007517 |
|  | GO:0019825 |
|  | GO:0005488 |
|  | GO:0001666 |
|  | GO:0046872 |
|  | GO:0043353 |
|  | GO:0005344 |


---

|  |  |
| --- | --- |
| MIM | MIM:160000 |


---

|  |  |
| --- | --- |
| Connectivity | 1216 |


---

|  |  |
| --- | --- |
| Entrez ID | 17189 |
|  | 59108 |
|  | 4151 |


---

|  |  |
| --- | --- |
| Agilent ID | A\_53\_P166669 |
|  | A\_42\_P765066 |
|  | A\_43\_P12081 |
|  | A\_14\_P122961 |
|  | A\_51\_P193185 |
|  | A\_44\_P257201 |
|  | A\_53\_P168260 |
|  | A\_53\_P148673 |
|  | A\_53\_P156636 |
|  | A\_23\_P6433 |


---

|  |  |
| --- | --- |
| Pathway | Zn def RIN |
|  | Master Regulators |
|  | Zn xs inventory |
|  | Zn xs DIN |


---

|  |  |
| --- | --- |
| GO Process | oxygen transport |
|  | transport |
|  | enucleate erythrocyte differentiation |
|  | response to hypoxia |
|  | muscle development |


---

|  |  |
| --- | --- |
| UniGene | Mm.201606 |
|  | Hs.517586 |
|  | Rn.40511 |


---

|  |  |
| --- | --- |
| Affymetrix Probeset ID | 100614\_at |
|  | 1387768\_at |
|  | 1422420\_at |
|  | 207434\_s\_at |
|  | 1451203\_at |
|  | 204179\_at |
|  | 32485\_at |
|  | g4885476\_3p\_at |
|  | rc\_AA946094\_at |
|  | X00371\_rna1\_at |
|  | X04405\_s\_at |
|  | 100615\_at |
|  | 40954\_at |
|  | Msa.2038.0\_at |
|  | TC27670\_at |
|  | TC27670\_g\_at |
|  | g11125763\_3p\_a\_at |


---

|  |  |
| --- | --- |
| GO Function | binding |
|  | oxygen transporter activity |
|  | iron ion binding |
|  | electron transporter activity |
|  | heme binding |
|  | oxygen binding |
|  | metal ion binding |


---

|  |  |
| --- | --- |
| Nucleotide | NM\_203378 |
|  | M10090 |
|  | BC018001 |
|  | BC025172 |
|  | CR456516 |
|  | BQ925917 |
|  | M14602 |
|  | CR541949 |
|  | DQ003030 |
|  | CB140824 |
|  | BC070511 |
|  | X04417 |
|  | X04405 |
|  | NM\_005368 |
|  | NM\_013593 |
|  | BQ956082 |
|  | X00371 |
|  | AK137456 |
|  | NM\_203377 |
|  | NM\_021588 |
|  | AF197916 |
|  | BU585249 |
|  | M14603 |
|  | BC014547 |


---

|  |  |
| --- | --- |
| Protein | BAE23359 |
|  | CAA25109 |
|  | NP\_976311 |
|  | NP\_976312 |
|  | NP\_038621 |
|  | CAG30402 |
|  | AAF05848 |
|  | NP\_005359 |
|  | AAH18001 |
|  | AAH14547 |
|  | P02144 |
|  | AAH25172 |
|  | NP\_067599 |
|  | CAA27994 |
|  | CAG46747 |
|  | AAA59595 |
|  | P04247 |
|  | AAH70511 |
|  | AAX84516 |


---

|  |  |
| --- | --- |
| Organism | Mammal |


---

|  |  |
| --- | --- |
| Location | chromosome 22, 22q13.1 (Homo sapiens) |
|  | chromosome 15, 15 43.3 cM, 15 D3 (Mus musculus) |
|  | chromosome 7, 7q34 (Rattus norvegicus) |
|  | 15 43.3 cM (Mus musculus) |


---

|  |  |
| --- | --- |
