# Supplementary material for: Dynamic transcriptomic profiles of zebrafish gills in response to zinc supplementation
Source: BMC Genomics. 2010 Oct 11;11:553. doi: 10.1186/1471-2164-11-553 (PMC3091702; doi:10.1186/1471-2164-11-553)
Supplement: Additional file 2 — Interactive Direct Interaction Network representing the molecular interactions between zinc, copper, iron, calcium and proteins encoded by transcripts changed by zinc supplementation. Mini web-site containing index.html and hyperlinked pages in subdirectory describing a Direct Interaction Network automatically generated based on curated interactions contained within the proprietary PathwayArchitect database. Ovals represent proteins and the circles symbolize metal ions. Objects are coloured by their abundance in zebrafish at the time-point they were significantly different from the control is a scale from -4 fold (dark green) to +4 fold (dark red). Where significant differences were found at more than one time-point, the colour overlay shows expression at the first instance. Dark blue squares denote 'binding', and light blue squares 'expression'; green squares stand for 'regulation', green diamonds for 'metabolism', and green circles for 'promoter binding'. Arrow heads indicate directionality of the interaction where annotated. All nodes and edges can be further interrogated by selecting the relative area of the image. [file 1471-2164-11-553-S2.zip › PathwayArchitect Zn xs DIN/133071.html]

# PROTEIN: CKM

|  |  |
| --- | --- |
| Name | CKM |
| Type | PROTEIN |
| Description | creatine kinase, muscle |
| Note | The protein encoded by this gene is a cytoplasmic enzyme involved in energy homeostasis and is an important serum marker for myocardial infarction. The encoded protein reversibly catalyzes the transfer of phosphate between ATP and various phosphogens such as creatine phosphate. It acts as a homodimer in striated muscle as well as in other tissues, and as a heterodimer with a similar brain isozyme in heart. The encoded protein is a member of the ATP:guanido phosphotransferase protein family. |
| Alias | CKMM |
|  | CKM |
|  | Creatine kinase, muscle form |
|  | muscle creatine kinase |
|  | Creatine kinase muscle form |
|  | creatine kinase M chain |
|  | M-CK |
|  | creatine kinase-M |
|  | Ckm |
|  | Ckmm |
|  | Creatine kinase, M chain |
|  | MCK |


---

|  |  |
| --- | --- |
| GO Component | cytoplasm |


---

|  |  |
| --- | --- |
| GO ID | GO:0046314 |
|  | GO:0016301 |
|  | GO:0006603 |
|  | GO:0004111 |
|  | GO:0016740 |
|  | GO:0005737 |
|  | GO:0016772 |


---

|  |  |
| --- | --- |
| MIM | MIM:123310 |


---

|  |  |
| --- | --- |
| Connectivity | 114 |


---

|  |  |
| --- | --- |
| Entrez ID | 12715 |
|  | 24265 |
|  | 1158 |


---

|  |  |
| --- | --- |
| Agilent ID | A\_23\_P50250 |
|  | A\_53\_P123335 |
|  | A\_53\_P154316 |
|  | A\_23\_P50247 |
|  | A\_51\_P403942 |
|  | A\_42\_P832832 |
|  | A\_51\_P380807 |
|  | A\_14\_P122988 |


---

|  |  |
| --- | --- |
| Cellular Localization | Cytoplasm |
|  | Cell |


---

|  |  |
| --- | --- |
| DbXref | KEGG pathway##00220##Urea cycle and metabolism of amino groups##http://www.genome.jp/dbget-bin/show\_pathway?rno00220+24265 |
|  | KEGG pathway##00220##Urea cycle and metabolism of amino groups##http://www.genome.jp/dbget-bin/show\_pathway?mmu00220+12715 |
|  | KEGG pathway##00330##Arginine and proline metabolism##http://www.genome.jp/dbget-bin/show\_pathway?hsa00330+1158 |
|  | KEGG pathway##00330##Arginine and proline metabolism##http://www.genome.jp/dbget-bin/show\_pathway?mmu00330+12715 |
|  | KEGG pathway##00220##Urea cycle and metabolism of amino groups##http://www.genome.jp/dbget-bin/show\_pathway?hsa00220+1158 |
|  | KEGG pathway##00330##Arginine and proline metabolism##http://www.genome.jp/dbget-bin/show\_pathway?rno00330+24265 |


---

|  |  |
| --- | --- |
| Pathway | Zn xs inventory |
|  | Zn xs DIN |


---

|  |  |
| --- | --- |
| GO Process | phosphocreatine biosynthesis |
|  | phosphocreatine metabolism |


---

|  |  |
| --- | --- |
| UniGene | Mm.2375 |
|  | Rn.10756 |
|  | Hs.334347 |


---

|  |  |
| --- | --- |
| Affymetrix Probeset ID | 1367626\_at |
|  | 1417614\_at |
|  | 160175\_at |
|  | 161569\_f\_at |
|  | 170247\_r\_at |
|  | 171149\_r\_at |
|  | 204810\_s\_at |
|  | 32486\_at |
|  | g4502852\_3p\_a\_at |
|  | M10140\_at |
|  | M21494\_at |
|  | Msa.19136.0\_f\_at |
|  | Msa.2213.0\_s\_at |
|  | Msa.22440.0\_f\_at |
|  | Msa.29369.0\_s\_at |
|  | X03233\_s\_at |
|  | 1430937\_at |
|  | 1444643\_at |
|  | 96409\_f\_at |
|  | TC15151\_at |
|  | TC40578\_at |


---

|  |  |
| --- | --- |
| EC Number | EC 2.7.3.2 |


---

|  |  |
| --- | --- |
| GO Function | transferase activity |
|  | creatine kinase activity |
|  | kinase activity |
|  | transferase activity, transferring phosphorus-containing groups |


---

|  |  |
| --- | --- |
| Nucleotide | X03233 |
|  | M14780 |
|  | NM\_012530 |
|  | M21494 |
|  | AC005781 |
|  | NM\_007710 |
|  | M10140 |
|  | BC007462 |
|  | M21487 |
|  | AK020867 |
|  | NM\_001824 |
|  | AK046115 |
|  | BT006793 |
|  | AK129878 |
|  | AY585238 |
|  | M14864 |
|  | BC062058 |
|  | AV086797 |
|  | AK137460 |
|  | AK009950 |
|  | M16440 |


---

|  |  |
| --- | --- |
| Protein | AAA96609 |
|  | CAA26979 |
|  | P00564 |
|  | AAA52025 |
|  | NP\_001815 |
|  | AAH07462 |
|  | AAA40936 |
|  | AAA40935 |
|  | NP\_031736 |
|  | NP\_036662 |
|  | AAS79321 |
|  | AAC62841 |
|  | P07310 |
|  | AAP35439 |
|  | BAB32233 |
|  | P06732 |
|  | AAA52026 |
|  | BAB26603 |
|  | AAH62058 |


---

|  |  |
| --- | --- |
| Organism | Mammal |


---

|  |  |
| --- | --- |
| Location | 7 4.5 cM (Mus musculus) |
|  | chromosome 19, 19q13.2-q13.3 (Homo sapiens) |
|  | chromosome 7, 7 4.5 cM, 7 A2 (Mus musculus) |
|  | chromosome 1, 1q21 (Rattus norvegicus) |


---

|  |  |
| --- | --- |
