# Supplementary material for: Dynamic transcriptomic profiles of zebrafish gills in response to zinc supplementation
Source: BMC Genomics. 2010 Oct 11;11:553. doi: 10.1186/1471-2164-11-553 (PMC3091702; doi:10.1186/1471-2164-11-553)
Supplement: Additional file 2 — Interactive Direct Interaction Network representing the molecular interactions between zinc, copper, iron, calcium and proteins encoded by transcripts changed by zinc supplementation. Mini web-site containing index.html and hyperlinked pages in subdirectory describing a Direct Interaction Network automatically generated based on curated interactions contained within the proprietary PathwayArchitect database. Ovals represent proteins and the circles symbolize metal ions. Objects are coloured by their abundance in zebrafish at the time-point they were significantly different from the control is a scale from -4 fold (dark green) to +4 fold (dark red). Where significant differences were found at more than one time-point, the colour overlay shows expression at the first instance. Dark blue squares denote 'binding', and light blue squares 'expression'; green squares stand for 'regulation', green diamonds for 'metabolism', and green circles for 'promoter binding'. Arrow heads indicate directionality of the interaction where annotated. All nodes and edges can be further interrogated by selecting the relative area of the image. [file 1471-2164-11-553-S2.zip › PathwayArchitect Zn xs DIN/133153.html]

# PROTEIN: IL15

|  |  |
| --- | --- |
| Name | IL15 |
| Type | PROTEIN |
| Description | interleukin 15 |
| Note | The protein encoded by this gene is a cytokine that regulates T and natural killer cell activation and proliferation. This cytokine and interleukine 2 share many biological activities. They are found to bind common hematopoietin receptor subunits, and may compete for the same receptor, and thus negatively regulate each other's activity. The number of CD8+ memory cells is shown to be controlled by a balance between this cytokine and IL2. This cytokine induces the activation of JAK kinases, as well as the phosphorylation and activation of transcription activators STAT3, STAT5, and STAT6. Studies of the mouse counterpart suggested that this cytokine may increase the expression of apoptosis inhibitor BCL2L1/BCL-x(L), possibly through the transcription activation activity of STAT6, and thus prevent apoptosis. Three alternatively spliced transcript variants of this gene encoding two distinct isoforms have been reported. |
| Alias | IL-15 |
|  | MGC9721 |
|  | Il15 |
|  | IL15 |
|  | AI503618 |


---

|  |  |
| --- | --- |
| GO Component | endosome |
|  | extracellular space |
|  | extracellular region |
|  | Golgi apparatus |
|  | integral to plasma membrane |
|  | membrane fraction |


---

|  |  |
| --- | --- |
| GO ID | GO:0005615 |
|  | GO:0042102 |
|  | GO:0045580 |
|  | GO:0005126 |
|  | GO:0045062 |
|  | GO:0005887 |
|  | GO:0005576 |
|  | GO:0005624 |
|  | GO:0004871 |
|  | GO:0007515 |
|  | GO:0001866 |
|  | GO:0005125 |
|  | GO:0007165 |
|  | GO:0007267 |
|  | GO:0048535 |
|  | GO:0005768 |
|  | GO:0005794 |
|  | GO:0006955 |
|  | GO:0050691 |
|  | GO:0050778 |
|  | GO:0008284 |


---

|  |  |
| --- | --- |
| MIM | MIM:600554 |


---

|  |  |
| --- | --- |
| Connectivity | 806 |


---

|  |  |
| --- | --- |
| Entrez ID | 16168 |
|  | 3600 |
|  | 25670 |


---

|  |  |
| --- | --- |
| Agilent ID | A\_53\_P167105 |
|  | A\_43\_P11728 |
|  | A\_14\_P113197 |
|  | A\_52\_P383066 |
|  | A\_53\_P171122 |
|  | A\_51\_P340788 |
|  | A\_14\_P123025 |
|  | A\_53\_P166107 |
|  | A\_14\_P131117 |
|  | A\_52\_P15461 |
|  | A\_23\_P29953 |
|  | A\_44\_P222004 |


---

|  |  |
| --- | --- |
| Cellular Localization | Extracellular region |
|  | Golgi apparatus |
|  | Cytoplasm |
|  | Organelle |
|  | Plasma membrane |
|  | Cell |
|  | Membrane |


---

|  |  |
| --- | --- |
| DbXref | KEGG pathway##04630##Jak-STAT signaling pathway##http://www.genome.jp/dbget-bin/show\_pathway?mmu04630+16168 |
|  | KEGG pathway##04060##Cytokine-cytokine receptor interaction##http://www.genome.jp/dbget-bin/show\_pathway?mmu04060+16168 |
|  | KEGG pathway##04630##Jak-STAT signaling pathway##http://www.genome.jp/dbget-bin/show\_pathway?rno04630+25670 |
|  | KEGG pathway##04630##Jak-STAT signaling pathway##http://www.genome.jp/dbget-bin/show\_pathway?hsa04630+3600 |
|  | KEGG pathway##04060##Cytokine-cytokine receptor interaction##http://www.genome.jp/dbget-bin/show\_pathway?hsa04060+3600 |
|  | KEGG pathway##04060##Cytokine-cytokine receptor interaction##http://www.genome.jp/dbget-bin/show\_pathway?rno04060+25670 |


---

|  |  |
| --- | --- |
| Pathway | Zn xs inventory |
|  | Zn xs DIN |


---

|  |  |
| --- | --- |
| GO Process | regulation of antiviral response by host |
|  | NK T cell proliferation |
|  | immune response |
|  | regulation of T cell differentiation |
|  | positive regulation of immune response |
|  | cell-cell signaling |
|  | signal transduction |
|  | lymph gland development |
|  | positive regulation of T cell proliferation |
|  | positive regulation of cell proliferation |
|  | extrathymic T cell selection |
|  | lymph node development |


---

|  |  |
| --- | --- |
| UniGene | Mm.4392 |
|  | Hs.311958 |
|  | Rn.2490 |


---

|  |  |
| --- | --- |
| Affymetrix Probeset ID | 1036\_at |
|  | 1368375\_a\_at |
|  | 1418219\_at |
|  | 161037\_at |
|  | 205992\_s\_at |
|  | 217371\_s\_at |
|  | 38488\_s\_at |
|  | AF015719\_s\_at |
|  | g10835152\_3p\_x\_at |
|  | Hs.168132.2.S1\_3p\_a\_at |
|  | U14332\_s\_at |
|  | U14407\_at |
|  | U69272\_at |
|  | U69272\_g\_at |
|  | 217372\_at |
|  | 92651\_at |


---

|  |  |
| --- | --- |
| GO Function | hematopoietin/interferon-class (D200-domain) cytokine receptor binding |
|  | cytokine activity |
|  | signal transducer activity |


---

|  |  |
| --- | --- |
| Nucleotide | X94223 |
|  | U69272 |
|  | BC018149 |
|  | AB022307 |
|  | AF176907 |
|  | NM\_008357 |
|  | Z38000 |
|  | AF015719 |
|  | BC100961 |
|  | X94222 |
|  | AK155616 |
|  | NM\_172174 |
|  | AK122993 |
|  | AJ012587 |
|  | U14407 |
|  | AK085441 |
|  | CR541980 |
|  | NM\_172175 |
|  | X91233 |
|  | U14332 |
|  | NM\_000585 |
|  | BC100962 |
|  | AF015718 |
|  | AY720442 |
|  | BC023698 |
|  | NM\_013129 |
|  | CR542007 |
|  | AF031167 |
|  | BC100963 |


---

|  |  |
| --- | --- |
| Protein | AAI00964 |
|  | NP\_032383 |
|  | AAH18149 |
|  | CAA10069 |
|  | AAB94536 |
|  | NP\_000576 |
|  | CAG46777 |
|  | AAA21551 |
|  | P40933 |
|  | CAA63914 |
|  | AAB94535 |
|  | AAI00962 |
|  | CAA86100 |
|  | AAB97518 |
|  | AAU21241 |
|  | AAB41697 |
|  | NP\_751914 |
|  | CAA63913 |
|  | P48346 |
|  | NP\_037261 |
|  | CAG46804 |
|  | BAE33347 |
|  | NP\_751915 |
|  | AAA75377 |
|  | AAI00963 |
|  | P97604 |
|  | AAH23698 |
|  | BAA37122 |
|  | CAA62616 |


---

|  |  |
| --- | --- |
| Organism | Mammal |


---

|  |  |
| --- | --- |
| Location | chromosome 8, 8 38.0 cM, 8 C2 (Mus musculus) |
|  | 8 38.0 cM (Mus musculus) |
|  | chromosome 19, 19q11 (Rattus norvegicus) |
|  | chromosome 4, 4q31 (Homo sapiens) |


---

|  |  |
| --- | --- |
