# Supplementary material for: Dynamic transcriptomic profiles of zebrafish gills in response to zinc supplementation
Source: BMC Genomics. 2010 Oct 11;11:553. doi: 10.1186/1471-2164-11-553 (PMC3091702; doi:10.1186/1471-2164-11-553)
Supplement: Additional file 2 — Interactive Direct Interaction Network representing the molecular interactions between zinc, copper, iron, calcium and proteins encoded by transcripts changed by zinc supplementation. Mini web-site containing index.html and hyperlinked pages in subdirectory describing a Direct Interaction Network automatically generated based on curated interactions contained within the proprietary PathwayArchitect database. Ovals represent proteins and the circles symbolize metal ions. Objects are coloured by their abundance in zebrafish at the time-point they were significantly different from the control is a scale from -4 fold (dark green) to +4 fold (dark red). Where significant differences were found at more than one time-point, the colour overlay shows expression at the first instance. Dark blue squares denote 'binding', and light blue squares 'expression'; green squares stand for 'regulation', green diamonds for 'metabolism', and green circles for 'promoter binding'. Arrow heads indicate directionality of the interaction where annotated. All nodes and edges can be further interrogated by selecting the relative area of the image. [file 1471-2164-11-553-S2.zip › PathwayArchitect Zn xs DIN/134273.html]

# PROTEIN: POLR2E

|  |  |
| --- | --- |
| Name | POLR2E |
| Type | PROTEIN |
| Description | polymerase (RNA) II (DNA directed) polypeptide E, 25kDa |
| Note | This gene encodes the fifth largest subunit of RNA polymerase II, the polymerase responsible for synthesizing messenger RNA in eukaryotes. This subunit is shared by the other two DNA-directed RNA polymerases and is present in two-fold molar excess over the other polymerase subunits. An interaction between this subunit and a hepatitis virus transactivating protein has been demonstrated, suggesting that interaction between transcriptional activators and the polymerase can occur through this subunit. A pseudogene is located on chromosome 11. |
| Alias | hsRPB5 |
|  | XAP4 |
|  | AW208866 |
|  | polymerase (RNA) II (DNA directed) polypeptide E (25kDa) |
|  | RPABC1 |
|  | DNA directed RNA polymerase II 23 kda polypeptide |
|  | hRPB25 |
|  | Polr2e |
|  | 25kDa |
|  | DNA directed RNA polymerase II polypeptide E |
|  | POLR2E |
|  | RPB25 |
|  | polymerase (RNA) II (DNA directed) polypeptide E (25kD) |
|  | RPB5 |
|  | 2410021N14Rik |


---

|  |  |
| --- | --- |
| GO Component | nucleus |
|  | DNA-directed RNA polymerase II, core complex |


---

|  |  |
| --- | --- |
| GO ID | GO:0005665 |
|  | GO:0003899 |
|  | GO:0005634 |
|  | GO:0003677 |
|  | GO:0005515 |
|  | GO:0006350 |
|  | GO:0016779 |
|  | GO:0016740 |
|  | GO:0006366 |


---

|  |  |
| --- | --- |
| MIM | MIM:180664 |


---

|  |  |
| --- | --- |
| Connectivity | 21 |


---

|  |  |
| --- | --- |
| Entrez ID | 66420 |
|  | 5434 |


---

|  |  |
| --- | --- |
| Agilent ID | A\_14\_P139676 |
|  | A\_52\_P362033 |
|  | A\_52\_P973604 |
|  | A\_51\_P239456 |
|  | A\_23\_P165280 |


---

|  |  |
| --- | --- |
| Cellular Localization | Nucleus |
|  | Organelle |
|  | Cell |


---

|  |  |
| --- | --- |
| DbXref | KEGG pathway##03020##RNA polymerase##http://www.genome.jp/dbget-bin/show\_pathway?mmu03020+66420 |
|  | Reactome##73894##DNA Repair##http://www.reactome.org/cgi-bin/eventbrowser?DB=gk\_current&ID=73894 |
|  | KEGG pathway##00240##Pyrimidine metabolism##http://www.genome.jp/dbget-bin/show\_pathway?mmu00240+66420 |
|  | Reactome##112155##RNA Polymerase III Simple Start Sequence Initiation At Type 2 Promoters##http://www.reactome.org/cgi-bin/eventbrowser?DB=gk\_current&ID=112155 |
|  | KEGG pathway##00240##Pyrimidine metabolism##http://www.genome.jp/dbget-bin/show\_pathway?hsa00240+5434 |
|  | Reactome##113409##Abortive termination of early transcription elongation by DSIF##http://www.reactome.org/cgi-bin/eventbrowser?DB=gk\_current&ID=113409 |
|  | Reactome##112386##Pausing and recovery of elongation##http://www.reactome.org/cgi-bin/eventbrowser?DB=gk\_current&ID=112386 |
|  | Reactome##112156##RNA Polymerase III Simple Start Sequence Initiation At Type 3 Promoters##http://www.reactome.org/cgi-bin/eventbrowser?DB=gk\_current&ID=112156 |
|  | Reactome##112054##RNA Polymerase III Abortive Initiation At Type 3 Open Promoters##http://www.reactome.org/cgi-bin/eventbrowser?DB=gk\_current&ID=112054 |
|  | Reactome##75862##Fall Back to Closed Pre-initiation Complex##http://www.reactome.org/cgi-bin/eventbrowser?DB=gk\_current&ID=75862 |
|  | Reactome##74159##Transcription##http://www.reactome.org/cgi-bin/eventbrowser?DB=gk\_current&ID=74159 |
|  | KEGG pathway##00230##Purine metabolism##http://www.genome.jp/dbget-bin/show\_pathway?mmu00230+66420 |
|  | KEGG pathway##03020##RNA polymerase##http://www.genome.jp/dbget-bin/show\_pathway?hsa03020+5434 |
|  | Reactome##112055##RNA Polymerase III Abortive Initiation At Type 1 Open Promoters##http://www.reactome.org/cgi-bin/eventbrowser?DB=gk\_current&ID=112055 |
|  | Reactome##75071##mRNA Processing##http://www.reactome.org/cgi-bin/eventbrowser?DB=gk\_current&ID=75071 |
|  | Reactome##113451##Resumption of RNA Polymerase III Productive Transcription##http://www.reactome.org/cgi-bin/eventbrowser?DB=gk\_current&ID=113451 |
|  | Reactome##113429##Elongating transcript encounters a lesion in the template##http://www.reactome.org/cgi-bin/eventbrowser?DB=gk\_current&ID=113429 |
|  | Reactome##74160##Gene Expression##http://www.reactome.org/cgi-bin/eventbrowser?DB=gk\_current&ID=74160 |
|  | Reactome##73946##Abortive initiation##http://www.reactome.org/cgi-bin/eventbrowser?DB=gk\_current&ID=73946 |
|  | Reactome##112149##RNA Polymerase III Abortive Initiation At Type 2 Open Promoters##http://www.reactome.org/cgi-bin/eventbrowser?DB=gk\_current&ID=112149 |
|  | Reactome##112153##RNA Polymerase III Simple Start Sequence Initiation At Type 1 Promoters##http://www.reactome.org/cgi-bin/eventbrowser?DB=gk\_current&ID=112153 |
|  | Reactome##113442##RNA Polymerase III Retractive RNase Activity at U-tract Pause Sites##http://www.reactome.org/cgi-bin/eventbrowser?DB=gk\_current&ID=113442 |
|  | Reactome##75891##Abortive Initiation After Second Transition##http://www.reactome.org/cgi-bin/eventbrowser?DB=gk\_current&ID=75891 |
|  | Reactome##75856##Abortive Initiation Before Second Transition##http://www.reactome.org/cgi-bin/eventbrowser?DB=gk\_current&ID=75856 |
|  | KEGG pathway##00230##Purine metabolism##http://www.genome.jp/dbget-bin/show\_pathway?hsa00230+5434 |


---

|  |  |
| --- | --- |
| Pathway | Zn xs inventory |
|  | Zn xs DIN |


---

|  |  |
| --- | --- |
| GO Process | transcription |
|  | transcription from RNA polymerase II promoter |


---

|  |  |
| --- | --- |
| UniGene | Hs.24301 |
|  | Mm.18579 |


---

|  |  |
| --- | --- |
| Affymetrix Probeset ID | 1417138\_s\_at |
|  | 1451093\_at |
|  | 1458326\_at |
|  | 165236\_f\_at |
|  | 165866\_f\_at |
|  | 213887\_s\_at |
|  | 217614\_3p\_at |
|  | 217614\_at |
|  | 217854\_s\_at |
|  | 41332\_at |
|  | 67567\_at |
|  | 84752\_r\_at |
|  | 93325\_at |
|  | D38251\_s\_at |
|  | g4505944\_3p\_at |
|  | Hs.165728.0.A1\_3p\_x\_at |
|  | Hs.24301.1.S1\_3p\_a\_at |
|  | 109287\_f\_at |
|  | 130260\_f\_at |
|  | TC17040\_at |
|  | TC17040\_g\_at |
|  | TC38412\_at |


---

|  |  |
| --- | --- |
| EC Number | EC 2.7.7.6 |


---

|  |  |
| --- | --- |
| GO Function | protein binding |
|  | DNA-directed RNA polymerase activity |
|  | transferase activity |
|  | DNA binding |
|  | nucleotidyltransferase activity |


---

|  |  |
| --- | --- |
| Nucleotide | BC026842 |
|  | AW208866 |
|  | NM\_002695 |
|  | AK132109 |
|  | BC004441 |
|  | BC045521 |
|  | BC005599 |
|  | D38251 |
|  | AK155368 |
|  | BC037681 |
|  | AK088610 |
|  | AK010567 |
|  | S42643 |
|  | AK015823 |
|  | AI845735 |
|  | BC034144 |
|  | AC004151 |
|  | J04965 |
|  | AK144668 |
|  | AK122813 |
|  | AK145201 |
|  | XM\_282920 |


---

|  |  |
| --- | --- |
| Protein | NP\_002686 |
|  | BAE33223 |
|  | Q80UW8 |
|  | AAB19339 |
|  | BAE43245 |
|  | XP\_282920 |
|  | BAE26292 |
|  | AAC03238 |
|  | AAH37681 |
|  | AAH26842 |
|  | AAH34144 |
|  | BAA07406 |
|  | P19388 |
|  | AAH04441 |
|  | AAH45521 |
|  | BAE25998 |
|  | BAE20985 |
|  | AAA62401 |


---

|  |  |
| --- | --- |
| Organism | Mammal |


---

|  |  |
| --- | --- |
| Location | chromosome 19, 19p13.3 (Homo sapiens) |
|  | chromosome 10, 10 C1 (Mus musculus) |


---

|  |  |
| --- | --- |
