# Supplementary material for: Dynamic transcriptomic profiles of zebrafish gills in response to zinc supplementation
Source: BMC Genomics. 2010 Oct 11;11:553. doi: 10.1186/1471-2164-11-553 (PMC3091702; doi:10.1186/1471-2164-11-553)
Supplement: Additional file 2 — Interactive Direct Interaction Network representing the molecular interactions between zinc, copper, iron, calcium and proteins encoded by transcripts changed by zinc supplementation. Mini web-site containing index.html and hyperlinked pages in subdirectory describing a Direct Interaction Network automatically generated based on curated interactions contained within the proprietary PathwayArchitect database. Ovals represent proteins and the circles symbolize metal ions. Objects are coloured by their abundance in zebrafish at the time-point they were significantly different from the control is a scale from -4 fold (dark green) to +4 fold (dark red). Where significant differences were found at more than one time-point, the colour overlay shows expression at the first instance. Dark blue squares denote 'binding', and light blue squares 'expression'; green squares stand for 'regulation', green diamonds for 'metabolism', and green circles for 'promoter binding'. Arrow heads indicate directionality of the interaction where annotated. All nodes and edges can be further interrogated by selecting the relative area of the image. [file 1471-2164-11-553-S2.zip › PathwayArchitect Zn xs DIN/134385.html]

# PROTEIN: DNMT3B

|  |  |
| --- | --- |
| Name | DNMT3B |
| Type | PROTEIN |
| Description | DNA (cytosine-5-)-methyltransferase 3 beta |
| Note | CpG methylation is an epigenetic modification that is important for embryonic development, imprinting, and X-chromosome inactivation. Studies in mice have demonstrated that DNA methylation is required for mammalian development. This gene encodes a DNA methyltransferase which is thought to function in de novo methylation, rather than maintenance methylation. The protein localizes primarily to the nucleus and its expression is developmentally regulated. Mutations in this gene cause the immunodeficiency-centromeric instability-facial anomalies (ICF) syndrome. Six alternatively spliced transcript variants have been described. The full length sequences of variants 4 and 5 have not been determined. |
| Alias | ICF |
|  | Dnmt3b |
|  | DNMT3B |
|  | M.HsaIIIB |
|  | MGC124407 |
|  | M.MmuIIIB |
|  | DNA cytosine-5 methyltransferase 3 beta |
|  | DNA MTase HsaIIIB |
|  | DNA MTase MmuIIIB |
|  | DNA methyltransferase HsaIIIB |
|  | DNA methyltransferase MmuIIIB |


---

|  |  |
| --- | --- |
| GO Component | nuclear heterochromatin |
|  | nucleus |
|  | heterochromatin |


---

|  |  |
| --- | --- |
| GO ID | GO:0005634 |
|  | GO:0003677 |
|  | GO:0006349 |
|  | GO:0008168 |
|  | GO:0008326 |
|  | GO:0000067 |
|  | GO:0006306 |
|  | GO:0046872 |
|  | GO:0005515 |
|  | GO:0007275 |
|  | GO:0003886 |
|  | GO:0016740 |
|  | GO:0000792 |
|  | GO:0005720 |


---

|  |  |
| --- | --- |
| MIM | MIM:602900 |
|  | MIM:242860 |


---

|  |  |
| --- | --- |
| Connectivity | 98 |


---

|  |  |
| --- | --- |
| Entrez ID | 444985 |
|  | 13436 |
|  | 1789 |


---

|  |  |
| --- | --- |
| Agilent ID | A\_53\_P112088 |
|  | A\_51\_P341540 |
|  | A\_44\_P287958 |
|  | A\_53\_P177869 |
|  | A\_23\_P28953 |
|  | A\_14\_P103258 |
|  | A\_43\_P23231 |
|  | A\_53\_P119191 |
|  | A\_14\_P138075 |
|  | A\_14\_P119979 |


---

|  |  |
| --- | --- |
| Cellular Localization | Nucleus |
|  | Chromosome |
|  | Organelle |
|  | Cell |


---

|  |  |
| --- | --- |
| DbXref | KEGG pathway##00271##Methionine metabolism##http://www.genome.jp/dbget-bin/show\_pathway?hsa00271+1789 |
|  | KEGG pathway##00271##Methionine metabolism##http://www.genome.jp/dbget-bin/show\_pathway?mmu00271+13436 |


---

|  |  |
| --- | --- |
| Pathway | Zn xs inventory |
|  | Zn xs DIN |


---

|  |  |
| --- | --- |
| GO Process | DNA replication and chromosome cycle |
|  | imprinting |
|  | development |
|  | DNA methylation |


---

|  |  |
| --- | --- |
| UniGene | Hs.251673 |
|  | Rn.117353 |
|  | Mm.89772 |


---

|  |  |
| --- | --- |
| Affymetrix Probeset ID | 100775\_at |
|  | 115066\_at |
|  | 1385662\_at |
|  | 1418351\_a\_at |
|  | 1442655\_at |
|  | 1443016\_at |
|  | 1449052\_a\_at |
|  | 1459384\_at |
|  | 164028\_at |
|  | 220668\_s\_at |
|  | 47584\_at |
|  | c81443\_rc\_at |
|  | 112717\_at |
|  | g5901939\_3p\_a\_at |
|  | RC\_AA477706\_at |
|  | TC21069\_at |
|  | TC21187\_at |


---

|  |  |
| --- | --- |
| EC Number | EC 2.1.1.37 |


---

|  |  |
| --- | --- |
| GO Function | protein binding |
|  | methyltransferase activity |
|  | transferase activity |
|  | DNA binding |
|  | DNA (cytosine-5-)-methyltransferase activity |
|  | site-specific DNA-methyltransferase (cytosine-specific) activity |
|  | metal ion binding |


---

|  |  |
| --- | --- |
| Nucleotide | AF176228 |
|  | AK001191 |
|  | NM\_175849 |
|  | AF068626 |
|  | AF151975 |
|  | BN000397 |
|  | AL035071 |
|  | NM\_001003963 |
|  | NM\_006892 |
|  | AY078427 |
|  | AF151969 |
|  | AF151974 |
|  | NM\_001003961 |
|  | AF331857 |
|  | AF068628 |
|  | AF151971 |
|  | NM\_175848 |
|  | AF129269 |
|  | AF129267 |
|  | AF156487 |
|  | NM\_175850 |
|  | AF156488 |
|  | NM\_001003959 |
|  | AF129268 |
|  | AF068627 |
|  | AF151972 |
|  | AI550177 |
|  | AI550146 |
|  | AF151973 |
|  | NM\_010068 |
|  | NM\_001003960 |
|  | AF151976 |
|  | AF151970 |
|  | AB208880 |
|  | BC105922 |


---

|  |  |
| --- | --- |
| Protein | O88509 |
|  | AAD53063 |
|  | AAC40180 |
|  | AAF74520 |
|  | CAB53071 |
|  | AAL85481 |
|  | CAE52319 |
|  | NP\_001003961 |
|  | NP\_787046 |
|  | AAC40179 |
|  | BAD92117 |
|  | AAF74522 |
|  | AAC40178 |
|  | NP\_001003960 |
|  | AAL57040 |
|  | Q9UBC3 |
|  | AAF74517 |
|  | AAF74518 |
|  | AAD31433 |
|  | NP\_787045 |
|  | CAB53069 |
|  | NP\_008823 |
|  | NP\_001003959 |
|  | AAF74519 |
|  | NP\_034198 |
|  | NP\_001003963 |
|  | AAF74516 |
|  | AAF74515 |
|  | AAD53062 |
|  | NP\_787044 |
|  | CAB53070 |
|  | AAD31434 |
|  | AAD31432 |
|  | AAF04015 |
|  | AAF74521 |
|  | AAI05923 |


---

|  |  |
| --- | --- |
| Organism | Mammal |


---

|  |  |
| --- | --- |
| Location | chromosome 20, 20q11.2 (Homo sapiens) |
|  | chromosome 3, 3q41 (Rattus norvegicus) |
|  | chromosome 2, 2 A2-A3 (Mus musculus) |


---

|  |  |
| --- | --- |
