# Supplementary material for: Dynamic transcriptomic profiles of zebrafish gills in response to zinc supplementation
Source: BMC Genomics. 2010 Oct 11;11:553. doi: 10.1186/1471-2164-11-553 (PMC3091702; doi:10.1186/1471-2164-11-553)
Supplement: Additional file 2 — Interactive Direct Interaction Network representing the molecular interactions between zinc, copper, iron, calcium and proteins encoded by transcripts changed by zinc supplementation. Mini web-site containing index.html and hyperlinked pages in subdirectory describing a Direct Interaction Network automatically generated based on curated interactions contained within the proprietary PathwayArchitect database. Ovals represent proteins and the circles symbolize metal ions. Objects are coloured by their abundance in zebrafish at the time-point they were significantly different from the control is a scale from -4 fold (dark green) to +4 fold (dark red). Where significant differences were found at more than one time-point, the colour overlay shows expression at the first instance. Dark blue squares denote 'binding', and light blue squares 'expression'; green squares stand for 'regulation', green diamonds for 'metabolism', and green circles for 'promoter binding'. Arrow heads indicate directionality of the interaction where annotated. All nodes and edges can be further interrogated by selecting the relative area of the image. [file 1471-2164-11-553-S2.zip › PathwayArchitect Zn xs DIN/1345414.html]

# REGULATION:

|  |  |
| --- | --- |
| Type | REGULATION |
| Effect | None |


---

|  |  |
| --- | --- |
| Score | 0 |


---

|  |  |
| --- | --- |
| Reference Count | 4 |


---

|  |  |
| --- | --- |
| Mechanism | Unknown |


---

|  |  |
| --- | --- |
| Reference:0 || Sentence | "These findings, coupled with the existence of AP-1 consensus DNA binding sites on the collagenase gene promoter, show that regulation of collagenase gene expression by IL-1beta involves the transcription factor AP-1 in gingival fibroblasts." |
| PMID | 11052811 |
| Year | 2000 |
| Species | Human |
| Journal | Cytokine |
| RefScore | 1 |
| Source | PArchNLP |
  |
|


---

|  |  |
| --- | --- |
 Reference:1 || Sentence | Reporter assays combined with deletion mutagenesis analysis and gel shift assays showed the involvement of activator protein 1 in the activation of MMP-2 promoter activity by Bcl-2. |
| PMID | 15994927 |
| Year | 2005 |
| Species | Human |
|  | Mouse |
| Journal | Cancer Res |
| RefScore | 2 |
| Source | PArchNLP |
  ||


---

|  |  |
| --- | --- |
 Reference:2 || Sentence | Tetraspanin CD9 induces MMP-2 expression by activating p38 MAPK, JNK and c-Jun pathways in human melanoma cells. |
| PMID | 16000878 |
| Year | 2005 |
| Species | Human |
| Journal | Exp Mol Med |
| RefScore | 2 |
| Source | PArchNLP |
  ||


---

|  |  |
| --- | --- |
 Reference:3 || Sentence | These results suggest that CD9 induces MMP-2 expression by activating c- Jun through p38 MAPK and JNK signaling pathways in human melanoma cells. |
| PMID | 16000878 |
| Year | 2005 |
| Species | Human |
| Journal | Exp Mol Med |
| RefScore | 1 |
| Source | PArchNLP |
  |


---

|  |  |
| --- | --- |
