# Supplementary material for: Dynamic transcriptomic profiles of zebrafish gills in response to zinc supplementation
Source: BMC Genomics. 2010 Oct 11;11:553. doi: 10.1186/1471-2164-11-553 (PMC3091702; doi:10.1186/1471-2164-11-553)
Supplement: Additional file 2 — Interactive Direct Interaction Network representing the molecular interactions between zinc, copper, iron, calcium and proteins encoded by transcripts changed by zinc supplementation. Mini web-site containing index.html and hyperlinked pages in subdirectory describing a Direct Interaction Network automatically generated based on curated interactions contained within the proprietary PathwayArchitect database. Ovals represent proteins and the circles symbolize metal ions. Objects are coloured by their abundance in zebrafish at the time-point they were significantly different from the control is a scale from -4 fold (dark green) to +4 fold (dark red). Where significant differences were found at more than one time-point, the colour overlay shows expression at the first instance. Dark blue squares denote 'binding', and light blue squares 'expression'; green squares stand for 'regulation', green diamonds for 'metabolism', and green circles for 'promoter binding'. Arrow heads indicate directionality of the interaction where annotated. All nodes and edges can be further interrogated by selecting the relative area of the image. [file 1471-2164-11-553-S2.zip › PathwayArchitect Zn xs DIN/1346268.html]

# TRANSPORT:

|  |  |
| --- | --- |
| Type | TRANSPORT |
| Effect | None |


---

|  |  |
| --- | --- |
| Score | 0 |


---

|  |  |
| --- | --- |
| Reference Count | 5 |


---

|  |  |
| --- | --- |
| Mechanism | Unknown |


---

|  |  |
| --- | --- |
| Reference:0 || Sentence | "Since S100A1 protein is differentially expressed in fast- and slow-twitch skeletal muscle, we used saponin-skinned murine Musculus extensor digitorum longus (EDL) and Musculus soleus (Soleus) fibers to assess the impact of S100A1 protein on SR Ca2+ release and isometric twitch force in functionally intact permeabilized muscle fibers." |
| PMID | 12721284 |
| Year | 2003 |
| Species | Mouse |
|  | Human |
| Journal | J Biol Chem |
| RefScore | 2 |
| Source | PArchNLP |
  |
|


---

|  |  |
| --- | --- |
 Reference:1 || Sentence | "S100A1 equally enhanced caffeine-induced SR Ca2+ release and Ca2+-induced isometric force transients in both muscle preparations in a dose-dependent manner." |
| PMID | 12721284 |
| Year | 2003 |
| Species | Mouse |
|  | Human |
| Journal | J Biol Chem |
| RefScore | 1 |
| Source | PArchNLP |
  ||


---

|  |  |
| --- | --- |
 Reference:2 || Sentence | "Thus, our data suggest that S100A1 may serve as an endogenous enhancer of SR Ca2+ release and might therefore be of physiological relevance in the process of excitation-contraction coupling in skeletal muscle." |
| PMID | 12721284 |
| Year | 2003 |
| Species | Mouse |
|  | Human |
| Journal | J Biol Chem |
| RefScore | 0 |
| Source | PArchNLP |
  ||


---

|  |  |
| --- | --- |
 Reference:3 || Sentence | "In our setting S100A1 gene transfer increased FS% by 55%, systolic Ca2+ amplitudes by 62%, while S100A1 protein increased SERCA activity by 28%." |
| PMID | 12479236 |
| Year | 2002 |
| Species | Human |
|  | Rat |
| Journal | Basic Res Cardiol |
| RefScore | 0 |
| Source | PArchNLP |
  ||


---

|  |  |
| --- | --- |
 Reference:4 || Sentence | "The Ca(2+)-binding protein S100A1 displays a tissue-specific expression pattern with highest levels in myocardium and has been shown to interact with SR-proteins regulating the Ca(2+)-induced Ca(2+)-release." |
| PMID | 8898862 |
| Year | 1996 |
| Species | Human |
| Journal | Biochim Biophys Acta |
| RefScore | 0 |
| Source | PArchNLP |
  |


---

|  |  |
| --- | --- |
