# Supplementary material for: Dynamic transcriptomic profiles of zebrafish gills in response to zinc supplementation
Source: BMC Genomics. 2010 Oct 11;11:553. doi: 10.1186/1471-2164-11-553 (PMC3091702; doi:10.1186/1471-2164-11-553)
Supplement: Additional file 2 — Interactive Direct Interaction Network representing the molecular interactions between zinc, copper, iron, calcium and proteins encoded by transcripts changed by zinc supplementation. Mini web-site containing index.html and hyperlinked pages in subdirectory describing a Direct Interaction Network automatically generated based on curated interactions contained within the proprietary PathwayArchitect database. Ovals represent proteins and the circles symbolize metal ions. Objects are coloured by their abundance in zebrafish at the time-point they were significantly different from the control is a scale from -4 fold (dark green) to +4 fold (dark red). Where significant differences were found at more than one time-point, the colour overlay shows expression at the first instance. Dark blue squares denote 'binding', and light blue squares 'expression'; green squares stand for 'regulation', green diamonds for 'metabolism', and green circles for 'promoter binding'. Arrow heads indicate directionality of the interaction where annotated. All nodes and edges can be further interrogated by selecting the relative area of the image. [file 1471-2164-11-553-S2.zip › PathwayArchitect Zn xs DIN/134639.html]

# PROTEIN: SMAD1

|  |  |
| --- | --- |
| Name | SMAD1 |
| Type | PROTEIN |
| Description | SMAD, mothers against DPP homolog 1 (Drosophila) |
| Note | The protein encoded by this gene belongs to the SMAD, a family of proteins similar to the gene products of the Drosophila gene 'mothers against decapentaplegic' (Mad) and the C. elegans gene Sma. SMAD proteins are signal transducers and transcriptional modulators that mediate multiple signaling pathways. This protein mediates the signals of the bone morphogenetic proteins (BMPs), which are involved in a range of biological activities including cell growth, apoptosis, morphogenesis, development and immune responses. In response to BMP ligands, this protein can be phosphorylated and activated by the BMP receptor kinase. The phosphorylated form of this protein forms a complex with SMAD4, which is important for its function in the transcription regulation. This protein is a target for SMAD-specific E3 ubiquitin ligases, such as SMURF1 and SMURF2, and undergoes ubiquitination and proteasome-mediated degradation. Alternatively spliced transcript variants encoding the same protein have been observed. |
| Alias | transforming growth factor-beta signaling protein 1 |
|  | Mothers-against-DPP-related-1 |
|  | MADR1 |
|  | Dwarfin-A |
|  | SMAD, mothers against DPP homolog 1 |
|  | Madr1 |
|  | JV41 |
|  | JV4-1 |
|  | Dwf-A |
|  | MADH1 |
|  | Madh1 |
|  | Mad-related protein 1 |
|  | AI528653 |
|  | Mlp1 |
|  | MAD (mothers against decapentaplegic, Drosophila) homolog 1 |
|  | MAD homolog 1 (Drosophila) |
|  | Mothers against DPP homolog 1 |
|  | BSP1 |
|  | MAD homolog1 (mothers against decapentaplegic Drosophila) |
|  | mothers against DPP homolog 1 |
|  | TGF-beta signaling protein 1 |
|  | MusMLP |
|  | Sma- and Mad-related protein 1 |
|  | mMad1 |
|  | Smad 1 |
|  | MAD homolog 1 |
|  | Smad1 |
|  | SMAD 1 |
|  | MAD, mothers against decapentaplegic homolog 1 |
|  | MAD, mothers against decapentaplegic homolog 1 (Drosophila) |
|  | MAD homolog1 (mothers against decapentaplegic, Drosophila) |
|  | MAD (mothers against decapentaplegic Drosophila) homolog 1 |


---

|  |  |
| --- | --- |
| GO Component | integral to membrane |
|  | transcription factor complex |
|  | intracellular |
|  | nucleus |


---

|  |  |
| --- | --- |
| GO ID | GO:0030901 |
|  | GO:0006355 |
|  | GO:0007183 |
|  | GO:0007179 |
|  | GO:0006357 |
|  | GO:0005515 |
|  | GO:0009880 |
|  | GO:0005667 |
|  | GO:0016021 |
|  | GO:0003700 |
|  | GO:0007276 |
|  | GO:0008285 |
|  | GO:0000165 |
|  | GO:0005634 |
|  | GO:0042592 |
|  | GO:0007165 |
|  | GO:0005057 |
|  | GO:0007182 |
|  | GO:0005622 |
|  | GO:0030509 |
|  | GO:0003702 |
|  | GO:0016563 |
|  | GO:0009887 |
|  | GO:0045944 |
|  | GO:0006350 |
|  | GO:0030902 |


---

|  |  |
| --- | --- |
| MIM | MIM:601595 |


---

|  |  |
| --- | --- |
| Connectivity | 495 |


---

|  |  |
| --- | --- |
| Entrez ID | 25671 |
|  | 17125 |
|  | 4086 |


---

|  |  |
| --- | --- |
| Agilent ID | A\_23\_P212870 |
|  | A\_52\_P65108 |
|  | A\_14\_P103768 |
|  | A\_53\_P160861 |
|  | A\_42\_P668072 |
|  | A\_51\_P409260 |
|  | A\_24\_P71938 |
|  | A\_53\_P143394 |
|  | A\_14\_P111144 |
|  | A\_14\_P138790 |
|  | A\_44\_P419162 |


---

|  |  |
| --- | --- |
| Cellular Localization | Nucleus |
|  | Cell |
|  | Membrane |
|  | Organelle |


---

|  |  |
| --- | --- |
| DbXref | KEGG pathway##04350##TGF-beta signaling pathway##http://www.genome.jp/dbget-bin/show\_pathway?hsa04350+4086 |
|  | KEGG pathway##04350##TGF-beta signaling pathway##http://www.genome.jp/dbget-bin/show\_pathway?rno04350+25671 |
|  | KEGG pathway##04350##TGF-beta signaling pathway##http://www.genome.jp/dbget-bin/show\_pathway?mmu04350+17125 |


---

|  |  |
| --- | --- |
| Pathway | Zn xs inventory |
|  | Zn xs DIN |


---

|  |  |
| --- | --- |
| GO Process | negative regulation of cell proliferation |
|  | common-partner SMAD protein phosphorylation |
|  | SMAD protein heteromerization |
|  | embryonic pattern specification |
|  | transforming growth factor beta receptor signaling pathway |
|  | MAPKKK cascade |
|  | positive regulation of transcription from RNA polymerase II promoter |
|  | hindbrain development |
|  | gametogenesis |
|  | regulation of transcription from RNA polymerase II promoter |
|  | regulation of transcription, DNA-dependent |
|  | transcription |
|  | signal transduction |
|  | BMP signaling pathway |
|  | organogenesis |
|  | homeostasis |
|  | midbrain development |


---

|  |  |
| --- | --- |
| UniGene | Rn.10635 |
|  | Mm.223717 |
|  | Hs.549050 |


---

|  |  |
| --- | --- |
| Affymetrix Probeset ID | 102983\_at |
|  | 102984\_g\_at |
|  | 1325\_at |
|  | 1369174\_at |
|  | 1389373\_at |
|  | 1396061\_at |
|  | 1416081\_at |
|  | 1448208\_at |
|  | 1459843\_s\_at |
|  | 208015\_at |
|  | 210993\_s\_at |
|  | 227798\_at |
|  | 37280\_at |
|  | 50875\_at |
|  | AF067727\_s\_at |
|  | AFFX-hum\_alu\_at |
|  | g1332713\_3p\_a\_at |
|  | g7661687\_3p\_at |
|  | Hs.322710.0.A1\_3p\_at |
|  | hum\_alu\_at |
|  | rc\_AI010195\_at |
|  | U59423\_at |
|  | U66478\_at |
|  | u74359\_s\_at |
|  | 244446\_at |
|  | 74647\_at |
|  | Hs.131586.0.A1\_3p\_at |
|  | 49392\_at |
|  | RC\_AA075298\_at |


---

|  |  |
| --- | --- |
| GO Function | protein binding |
|  | transcriptional activator activity |
|  | RNA polymerase II transcription factor activity |
|  | transcription factor activity |
|  | receptor signaling protein activity |


---

|  |  |
| --- | --- |
| Nucleotide | AL117396 |
|  | CB956339 |
|  | AF295766 |
|  | AF295771 |
|  | BT007386 |
|  | BC061757 |
|  | U59912 |
|  | AF295768 |
|  | U66478 |
|  | AF295764 |
|  | U74359 |
|  | U59423 |
|  | AF295763 |
|  | U54826 |
|  | AF295767 |
|  | BI772689 |
|  | AF295770 |
|  | BU163870 |
|  | BI792941 |
|  | AF295762 |
|  | BC058693 |
|  | AF067727 |
|  | AF295765 |
|  | AK158509 |
|  | AK054104 |
|  | NM\_013130 |
|  | AK084505 |
|  | U58992 |
|  | NM\_001003688 |
|  | NM\_008539 |
|  | AK017583 |
|  | BQ962660 |
|  | AF295769 |
|  | AF295772 |
|  | U57456 |
|  | AK096624 |
|  | NM\_005900 |
|  | BC001878 |
|  | BE644607 |
|  | AK034045 |


---

|  |  |
| --- | --- |
| Protein | BAC35658 |
|  | BAC39200 |
|  | NP\_032565 |
|  | BAC28558 |
|  | P70340 |
|  | NP\_005891 |
|  | NP\_037262 |
|  | Q15797 |
|  | AAH01878 |
|  | AAC52943 |
|  | BAB30820 |
|  | AAC19116 |
|  | AAB18256 |
|  | NP\_001003688 |
|  | AAG41407 |
|  | P97588 |
|  | AAB06852 |
|  | AAC50493 |
|  | AAH61757 |
|  | AAP36050 |
|  | AAC50790 |
|  | AAC52785 |
|  | AAH58693 |
|  | AAC50621 |
|  | CAB55898 |


---

|  |  |
| --- | --- |
| Organism | Mammal |


---

|  |  |
| --- | --- |
| Location | chromosome 19, 19q11 (Rattus norvegicus) |
|  | chromosome 8, 8 C1 (Mus musculus) |
|  | chromosome 4, 4q31 (Homo sapiens) |


---

|  |  |
| --- | --- |
