# Supplementary material for: Dynamic transcriptomic profiles of zebrafish gills in response to zinc supplementation
Source: BMC Genomics. 2010 Oct 11;11:553. doi: 10.1186/1471-2164-11-553 (PMC3091702; doi:10.1186/1471-2164-11-553)
Supplement: Additional file 2 — Interactive Direct Interaction Network representing the molecular interactions between zinc, copper, iron, calcium and proteins encoded by transcripts changed by zinc supplementation. Mini web-site containing index.html and hyperlinked pages in subdirectory describing a Direct Interaction Network automatically generated based on curated interactions contained within the proprietary PathwayArchitect database. Ovals represent proteins and the circles symbolize metal ions. Objects are coloured by their abundance in zebrafish at the time-point they were significantly different from the control is a scale from -4 fold (dark green) to +4 fold (dark red). Where significant differences were found at more than one time-point, the colour overlay shows expression at the first instance. Dark blue squares denote 'binding', and light blue squares 'expression'; green squares stand for 'regulation', green diamonds for 'metabolism', and green circles for 'promoter binding'. Arrow heads indicate directionality of the interaction where annotated. All nodes and edges can be further interrogated by selecting the relative area of the image. [file 1471-2164-11-553-S2.zip › PathwayArchitect Zn xs DIN/137268.html]

# PROTEIN: CDC14A

|  |  |
| --- | --- |
| Name | CDC14A |
| Type | PROTEIN |
| Description | CDC14 cell division cycle 14 homolog A (S. cerevisiae) |
| Note | The protein encoded by this gene is a member of the dual specificity protein tyrosine phosphatase family. This protein is highly similar to Saccharomyces cerevisiae Cdc14, a protein tyrosine phosphatase involved in the exit of cell mitosis and initiation of DNA replication, which suggests the role in cell cycle control. This protein has been shown to interact with and dephosphorylates tumor suppressor protein p53, and is thought to regulate the function of p53. Alternative splice of this gene results in 3 transcript variants encoding distinct isoforms. |
| Alias | CDC14a1 |
|  | hCDC14 |
|  | Cdc14 |
|  | Cdc14A1 |
|  | CDC14 cell division cycle 14 homolog A |
|  | CDC10 (cell division cycle 10, S. cerevisiae, homolog) |
|  | Cdc14a |
|  | A830059A17Rik |
|  | Cdc14A2 |
|  | CDC14A2 |
|  | cdc14 |
|  | CDC14A |
|  | CDC14 homolog A |


---

|  |  |
| --- | --- |
| GO Component | nucleus |


---

|  |  |
| --- | --- |
| GO ID | GO:0005634 |
|  | GO:0000074 |
|  | GO:0004725 |
|  | GO:0008283 |
|  | GO:0016787 |
|  | GO:0004721 |
|  | GO:0008138 |
|  | GO:0051301 |
|  | GO:0006470 |
|  | GO:0007049 |


---

|  |  |
| --- | --- |
| MIM | MIM:603504 |


---

|  |  |
| --- | --- |
| Connectivity | 15 |


---

|  |  |
| --- | --- |
| Entrez ID | 8556 |
|  | 229776 |


---

|  |  |
| --- | --- |
| Agilent ID | A\_14\_P110107 |
|  | A\_14\_P128130 |
|  | A\_14\_P102692 |
|  | A\_52\_P538145 |
|  | A\_23\_P405110 |
|  | A\_23\_P201921 |
|  | A\_14\_P131083 |
|  | A\_23\_P424472 |
|  | A\_52\_P319726 |
|  | A\_52\_P325900 |
|  | A\_52\_P16919 |
|  | A\_51\_P489560 |


---

|  |  |
| --- | --- |
| Cellular Localization | Nucleus |
|  | Organelle |
|  | Cell |


---

|  |  |
| --- | --- |
| DbXref | KEGG pathway##04110##Cell cycle##http://www.genome.jp/dbget-bin/show\_pathway?mmu04110+229776 |
|  | KEGG pathway##04110##Cell cycle##http://www.genome.jp/dbget-bin/show\_pathway?hsa04110+8556 |


---

|  |  |
| --- | --- |
| Pathway | Zn def RIN |
|  | Master Regulators |
|  | Zn xs inventory |
|  | Zn xs DIN |


---

|  |  |
| --- | --- |
| GO Process | cell proliferation |
|  | protein amino acid dephosphorylation |
|  | cell cycle |
|  | regulation of progression through cell cycle |
|  | cell division |


---

|  |  |
| --- | --- |
| UniGene | Mm.17647 |
|  | Hs.533582 |


---

|  |  |
| --- | --- |
| Affymetrix Probeset ID | 115348\_i\_at |
|  | 115349\_r\_at |
|  | 1436913\_at |
|  | 1443184\_at |
|  | 1446493\_at |
|  | 1459517\_at |
|  | 1567303\_at |
|  | 1567304\_at |
|  | 205288\_at |
|  | 210440\_s\_at |
|  | 210441\_at |
|  | 210742\_at |
|  | 210743\_s\_at |
|  | 243640\_x\_at |
|  | 40320\_at |
|  | 72539\_at |
|  | 80535\_r\_at |
|  | 87577\_at |
|  | g3136327\_3p\_a\_at |
|  | g3136327\_3p\_at |
|  | g3136329\_3p\_at |
|  | g4502696\_3p\_at |
|  | Hs2.385268.1.S1\_3p\_at |
|  | Hs.271443.0.A1\_3p\_at |
|  | RC\_AA002017\_at |
|  | RC\_AA283949\_at |
|  | TC38094\_at |


---

|  |  |
| --- | --- |
| EC Number | EC 3.1.3.16 |
|  | EC 3.1.3.48 |


---

|  |  |
| --- | --- |
| GO Function | hydrolase activity |
|  | protein tyrosine phosphatase activity |
|  | protein tyrosine/serine/threonine phosphatase activity |
|  | phosphoprotein phosphatase activity |


---

|  |  |
| --- | --- |
| Nucleotide | AF064103 |
|  | AL589990 |
|  | AK142294 |
|  | AB209857 |
|  | AK036556 |
|  | NM\_033312 |
|  | BC093918 |
|  | BC072644 |
|  | NM\_003672 |
|  | AF064102 |
|  | AI047562 |
|  | AK048250 |
|  | AK043956 |
|  | AK037269 |
|  | XM\_149387 |
|  | AK032515 |
|  | AK052560 |
|  | BC038979 |
|  | BC093916 |
|  | AF000367 |
|  | NM\_033313 |
|  | AY623111 |
|  | AF122013 |


---

|  |  |
| --- | --- |
| Protein | AAD49217 |
|  | BAD93094 |
|  | AAT38107 |
|  | AAH38979 |
|  | NP\_201569 |
|  | Q6GQT0 |
|  | Q9UNH5 |
|  | AAC16660 |
|  | AAB88277 |
|  | AAH93918 |
|  | CAH70070 |
|  | AAC16659 |
|  | BAC29476 |
|  | AAH72644 |
|  | XP\_149387 |
|  | NP\_201570 |
|  | NP\_003663 |
|  | CAH70069 |
|  | CAH70068 |
|  | AAH93916 |


---

|  |  |
| --- | --- |
| Organism | Mammal |


---

|  |  |
| --- | --- |
| Location | chromosome 3, 3 G1 (Mus musculus) |
|  | chromosome 1, 1p21 (Homo sapiens) |


---

|  |  |
| --- | --- |
