# Supplementary material for: Dynamic transcriptomic profiles of zebrafish gills in response to zinc supplementation
Source: BMC Genomics. 2010 Oct 11;11:553. doi: 10.1186/1471-2164-11-553 (PMC3091702; doi:10.1186/1471-2164-11-553)
Supplement: Additional file 2 — Interactive Direct Interaction Network representing the molecular interactions between zinc, copper, iron, calcium and proteins encoded by transcripts changed by zinc supplementation. Mini web-site containing index.html and hyperlinked pages in subdirectory describing a Direct Interaction Network automatically generated based on curated interactions contained within the proprietary PathwayArchitect database. Ovals represent proteins and the circles symbolize metal ions. Objects are coloured by their abundance in zebrafish at the time-point they were significantly different from the control is a scale from -4 fold (dark green) to +4 fold (dark red). Where significant differences were found at more than one time-point, the colour overlay shows expression at the first instance. Dark blue squares denote 'binding', and light blue squares 'expression'; green squares stand for 'regulation', green diamonds for 'metabolism', and green circles for 'promoter binding'. Arrow heads indicate directionality of the interaction where annotated. All nodes and edges can be further interrogated by selecting the relative area of the image. [file 1471-2164-11-553-S2.zip › PathwayArchitect Zn xs DIN/138105.html]

# PROTEIN: WT1

|  |  |
| --- | --- |
| Name | WT1 |
| Type | PROTEIN |
| Description | Wilms tumor 1 |
| Note | This gene encodes a transcription factor that contains four zinc-finger motifs at the C-terminus and a proline/glutamine-rich DNA-binding domain at the N-terminus. It has an essential role in the normal development of the urogenital system, and it is mutated in a small subset of patients with Wilm's tumors. Multiple transcript variants, resulting from alternative splicing at two coding exons, have been well characterized. There is also evidence for the use of non-AUG (CUG) translation initiation site upstream of, and in-frame with the first AUG, leading to additional isoforms. |
| Alias | GUD |
|  | WT33 |
|  | WAGR |
|  | WIT-2 |
|  | Wilms' tumor protein homolog |
|  | D630046I19Rik |
|  | WT1 |
|  | Wt-1 |
|  | Wt1 |


---

|  |  |
| --- | --- |
| GO Component | nucleus |
|  | cytoplasm |


---

|  |  |
| --- | --- |
| GO ID | GO:0003677 |
|  | GO:0045786 |
|  | GO:0006355 |
|  | GO:0009888 |
|  | GO:0005737 |
|  | GO:0008584 |
|  | GO:0006357 |
|  | GO:0005515 |
|  | GO:0008270 |
|  | GO:0001747 |
|  | GO:0003700 |
|  | GO:0001823 |
|  | GO:0001656 |
|  | GO:0005634 |
|  | GO:0001654 |
|  | GO:0003676 |
|  | GO:0007281 |
|  | GO:0046872 |
|  | GO:0006350 |
|  | GO:0007049 |
|  | GO:0030855 |


---

|  |  |
| --- | --- |
| MIM | MIM:194070 |
|  | MIM:607102 |
|  | MIM:256370 |
|  | MIM:194080 |
|  | MIM:194072 |
|  | MIM:136680 |


---

|  |  |
| --- | --- |
| Connectivity | 473 |


---

|  |  |
| --- | --- |
| Entrez ID | 7490 |
|  | 22431 |
|  | 24883 |


---

|  |  |
| --- | --- |
| Agilent ID | A\_43\_P12576 |
|  | A\_14\_P123086 |
|  | A\_51\_P360809 |
|  | A\_44\_P176466 |
|  | A\_52\_P673458 |
|  | A\_53\_P136459 |
|  | A\_14\_P114899 |
|  | A\_23\_P116280 |
|  | A\_53\_P100038 |


---

|  |  |
| --- | --- |
| Cellular Localization | Cytoplasm |
|  | Nucleus |
|  | Cell |
|  | Organelle |


---

|  |  |
| --- | --- |
| Pathway | Zn def RIN |
|  | Zn xs inventory |
|  | Zn xs DIN |


---

|  |  |
| --- | --- |
| GO Process | male gonad development |
|  | negative regulation of progression through cell cycle |
|  | germ cell development |
|  | tissue development |
|  | eye development (sensu Mammalia) |
|  | regulation of transcription, DNA-dependent |
|  | metanephros development |
|  | regulation of transcription from RNA polymerase II promoter |
|  | transcription |
|  | eye development |
|  | cell cycle |
|  | mesonephros development |
|  | epithelial cell differentiation |


---

|  |  |
| --- | --- |
| UniGene | Mm.246679 |
|  | Rn.92531 |
|  | Hs.555896 |


---

|  |  |
| --- | --- |
| Affymetrix Probeset ID | 135883\_at |
|  | 1369695\_at |
|  | 1425995\_s\_at |
|  | 1443221\_at |
|  | 1500\_at |
|  | 161258\_at |
|  | 1684\_s\_at |
|  | 206067\_s\_at |
|  | 216953\_s\_at |
|  | 93856\_at |
|  | g13386509\_3p\_a\_at |
|  | Hs.1145.2.S1\_3p\_a\_at |
|  | 1377223\_at |
|  | Msa.1292.0\_at |
|  | Msa.1292.0\_g\_at |
|  | rc\_AA965119\_at |
|  | S63358\_s\_at |
|  | X51630\_at |
|  | X69716\_at |
|  | RC\_AA130187\_s\_at |
|  | rc\_AA899753\_at |


---

|  |  |
| --- | --- |
| GO Function | protein binding |
|  | transcription factor activity |
|  | DNA binding |
|  | zinc ion binding |
|  | nucleic acid binding |
|  | metal ion binding |


---

|  |  |
| --- | --- |
| Nucleotide | U06486 |
|  | S61515 |
|  | AK052767 |
|  | X72314 |
|  | X69716 |
|  | NM\_024425 |
|  | NM\_031534 |
|  | NM\_000378 |
|  | BC032861 |
|  | AL049692 |
|  | AK093168 |
|  | NM\_024426 |
|  | NM\_024424 |
|  | S77896 |
|  | X74840 |
|  | U77682 |
|  | X51630 |
|  | X77549 |
|  | L25110 |
|  | NM\_144783 |
|  | S60755 |
|  | AY245105 |
|  | M30393 |
|  | AK013905 |
|  | S75264 |
|  | BC046461 |
|  | M80217 |
|  | X61631 |
|  | M55512 |


---

|  |  |
| --- | --- |
| Protein | P22561 |
|  | CAI95760 |
|  | P49952 |
|  | AAH32861 |
|  | NP\_659032 |
|  | CAC39220 |
|  | AAA40573 |
|  | CAA35956 |
|  | AAC60605 |
|  | NP\_077743 |
|  | P19544 |
|  | CAI95759 |
|  | AAB20110 |
|  | CAA51057 |
|  | CAA43819 |
|  | AAD14879 |
|  | CAI95758 |
|  | AAB33443 |
|  | NP\_113722 |
|  | NP\_077744 |
|  | NP\_000369 |
|  | AAO61088 |
|  | AAA36810 |
|  | AAH46461 |
|  | AAA62865 |
|  | NP\_077742 |
|  | AAA61299 |
|  | CAA49373 |


---

|  |  |
| --- | --- |
| Organism | Mammal |


---

|  |  |
| --- | --- |
| Location | 2 58.0 cM (Mus musculus) |
|  | chromosome 11, 11p13 (Homo sapiens) |
|  | chromosome 2, 2 58.0 cM, 2 E (Mus musculus) |
|  | chromosome 3, 3q32 (Rattus norvegicus) |


---

|  |  |
| --- | --- |
