# Supplementary material for: Dynamic transcriptomic profiles of zebrafish gills in response to zinc supplementation
Source: BMC Genomics. 2010 Oct 11;11:553. doi: 10.1186/1471-2164-11-553 (PMC3091702; doi:10.1186/1471-2164-11-553)
Supplement: Additional file 2 — Interactive Direct Interaction Network representing the molecular interactions between zinc, copper, iron, calcium and proteins encoded by transcripts changed by zinc supplementation. Mini web-site containing index.html and hyperlinked pages in subdirectory describing a Direct Interaction Network automatically generated based on curated interactions contained within the proprietary PathwayArchitect database. Ovals represent proteins and the circles symbolize metal ions. Objects are coloured by their abundance in zebrafish at the time-point they were significantly different from the control is a scale from -4 fold (dark green) to +4 fold (dark red). Where significant differences were found at more than one time-point, the colour overlay shows expression at the first instance. Dark blue squares denote 'binding', and light blue squares 'expression'; green squares stand for 'regulation', green diamonds for 'metabolism', and green circles for 'promoter binding'. Arrow heads indicate directionality of the interaction where annotated. All nodes and edges can be further interrogated by selecting the relative area of the image. [file 1471-2164-11-553-S2.zip › PathwayArchitect Zn xs DIN/138643.html]

# PROTEIN: CYP11B1

|  |  |
| --- | --- |
| Name | CYP11B1 |
| Type | PROTEIN |
| Description | cytochrome P450, family 11, subfamily B, polypeptide 1 |
| Note | This gene encodes a member of the cytochrome P450 superfamily of enzymes. The cytochrome P450 proteins are monooxygenases which catalyze many reactions involved in drug metabolism and synthesis of cholesterol, steroids and other lipids. This protein localizes to the mitochondrial inner membrane and is involved in the conversion of progesterone to cortisol in the adrenal cortex. Mutations in this gene cause congenital adrenal hyperplasia due to 11-beta-hydroxylase deficiency. Transcript variants encoding different isoforms have been noted for this gene. |
| Alias | FLJ36771 |
|  | CYP11B1 |
|  | steroid 11-beta-hydroxylase |
|  | CPN1 |
|  | CYP11B |
|  | DKFZp686B05283 |
|  | aldosterone synthase |
|  | Cp11ba |
|  | cytochrome P450, subfamily XIB (steroid 11-beta-hydroxylase), polypeptide 1 |
|  | Cyp11b-1 |
|  | P450C11 |
|  | Cytochrome P450, subfamily XIB, polypeptide 1 (steroid 11-beta-hydroxylase) |
|  | steroid 11-beta-monooxygenase |
|  | Steroid 11-beta-hydroxylase |
|  | Cytochrome P450 subfamily XIB polypeptide 1 (steroid 11-beta-hydroxylase) |
|  | CYPXIB1 |
|  | AA389061 |
|  | RATCP11BA |
|  | steroid-11-beta-hydroxylase |
|  | Cyp11b |
|  | cytochrome p450 XIB1 |
|  | S11BH |
|  | P-450c11 |
|  | FHI |


---

|  |  |
| --- | --- |
| GO Component | integral to endoplasmic reticulum membrane |
|  | mitochondrion |
|  | membrane |


---

|  |  |
| --- | --- |
| GO ID | GO:0006810 |
|  | GO:0016020 |
|  | GO:0020037 |
|  | GO:0005506 |
|  | GO:0006118 |
|  | GO:0004507 |
|  | GO:0046872 |
|  | GO:0006700 |
|  | GO:0030176 |
|  | GO:0004497 |
|  | GO:0005739 |
|  | GO:0016491 |
|  | GO:0006629 |


---

|  |  |
| --- | --- |
| MIM | MIM:202010 |
|  | MIM:103900 |


---

|  |  |
| --- | --- |
| Connectivity | 81 |


---

|  |  |
| --- | --- |
| Entrez ID | 1584 |
|  | 110115 |
|  | 24293 |


---

|  |  |
| --- | --- |
| Agilent ID | A\_14\_P136693 |
|  | A\_44\_P278418 |
|  | A\_43\_P11452 |
|  | A\_23\_P168928 |
|  | A\_24\_P329424 |


---

|  |  |
| --- | --- |
| Cellular Localization | Membrane |
|  | Mitochondrion |
|  | Cell |
|  | Cytoplasm |
|  | Endoplasmic reticulum |
|  | Organelle |


---

|  |  |
| --- | --- |
| DbXref | KEGG pathway##00140##C21-Steroid hormone metabolism##http://www.genome.jp/dbget-bin/show\_pathway?rno00140+24293 |
|  | KEGG pathway##00150##Androgen and estrogen metabolism##http://www.genome.jp/dbget-bin/show\_pathway?hsa00150+1584 |
|  | KEGG pathway##00140##C21-Steroid hormone metabolism##http://www.genome.jp/dbget-bin/show\_pathway?hsa00140+1584 |
|  | KEGG pathway##00150##Androgen and estrogen metabolism##http://www.genome.jp/dbget-bin/show\_pathway?rno00150+24293 |


---

|  |  |
| --- | --- |
| Pathway | Zn xs inventory |
|  | Zn xs DIN |


---

|  |  |
| --- | --- |
| GO Process | transport |
|  | electron transport |
|  | C21-steroid hormone biosynthesis |
|  | lipid metabolism |


---

|  |  |
| --- | --- |
| UniGene | Hs.184927 |
|  | Mm.322606 |
|  | Rn.88586 |


---

|  |  |
| --- | --- |
| Affymetrix Probeset ID | 1411\_at |
|  | 1412\_g\_at |
|  | 166619\_f\_at |
|  | 1456362\_at |
|  | 1552493\_s\_at |
|  | 214610\_at |
|  | 34548\_at |
|  | 34549\_g\_at |
|  | 79018\_at |
|  | Hs2.377912.1.S1\_3p\_s\_at |
|  | Hs.301118.0.S2\_3p\_at |
|  | M32879\_at |
|  | M32879\_s\_at |
|  | D16154\_at |
|  | 129183\_f\_at |
|  | TC36129\_at |


---

|  |  |
| --- | --- |
| EC Number | EC 1.14.15.4 |


---

|  |  |
| --- | --- |
| GO Function | steroid 11-beta-monooxygenase activity |
|  | oxidoreductase activity |
|  | monooxygenase activity |
|  | iron ion binding |
|  | heme binding |
|  | metal ion binding |


---

|  |  |
| --- | --- |
| Nucleotide | M32863 |
|  | M24667 |
|  | D10169 |
|  | X15431 |
|  | AJ431382 |
|  | AK142439 |
|  | NM\_001026213 |
|  | D16155 |
|  | D11354 |
|  | BC096287 |
|  | NM\_012537 |
|  | AJ431380 |
|  | BX647738 |
|  | M32879 |
|  | AJ431381 |
|  | BC096286 |
|  | X55764 |
|  | AA389061 |
|  | D10107 |
|  | D16153 |
|  | AK168839 |
|  | D00567 |
|  | BC096285 |
|  | NM\_000497 |
|  | AK094090 |
|  | AF478474 |
|  | AK163083 |


---

|  |  |
| --- | --- |
| Protein | BAA01039 |
|  | AAH96285 |
|  | BAA00444 |
|  | CAA39290 |
|  | NP\_000488 |
|  | AAA52148 |
|  | CAA33472 |
|  | BAA00988 |
|  | AAH96286 |
|  | BAE40662 |
|  | BAE25066 |
|  | CAD24090 |
|  | BAC04283 |
|  | P15393 |
|  | CAD24088 |
|  | NP\_001021384 |
|  | BAA03717 |
|  | BAA01957 |
|  | BAB71992 |
|  | AAA52149 |
|  | CAD24089 |
|  | AAL84813 |
|  | AAH96287 |
|  | P15538 |
|  | NP\_036669 |
|  | BAE37185 |


---

|  |  |
| --- | --- |
| Organism | Mammal |


---

|  |  |
| --- | --- |
| Location | chromosome 15, 15 44.9 cM, 15 (Mus musculus) |
|  | chromosome 7, 7q34 (Rattus norvegicus) |
|  | chromosome 8, 8q21 (Homo sapiens) |
|  | 15 44.9 cM (Mus musculus) |


---

|  |  |
| --- | --- |
