# Supplementary material for: Dynamic transcriptomic profiles of zebrafish gills in response to zinc supplementation
Source: BMC Genomics. 2010 Oct 11;11:553. doi: 10.1186/1471-2164-11-553 (PMC3091702; doi:10.1186/1471-2164-11-553)
Supplement: Additional file 2 — Interactive Direct Interaction Network representing the molecular interactions between zinc, copper, iron, calcium and proteins encoded by transcripts changed by zinc supplementation. Mini web-site containing index.html and hyperlinked pages in subdirectory describing a Direct Interaction Network automatically generated based on curated interactions contained within the proprietary PathwayArchitect database. Ovals represent proteins and the circles symbolize metal ions. Objects are coloured by their abundance in zebrafish at the time-point they were significantly different from the control is a scale from -4 fold (dark green) to +4 fold (dark red). Where significant differences were found at more than one time-point, the colour overlay shows expression at the first instance. Dark blue squares denote 'binding', and light blue squares 'expression'; green squares stand for 'regulation', green diamonds for 'metabolism', and green circles for 'promoter binding'. Arrow heads indicate directionality of the interaction where annotated. All nodes and edges can be further interrogated by selecting the relative area of the image. [file 1471-2164-11-553-S2.zip › PathwayArchitect Zn xs DIN/139067.html]

# PROTEIN: CYP2C19

|  |  |
| --- | --- |
| Name | CYP2C19 |
| Type | PROTEIN |
| Description | cytochrome P450, family 2, subfamily C, polypeptide 19 |
| Note | This gene encodes a member of the cytochrome P450 superfamily of enzymes. The cytochrome P450 proteins are monooxygenases which catalyze many reactions involved in drug metabolism and synthesis of cholesterol, steroids and other lipids. This protein localizes to the endoplasmic reticulum and is known to metabolize many xenobiotics, including the anticonvulsive drug mephenytoin, omeprazole, diazepam and some barbiturates. Polymorphism within this gene is associated with variable ability to metabolize mephenytoin, known as the poor metabolizer and extensive metabolizer phenotypes. The gene is located within a cluster of cytochrome P450 genes on chromosome 10q24. |
| Alias | cytochrome P-450 II C |
|  | CYP 2C |
|  | CYPIIC19 |
|  | (S)- limonene 7-monooxygenase |
|  | CYP2C |
|  | mephenytoin 4'-hydroxylase |
|  | OTTHUMP00000059588 |
|  | P450-254C |
|  | (S)-limonene 6-monooxygenase |
|  | S-mephenytoin 4-hydroxylase |
|  | flavoprotein-linked monooxygenase |
|  | CYPIIC17 |
|  | CYP2C19 |
|  | CPCJ |
|  | microsomal monooxygenase |
|  | (R)-limonene 6-monooxygenase |
|  | xenobiotic monooxygenase |
|  | cytochrome P450, subfamily IIC (mephenytoin 4-hydroxylase), polypeptide 19 |
|  | P450-11A |
|  | P450IIC19 |
|  | P450C2C |
|  | Mephenytoin 4- hydroxylase |


---

|  |  |
| --- | --- |
| GO Component | endoplasmic reticulum |
|  | microsome |
|  | membrane |


---

|  |  |
| --- | --- |
| GO ID | GO:0006810 |
|  | GO:0016020 |
|  | GO:0020037 |
|  | GO:0005783 |
|  | GO:0005792 |
|  | GO:0005506 |
|  | GO:0006118 |
|  | GO:0019825 |
|  | GO:0046872 |
|  | GO:0018676 |
|  | GO:0016712 |


---

|  |  |
| --- | --- |
| MIM | MIM:124020 |


---

|  |  |
| --- | --- |
| Connectivity | 345 |


---

|  |  |
| --- | --- |
| Entrez ID | 1557 |


---

|  |  |
| --- | --- |
| Agilent ID | A\_14\_P123950 |
|  | A\_23\_P158484 |
|  | A\_23\_P158481 |
|  | A\_14\_P135829 |
|  | A\_14\_P133020 |


---

|  |  |
| --- | --- |
| Cellular Localization | Membrane |
|  | Endoplasmic reticulum |
|  | Cell |
|  | Cytoplasm |
|  | Organelle |


---

|  |  |
| --- | --- |
| DbXref | KEGG pathway##00071##Fatty acid metabolism##http://www.genome.jp/dbget-bin/show\_pathway?hsa00071+1557 |
|  | KEGG pathway##00361##gamma-Hexachlorocyclohexane degradation##http://www.genome.jp/dbget-bin/show\_pathway?hsa00361+1557 |
|  | KEGG pathway##00380##Tryptophan metabolism##http://www.genome.jp/dbget-bin/show\_pathway?hsa00380+1557 |


---

|  |  |
| --- | --- |
| Pathway | Zn xs inventory |
|  | Zn xs DIN |


---

|  |  |
| --- | --- |
| GO Process | transport |
|  | electron transport |


---

|  |  |
| --- | --- |
| UniGene | Hs.282409 |


---

|  |  |
| --- | --- |
| Affymetrix Probeset ID | 1002\_f\_at |
|  | 216058\_s\_at |
|  | 34078\_s\_at |
|  | Hs.296816.0.S1\_3p\_s\_at |
|  | X65962\_s\_at |
|  | 216025\_x\_at |
|  | M61854\_s\_at |


---

|  |  |
| --- | --- |
| EC Number | EC 1.14.13.48 |
|  | EC 1.14.13.49 |
|  | EC 1.14.13.80 |


---

|  |  |
| --- | --- |
| GO Function | oxidoreductase activity, acting on paired donors, with incorporation or reduction of molecular oxygen, reduced flavin or flavoprotein as one donor, and incorporation of one atom of oxygen |
|  | iron ion binding |
|  | (S)-limonene 7-monooxygenase activity |
|  | heme binding |
|  | oxygen binding |
|  | metal ion binding |


---

|  |  |
| --- | --- |
| Nucleotide | X65962 |
|  | L39102 |
|  | AY796203 |
|  | AL133513 |
|  | NM\_000769 |
|  | AL583836 |
|  | AB113829 |
|  | L07093 |
|  | L39098 |
|  | M61854 |


---

|  |  |
| --- | --- |
| Protein | AAL31348 |
|  | AAV41877 |
|  | CAH74068 |
|  | AAB59426 |
|  | BAD02827 |
|  | AAA36660 |
|  | NP\_000760 |
|  | AAL31347 |
|  | P33261 |
|  | CAH73444 |
|  | CAA46778 |


---

|  |  |
| --- | --- |
| Organism | Mammal |


---

|  |  |
| --- | --- |
| Location | chromosome 10, 10q24.1-q24.3 (Homo sapiens) |


---

|  |  |
| --- | --- |
