# Supplementary material for: Dynamic transcriptomic profiles of zebrafish gills in response to zinc supplementation
Source: BMC Genomics. 2010 Oct 11;11:553. doi: 10.1186/1471-2164-11-553 (PMC3091702; doi:10.1186/1471-2164-11-553)
Supplement: Additional file 2 — Interactive Direct Interaction Network representing the molecular interactions between zinc, copper, iron, calcium and proteins encoded by transcripts changed by zinc supplementation. Mini web-site containing index.html and hyperlinked pages in subdirectory describing a Direct Interaction Network automatically generated based on curated interactions contained within the proprietary PathwayArchitect database. Ovals represent proteins and the circles symbolize metal ions. Objects are coloured by their abundance in zebrafish at the time-point they were significantly different from the control is a scale from -4 fold (dark green) to +4 fold (dark red). Where significant differences were found at more than one time-point, the colour overlay shows expression at the first instance. Dark blue squares denote 'binding', and light blue squares 'expression'; green squares stand for 'regulation', green diamonds for 'metabolism', and green circles for 'promoter binding'. Arrow heads indicate directionality of the interaction where annotated. All nodes and edges can be further interrogated by selecting the relative area of the image. [file 1471-2164-11-553-S2.zip › PathwayArchitect Zn xs DIN/1393895.html]

# BINDING:

|  |  |
| --- | --- |
| Type | BINDING |
| Effect | None |


---

|  |  |
| --- | --- |
| Score | 0 |


---

|  |  |
| --- | --- |
| Reference Count | 3 |


---

|  |  |
| --- | --- |
| Mechanism | Unknown |


---

|  |  |
| --- | --- |
| Reference:0 || Sentence | "In view of their identical apparent molecular weight and solubilization properties, p20, recognized by anti-T or anti-T alpha antibodies, and the 20 KDa band, recognized by anti-ras antibodies, seem to correspond to the same polypeptide. [3H] GDP and [3H] GMP-PNP binding experiments revealed the presence of guanine nucleotide binding proteins in total epimastigote crude extracts, as well as, in the soluble, detergent soluble, and particulate fractions." |
| PMID | 7670530 |
| Year | 1993 |
| Species | Mouse |
|  | Rat |
|  | Human |
| Journal | Biol Res |
| RefScore | 1 |
| Source | PArchNLP |
  |
|


---

|  |  |
| --- | --- |
 Reference:1 || Sentence | "The interdependence of the LTB4-binding membrane protein and guanine nucleotide-binding protein was suggested by the capacity of LTB4 to enhance by a maximum of 150% the binding of [3H]GMP-PNP to PMN leukocyte membranes by increasing the number, but not altering the affinity, of receptors for GMP-PNP." |
| PMID | 2836504 |
| Year | 1988 |
| Species | Human |
| Journal | J Immunol |
| RefScore | 3 |
| Source | PArchNLP |
  ||


---

|  |  |
| --- | --- |
 Reference:2 || Sentence | "Pertussis toxin, but not cholera toxin, reversed the enhancement of binding of [3H]GMP-PNP produced by LTB4." |
| PMID | 2836504 |
| Year | 1988 |
| Species | Human |
| Journal | J Immunol |
| RefScore | 1 |
| Source | PArchNLP |
  |


---

|  |  |
| --- | --- |
