# Supplementary material for: Dynamic transcriptomic profiles of zebrafish gills in response to zinc supplementation
Source: BMC Genomics. 2010 Oct 11;11:553. doi: 10.1186/1471-2164-11-553 (PMC3091702; doi:10.1186/1471-2164-11-553)
Supplement: Additional file 2 — Interactive Direct Interaction Network representing the molecular interactions between zinc, copper, iron, calcium and proteins encoded by transcripts changed by zinc supplementation. Mini web-site containing index.html and hyperlinked pages in subdirectory describing a Direct Interaction Network automatically generated based on curated interactions contained within the proprietary PathwayArchitect database. Ovals represent proteins and the circles symbolize metal ions. Objects are coloured by their abundance in zebrafish at the time-point they were significantly different from the control is a scale from -4 fold (dark green) to +4 fold (dark red). Where significant differences were found at more than one time-point, the colour overlay shows expression at the first instance. Dark blue squares denote 'binding', and light blue squares 'expression'; green squares stand for 'regulation', green diamonds for 'metabolism', and green circles for 'promoter binding'. Arrow heads indicate directionality of the interaction where annotated. All nodes and edges can be further interrogated by selecting the relative area of the image. [file 1471-2164-11-553-S2.zip › PathwayArchitect Zn xs DIN/142407.html]

# PROTEIN: GNAT1

|  |  |
| --- | --- |
| Name | GNAT1 |
| Type | PROTEIN |
| Description | guanine nucleotide binding protein (G protein), alpha transducing activity polypeptide 1 |
| Note | Transducin is a 3-subunit guanine nucleotide-binding protein (G protein) which stimulates the coupling of rhodopsin and cGMP-phoshodiesterase during visual impulses. The transducin alpha subunits in rods and cones are encoded by separate genes. This gene encodes the alpha subunit in rods. Alternative splicing of this gene results in two transcript variants. |
| Alias | guanine nucleotide binding protein, alpha transducing activity polypeptide 1 |
|  | Tralpha |
|  | GBT1 |
|  | transducin, rod-specific |
|  | Gnat-1 |
|  | Transducin alpha-1 chain |
|  | GNAT1 |
|  | transducin alpha-1 chain |
|  | transducin |
|  | Gnat1 |
|  | guanine nucleotide-binding protein G(T), alpha-1 subunit |
|  | GNATR |


---

|  |  |
| --- | --- |
| GO Component | membrane fraction |
|  | heterotrimeric G-protein complex |


---

|  |  |
| --- | --- |
| GO ID | GO:0007186 |
|  | GO:0007165 |
|  | GO:0007600 |
|  | GO:0005834 |
|  | GO:0007199 |
|  | GO:0007601 |
|  | GO:0003924 |
|  | GO:0007602 |
|  | GO:0008283 |
|  | GO:0000166 |
|  | GO:0019001 |
|  | GO:0004871 |
|  | GO:0005525 |
|  | GO:0005624 |


---

|  |  |
| --- | --- |
| MIM | MIM:139330 |


---

|  |  |
| --- | --- |
| Connectivity | 105 |


---

|  |  |
| --- | --- |
| Entrez ID | 2779 |
|  | 14685 |


---

|  |  |
| --- | --- |
| Agilent ID | A\_24\_P320036 |
|  | A\_53\_P146738 |
|  | A\_52\_P211185 |
|  | A\_14\_P114750 |
|  | A\_51\_P160858 |
|  | A\_23\_P166884 |


---

|  |  |
| --- | --- |
| Cellular Localization | Plasma membrane |
|  | Cell |
|  | Membrane |


---

|  |  |
| --- | --- |
| Pathway | Zn xs inventory |
|  | Zn xs DIN |


---

|  |  |
| --- | --- |
| GO Process | phototransduction |
|  | cell proliferation |
|  | G-protein coupled receptor protein signaling pathway |
|  | signal transduction |
|  | G-protein signaling, coupled to cGMP nucleotide second messenger |
|  | visual perception |
|  | sensory perception |


---

|  |  |
| --- | --- |
| UniGene | Mm.284853 |
|  | Hs.517978 |


---

|  |  |
| --- | --- |
| Affymetrix Probeset ID | 1460212\_at |
|  | 207514\_s\_at |
|  | 214286\_at |
|  | 34073\_s\_at |
|  | 34074\_s\_at |
|  | 36085\_at |
|  | 50484\_at |
|  | 99860\_at |
|  | AC002077\_at |
|  | g4504048\_3p\_a\_at |
|  | Hs.51147.1.S1\_3p\_a\_at |
|  | Hs.51147.1.S1\_3p\_x\_at |
|  | Msa.1247.0\_at |
|  | Msa.1247.0\_g\_at |
|  | X15088\_at |


---

|  |  |
| --- | --- |
| GO Function | GTP binding |
|  | GTPase activity |
|  | nucleotide binding |
|  | signal transducer activity |
|  | guanyl nucleotide binding |


---

|  |  |
| --- | --- |
| Nucleotide | BC095505 |
|  | AC002077 |
|  | M25507 |
|  | BC058810 |
|  | M25513 |
|  | BC022793 |
|  | X63749 |
|  | AF493908 |
|  | BC051412 |
|  | AK149254 |
|  | NM\_008140 |
|  | M25510 |
|  | M25509 |
|  | M25512 |
|  | U38504 |
|  | M25508 |
|  | NM\_000172 |
|  | M25506 |
|  | M25511 |
|  | X15088 |
|  | NM\_144499 |


---

|  |  |
| --- | --- |
| Protein | AAH58810 |
|  | P20612 |
|  | AAB54048 |
|  | AAH95505 |
|  | NP\_032166 |
|  | AAB01735 |
|  | CAA33196 |
|  | AAM12622 |
|  | AAA40473 |
|  | NP\_653082 |
|  | NP\_000163 |
|  | CAB37839 |
|  | AAH51412 |
|  | AAH22793 |
|  | P11488 |


---

|  |  |
| --- | --- |
| Organism | Mammal |


---

|  |  |
| --- | --- |
| Location | chromosome 3, 3p21 (Homo sapiens) |
|  | chromosome 9, 9 59.0 cM, 9 F1 (Mus musculus) |
|  | 9 59.0 cM (Mus musculus) |


---

|  |  |
| --- | --- |
